# An Automation Platform for the Chemoenzymatic Synthesis of Complex Sulfated and Branched Glycans

**DOI:** 10.1021/jacs.5c22181

**Published:** 2026-03-06

**Authors:** Saptashwa Chakraborty, Kyle Minder, Anthony Robert Prudden, Geert-Jan Boons

**Affiliations:** † Complex Carbohydrate Research Center, 1355 University of Georgia, Athens, Georgia 30602, United States; ‡ Department of Chemistry, 1355 University of Georgia, Athens, Georgia 30602, United States; § Chemical Biology and Drug Discovery, Utrecht Institute for Pharmaceutical Sciences, and Bijvoet Center for Biomolecular Research, Utrecht University, 3584 CG Utrecht, The Netherlands

## Abstract

Diverse collections of well-defined glycans are needed to investigate the molecular mechanisms by which these biomolecules mediate biological and disease processes. Several automation approaches have been introduced to accelerate the enzymatic synthesis of complex glycans. These methodologies have, however, provided only relatively simple oligosaccharides due to limitations of glycosyl transferase selectivity. Here, we describe an automation platform that makes it possible, for the first time, to prepare in an automated fashion sulfated polylactosamines and asymmetric multiantennary complex *N*-glycans via sequential enzymatic and chemical reaction cycles. It integrates glycosyltransferase catalyzed glycosylations, the use of the unnatural sugar nucleotide donor 5′-diphosphate-2-deoxy-2-trifluoro-*N*-acetamido-glucose (UDP-GlcNHTFA), and chemical manipulations including base-mediated trifluoroacetamido (TFA) removal, azido transfer and azido reduction, *tert*-butyloxycarbonyl (Boc) protection, acid mediated deprotection, and amine acylation. The latter transformations are important for *stop-and-go* chemoenzymatic synthetic strategies in which unnatural monosaccharides are introduced to temporarily disable specific sites from enzymatic modification. It is shown that, due to the modular architecture of glycans, a limited number of glycosyl transferases can provide access to large numbers of structurally diverse glycans. In this study, only 11 recombinant human glycosyl- and sulfo transferases were employed to prepare highly complex glycans. Removal of the Nap tag can be performed by hydrogenation to give oligosaccharides that are ready for microarray printing or bioconjugation.

## Graphical abstract

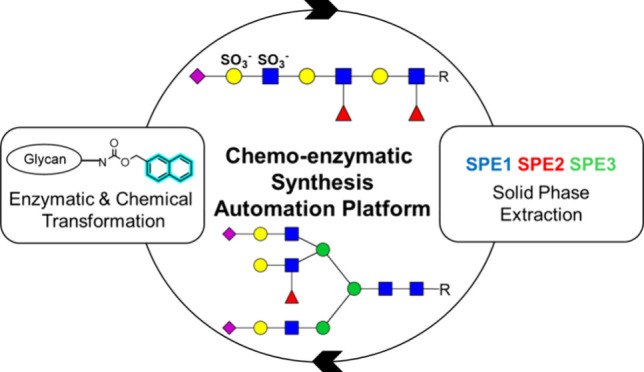

## Introduction

Almost all eukaryotic cell surfaces and secreted proteins are modified by complex carbohydrates. These biomolecules, which are also known as glycans, are expressed in tissue- and cell-specific manners and can differ considerably even between closely related organisms.^1^ Glycans are key regulators of many biological processes such as protein folding, cell–cell communication, immune regulation, fertilization, neuronal development, and hormone activity.^2^ Aberrant glycosylation has been implicated in many diseases, and examples include inflammation, autoimmunity, allergies, and cancer.^3^ Many pathogens have proteins on their surface that can bind glycans on host cells to initiate infection.^4,5^

Despite mammalian glycans being composed of only ten different monosaccharides, they exhibit remarkable structural diversity that arises from the modular assembly of a limited number of structural elements into highly complex structures.^6^ Glycans are biosynthesized by glycosyltransferases that transfer a monosaccharide from an activated sugar-nucleotide to a specific hydroxyl of a growing oligosaccharide chain.^7^ The resulting product can then act as an acceptor for another glycosyltransferase. Glycan structures expressed by a cell reflect multiple factors including cellular metabolism, developmental stage, nutrient availability, and other cues from the cellular environment. It is precisely this diversity and plasticity that are critical for many biological functions of glycans.

Diverse collections of well-defined glycans are needed to investigate the molecular mechanisms by which these biomolecules mediate biological and disease processes. They are needed as ligands to study interactions with glycan-binding proteins,^8,9^ as standards for glycan structure determination of heterogeneous biological samples,^10,11^ and as probes to examine the molecular basis of glycoconjugate biosynthesis and as starting materials for glycoprotein synthesis.^12^ The enormous structural variability of glycans has stimulated the development of automated methods to prepare this class of compounds.^13–16^ Solid-supported synthesis has been the paradigm to automate the preparation of compounds, such as peptides and oligonucleotides. It has also been employed to streamline the chemical synthesis of glycans. For example, a commercial glycan synthesizer (Glyconeer 2.1),^17^ which uses linker-functionalized polystyrene and a set of standardized glycosyl donors, has been employed to prepare sulfated polysaccharides derived from algae.^18^ Furthermore, a standard HPLC has been reconfigured for solution-based reagent delivery for polymer-supported automated oligosaccharide assembly. In this step up, the reaction progress is monitored in real time using a UV detector. Another automation approach is based on solution-phase chemistry using CF_3_(CF_2_)_*n*_-tagged saccharides that can be captured on fluorous silica gel. Product release is accomplished by washing with an organic solvent such as methanol or acetonitrile, which after coevaporation with toluene gives a compound ready for the next reaction.^19^ The attraction of this approach is that chemical glycosylations are generally more efficient in solution than on the polymeric support. Automated solution-phase chemical oligosaccharide synthesis has also been accomplished by one-pot iterative additions of preactivated glycosyl donors, making it possible to perform as many as ten-component tandem reactions to provide arabinans up to a 1,080-mer.^20^ Despite these advances, a hurdle for the automated chemical synthesis of complex carbohydrates is a lack of general chemical glycosylation protocols. In particular, the installation of 1,2-*cis*-glycosidic linkages often gives a mixture of anomers that may be difficult to resolve after multiple glycosylation cycles.^21,22^

Several shortcomings of chemical oligosaccharide assembly can be addressed by using glycosyl transferases.^12^ Glycosylations by these enzymes make it possible to prepare a wide range of eukaryotic oligosaccharides including *N*- and *O*-glycans, glycosphingolipids, and glycosylaminoglycans.^23–26^ Many microbial and mammalian derived glycosyltransferases have been identified that can be expressed in *E. coli* or in mammalian cell culture. These enzymes are highly regio- and stereoselective, allowing the installation of a wide range of glycosidic linkages.^27,28^ Sugar nucleotide donors employed for eukaryotic glycan biosynthesis are commercially available and can be prepared by chemical-^29^ or enzyme-based methods. It is also possible to generate sugar nucleotides *in situ* by coupled enzymatic transformations.^30^

We have introduced *stop-and-go* strategies to prepare multiantennary *N*-glycans having unique appendages at each branching point (Scheme 1).^31–34^ The premise of this methodology is the use of unnatural sugar nucleotide donors, such as 5′-diphosphate-2-deoxy-2-trifluoro-*N*-acetamido-glucose (UDP-GlcNHTFA). The trifluoroacetamido (TFA) moiety of the resulting glycan can be hydrolyzed by a mild base to give GlcNH_2_ that can be converted into various derivatives such as an azide (GlcN_3_) or protected as *tert*-butoxycarbonyl (Boc) (GlcNHBoc). The modified GlcN moieties block modifications by various glycosyl transferases, thereby allowing the selective extension of a natural GlcNAc branching point. At an appropriate point in the synthesis, a GlcNH_2_, GlcN_3_, or GlcNHBoc moiety can chemically be converted into natural GlcNAc, which can then enzymatically be elaborated into various epitopes. The use of an unnatural sugar nucleotide donor has also been employed to install in regioselective fucosides,^35–39^ I-branching,^40–42^ and sulfates.^43–45^

**Scheme 1:**
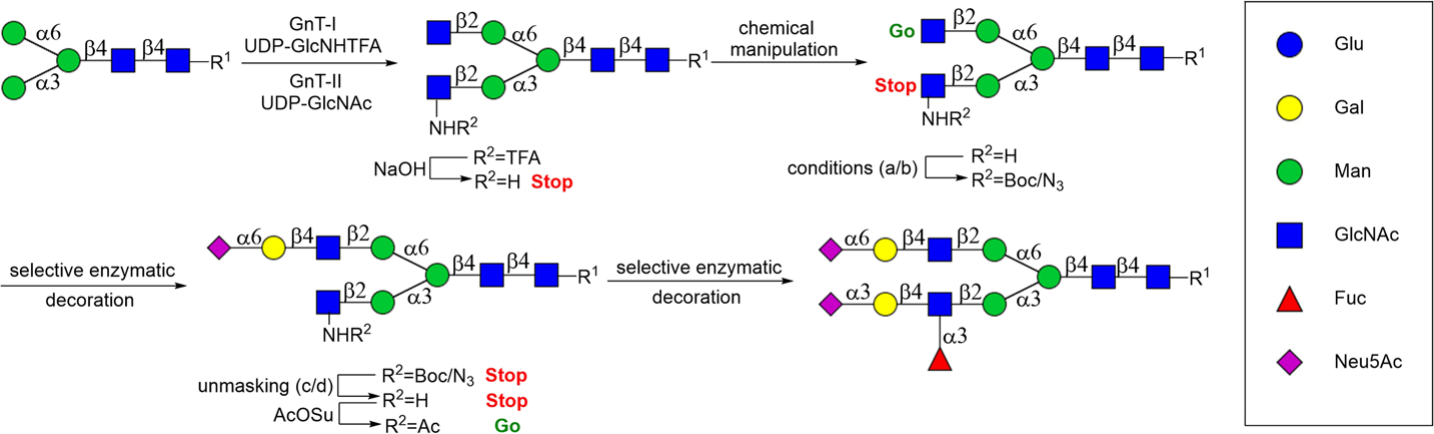
Stop-and-Go Chemoenzymatic Synthesis of Branched Glycans^a^

a Condition a: di-^t^butyl dicarbonate, NaHCO_3_; Condition b: Imidazole-1-sulfonyl azide, CuSO_4_, unmasking; Condition c: Trifluoroacetic acid, unmasking condition; d: PMe_3_, NaOH.

Several approaches have been introduced to streamline and automate the enzymatic synthesis of complex glycans.^16,46,47^ Glycosyl transferase catalyzed reactions are generally slow when an acceptor is immobilized to a resin, and as a result, such an approach is not amenable to automation.^48,49^ A more promising approach is the use of microfluidic reactors in which glycosyl transferases are immobilized for cascade reactions with acceptors in solution.^13,50–52^ This approach has, however, only been applied to a limited number of glycosyltransferases, possibly due to their fragility. One-pot multienzyme strategies are another attractive means to speed up enzymatic glycan synthesis. In this methodology, enzymes for sugar nucleotide synthesis and glycosyl transferases are employed in the same reaction vessel to provide access to glycans such as human milk oligosaccharides and glycosphingolipids.^53^ This strategy can, however, not easily switch between buffer systems, which can cause difficulties because different glycosyl transferases require different buffer conditions for optimal performance.

Fully automated platforms for glycosyl transferase-catalyzed reactions have also been reported. One approach is based on a reconfigured peptide synthesizer using a temperature-responsive polymer that is soluble at low temperatures (25 °C) and insoluble at higher temperatures.^47^ It combines the benefits of homogeneous enzyme catalysis and the recovery of polymer-bound products. The thermoresponsive property of the polymer is, however, compromised when modified by large glycans limiting the types of glycans that can readily be prepared by this methodology. We introduced an automation platform based on a catch and release approach in which glycosyltransferase-catalyzed reactions are performed in solution and product purification is achieved by solid phase extraction (SPE).^54^ The challenge was to identify a tag for SPE that allows the capture and release of a wide variety of oligosaccharide acceptors with high efficiency using a single set of conditions in a small elution volume. Such stringent requirements cannot easily be met by for example ionic-liquid-based^55^ or light fluorous tags.^56^ We addressed this challenge by introducing a sulfonate-based tagging approach. Such a tag can be introduced by oxime ligation of a reducing sugar and compound **1** (Scheme 2a).^54^ The resulting tagged compounds (e.g., **2**) can be retrieved on a diethylaminoethyl (DEAE) ion-exchange resin, and washing with a low concentration of ammonium bicarbonate (NH_4_HCO_3_) removes all reaction components including sugar nucleotides. Product release can be accomplished by washing with a high concentration of aqueous NH_4_HCO_3_ (0.3 M). Simply adjusting the pH of the eluent with acetic acid gives a buffer appropriate for the next enzymatic transformation, giving access to various classes of complex glycans.

**Scheme 2:**
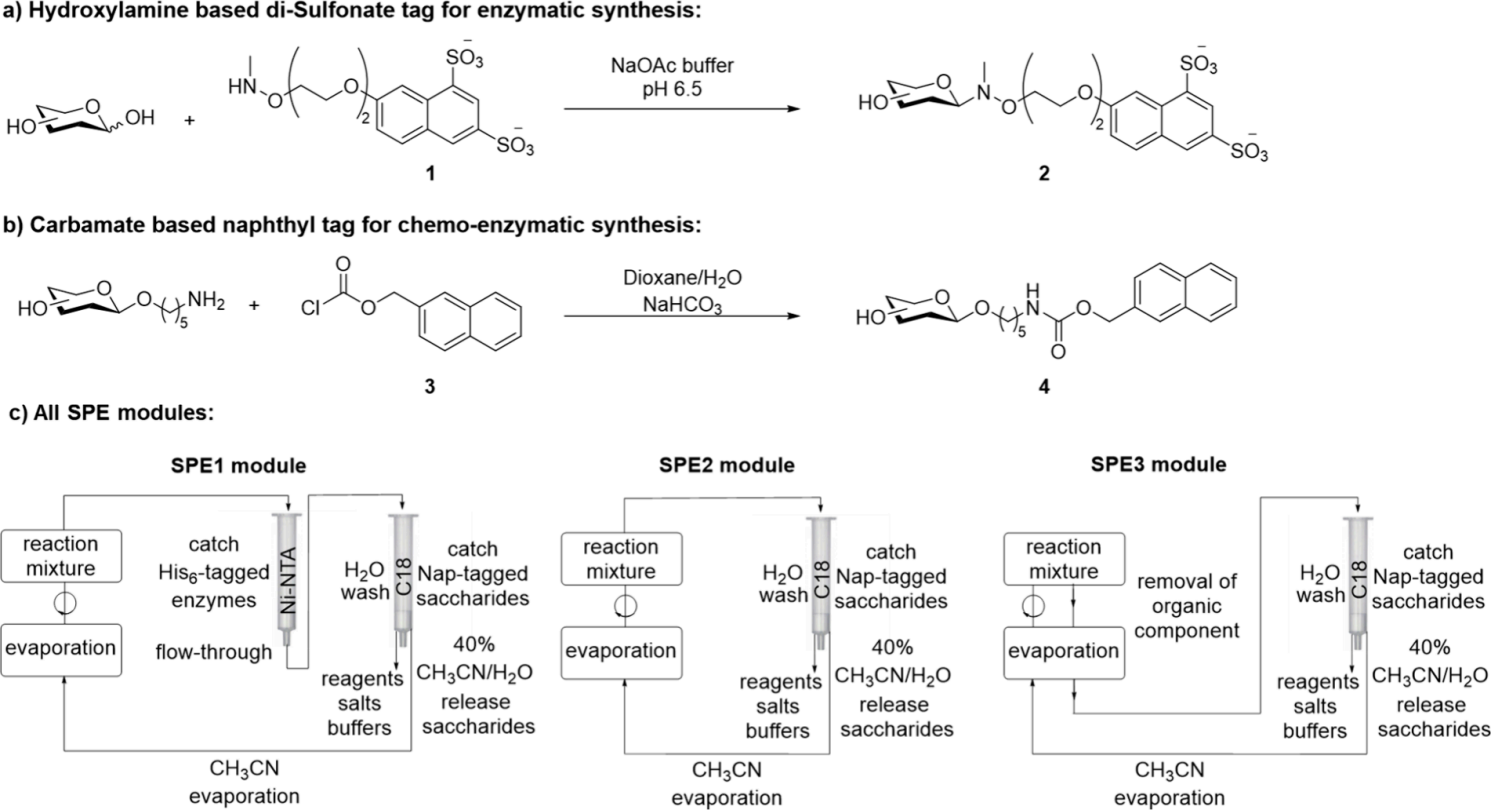
(a) Sulfonate Catch and Release Tag for Automated Enzymatic Synthesis of Complex Glycan; (b) Versatile 2-Naphthylmethyl Tag for the Chemoenzymatic Synthesis of Complex Glycans; (c) All SPE Modules

Despite the many attractive features of linker **1**, it has several shortcomings that limit the scope of the types of glycans that can readily be synthesized in an automated fashion. Most notably, linker **1** is not compatible with chemical manipulations required for *stop-and-go* glycosylation protocols, and as a result, asymmetrically branched glycans cannot be prepared by the catch and release approach. For example, compound release by 0.3 M aqueous NH_4_HCO_3_ is not compatible with base-sensitive functional groups and will result in the hydrolysis of GlcNHTFA, critical for *stop-and-go* glycosylation strategies. Furthermore, a large excess of linker **1** is required to drive the reaction to completion. Also, the sulfonate linker paradigm cannot be extended to the synthesis of highly charged oligosaccharides that contain, for example, a sulfate or multiple sialic acids because of too strong a binding to the DEAE ion-exchange resin. Finally, *in situ-generated* ammonium acetate buffer is suboptimal for several enzymatic transformations.

To address these limitations, we describe here an automation platform that integrates enzymatic glycan assembly with chemical modifications, making it possible to prepare asymmetrically branched glycans that can be modified by sialic acids and sulfates. The methodology is based on an anomeric 2-methylnaphthalene (Nap) tag (Scheme 2b), which facilitates product purification utilizing three optimized capture and release modules. In each module (Scheme 2c), the product is captured on a C18 resin, and product release is accomplished by a single elution step using 40% aqueous acetonitrile to give a product in a small volume. A custom 3D-printed device allows controlled evaporation of volatile organic solvents, leaving the product in water ready for a subsequent reaction cycle. Reactions and purification cycles can be easily programmed through the Autosuite software of the ISYNTH AI SWING workstation (ChemSpeed Technologies), and all liquid-handling is executed without manual intervention. In the case of enzymatic transformations, the C18 solid phase extraction is preceded by passing the reaction mixture over a Ni-NTA resin to remove His_6_-tagged enzymes (SPE1), whereas for chemical transformation in aqueous solutions, this step is omitted (SPE2) (Scheme 2c). For chemical reactions performed in an organic solvent, reaction mixtures are first subjected to evaporation, followed by C18 solid phase extraction (SPE3) (Scheme 2c). The newly developed platform made it possible, for the first time, to prepare in an automated fashion sulfated polylactosamines and asymmetric multiantennary complex *N*-glycans via sequential enzymatic and chemical reaction cycles. Removal of the Nap tag can be performed by hydrogenation to give oligosaccharides ready for microarray printing or bioconjugation.

## Results and Discussion

### A Catch and Release Approach Using a 2-Methylnaphthalene (Nap) Tag

We anticipated that an anomeric Nap tag would offer sufficient hydrophobicity to capture a wide range of oligosaccharides of different complexities, including charged derivatives, on a reverse-phase C18 resin. Subsequent washing with water would remove all reaction components, including buffer salts and excess sugar nucleotide. The Nap-tagged compound can then be released by a further washing step using an acetonitrile/water mixture. It was expected that the catch and release approach would be compatible with chemical manipulations required for *stop-and-go* chemoenzymatic synthesis protocols. Only neutral catch and release conditions are employed that were expected to be compatible with GlcNHTFA moieties. The robust UV absorbance of the Nap group facilitates facile detection during HPLC purification of the final targets. To prepare versatile Nap-tagged starting materials, the aminopentyloxy linker of lactose and *N*-acetyl lactosamine (LacNAc) derivatives **5** and **8**, as well as the α-amine of the asparagine moiety of *N*-glycan **11**, were reacted with 2-naphthylmethyl chloroformate to yield the corresponding 2-methylnaphthalene-tagged compounds **6**, **9**, and **12**, respectively. Aqueous solutions of the Nap-tagged compounds (5 mg) were applied to a C18 resin bed (0.4 mL), which was washed with 4 mL of water (10 column volumes). Residual reagents, salts, and buffer components employed for enzymatic transformations are not retained by the reverse phase resin and elute in the void volume. Various conditions were examined for compound release, and it was found that elution with 3 mL of 40% aqueous acetonitrile resulted in the quantitative release of all products (see Supporting Information Section 5c). Acetonitrile could easily be removed by subjecting the mixture to an airflow with shaking and heating to 37 °C for 45 min to give an aqueous solution ready for the next transformations. The volume of the mixture had been reduced by 45%, and thus, it was assumed that acetonitrile had been efficiently removed. It can, however, not be excluded that residual acetonitrile is present. If this is the case, it did not impact any of the subsequent enzymatic transformations. Thus, a single set of capture and release conditions can be employed for glycans of different complexities, which is critical for achieving a fully programmable workflow.

Next, we explored the compatibility of the catch-release approach with enzymatic transformations. Thus, HEPES buffer (1 M, pH 7.5) was added to aqueous solutions of compounds **6** and **9** to give a final buffer concentration of 100 mmol. Next, β1,3-*N*-acetylglucosaminyltransferase (B3GNT2), uridine 5′-diphosphate *N*-acetylglucosamine (UDP-GlcNAc), and MgCl_2_ (10 mM) were added, and the resulting reaction mixtures were incubated for 16 h at 37 °C to give compounds **7** and **10**, respectively. In parallel, Tris-buffer (1 M, pH 7.5) was added to Nap-tagged N-glycan **12** in water to give a buffer concentration of 100 mmol, which was followed by the addition of β1,4-galactosyltransferase 1 (B4GALT1), uridine 5′-diphosphogalactose (UDP-Gal), and MnCl_2_ (10 mM) to give **13** (Scheme 3). We implemented a dual purification strategy. All employed bacterial and human recombinant glycosyltransferases carry an *N*-terminal His_6_ tag, allowing efficient capture on the Ni-NTA resin. Thus, the reaction mixtures were first passed through a 0.1 mL Ni-NTA resin bed to retain the enzyme. The flow-through, which contains the Nap-tagged product along with the remaining reaction components, was applied to a C18 resin-packed column (0.4 mL) and washed with 4 mL of water to remove excess nucleotide donor, salts, and buffer components. The product was released using 3 mL of 40% aqueous acetonitrile and then subjected to a controlled airflow to remove acetonitrile (SPE1). LC-MS analysis of the eluants confirmed the presence of a single product peak, demonstrating the robustness of the solid phase extraction protocol.

**Scheme 3:**
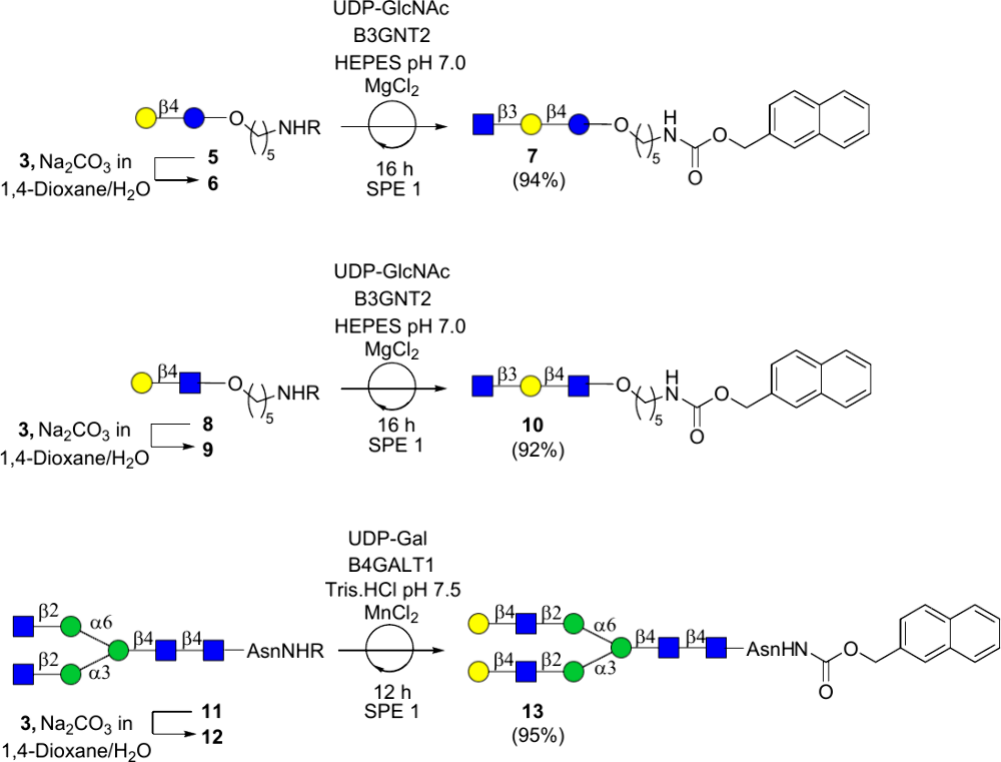
Installation of a Nap Tag at Various Versatile Precursors and Subsequent Enzymatic Transformation

Different tagging technologies can be used for recombinant protein expression and SPE-1 can be adapted accordingly with an appropriate resin to remove the protein from the reaction mixture. The tag is exploited in SPE1 for enzyme removal to prevent potential clogging during the C18 affinity purification step. Other tags, such as the Strep-tag, are also employed for recombinant enzyme expression and purification. If such a tag is used, SPE-1 can be adapted and, for example, Strep-Tactin can be used to remove Strep-tag containing enzymes.

### Automated Workflows on a Glycosynthesizer

Next, attention was focused on the automation of the catch and release workflow. An AI Swing Isynth (Chemspeed) was employed that can perform liquid handling in a fully automated manner by a robotic arm to move a 4-needle head that is controlled by high-resolution syringe pumps of different capacities (see Figure 1). The platform is equipped with a cooled rack (4 °C) for storage of sensitive reagents such as enzymes and sugar nucleotides, a temperature-controlled reactor block with shakingability, and a rack holding solid phase extraction cartridges. Also, racks are incorporated for bulk storage of buffers, reagents, and solvents and for holding samples for off-line LC-MS analysis. To adapt the robotic system for the removal of volatile organic components from aqueous mixtures, we integrated a custom-designed “*evaporation zone*” within the temperature-controlled reaction rack. An in-house 3D-printed clip, engineered to fit securely onto the neck of a 15 mL reaction tube, was fabricated with two nozzles: one connected to a regulator-controlled continuous airflow source and the other equipped with a needle that directs airflow precisely onto the solution surface (see Figure 1A,B). This setup enables efficient evaporation of organic solvents, such as acetonitrile and tetrahydrofuran, at a rate of approximately 2.5–4 mL/h when vortexed and heated at 37 °C.

**Figure 1:**
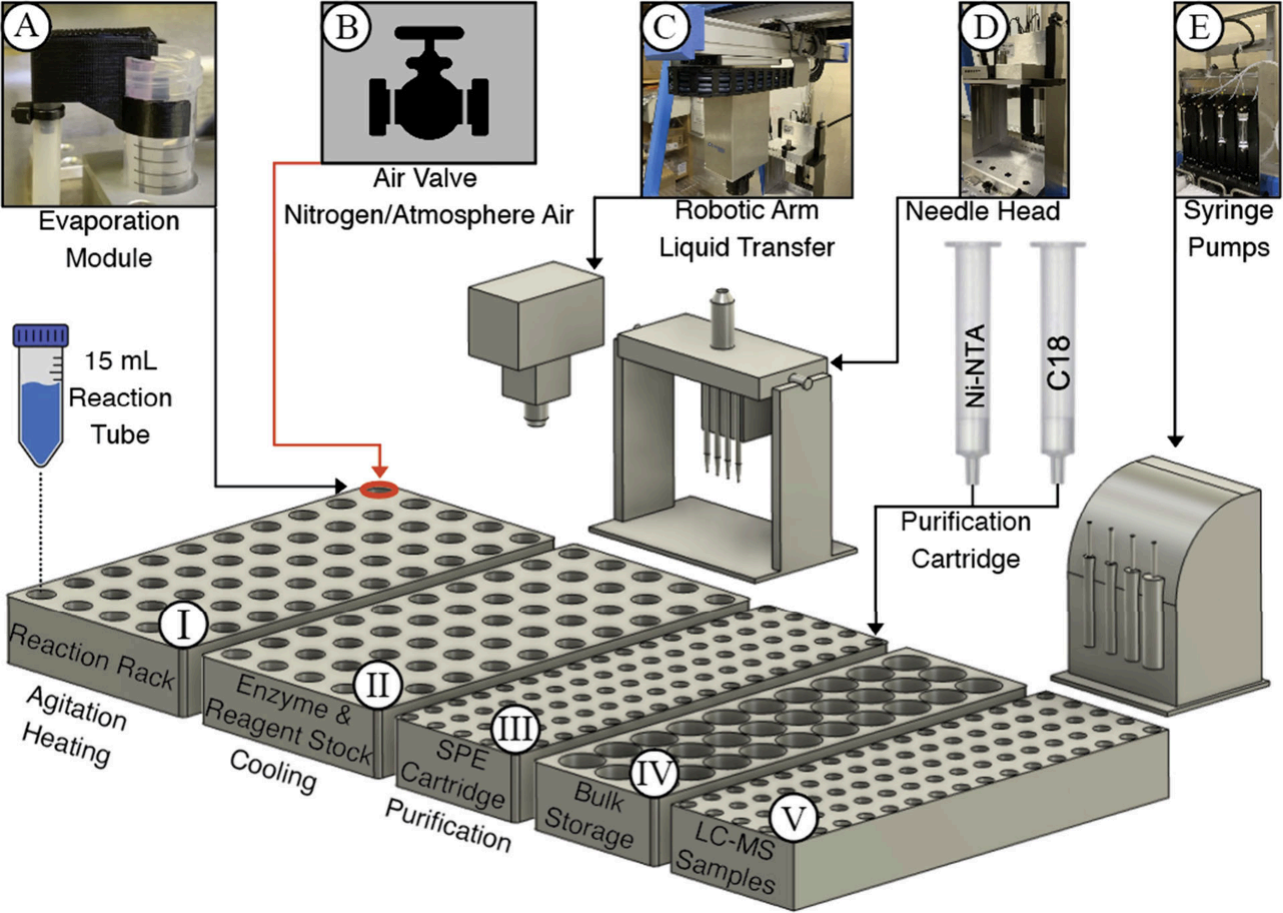
Schematic representation of the glycosynthesizer based on a Chemspeed AI Swing Isynth platform. (A) 3D-printed evaporation fitting; (B) Valve-controlled airflow; (C) Robotic arm for liquid transfer and dispensing; (D) Needle heads; (E) Syringe pumps of different capacities; (I) Reaction rack; (II) Enzyme/reagent stock rack; (III) SPE rack; (IV) Bulk storage for buffers, reagents, solvents; and (V) LC-MS sample holding rack.

In a typical fully automated workflow for enzymatic transformations, an aqueous solution of an oligosaccharide is placed in a 15 mL tube on the temperature-controlled reaction rack. Using the robotic arm-controlled needle head, an appropriate buffer, sugar nucleotide, enzyme, and metal salts are transferred from respective stock solutions kept at predefined positions in the bulk storage rack and gently mixed at 37 °C for a set period. Next, the robotic arm transfers the reaction mixture onto a pre-equilibrated Ni-NTA resin-packed cartridge with the SPE rack set in the “*collect*” position. The His_6_-tagged enzyme is retained on the column, while the flow-through, containing the Nap-tagged product, is collected and transferred to a C18 cartridge set in the “*waste*” position. Deionized water (4 mL) is passed through the C18 column to remove residual reaction components. The program then adjusted the SPE rack to the “*collect*” position, and the product is eluted using 3 mL of 40% aqueous acetonitrile. The collected eluant is transferred to the evaporation zone, where the acetonitrile is removed by vortexing and heating at 37 °C for 45 min under an airflow, yielding an aqueous solution of the product ready for the next reaction cycle.

The sequence of solid phase extraction by Ni-NTA resin, a C18 cartridge followed by evaporation, is termed SPE1 (Scheme 2c). Reactions and purification cycles are programmed through the Autosuite software of the ISYNTH AI SWING workstation (see Supporting Tables for detailed programs).

### Chemical Transformations on the Automation Platform

To construct asymmetric glycans by *stop-and go* strategies, automated workflows are needed to introduce a GlcNHTFA moiety for subsequent chemical conversions into GlcNH_2_, GlcN_3_, or GlcNHBoc that can act as stopping points (see Scheme 1). Also, protocols are needed to chemically unmask the unnatural monosaccharides to give GlcNAc. To achieve this goal, we assembled, in an automated fashion, oligo-LacNAc derivative **15** having a GlcNHTFA moiety, which was chemically converted into derivatives **16**–**18**. The latter compounds were then transformed into **19** having a natural terminal GlcNAc moiety.

Thus, compound **14** was enzymatically assembled starting from **6** by consecutive cycles of B3GNT2- and B4GALT1-catalyzed transformations, each followed by SPE1. Next, noncanonical GlcNHTFA was introduced to generate **15** using *Helicobacter pylori* β1,3-*N*-acetylglucosaminyl transferase^57^ in the presence of UDP-GlcNHTFA^58^ in Tris buffer (100 mM, pH 7.0), MgCl_2_ (10 mM), and DTT (1 mM) followed by SPE1. To install various stopping glucosamine derivatives, compound **15** was treated with aqueous NaOH (1 M) to yield **16**, bearing a terminal GlcNH_2_ moiety. As no enzyme removal was required, the reaction mixture was directly loaded onto a C18 cartridge, and residual NaOH was removed by washing with water (4 mL). The retained product was eluted with an acetonitrile–water mixture, followed by evaporation of the acetonitrile to give an aqueous sample ready for a subsequent reaction. This solid phase extraction protocol, using only a C18 cartridge followed by evaporation, is termed SPE2 (see Scheme 2c). The terminal glucosamine moiety of **16** was converted into 2-deoxy-2-azido-glucoside by the addition of imidazole-1-sulfonyl azide^59,60^ in water, and after a reaction time of 12 h, the reaction mixture was subjected to SPE2 to yield **17**. In parallel, compound **16** was converted into Boc-protected derivative **18** by treatment with di-*tert*-butyl dicarbonate in a mixture of water and 1,4-dioxane for 12 h. A separate solid phase extraction protocol (SPE3) was developed to remove the organic solvent to prevent premature elution of the product from the C18 resin. Thus, the reaction mixture was transferred to the evaporation zone to remove volatile organic components by subjecting it to an airflow under vortexing and heating to 45 °C. The resulting aqueous solution was transferred to a C18 cartridge, which was washed with water and then aqueous acetonitrile, and the resulting eluant was subjected to evaporation to give an aqueous solution of **18** ready for the next transformation. The azide of **17** could be reduced to an amine by a Staudinger reaction using PMe_3_ in THF, and the resulting product **16** was retrieved using SPE3. In this respect, PMe_3_ has a low boiling point of 38 °C and can be easily removed by the evaporation step of SPE3. The GlcNH_2_ moiety of **16** was acetylated using an aqueous solution of *N*-acetyl-*N*-hydroxysuccinimide ester (AcOSu), and the resulting compound **19** was isolated using SPE2. Removal of Boc of **11** could be achieved by treatment of 2% aqueous trifluoroacetic acid (TFA) for 4 h to give an aqueous solution of compound **9** after retrieval by SPE2 (Scheme 4).

**Scheme 4:**
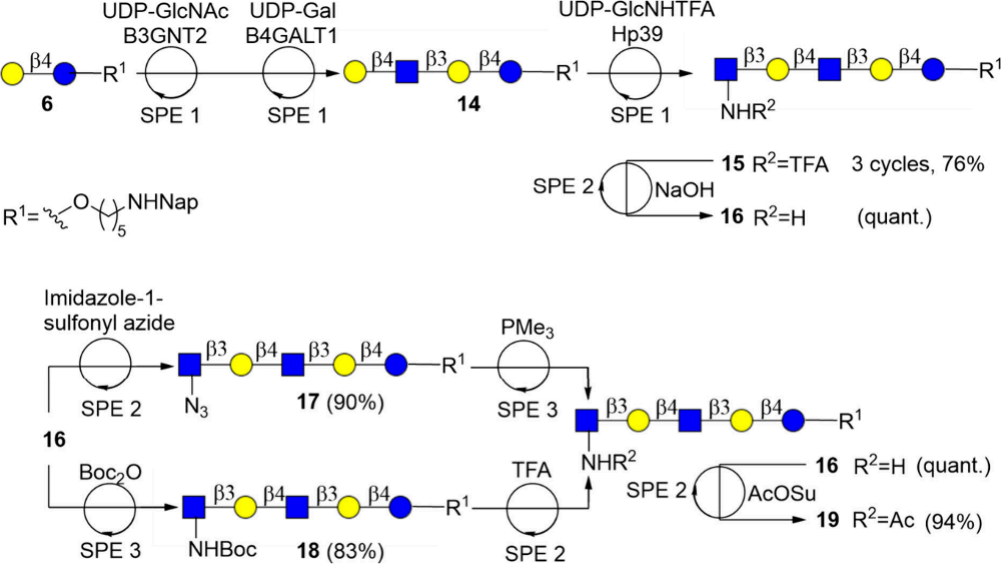
Automated Chemical Transformations

LC-MS analysis of the product of each transformation confirmed the efficiency of the chemical transformations executed within the fully integrated automated workflow (Figure 2). NMR analysis confirmed the purity and structural integrity of compounds **15**–**19**.

**Figure 2:**
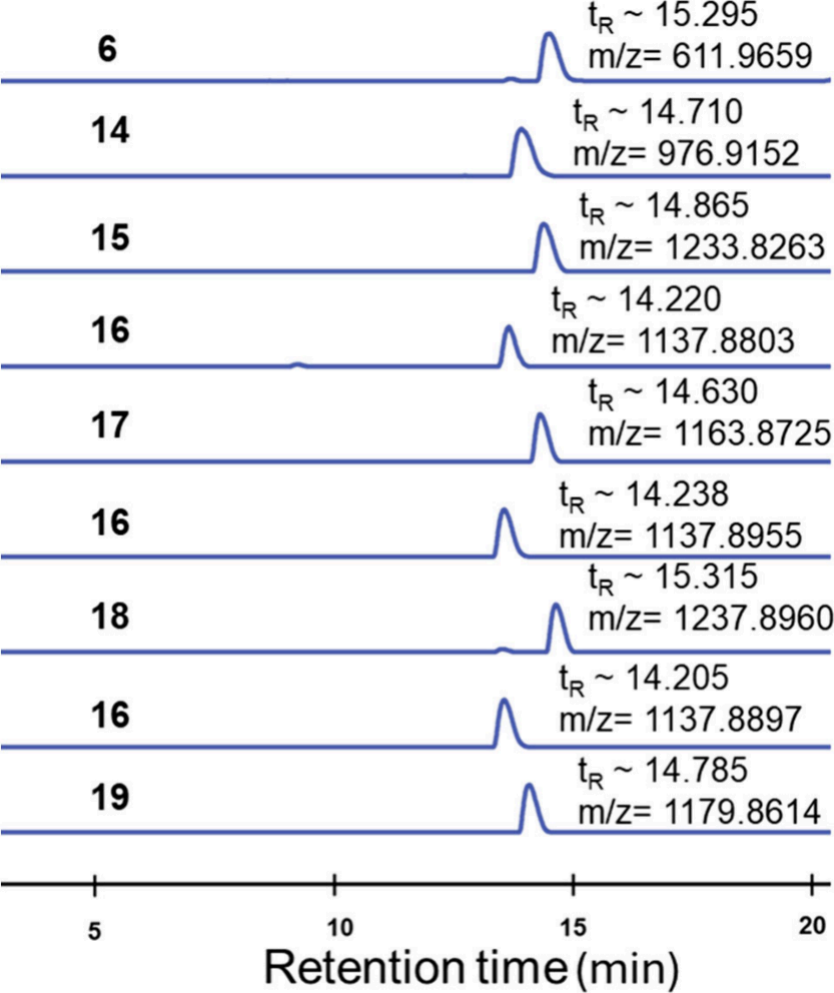
LC-MS traces after completion of each reaction and SPE in Scheme 4.

### Automated Synthesis of Sulfated Glycans

Sulfation is a common modification of complex glycans that has been implicated in many biological processes.^61^ For example, 6-*O*-sulfo-sialyl Lewis^*x*^ (Neu5Acα­(2,3)­Galβ­(1,4)­[Fucα­(1,3)]­GlcNAc6S) is a ligand for l-selectin, which is important for lymphocyte extravasation and inflammatory response.^62,63^ It is also a preferred epitope for immunoglobulin-like lectin (Siglec) 9,^64^ which is an immune-inhibitor receptor implicated in several cancers. On the other hand, Siglec 8, which is selectively expressed on human mast cells and eosinophils and can participate in inflammatory responses,^65^ preferentially binds Neu5Acα­(2,3)­6S-Galβ­(1,4)­GlcNAc.^66^

The C-6 hydroxyl of a terminal GlcNAc moiety can be sulfated by GlcNAc-6-*O*-sulfotransferases 2 and 6 (CHST2 and 6) to give GlcNAc6S^67^ that can be galactosylated by β1,4-galactosyltransferase 4 (B4GALT4). Furthermore, C-6 hydroxyls of internal galactosides of LacNAc moieties can be sulfated by keratan sulfate galactose 6-sulfotransferase (KSGal6ST or CHST1) or chondroitin sulfotransferase-1 (C6ST1).^67^ It has been found that CHST1 prefers galactosides having a neighboring GlcNAc6S residue, and its activity can further be modulated by a 2,3-linked sialoside.^43^ Furthermore, it is known that α1,3-fucosides block the action of CHST1, whereas a C-6 sulfate on galactoside (Gal6S) prevents fucosylation by FUT6.^44^

We explored the potential of the Nap tag for the automated synthesis of sulfated glycans, which was not possible with the previously developed sulfonate affinity tag.^54^ The focus was on the preparation of sulfated glycans **23** and **26** from common intermediate **21**. We exploited that CHST2 only sulfates terminal GlcNAc moieties, and furthermore, the preparation of **19** relied on the mutual exclusivity of α1,3-fucosides and Gal6S moieties. The synthesis of key intermediate **14** began with the sequential enzymatic extension of LacNAc **9**, bearing a Nap-tagged aminopentyloxy linker, by B3GNT2 and B4GALT1 in combination with SPE1 to yield pentasaccharide **20**. Regioselective sulfation of the C-6 hydroxyl of the terminal GlcNAc residue of **20** was achieved by recombinant CHST2 and 3′-phosphoadenosine-5′-phosphosulfate (PAPS) in the presence of MnCl_2_ to furnish **21**. Notably, compound **21** could easily be isolated by the SPE1 module (Scheme 2c), demonstrating the compatibility of the methodology with charged glycans.

The terminal 6-sulfo-GlcNAc moiety of **21** was galactosylated by B4GALT4 and UDP-Gal to generate hexasaccharide **22**, which was capped with an α2,3-linked sialoside using recombinant ST3GAL4 and CMP-Neu5Ac, affording glycan **23**.

Compound **21** could selectively be fucosylated by FUT6 to install two Lewis^*x*^ moieties, as FUT6 modifies only LacNAc moieties and not a terminal GlcNS moiety. Galactosylation of the resulting compound using B4GALT4 and UDP-Gal furnished **24**, which was 2,3-sialylated by ST3GAL4 and CMP-Neu5Ac to afford **25**. The galactoside, flanked by the sialoside and 6-sulfated GlcNAc residue, was selectively sulfated using CHST1 and PAPS, yielding disulfated compound **26** (Scheme 5). In the latter transformation, we exploited the fact that Le^*x*^ moieties block sulfation by CHST1. The SPE1 module was employed to purify each intermediate and final product. After each transformation, a small sample was collected by using the needle head and transferred to the sample holding rack (see Figure 1). Offline analysis by LC-MS revealed the complete conversion of the starting materials into the expected products (see Figure S7). The final products were subjected to size exclusion column chromatography over extra fine P4 biogel to give **23** and **26** in an average yield of 88% per transformation. The compounds were characterized by two-dimensional homo- and heteronuclear NMR, confirming the structural integrity of the compounds. For example, structural assignments of GlcNAc-6-sulfation, Gal-6-sulfation, α2,3-sialylation, and fucosylation for compounds **16** and **19** were validated through NMR experiments, including ^1^H, COSY, NOESY, TOCSY, and HSQC. The ^1^D ^1^H NMR and 2D ^13^C–^1^H HSQC spectra of **16** and **19** are depicted in Figure 3a,b. The 1D ^1^H NMR spectrum of **16** shows six anomeric signals, correlating with residues A, B, C, D, E, and F. The H-1 signal at δ H 4.42 ppm stems from a reducing-end GlcNAc residue (A), and a carbon signal was observed at δ C 100.94 ppm, while the anomeric protons of the internal GlcNAc units (C and E) appear at 4.70 and δ 4.72 ppm, respectively. The three nonreducing βGal residues were readily distinguishable with anomeric protons at δ 4.43 (B), 4.47 (D), and 4.59 (F) ppm. Notably, two diastereotopic 6-H protons on the GlcNAc unit E have a much higher downfield shift due to the presence of the 6-*O*-sulfate, making it easily detectable at δ 4.32 and 4.40 ppm. Furthermore, the presence of an α2,3-linked sialic acid on the terminal galactoside (F) induced an additional downfield shift of its H-3 proton, observed at 4.12 ppm, which is in line with the expected deshielding effect of the sialic acid. Similarly, the extended polyLacNAc **19** features two additional fucose units (H and I) with aromatic H1 peaks observed at δ 5.06 and 5.12 ppm (Figure 3b). The characteristic H5 protons of the fucoside were detected at δ 4.81 ppm. Additionally, a substantial downfield shift of the H6 protons of galactoside F supports regioselective 6-*O*-sulfation at the primary hydroxyl.

**Scheme 5:**
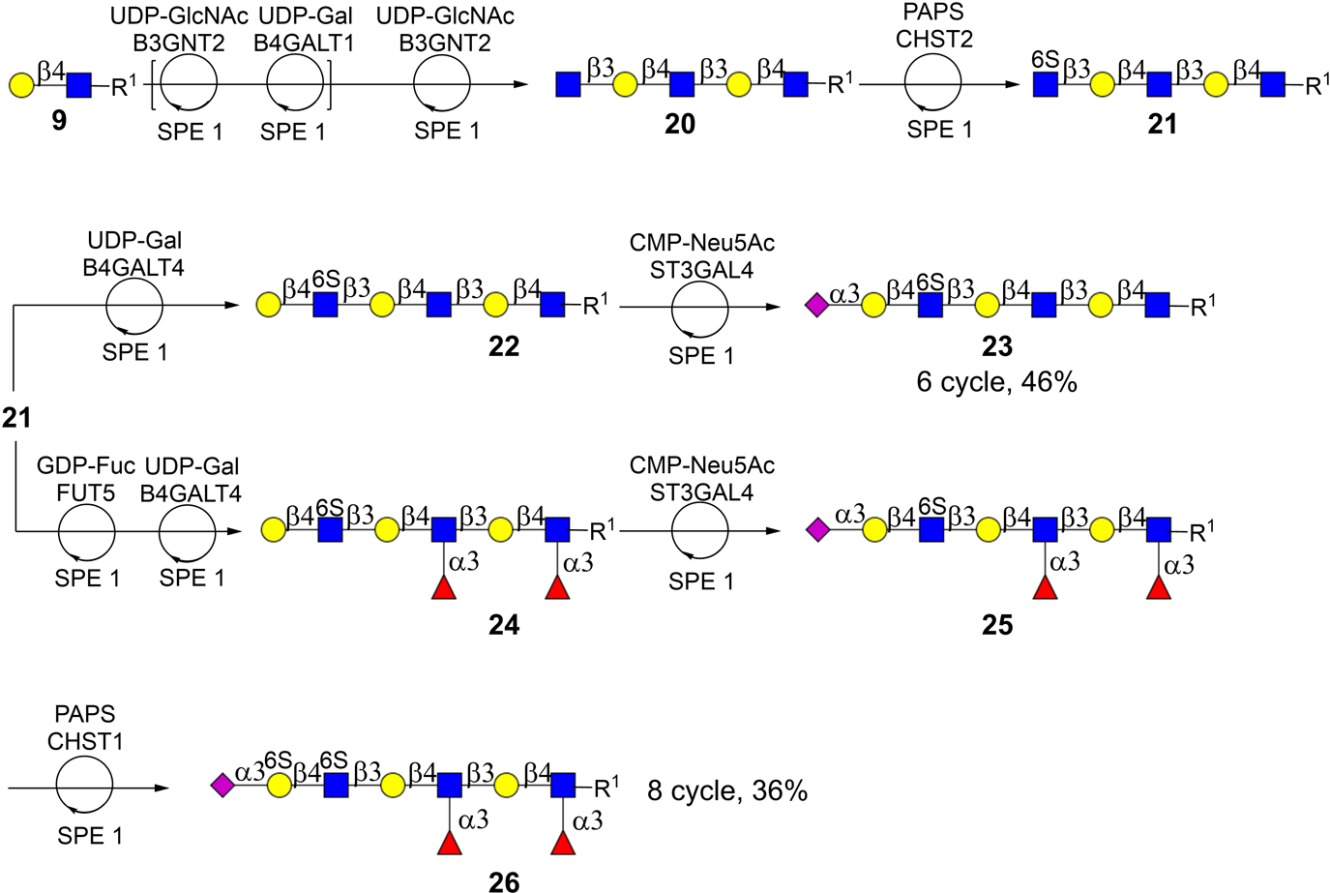
Automated Synthesis of Sulfated polyLacNAc Derivatives

**Figure 3:**
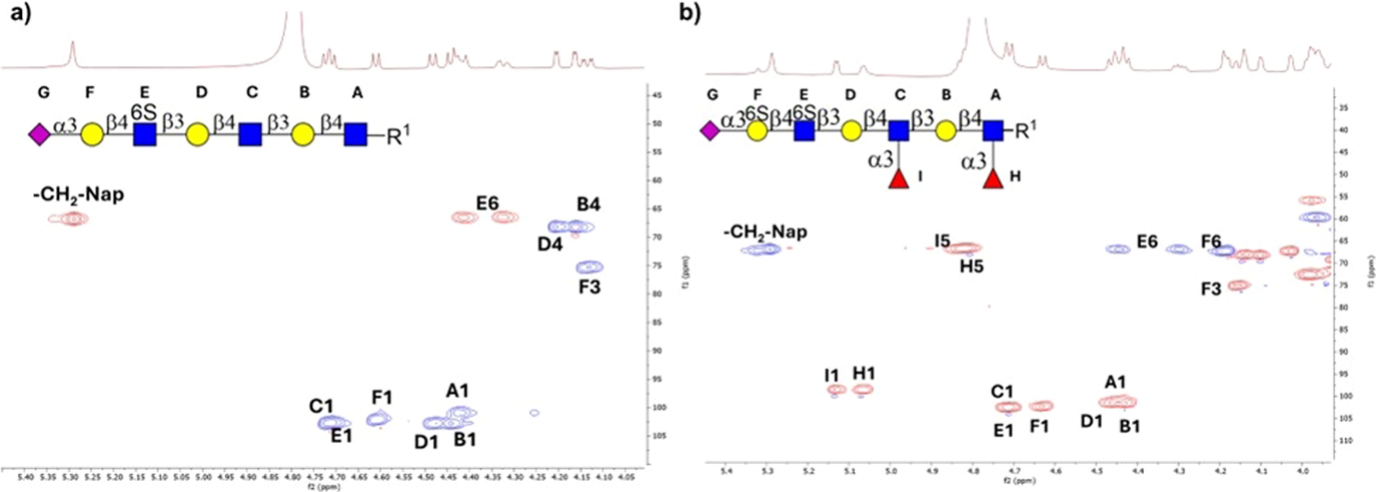
^1^H and ^1^H–^13^C HSQC NMR spectra of sulfated polyLacNAc (a) **23** and (b) **26** annotated with characteristic peaks.

### Automated Synthesis of Complex Asymmetric *N*-Glycans

The complexity of *N*-glycans arises from the modification of a common core pentasaccharide by a family of *N*-acetylglucosaminyltransferases to give oligosaccharides having various numbers and patterns of branching GlcNAc moieties.^1^ These terminal GlcNAc residues can be galactosylated by a galactosyltransferase to give *N*-acetyllactosamine (LacNAc), which in turn can be elaborated by a range of glycosyltransferases into various appendages, resulting in enormous structural diversity. Mature *N*-glycans usually have architectures in which each branching point is extended by a unique epitope to give asymmetrical glycans.

To demonstrate that the automation platform, based on the Nap-tag technology, can be extended to the synthesis of asymmetrical *N*-glycans, we first prepared bi-antennary *N*-glycan **34**, which has two different appendages at the α1,3- and α1,6-antenna (Scheme 6). Pauci mannoside **28** was used as the starting material, which can readily be prepared from sialylated glycopeptides (SGPs) isolated from egg yolk powder.^68^ In two steps, compound **28** was converted into bi-antennary *N*-glycan **30** employing UDP-GlcNHTFA for installation of a β1,2-linked GlcNHTFA at the α1,3-antenna followed by base treatment to cleave the TFA moiety. Next, we exploited that the resulting GlcNH_2_ is resistant to enzymatic modification, allowing for selective enzymatic assembly at the α6-antenna. At an appropriate stage of the synthesis, the GlcNH_2_ moiety can be acetylated to give natural GlcNAc (→**33**), allowing elaboration of the α3-antenna into a unique structure, thereby providing access to compound **34**.

**Scheme 6:**
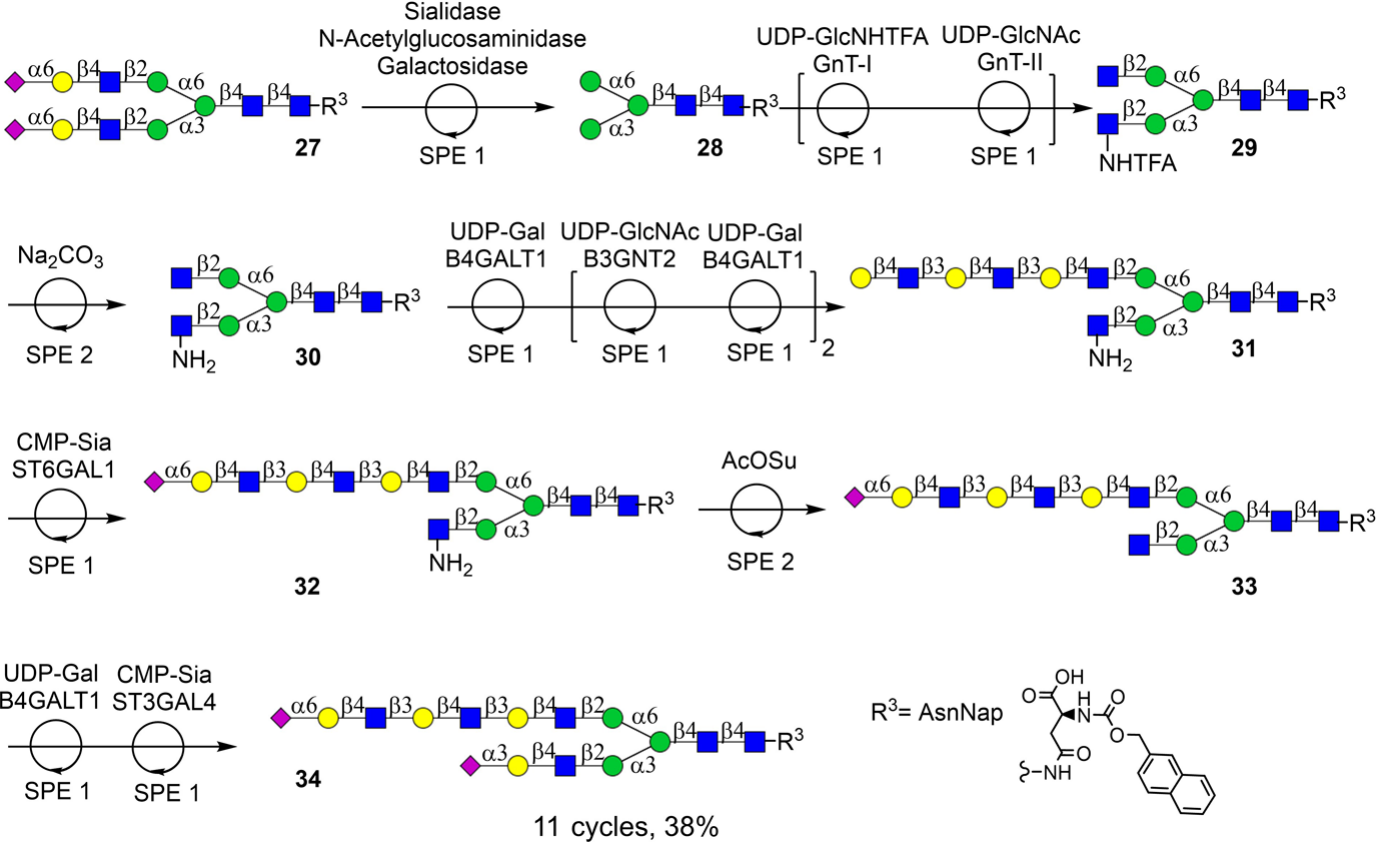
Automated Chemoenzymatic Synthesis of an Asymmetrical Bi-Antennary Glycan Using a Stop-and-Go Strategy and SPE1 and SPE2 Modules

Thus, SGP isolated from egg yolk powder^68^ was subjected to enzymatic trimming of its hexapeptide to give a compound having a single asparagine moiety that was derivatized with a Nap tag using 2-naphthylmethyl chloroformate to give, after purification by HPLC over a HILIC column, homogeneous **27**. Treatment of the latter compound with a cocktail of three glycosidases in one-pot in the presence of sodium acetate buffer (pH 5.0) resulted in the formation of Pauci-mannoside **28**. In an automated fashion, the Man α­(1,3) arm of intermediate **28** was modified with GlcNHTFA using GnT-I and UDP-GlcNHTFA. Importantly, the neutral elution conditions of SPE1 preserved the base-labile NHTFA functionality of the resulting compound. This is an important advance over the previous workflow utilizing sulfonate-based linker **1** (Scheme 1b) that requires basic ammonium bicarbonate for compound release,^54^ which is incompatible with base-sensitive functional groups such as NHTFA. Next, the Man α­(1,6) arm of **28** was modified with GlcNAc by employing GnT-II and UDP-GlcNAc to afford heptasaccharide **29**, which was subjected to aqueous Na_2_CO_3_ followed by SPE2 to provide **30** having a GlcNH_2_ moiety at α1,3-antennae. Next, the GlcNAc residue at the α1,6-antennae was selectively extended by three consecutive cycles of B3GNT2- and B4GALT1-mediated transformations, yielding **31**, which was capped with an α2,6-linked sialic acid employing ST6GAL1 to furnish compound **32**. Next, the GlcNH_2_ residue of **32** was acetylated using AcOSu followed by SPE2 to give **33**. The latter compound was subjected to consecutive modifications by B4GALT1 and ST3GAL4 to give, after purification by size exclusion column chromatography over P4, targeted asymmetric *N*-glycan **34** in 38% overall yield. The synthesis was completed in 11 automated reaction cycles, incorporating both the SPE1 and SPE2 modules, with an average yield of 93% per transformation cycle. NMR and LC-MS analyses of *N*-glycan **34** confirmed homogeneity and compound integrity. The sequence of reactions demonstrates that highly complex glycans can be prepared in an automated fashion by standardized enzymatic protocols and SPE modules.

Next, attention was focused on the synthesis of bi-antennary *N*-glycan **44** that has different epitopes at the α1,3- and α1,6-antenna (Scheme 7). Furthermore, its tri-LacNAc chain at the α3-antenna is selectively fucosylated and capped by an α2,6-linked sialoside. We adopted a chemoenzymatic strategy in which GlcNH_2_ and GlcN_3_ function as orthogonal stopping points. In particular, the GlcNH_2_ moiety of **40** is resistant to fucosylation whereas the GlcN_3_ residue masks the α6-antenna. In a selective manner, these noncanonical monosaccharides can be converted into natural GlcNAc allowing selective modifications.

**Scheme 7:**
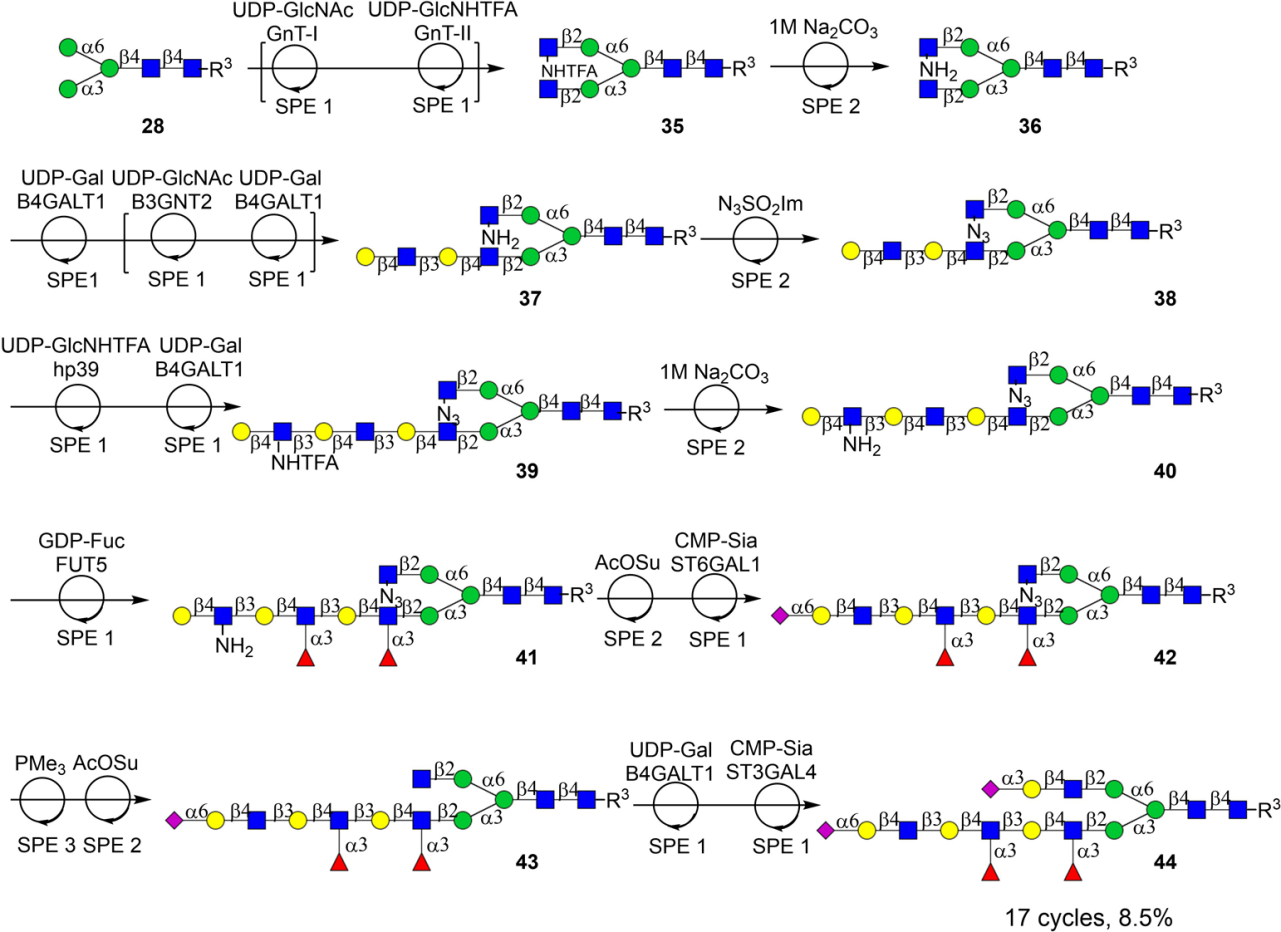
Automated Chemoenzymatic Synthesis of an Asymmetrical Bi-Antennary Glycan That Is Selectively Fucosylated at a tri-LacNAc Chain Using the SPE1, SPE2, and SPE3 Modules

Thus, Nap-tagged **28** was subjected to GnT-I and UDP-GlcNAc followed by GnT-II and UDP-GlcNHTFA to yield compound **35**. Base treatment of the latter derivative resulted in the formation of **36**, having a GlcNH_2_ moiety that temporarily is blocked from enzymatic modifications. Therefore, the GlcNAcβ­(1,2)­Manα­(1,3) arm of compound **36** could be extended by two LacNAc units by iterative enzymatic cycles of B3GNT2 and B4GALT1, producing compound **37**. To introduce an orthogonal enzymatic stopping point, the GlcNH_2_ moiety of **37** was converted into GlcN_3_ using imidazole-1-sulfonyl azide, followed by SPE2 to yield compound **38**. Next, a β1,3-linked GlcNHTFA residue was selectively installed on the extended α1,3-antenna using *Helicobacter pylori* β1,3-*N*-acetylglucosaminyltransferase^57^ and UDP-GlcNHTFA at 30 °C. Subsequent galactosylation by B4GalT1 afforded compound **39**, which contains two internal LacNAc units and one terminal LacNHTFA residue. To allow selective fucosylation of the internal LacNAc residues, compound **39** was treated with aqueous Na_2_CO_3_ to convert GlcNHTFA into GlcNH_2_ (**40**), which is resistant to α1,3-fucosylation by FUT6. Indeed, the treatment of **40** with FUT6 and GDP-Fuc yielded compound **41** bearing two fucosides at the internal GlcNAc moieties. The GlcNH_2_ of **41** was acelyated followed by sialylated using ST6Gal1 afforded intermediate **42**. The GlcN_3_ residue on the GlcNAcβ­(1,2)­Manα­(1,6) arm was then reduced to an amine using PMe_3_ in THF followed by SPE3 to remove the organic solvent and volatile components. Subsequent acylation of the resulting GlcNH_2_ residue with AcOSu generated native GlcNAc (**43**) that was further elaborated by B4GALT1 and ST3GAL4 to provide, after purification over the P4 biogel, the asymmetrically branched *N*-glycan **44**. The synthesis encompassed 17 consecutive reaction cycles, entailing both enzymatic and chemical transformation utilizing the SPE1, SPE2, and SPE3 modules, achieving an average yield of ∼86% per step.

The final challenge we addressed was to prepare, in an automated fashion, a tri-antennary *N*-glycan having unique appendages at each antenna (**52**, Scheme 8). It was envisaged that intermediate **47**, having GlcNAc, GlcNH_2_, and GlcN_3_ residues as branching moieties, would serve as an appropriate starting material for this purpose. The branching of *N*-glycans is a highly orchestrated process whereby a terminal GlcNAc residue at the α1,3-antenna (GnT-I arm) serves as critical recognition motif for GnT-II to install a β1,3-linked GlcNAc moiety at the α1,6-antenna.^69^ The latter residue is in turn a recognition element for GnT-V, which installs a β1,6-linked GlcNAc moiety at the α1,6-antenna. The branching GnTs can utilize UDP-GlcNHTFA as a substrate, and the resulting GlcNHTFA-containing glycan can serve as an acceptor for a subsequent branching GnT. Based on these considerations, we first assembled intermediate **29**, which has GlcNHTFA at the GnT-I arm and GlcNAc at the GnT-II antenna. By a two-step procedure, the GlcNHTFA moiety of **29** was converted into GlcN_3_ to give compound **45**, which was expected to be a substrate for GnT-V providing access to critical intermediate **40**.

**Scheme 8:**
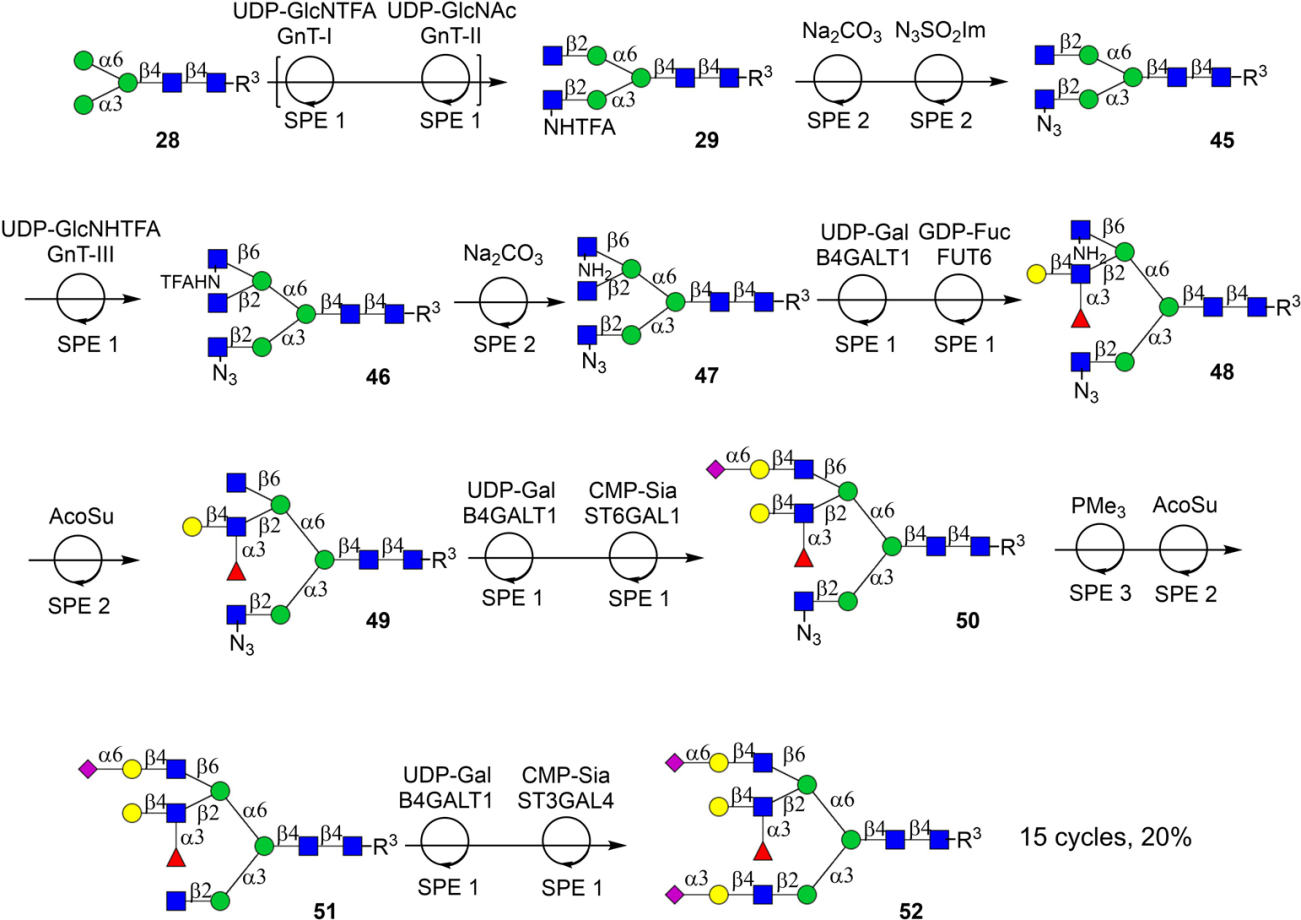
Automated Chemo-Enzymatic Assembly of a Tri-Antennary Glycan Having Different Appendages at Each Antenna Using Key Intermediate **46** with GlcNAc, GlcN_3_, and GlcNHTFA as Complementary Noncanonical Monosaccharides

To this end, intermediate **29** was prepared *via* the automated reaction cycles outlined in Scheme 5. Using aqueous Na_2_CO_3_, the NHTFA moiety of **29** was hydrolyzed to an amine, which was then converted into an azide by imidazole-1-sulfonyl azide to give compound **45**. Next, β1,6-linked GlcNHTFA was introduced using recombinant GnT-V and UDP-GlcNHTFA to produce tri-antennary glycan **46**. The reaction kinetics of this transformation are slow, and therefore, it was subjected twice to GnT-V and UDP-GlcNHTFA and SPE1 to drive the transformation to completion. Next, the GlcNHTFA of the resulting product was hydrolyzed under standard conditions to give critical intermediate **47**, which has GlcNH_2_ and GlcN_3_ as orthogonal stopping points. We exploited the fact that only the GlcNAc moiety at the GnT-II arm of **47** is amenable for enzymatic modification. Accordingly, galactosylation of **47** with B4GALT1 installed a LacNAc moiety at the GnT-II antenna, which was fucosylated by FUT6 yielding Lewis^*x*^ containing glycan **48**. It is well-known that Lewis^*x*^ is resistant to modification by many glycosyl transferases including sialyl transferases, thereby providing opportunities to selectively assemble appendages at the other antenna of **48**. Thus, the GlcNH_2_ residue at the GnT-V arm of **48** was acetylated to give natural GlcNAc (→**49**) enabling further enzymatic modifications. Galactosylation followed by sialylation with ST6Gal1 installed a 2,6-sialyl LacNAc moiety, furnishing compound **50**. Finally, the GlcN_3_ motif at the GnT-I antenna was reduced to an amine using PMe_3_, followed by purification by the SPE3, and subsequent acetylation of the resulting amine under standard conditions gave, after SPE2, compound **51** bearing a natural GlcNAc at the Manα­(1,3)-arm. This antenna was then extended by the sequential treatment with B4GALT1 and ST3GAL4 to give the targeted glycan after purification by size exclusion column chromatography over the P4 biogel to provide targeted compound **52**. The synthesis of this compound entailed 15 reaction cycles, comprising both enzymatic and chemical transformations, with an average yield of 89% per step. After each reaction, a sample was taken that was analyzed by LC-MS, which demonstrated efficient conversion of the starting materials into the expected products. The final product **52** was characterized by homo- and heteronuclear NMR. A combination of high-resolution 1D ^1^H NMR and 2D ^13^C–^1^H HSQC spectra revealed the anomeric proton of the asparagine moiety attached to the reducing end GlcNAc unit at δ 4.92 ppm, whereas the nonreducing end GlcNAc residues of the chitobiose core appeared at δ 4.58 ppm. The anomeric proton of the β­(1,4)-linked mannoside to the chitobiose core resides at δ 4.77 ppm, and the other two mannosides of the paucimannose core were detected at δ 5.12 ppm for Manα­(1,3) and δ 4.88 ppm for Manα­(1,6), respectively. GnT-I and GnT-II GlcNAc have anomeric protons at δ 4.59 ppm and β­(1,6)-linked GnT-V GlcNAc at 4.58 ppm. Anomeric protons of the three galactosides reside in the δ range of 4.55–4.45 ppm.

The anomeric proton of the α­(1,3)-linked fucoside of the Le^*x*^ epitope appeared at δ 5.14 ppm (see Figure S12). Characteristic H3 protons of the α­(2,6)-linked Neu5Ac unit were detected at δ 2.68 – H_eq_ and δ 1.72 – H_ax_, whereas for α­(2,3)-linked Neu5Ac, the peaks appeared at δ 2.77 – H_eq_ and δ 1.81 – H_ax_.

## Conclusions

Large collections of glycans are needed to investigate the molecular mechanisms by which these biomolecules mediate biological and disease processes, as ligands to study interactions with glycan-binding proteins,^8,9^ as standards for glycan structure determination of biological samples,^10,11^ and as probes to examine the molecular basis of glycoconjugate biosynthesis and as starting materials for glycoprotein synthesis.^12^ This need has stimulated the development of automated chemical and enzymatic synthesis for complex carbohydrates.^13–16^ Glycosylations by glycosyl transferases^12^ are attractive and proceed in a regio- and stereoselective manner, making it possible to prepare eukaryotic oligosaccharides including *N*- and *O*-glycans, glycosphingolipids, and glycosylaminoglycans.^23–26^

The scope of automated and other streamlined enzymatic approaches for complex glycan synthesis is still limited, and for example, current methods are not amenable to the preparation of glycans having complex branched architectures. The preparation of such compounds requires the integration of enzymatic and chemical protocols to construct, in a controlled manner, each antenna of a multibranched structure. Furthermore, platforms based on immobilized enzymes,^13,50–52^ one-pot multienzyme cascade reactions,^53^ or sulfonate-tagged acceptors^54^ cannot easily switch between buffer systems. This is a limitation, because different glycosyl transferases require different buffer conditions for optimal performance.

Here, we describe the first automation platform that can perform enzymatic and chemical manipulations. It made it possible, for the first time, to prepare asymmetrically branched *N*-glycans and sulfated poly-LacNAc derivatives in an automated fashion. It employs versatile acceptors modified by an apolar Nap-tag, and only three highly standardized catch and release modules can perform compound extraction to give products ready for the subsequent transformation. We have found that the Nap-tag is sufficiently apolar to capture sulfated and large *N*-glycans on a C18 resin. Washing with 40% aqueous acetonitrile efficiently releases glycans ranging from a dimer to a septendecimer into a small volume. Removal of volatile organic solvents by continuous airflow gives a product in water ready for the next reaction. It makes it possible to employ an optimal buffer for a subsequent enzymatic transformation. The alternative use of a benzyl carbamate CBz proved insufficient to retain large oligosaccharides,^70^ and furthermore, a fluorenylmethyloxycarbonate (Fmoc) containing linker^71^ is cleaved under the basic reaction conditions required for the removal of an NHTFA moiety. The methodology integrates glycosyltransferase catalyzed glycosylations, the use of the modified sugar nucleotide donor UDP-GlcNHTFA, and chemical manipulations including base-mediated TFA removal, azido transfer and azido reduction, Boc protection and deprotection, and amine acylation. The latter transformations are important for *stop-and-go* chemoenzymatic synthetic strategies in which unnatural monosaccharide residues are introduced to temporarily disable specific sites from enzymatic modification.^32,33,35–45^ The strategy also exploits that eukaryotic glycans have modular architectures in which a limited number of structural elements can be assembled in different ways to generate enormous structural diversity.^6^ As a result, a limited number of glycosyl transferases can provide access to large numbers of structurally diverse glycans. In this study, only 11 human glycosyl- and sulfotransferases were employed to prepare highly complex glycans. Reaction conditions for each enzyme have been optimized, and as a result, multistep syntheses can be reliably performed. Double couplings are performed for enzymatic transformations that are known to proceed slowly. It is also possible to program the workflow in a way that offline analysis by LC-MS precedes the next enzymatic transformation. The glycosynthesizer can handle 30 mg of material and can also perform parallel reactions, thereby providing opportunities for larger scale synthesis. It is envisaged that automated chemoenzymatic synthesis can be further streamlined by monitoring product formation in an automated fashion with the feedback control of the subsequent reaction. In this respect, automated LC-MS analysis, which integrates liquid chromatography and mass spectrometry with robotics and software for automated sample preparation, data acquisition, and peak integration, is well established in metabolomics, drug monitoring, and clinical diagnostics.^72^ It is expected that automated LC-MS analysis can be integrated in the ISYNTH AI SWING workstation and its Autosuite software. The platform already has the capability to collect a sample for analysis, which is amenable to automated LC-MS analysis. Computer guided decision making can be based on the consumption of starting material and product appearance. A double coupling can be performed in case the starting material is remaining. It is also expected that a reaction sequence can be paused when the automated analysis is ambiguous enough for manual intervention. The planning of a chemoenzymatic reaction sequence requires a detailed understanding of the biosynthesis of complex glycans. It is the expectation that machine learning approaches can be developed for synthetic planning similar to the hierarchical and programmable one-pot synthesis of oligosaccharides developed by Wong and co-workers.^73^

Although many eukaryotic glycosyltransferases have been described and can be recombinantly expressed,^27,28^ not every glycosidic bond can be installed enzymatically. Chemoenzymatic methodologies are most advanced for *N*- and *O*-linked glycan, glycospingolipids, milk oligosaccharides, and glycosylaminoglycans. Technologies such as genome sequencing, gene synthesis, and recombinant protein expression offer opportunities to identify and produce many more glycosyltransferases, thereby expanding the capabilities of enzyme-mediated oligosaccharide assembly. Currently, a limited number of noncanonical sugar nucleotide donors are available for stop-and-go glycosylation strategies.^32,45^ Bump-and-hole genetic engineering strategies^74^ may produce glycosyl transferases that can tolerate a wider range of artificial entities, thereby expanding the capabilities of chemoenzymatic synthesis. Enzymatic glycosylations are rather slow, and the multistep reaction sequence takes multiple days. Genetic engineering technologies can also be considered to improve the kinetic performance of enzymes. Automated chemical- and enzyme-mediated syntheses are highly complementary, and the combined use of these methodologies will greatly expand the chemical space that can be assessed by synthesis.

## Supplementary Material



## Notes

General materials and methods, preparation of nucleoside donors and glycosyltransferase expression and purification, extraction of sialylglycopeptide (SGP) from egg yolk powder, preparation of 2-naphthylmethyl chloroformate and general procedure for installation of Nap-tag, preparation of Ni-NTA and C18 SPE cartridges and optimization of the elution conditions of Nap-tagged saccharides, methods for LC-MS analysis, comparison of hydrophobic tags, optimizing dual purification strategy for enzymatic reactions, enzymes and reagent solutions storage, settings for automated liquid transfer, automation programs, automated reaction cycles and LC-MS traces, NMR nomenclature, general procedure for removal of 2-naphthylmethyl carbamate (Nap)-tag, and copies of NMR spectra (PDF)
