# Supplementary material for: An Automation Platform for the Chemoenzymatic Synthesis of Complex Sulfated and Branched Glycans
Source: J Am Chem Soc. 2026 Mar 6;148(10):11020–32. doi: 10.1021/jacs.5c22181 (PMC13003436; doi:10.1021/jacs.5c22181)

Supporting Information

An Automation Platform for the Chemoenzymatic Synthesis of Complex Sulfated and Branched Glycans

Saptashwa Chakraborty,[1] Kyle Minder,[1,2] Anthony Robert Prudden,[1] Geert-Jan Boons[1,2,3]*

[1] Complex Carbohydrate Research Center, University of Georgia, Athens, Georgia 30602, United States

[2] Department of Chemistry, University of Georgia, Athens, Georgia 30602, United States

[3] Chemical Biology and Drug Discovery, Utrecht Institute for Pharmaceutical Sciences, and Bijvoet Center for Biomolecular Research, Utrecht University, 3584 CG Utrecht, The Netherlands

Table of Content **Page**

1. General materials and methods	S3
2. Preparation of nucleoside donors and glycosyltransferase expression and purification	S3
3. Extraction of sialylglycopeptide (SGP) from egg yolk powder	S3
4. Preparation of 2-naphthylmethyl chloroformate and general procedure for installation of Nap-tag	S4
5. Preparation of Ni-NTA and C18 SPE cartridge and optimization of the minimum elution volume of Nap-tagged saccharides	S5
6. Methods for LC-MS	S6
7. Comparison of hydrophobic tag retention on C18 resin	S8
8. Optimizing a dual purification strategy for enzymatic reactions	S8
9. Enzymes and reagent solutions storage	S9
10. Settings for automated liquid transfer	S10
11. General procedure for the automated SPE modules	S11
12. Building an automation program; supplementary tables for automated enzymatic reaction	S14
13. Automated reaction cycles and LC-MS traces	S64
14. NMR nomenclature	S81
15. Characterization data	S82
16. General procedure for removal of 2-naphthylmethyl carbamate (Nap)-tag	S104
17. References	S105
18. Copies of NMR spectra	S106

1. General materials and methods

Organic reactions were performed under an atmosphere of argon using anhydrous solvents unless otherwise noted. Proton nuclear magnetic resonance (^{1}H-NMR) spectra were recorded on a Bruker 600 (at 600 MHz), Bruker 900 (at 900 MHz) or Bruker 1100 (at 1100 MHz); multiplicities were given as singlet (s), broad signal (br), doublet (d), doublet of doublets (dd), triplet (t) or multiplet (m). Carbon nuclear magnetic resonance (^{13}C) spectra were recorded on Bruker 600 (at 150 MHz). Spectra were assigned using gCOSY, HSQCAD, zTOCSY, and NOESY analysis. LC traces were performed using a Shimadzu LC-ESI-IT TOF with either an XBridge Amide BEH 3.5 μm, 2.1 mm × 150 mm column (Waters), a Halo C18 2.7μm, 3 mm × 20 mm column, or a Halo Penta-HILIC 2 μm, 2.1 mm × 100 mm column. LC MS traces were plotted in Excel sheet with time converted to minutes and relative intensity data (TIC). Colors are changed using Adobe Illustrator. Thin-layer chromatography (TLC) was performed on Merck silica gel 60 F254-coated aluminum sheets. TLC plates were detected with UV-absorption (254 nm) and sprayed with 10%(v/v) sulfuric acid in ethanol, followed by heating for visualization. Flash column chromatography was performed on silica gel (40-63μm). Chemical reagents were purchased from Sigma-Aldrich and TCI America.

2. Preparation of nucleoside donors and glycosyltransferase expression and purification

Recombinant human glycosyl transferases including α-1,3-mannosyl-glycoprotein 2-β-Nacetylglucosaminyltransferase (GnT-I), α-1,6-mannosyl-glycoprotein 2-β-Nacetylglucosaminyltransferase (GnT-II), alpha-1,3-mannosyl-glycoprotein 4-beta-Nacetylglucosaminyltransferase B (GnT-IVB), alpha-1,6-mannosylglycoprotein 6-beta-Nacetylglucosaminyltransferase (GnT-V), beta-1,4-mannosyl-glycoprotein 4-beta-Nacetylglucosaminyltransferase (GnT-III), β-1,4-galactosyltransferase 1 (B4GALT1), β-1,4-galactosyltransferase 4 (B4GALT4), β-galactosideα-2,6-sialyltransferase 1 (ST6Gal1), and β-galactoside-α-2, 3-sialyltransferase 4 (ST3GAL4) Fucosyltransferase 8 (FUT8), Fucosyltransferase 6 (FUT6) were expressed according to published protocol.[1-3] β-N-acetylglucosaminidase S was purchased from New England Biolabs (Catalog # P0744S). Nucleoside donors were prepared on a gram scale according to previously reported methods. Non-cannonical UDP-GlcNHTFA was prepared by utilizing a one-pot three-enzyme combination as previously reported protocol.[4]

3. Extraction of sialylglycopeptide (SGP) from egg yolk powder

SGP was isolated from commercially available egg yolk powder using a modified version of a previously reported method.[5] In short, Egg yolk powder (3 lbs) was suspended in acetone (2.0 L), stirred at room temperature for 2 h, and filtered to remove lipids and other acetone-soluble materials; this step was repeated twice. The resulting solid was sequentially treated with 70% (v/v) aqueous acetone (2.0 L) and 40% (v/v) aqueous

acetone (2.0 L), each with 2 h stirring, followed by filtration. The final 40% aqueous acetone filtrate was retained, concentrated under reduced pressure at 37 °C, and stored at 0 °C for 12 h. The solution was centrifuged (10 min, 3500 rpm) to remove insoluble material, and the supernatant was washed with dichloromethane, centrifuged again, and the aqueous phase was collected. This was concentrated in vacuo to a minimal volume and subjected to size-exclusion chromatography using a Bio-Gel G50 column with 100mM ammonium bicarbonate as eluent. Desired fractions were pooled and lyophilized to yield pure SGP as a fluffy white powder (0.5 g SGP per pound egg yolk powder). Next, SGP (900 mg) was dissolved in 4 mL of Tris buffer (100 mM, pH 8.0) containing 5 mM $CaCl_2$, resulting in a final reaction concentration of 10 mM. Pronase from *Streptomyces griseus* (Sigma-Aldrich, 300 mg) was added in portions, and the reaction mixture was incubated at 50 °C for 4 days, until complete consumption of the starting material was confirmed by ESI-MS. An equal volume of ethanol was added, and the mixture was shaken and stored at 0 °C to precipitate residual proteins. The supernatant was collected, concentrated under reduced pressure, and purified by size-exclusion chromatography using Bio-Gel G-50 (Cytiva) with 100 mM ammonium bicarbonate as the eluent. Product-containing fractions were combined and lyophilized to yield the asparagine-linked N-glycan as a white solid. To a solution of this heterogeneous N-glycan (400mg, 10mM reaction conc.) in sodium acetate buffer (50 mM, pH 5.5) containing 5 mM $CaCl_2$, neuraminidase from *Clostridium perfringens* (New England Biolabs; 50 µL) and β-galactosidase from *Aspergillus niger* (Megazyme; 100 µL) were added, and the mixture was incubated at 37 °C for 18 h. Complete removal of N-acetyl neuraminic acid and galactose was confirmed by monitoring ESI-MS spectra. An equal volume of cold ethanol was added to the reaction mixture, which was then stored at 0 °C to precipitate protein residues. The supernatant was collected, concentrated under reduced pressure, and purified by size-exclusion chromatography using Bio-Gel P2 (BioRad) with 100 mM ammonium bicarbonate as the eluent. Carbohydrate-positive fractions were combined and lyophilized to afford the desired product as a white amorphous solid.

4. Preparation of 2-naphthylmethyl chloroformate and general procedure for installation of Nap-tag

4a. Synthesis of 2-naphthylmethyl chloroformate

A flame-dried flask containing sodium carbonate (dried under reduced pressure with a heat gun) was charged with a solution of triphosgene (1.41 g, 4.74 mmol) in anhydrous toluene (15 mL) at 0 °C under an inert atmosphere. The mixture was stirred at 0 °C for 30 min, after which a solution of 2-naphthylmethanol (500 mg, 3.16 mmol) in anhydrous toluene (5 mL) was added dropwise. The reaction mixture was then allowed to warm to room temperature and stirred for 18 h. Upon completion of the reaction as adjudged by TLC analysis, the resulting suspension was filtered to remove solids, and the filtrate was concentrated under reduced pressure. The crude residue was co-evaporated with toluene

(4 × 30 mL) until a white solid formed. The solid was triturated with hexane and filtered to afford 2-naphthylmethyl chloroformate as a white solid in 88% isolated yield (615 mg isolated).

^{1}H NMR (Chloroform-d3, 600 MHz): δ (ppm) 5.47 (s, 2H, CH_2-Nap), 7.86-7.45 (m, 7H, aromatic). **^{13}C NMR (Chloroform-d3, 150 MHz): δ (ppm) :** 150.74 (carbonyl) 133.53, 133.04, 130.64, 128.80, 128.64, 128.17, 127.78, 126.91, 126.64, 125.88 (aromatic ring), 73.63 (-CH_2).

4b. General procedure for installation of 2-naphthylmethyl carbamate (Nap) tag

To a stirred solution of free amine acceptors (1.0 equiv) in *tert*-butanol/water (1:1, v/v) was added sodium bicarbonate (1.5 equiv), followed by 2-naphthylmethyl chloroformate (1.2 equiv). The reaction mixture was stirred at 45 °C for 12 h. Upon completion of the reaction as monitored by TLC and LC-MS traces, the mixture was filtered to remove insoluble solids, and the filtrate was concentrated under reduced pressure. The crude product was purified by silica gel flash chromatography or size exclusion chromatography to afford the desired Nap-tagged product.

5. Preparation of Ni-NTA and C18 SPE cartridge and optimization of the minimum elution volume of Nap-tagged saccharides

5a. Preparation of Ni-NTA cartridge

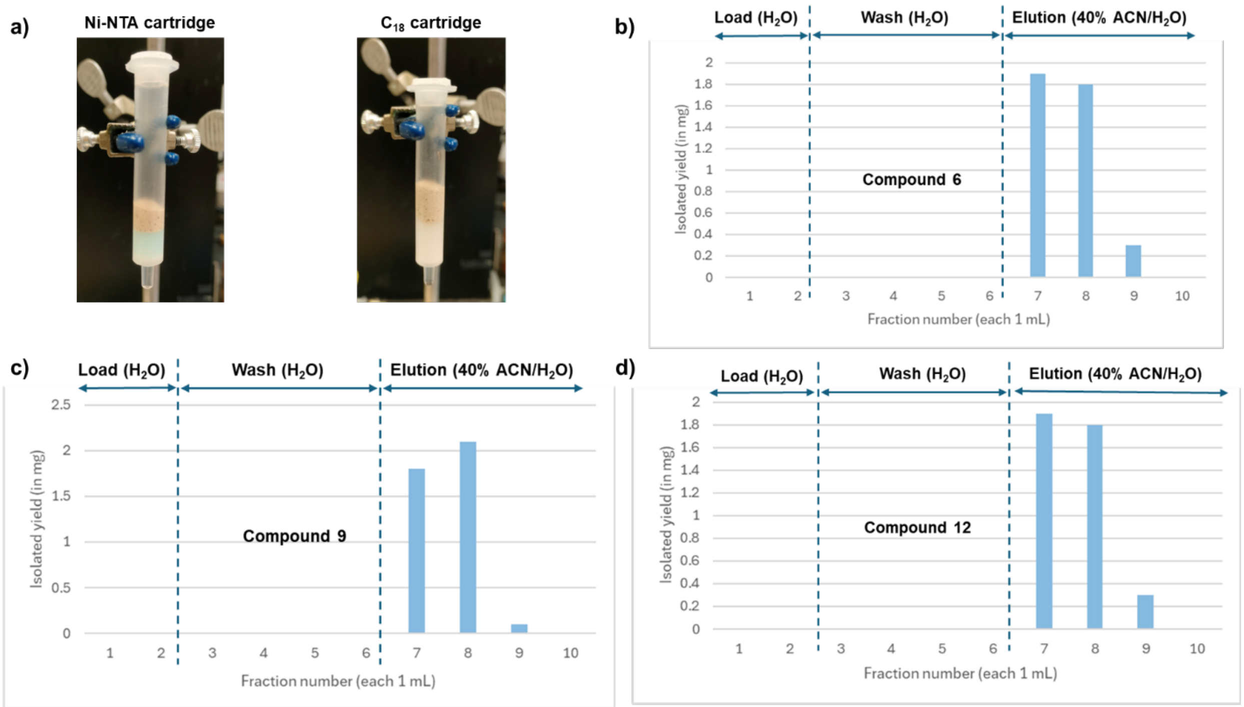

Figure S1. (a) Ni-NTA and C18 cartridge used for SPEs. (b-d) Minimum elution volume of compounds **6**, **9**,and **12,** respectively.

A 3 mL cartridge (Agilent Technologies, #12131008) fitted with a corresponding frit (Agilent Technologies, #12131020) was packed with a slurry of HisPur™ Ni-NTA (Thermo Scientific) suspended in 20% ethanol. The slurry was added until the settled resin bed reached a volume of approximately 0.2 mL. Once the desired resin volume was achieved, 0.1 mL of sand was carefully layered on top to stabilize the resin bed and prevent disruption. The packed column was then equilibrated by washing with water.

5b. Preparation of C18 cartridge

A 3 mL cartridge (Agilent Technologies, #12131008) fitted with a corresponding frit (Agilent Technologies, #12131020) was packed with a slurry of Bondapak C18 resin (Waters, #17070910) suspended in acetonitrile. The slurry was added until the settled resin bed reached a volume of approximately 0.4 mL. Once the desired resin volume was achieved, 0.2 mL of sand was carefully layered on top to stabilize the resin bed and prevent disruption. The packed column was then equilibrated by sequentially washing with pure acetonitrile, followed by a gradient from acetonitrile to water, and finally with 2 mL of pure water to ensure the resin was fully conditioned for subsequent use.

5c. Minimum elution volume of Nap-tagged saccharides

5 mg of each compound, **6**, **9**, and **12**, were dissolved in 2 mL of water and loaded onto the C18 cartridge for adsorption. Following loading, the resin was washed with 4 mL of water. No detectable elution of the Nap-tagged compounds was observed in the wash fractions, indicating that the compounds remained bound to the resin under aqueous conditions. This demonstrates that a 4 mL water wash is sufficient to remove residual reagents, salts, and buffers typically present in micromolar to millimolar scale enzymatic or chemical transformations, without prematurely eluting the target compounds. To identify a suitable single-set elution condition, different ratios of acetonitrile in water were tested. Each condition was evaluated by collecting 1 mL fractions and lyophilizing them to monitor the recovery of the compound. It was determined that a 40% acetonitrile in water mixture was sufficient to effectively elute all three compounds, ranging from disaccharides (compounds **6** and **9**) to an oligosaccharide (compound **12**), within 3 mL volume (Fig. S1 b-d).

6. Methods for LC-MS

6a. Methods for analytical C18 column LC traces

Analytical C18 traces were performed on a Shimadzu LC-ESI-IT-TOF with a HALO C18 2.7 µm, 3 mm × 20 mm column at a flow rate of 0.20 mL/min, injection volume of 5 µL (100 mM conc). Mobile phase A was 10 mM ammonium formate in water, adjusted to pH 3.5 with formic acid; mobile phase B was 100% acetonitrile. The general condition using a linear gradient is as follows;

Time (min)	%A	%B
0	100	0
20	50	50
21	20	80
25	20	80
30	100	0

6b. Methods for analytical Xbridge Amide BEH HILIC column LC traces

Analytical traces were performed on a Shimadzu LC-ESI-IT-TOF with an XBridge Amide BEH 3.5 µm, 2.1 mm × 150 mm column at a flow rate of 0.25 mL/min, injection volume of 5 µL (100 mM conc). Mobile phase A was 10 mM ammonium formate in water, adjusted to pH 3.5 with formic acid; mobile phase B was 100% acetonitrile. The general condition using a linear gradient is as follows;

Time (min)	%A	%B
0	20	80
18	50	50
20	25	20
24	25	25
26	20	80
30	20	80

6c. Methods for analytical Halo Penta-HILIC column LC traces

Analytical traces were performed on a Shimadzu LC-ESI-IT-TOF with a Halo Penta-HILIC 2 µm, 2.1 mm × 100 mm column at a flow rate of 0.20 mL/min, injection volume of 5 µL (100 mM conc). Mobile phase A was 10 mM ammonium formate in water, adjusted to pH 3.5 with formic acid; mobile phase B was 100% acetonitrile. The general condition using a linear gradient is as follows:

Time (min)	%A	%B
0	20	80
18	70	30
20	80	20
24	80	20
26	20	80
30	20	80

7. Comparison of hydrophobic tag retention on C18 resin

To enable a C18-based "catch-and-release" workflow for the synthesis of highly charged sulfated glycans and complex N-glycans, the apolar tag must impart sufficient hydrophobicity to ensure that these highly polar intermediates remain retained on the resin during washing. To evaluate suitable hydrophobic tags, we modified the α-amine of the asparagine moiety of N-glycan **11** with a Cbz group to generate compound **SI1**, and, in parallel, installed a 2-naphthylmethyl (Nap) tag to produce compound **12**. A mixture prepared by combining equal volumes of **SI1** and **12** (2 mg/mL each) was analyzed on a Shimadzu IT-TOF LC–MS equipped with a Halo C18 column. As expected, compound **12** exhibited a substantially longer retention time (14.91 min) compared to **SI1** (9.97 min), indicating that the Nap tag confers greater hydrophobicity (Fig. S2) and is therefore better suited for a C18-based catch-and-release purification strategy. We did not pursue an Fmoc group as an alternative hydrophobic tag because its base-labile nature is incompatible with the chemical manipulations required in the automated workflow.

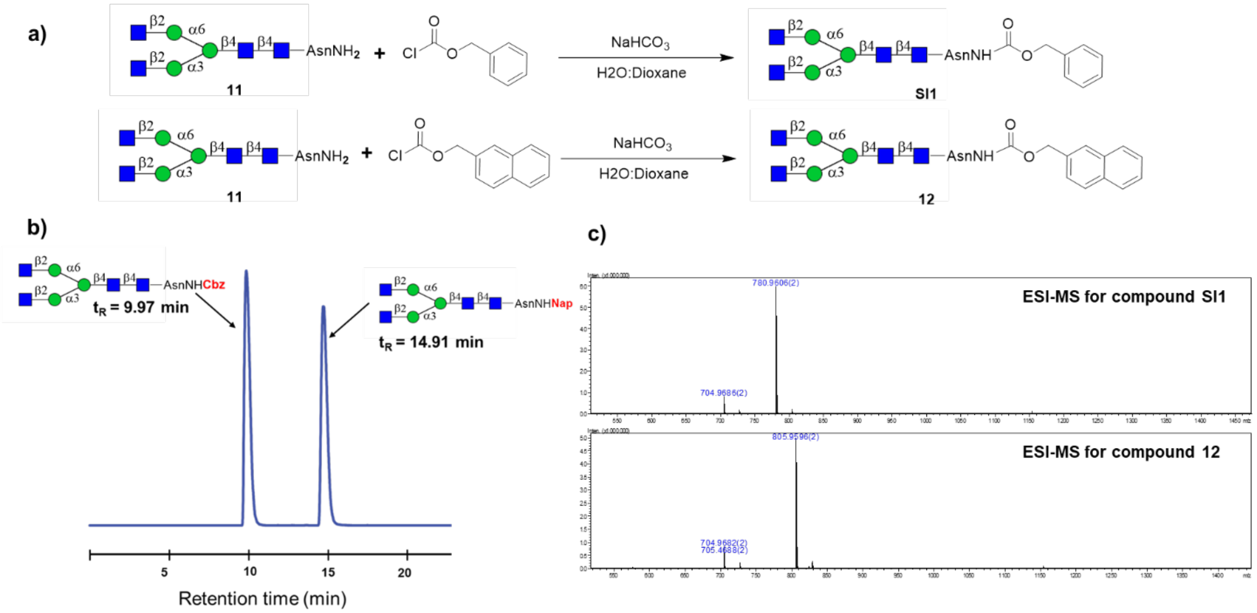

Figure S2. Retention of apolar tags on a HALO C18 analytical column.

8. Optimizing a dual purification strategy for enzymatic reactions

We envisioned a dual-purification strategy using sequential Ni–NTA and C18 resins to isolate Nap-tagged products directly from reaction mixtures. As a representative example, compound **12** was dissolved in Tris-buffered media, followed by addition of UDP–Gal, $MnCl_2$, and recombinant His_6-tagged human B4GALT1, and the reaction was incubated at 37 °C for 12 h. After confirming reaction completion by LC–MS, the mixture was first

passed through a Ni–NTA cartridge to remove the His_6-tagged B4GALT1, and the flow-through was collected. The resulting solution was then loaded onto a C18 cartridge, washed with 4 mL of water, and eluted with 3 mL of 40% acetonitrile in water to release the Nap-tagged product. LC–MS analysis of the flow-through, wash, and elution fractions revealed that all buffer components and excess nucleotide donor were efficiently removed during the washing step, while no detectable product was observed in either the flow-through or the water wash, confirming strong resin retention and validating the effectiveness of the purification protocol for isolating Nap-tagged compound **13** (Fig. S3).

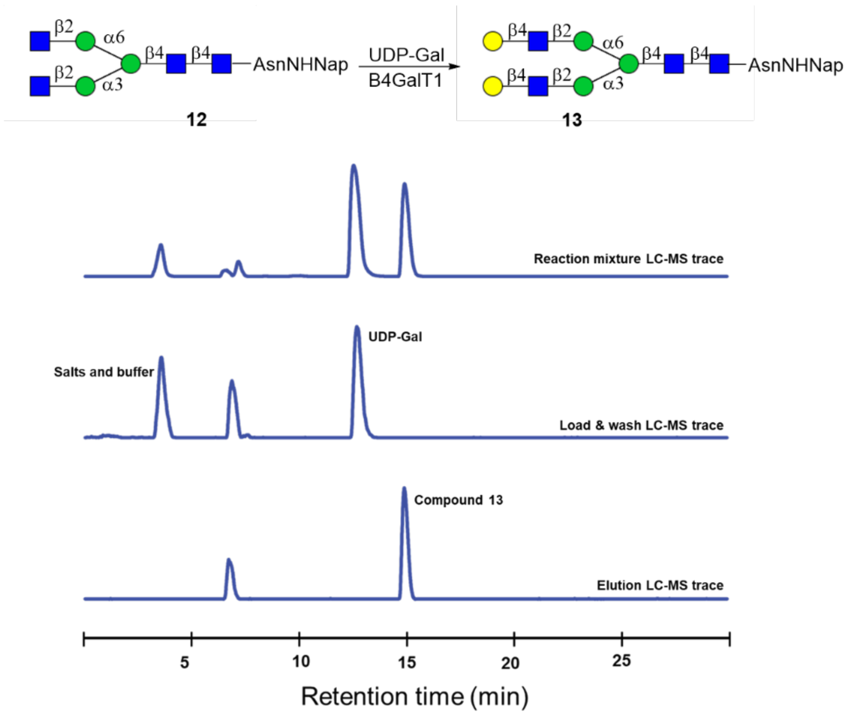

Figure S3. LC traces for reaction mixture, load & wash fraction, and elution fraction on Xbridge Amide HILIC column with method 4b.

9. Enzymes and reagent solutions storage

The following reagents are prepared in 8 mL vials and stored at 4 °C in rack **D**:

Sugar nucleotide solutions: UDP-GlcNAc (100 mM), UDP-GlcNHTFA (100 mM), UDP-Gal (100 mM), GDP-Fuc (100 mM), and CMP-Neu5Ac (100 mM) in water. **Human glycosyltransferases:** GnT-I (1 mg/mL), GnT-II (1 mg/mL), GnT-V (1 mg/mL), B4GALT1 (1.7 mg/mL), B4GALT4 (1 mg/mL), B3GNT2 (1 mg/mL), ST6GAL1 (2 mg/mL), ST3GAL4 (1 mg/mL), FUT5 (1 mg/mL), CHST1 (1 mg/mL), and CHST2 (1 mg/mL); **Bacterial glycosyltransferase:** *H. pylori* β-1,3-N-acetylglucosaminyltransferase (3 mg/mL);

glycosidases including Neuraminidase from *Clostridium perfringens* (2 mg/mL), β-galactosidase from *Aspergillus niger* (1mg/mL), and β-N-acetylglucosaminidase S (1mg/mL); other enzymes such as calf intestine alkaline phosphatase (CIAP, 20 U/mL); **Salts and Chemical reagents:** $MnCl_2$ (1 M), $MgCl_2$ (1 M), KCl (1 M), DTT (100 mM), 3′-Phosphoadenosine-5′-phosphosulfate (PAPS)(160 mM), Imidazole-1-sulfonyl azide (10mg/mL), PMe_3 (1M in THF)

Additionally, the following buffers and solutions are stored in 60 mL vials at room temperature in rack **G**:

$NaHCO_3$ (1M), Na_2CO_3 (1 M), NaOH (20% w/v), Boc-anhydride in THF (100 mg/mL), Tris-HCl buffer (1 M, pH 7.5), Sodium cacodylate buffer (1 M, pH 6.5), HEPES buffer (1 M, pH 7.2), MES buffer (1 M, pH 6.5) and sodium acetate buffer (1 M, pH 5).

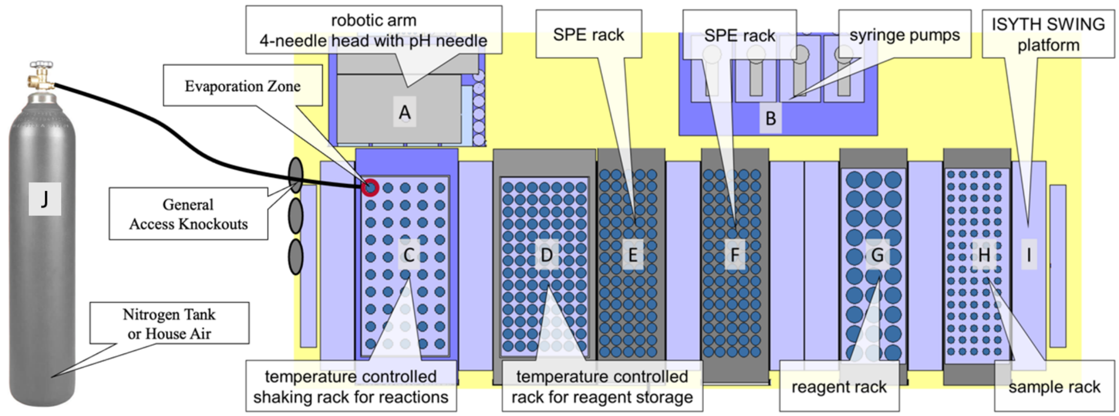

Figure S4. Schematic of the modified Chemspeed Isynth AI Swing platform for automated synthesis.

10. Settings for automated liquid transfer

(i). Volume ≥ 100 μL: source speed at 5 mL/min, destination speed at 10 mL/min, extra volume = 0 mL, airgap volume = 0.1 mL.

(ii). Volume <100 μL: source speed at 1 mL/min, destination speed at 5 mL/min, extra volume = 0 mL, airgap volume = 0.1 mL.

(iii). Transferring reaction solution onto SPE columns: source speed at 10 mL/min, destination speed at 0.3 mL/min, extra volume = 0.3 mL, airgap volume = 0.1 mL. Both the extra volume and airgap volume are disposed of in the destination zone.

(iv). Transferring the washing and eluting solution onto SPE: source speed at 10 mL/min, destination speed at 0.3 mL/min, extra volume = 0.3 mL, airgap volume = 0.1 mL. Both the extra volume and airgap volume are disposed of in the destination zone.

(v). Transferring the SPE eluting solution onto evaporation zone: source speed at 5 mL/min, destination speed at 0.3 mL/min, extra volume = 0.5 mL, airgap volume = 0.2 mL. Both the extra volume and airgap volume are disposed of in the destination zone.

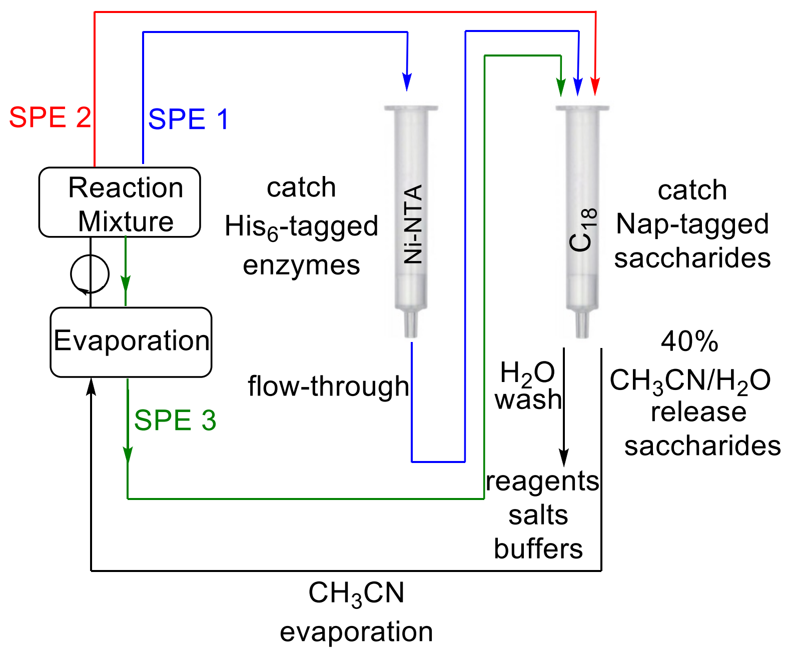

Figure S5. All SPE modules.

11. General procedure for the automated SPE modules

11a. General procedure for the automated SPE1 modules

i) Enzymatic reaction: The needle head transferred 250 µL of 1 M corresponding aqueous buffer from rack **G** to a reaction tube in rack **C** containing the starting material in water, and the resulting solution (0.5 mmolar–5 mmolar reaction) was incubated at 37 °C with vortexing at 300 rpm. Subsequently, the needle head administered the corresponding sugar nucleotide donor and metal ions (25 µL, 1 M) from rack **D** to the reaction tube. The enzyme was then added, and the reaction mixture was incubated at 37 °C with continuous vortexing at 300 rpm for the programmed duration. For MS analysis, the needle head dispensed 100 µL of water into a 2 mL vial placed in rack **H**, into which 10 µL of the reaction mixture was added

ii) Solid phase extraction: Upon completion of the enzymatic reaction, the needle head transferred the reaction mixture onto a pre-equilibrated Ni-NTA column set to the ‘collect’ position in rack **E**. The flow-through was collected, and the column was rinsed with 300 µL of water. The collected solution was subsequently transferred to a holding station and then onto a pre-equilibrated C18 column set to the ‘waste’ position, where the flow-through was discarded. The needle head then administered 4 mL of water onto the C18 cartridge for washing, with the wash solution directed to waste. The rack was then shifted to the ‘collect’ position according to the program, and 3 mL of 40% acetonitrile in water was dispensed onto the C18 cartridge. The resulting acetonitrile–water eluate was transferred to the evaporation zone at rack **C**, where the solution was subjected to controlled airflow at 37 °C with vortexing at 300 rpm to evaporate the acetonitrile component (Fig S5).

11b. General procedure for the automated SPE2 module

i) Chemical reaction: The needle head transferred 250 µL of 1 M corresponding aqueous buffer from rack **G** to a reaction tube in rack **C** containing the starting material in water, and the resulting solution (0.5 mmolar–5 mmolar reaction) was incubated at 37 °C with vortexing at 300 rpm. Subsequently, the needle head administered the corresponding reagents from rack **D** or **G** to the reaction tube and the reaction mixture was incubated at 37 °C with continuous vortexing at 300 rpm for the programmed duration. For MS analysis, the needle head dispensed 100 µL of water into a 2 mL vial placed in rack **H**, into which 10 µL of the reaction mixture was added.

ii) Solid phase extraction: Upon completion of the enzymatic reaction, the needle head transferred the reaction mixture onto a pre-equilibrated C18 column set to the ‘waste’ position, where the flow-through was discarded. The needle head then administered 4 mL of water onto the C18 cartridge for washing, with the wash solution directed to waste. The rack was then shifted to the ‘collect’ position according to the program, and 3 mL of 40% acetonitrile in water was dispensed onto the C18 cartridge. The resulting acetonitrile–water eluate was transferred to the evaporation zone at rack **C**, where the solution was subjected to controlled airflow at 37 °C with vortexing at 300 rpm to evaporate the acetonitrile component (Fig S5).

11c. General procedure for the automated SPE3 module

i) Chemical reaction: The needle head transferred 250 µL of 1 M corresponding aqueous buffer (if required) from rack **G** to a reaction tube in rack **C** containing the starting material in water, and the resulting solution (0.5 mmolar–5 mmolar reaction) was incubated at 37 °C with vortexing at 300 rpm. Subsequently, the needle head administered the corresponding reagents from rack **D** or **G** to the reaction tube and the reaction mixture was incubated at 37 °C with continuous vortexing at 300 rpm for the programmed

duration. For MS analysis, the needle head dispensed 100 µL of water into a 2 mL vial placed in rack **H**, into which 10 µL of the reaction mixture was added.

ii) Evaporation of organic component from reaction mixture: Upon completion of the enzymatic reaction, the needle head transferred the reaction mixture to a the evaporation zone at rack **C**, where the solution was subjected to controlled airflow at 37 °C with vortexing at 300 rpm for to evaporate of the volatile organic component (THF, Boc-anhydride, trimethyl phosphene etc.) prior to C18 SPE.

iii) Solid phase extraction: Upon evaporation of the organic components from the reaction mixture, the needle head transferred the reaction mixture onto a pre-equilibrated C18 column set to the 'waste' position, where the flow-through was discarded. The needle head then administered 4 mL of water onto the C18 cartridge for washing, with the wash solution directed to waste. The rack was then shifted to the 'collect' position according to the program, and 3 mL of 40% acetonitrile in water was dispensed onto the C18 cartridge. The resulting acetonitrile–water eluate was transferred to the evaporation zone at rack **C**, where the solution was subjected to controlled airflow at 37 °C with vortexing at 300 rpm to evaporate the acetonitrile component (Fig S5).

12. Building an automation program

Table S1. Installation of β1,4-Gal by B4GalT1.

Step	Task	Reagents and Operation
1	Transfer	i) Transfer 250 uL Tris-HCL buffer (1M, pH 7.5) to the reaction vial containing starting material (2 mL water)<br>ii) Transfer UDP-Gal (1.5 eq), 25 uL $MnCl_2$ (1M), B4GalT1 (0.2% wt/wt) to the reaction tube
2	Reaction	Vortex the reaction mixture at 300 rpm for 12h at 37 °C
3	MS Sample	10 uL sample taken from the reaction mixture for ESI MS-analysis.
4	SPE1 purification	i) transfer reaction mixture to Ni-NTA cartridge (collect position) at 0.3 mL/min rate<br>ii) transfer water to rinse the reaction tube (2 mL)<br>iii) transfer the rinse solution to Ni-NTA cartridge<br>iv) transfer collected flow-through solution to holding station<br>v) transfer solution from holding station to C18 cartridge (waste position) at 0.3 mL/min rate<br>vi) transfer 4 mL water to C18 cartridge (waste position) for wash<br>vii) transfer 3 mL 40% ACN-water to C18 cartridge (collect position) and collect the released solution
5	Transfer	Transfer collected 40% ACN-water solution containing purified product to evaporation zone
6	Evaporation	Evaporate the acetonitrile from the eluted solution under controlled airflow at 37 °C with vortexing at 300 rpm for 45 min
7	MS Sample	10 uL sample taken from the reaction mixture for ESI MS-analysis, and the MS result showed that the reaction is complete.
8	Transfer	i) transfer 1.8 mL water from the evaporation zone to the next reaction tube<br>ii) transfer water to rinse the evaporation tube (200 uL)<br>iii) transfer rinsed solution to the next reaction tube

Automation program B4GalT1

Task	Name	Parameter	Description	Estimated Time	Task Number
1	Control Cryostat	Thermostat ON on zone cryo_cooling	Cooling to 4C	00 sec	1
2	Heat / Cool	Thermostat ON on zone electro_heating		00 sec	2
3	Sequence	Execute Once		15 hours 53 min 56 sec	3
1	B4GalT1 Reaction	Execute Once	Reaction	12 hours 02 min 24 sec	3.1
1	Add Reagents	Execute Once		33 sec	3.1.1
1	Transfer Volumetrically	Transfer liquid from 1M Tris pH 7.5 to Reaction1 with Needle Head #1	Add buffer	33 sec	3.1.1.1
2	Other Reagents	Execute If 'Other_Reagents = 1'	Add other reagents	00 sec	3.1.1.2
1	Transfer Volumetrically	Transfer liquid from UDP-Gal to Reaction1 with Needle Head #1	transfer sugar nucleotide solution to reaction	01 min 03 sec	3.1.1.2.1
2	Transfer Volumetrically	Transfer liquid from Mn_solution to Reaction1 with Needle Head #1	transfer Mn solution to reaction_vial	01 min 01 sec	3.1.1.2.2
3	Transfer Volumetrically	Transfer liquid from Enzyme_B4GalT1 to Reaction1 with Needle Head #1	transfer enzyme solution to reaction_vial	01 min 23 sec	3.1.1.2.3
<insert sub tasks here>					
<insert sub tasks here>					
2	Synthesis	Execute Once		12 hours 00 min 02 sec	3.1.2
1	Stir	Agitation ON on zone shaker		02 sec	3.1.2.1
2	Wait	Waiting for 12:00:00 hours	12 h wait	12 hours 00 min 00 sec	3.1.2.2
<insert sub tasks here>					
3	MS Analysis	Execute Once		01 min 48 sec	3.1.3
4	Extend Reaction?	Execute Once		00 sec	3.1.4
1	Show Dialog	Yes/No/Stop-Dialog: 'First round of B4GalT1 reaction finished. Do you want to add more reagents (Select Yes) or directly go to SPE (select No)?'		00 sec	3.1.4.1
<insert sub tasks here>					
5	Extend Reaction	Execute If 'Extend_Reaction = 1'		00 sec	3.1.5
<insert sub tasks here>					
2	Continue?	Execute Once		02 sec	3.2
3	Purification	Execute Once		03 hours 06 min 26 sec	3.3
1	Stir	Agitation OFF on zone shaker		02 sec	3.3.1
2	Ni-NTA Purification	Execute Once	Ni-NTA Purification	55 min 24 sec	3.3.2
3	C18 Purification	Execute Once	C18 Purification	02 hours 11 min 00 sec	3.3.3
<insert sub tasks here>					
4	Evaporation	Execute Once		45 min 04 sec	3.4
<insert sub tasks here>					
4	Control Cryostat	Thermostat OFF on zone cryo_cooling		00 sec	4
5	Heat / Cool	Thermostat OFF on zone electro_heating		00 sec	5

Table S2. Installation of β1,3-GlcNAc by B3GnT2.

Step	Task	Reagents and Operation
1	Transfer	i) Transfer 250 uL HEPES buffer (1M, pH 7) to the reaction vial containing starting material (2 mL water)<br>ii) Transfer UDP-GlcNAc (1.5 eq), 25 uL $MgCl_2$ (1 M), 25 uL DTT (100 mM), 10 uL KCl (1 M), B3GNT2 (1% wt/wt) to the reaction tube
2	Reaction	Vortex the reaction mixture at 300 rpm for 16 h at 37 °C
3	MS Sample	10 uL sample taken from the reaction mixture for ESI MS-analysis
4	SPE1 purification	i) transfer reaction mixture to Ni-NTA cartridge (collect position) at 0.3 mL/min rate<br>ii) transfer water to rinse the reaction tube (2 mL)<br>iii) transfer the rinse solution to Ni-NTA cartridge<br>iv) transfer collected flow-through solution to holding station<br>v) transfer solution from holding station to C18 cartridge (waste position) at 0.3 mL/min rate<br>vi) transfer 4 mL water to C18 cartridge (waste position) for wash<br>vii) transfer 3 mL 40% ACN-water to C18 cartridge (collect position) and collect the released solution
5	Transfer	Transfer collected 40% ACN-water solution containing purified product to evaporation zone
6	Evaporation	Evaporate the acetonitrile from the eluted solution under controlled airflow at 37 °C with vortexing at 300 rpm for 45 min
7	MS Sample	10 uL sample taken from the reaction mixture for ESI MS-analysis, and the MS result showed that the reaction is complete.
8	Transfer	i) transfer 1.8 mL water from the evaporation zone to the next reaction tube<br>ii) transfer water to rinse the evaporation tube (200 uL)<br>iii) transfer rinsed solution to the next reaction tube

Automation program B3GNT2

Task	Name	Parameter	Description	Estimated Time	Task Number
1	Control Cryostat	Thermostat ON on zone cryo_cooling	Cooling to 4C	00 sec	1
2	Heat / Cool	Thermostat ON on zone electro_heating		00 sec	2
3	Sequence	Execute Once		19 hours 52 min 09 se	3
1	B3GNT2 GlcNAc-Beta-1,3-Gal Reaction	Execute Once	Reaction	16 hours 02 min 23 se	3.1
1	Add Reagents	Execute Once		33 sec	3.1.1
1	Transfer Volumetrically	Transfer liquid from 1M HEPES pH 7.5 to Reaction1 with Needle Head #1	Add buffer	33 sec	3.1.1.1
2	Other Reagents	Execute If 'Other_Reagents = 1'	Add other reagents	00 sec	3.1.1.2
1	Transfer Volumetrically	Transfer liquid from UDP-GlcNAc to Reaction1 with Needle Head #1	transfer sugar nucleotide solution to reaction_vial	01 min 04 sec	3.1.1.2.1
2	Transfer Volumetrically	Transfer liquid from Mg_solution to Reaction1 with Needle Head #1	transfer Mg solution to reaction_vial	01 min 01 sec	3.1.1.2.2
3	Transfer Volumetrically	Transfer liquid from KCl_solution to Reaction1 with Needle Head #1	transfer KCl solution to reaction_vial	01 min 01 sec	3.1.1.2.3
4	Transfer Volumetrically	Transfer liquid from DTT_solution to Reaction1 with Needle Head #1	transfer DTT solution to reaction_vial	01 min 01 sec	3.1.1.2.4
5	Transfer Volumetrically	Transfer liquid from enzyme_B3GNT2 to Reaction1 with Needle Head #1	transfer enzyme solution to reaction_vial	01 min 21 sec	3.1.1.2.5
<insert sub tasks here>					
<insert sub tasks here>					
2	Synthesis	Execute Once		16 hours 00 min 02 se	3.1.2
3	MS Analysis	Execute Once		01 min 47 sec	3.1.3
4	Extend Reaction?	Execute Once		00 sec	3.1.4
1	Show Dialog	Yes/No/Stop-Dialog: 'First round of B3GNT2 finished. Do you want to add more reagents (Select Yes) or directly go to SPE (select No)?'		00 sec	3.1.4.1
<insert sub tasks here>					
5	Extend Reaction	Execute If 'Extend_Reaction = 1'		00 sec	3.1.5
<insert sub tasks here>					
2	Continue?	Execute Once		02 sec	3.2
3	Purification	Execute Once		03 hours 04 min 40 se	3.3
1	Stir	Agitation OFF on zone shaker		02 sec	3.3.1
2	Ni-NTA Purification	Execute Once	Ni-NTA Purification	55 min 24 sec	3.3.2
3	C18 Purification	Execute Once	C18 Purification	02 hours 09 min 14 se	3.3.3
<insert sub tasks here>					
4	Evaporation	Execute Once		45 min 04 sec	3.4
<insert sub tasks here>					
4	Control Cryostat	Thermostat OFF on zone cryo_cooling		00 sec	4
5	Heat / Cool	Thermostat OFF on zone electro_heating		00 sec	5

Table S3. Installation of β1,4-Gal by B4GalT4.

Step	Task	Reagents and Operation
1	Transfer	i) Transfer 250 uL Tris-HCL buffer (1M, pH 7.5) to the reaction vial containing starting material (2 mL water)<br>ii) Transfer UDP-Gal (1.5 eq), 25 uL $MnCl_2$ (1M), B4GalT4 (1% wt/wt) to the reaction tube
2	Reaction	Vortex the reaction mixture at 300 rpm for 12 h at 37 °C
3	MS Sample	10 uL sample taken from the reaction mixture for ESI MS-analysis.
4	SPE1 purification	i) transfer reaction mixture to Ni-NTA cartridge (collect position) at 0.3 mL/min rate<br>ii) transfer water to rinse the reaction tube (2 mL)<br>iii) transfer the rinse solution to Ni-NTA cartridge<br>iv) transfer collected flow-through solution to holding station<br>v) transfer solution from holding station to C18 cartridge (waste position) at 0.3 mL/min rate<br>vi) transfer 4 mL water to C18 cartridge (waste position) for wash<br>vii) transfer 3 mL 40% ACN-water to C18 cartridge (collect position) and collect the released solution
5	Transfer	Transfer collected 40% ACN-water solution containing purified product to evaporation zone
6	Evaporation	Evaporate the acetonitrile from the eluted solution under controlled airflow at 37 °C with vortexing at 300 rpm for 45 min
7	MS Sample	10 uL sample taken from the reaction mixture for ESI MS-analysis, and the MS result showed that the reaction is complete.
8	Transfer	i) transfer 1.8 mL water from the evaporation zone to the next reaction tube<br>ii) transfer water to rinse the evaporation tube (200 uL)<br>iii) transfer rinsed solution to the next reaction tube

Automation program B4GalT4

Task	Name	Parameter	Description	Estimated Time	Task Number
1	Control Cryostat	Thermostat ON on zone cryo_cooling	Cooling to 4C	00 sec	1
2	Heat / Cool	Thermostat ON on zone electro_heating		00 sec	2
3	Sequence	Execute Once		15 hours 53 min 56 sec	3
1	B4GalT4 Reaction	Execute Once	Reaction	12 hours 02 min 24 sec	3.1
1	Add Reagents	Execute Once		33 sec	3.1.1
1	Transfer Volumetrically	Transfer liquid from 1M Tris pH 7.5 to Reaction1 with Needle Head #1	Add buffer	33 sec	3.1.1.1
2	Other Reagents	Execute If 'Other_Reagents = 1'	Add other reagents	00 sec	3.1.1.2
1	Transfer Volumetrically	Transfer liquid from UDP-Gal to Reaction1 with Needle Head #1	transfer sugar nucleotide solution to reaction_	01 min 03 sec	3.1.1.2.1
2	Transfer Volumetrically	Transfer liquid from Mn_solution to Reaction1 with Needle Head #1	transfer Mn solution to reaction_vial	01 min 01 sec	3.1.1.2.2
3	Transfer Volumetrically	Transfer liquid from Enzyme_B4GalT4 to Reaction1 with Needle Head #1	transfer enzyme solution to reaction_vial	01 min 21 sec	3.1.1.2.3
<insert sub tasks here>					
<insert sub tasks here>					
2	Synthesis	Execute Once		12 hours 00 min 02 sec	3.1.2
3	MS Analysis	Execute Once		01 min 48 sec	3.1.3
4	Extend Reaction?	Execute Once		00 sec	3.1.4
1	Show Dialog	Yes/No/Stop-Dialog: 'First round of B4GalT4 reaction finished. Do you want to add more reagents (Select Yes) or directly go to SPE (select No)?'		00 sec	3.1.4.1
<insert sub tasks here>					
5	Extend Reaction	Execute If 'Extend_Reaction = 1'		00 sec	3.1.5
<insert sub tasks here>					
2	Continue?	Execute Once		02 sec	3.2
3	Purification	Execute Once		03 hours 06 min 26 sec	3.3
1	Stir	Agitation OFF on zone shaker		02 sec	3.3.1
2	Ni-NTA Purification	Execute Once	Ni-NTA Purification	55 min 24 sec	3.3.2
3	C18 Purification	Execute Once	C18 Purification	02 hours 11 min 00 sec	3.3.3
<insert sub tasks here>					
4	Evaporation	Execute Once		45 min 04 sec	3.4
<insert sub tasks here>					
4	Control Cryostat	Thermostat OFF on zone cryo_cooling		00 sec	4
5	Heat / Cool	Thermostat OFF on zone electro_heating		00 sec	5

Table S4. Installation of β1,2-GlcNAc or β1,2-GlcNHTFA by GnT-I.

Step	Task	Reagents and Operation
1	Transfer	i) Transfer 250 uL MES buffer (1M, pH 6.5) to the reaction vial containing starting material (2 mL water)<br>ii) Transfer UDP-GlcNAc or UDP-GlcNHTFA (1.5 eq), 25 uL $MnCl_2$ (1M), GnT-I (1% wt/wt) to the reaction tube
2	Reaction	Vortex the reaction mixture at 300 rpm for 16h at 37 °C
3	MS Sample	10 uL sample taken from the reaction mixture for ESI MS-analysis.
4	SPE1 purification	i) transfer reaction mixture to Ni-NTA cartridge (collect position) at 0.3 mL/min rate<br>ii) transfer water to rinse the reaction tube (2 mL)<br>iii) transfer the rinse solution to Ni-NTA cartridge<br>iv) transfer collected flow-through solution to holding station<br>v) transfer solution from holding station to C18 cartridge (waste position) at 0.3 mL/min rate<br>vi) transfer 4 mL water to C18 cartridge (waste position) for wash<br>vii) transfer 3 mL 40% ACN-water to C18 cartridge (collect position) and collect the released solution
5	Transfer	Transfer collected 40% ACN-water solution containing purified product to evaporation zone
6	Evaporation	Evaporate the acetonitrile from the eluted solution under controlled airflow at 37 °C with vortexing at 300 rpm for 45 min
7	MS Sample	10 uL sample taken from the reaction mixture for ESI MS-analysis, and the MS result showed that the reaction is complete.
8	Transfer	i) transfer 1.8 mL water from the evaporation zone to the next reaction tube<br>ii) transfer water to rinse the evaporation tube (200 uL)<br>iii) transfer rinsed solution to the next reaction tube

Automation program GnT-I to install β1,2-GlcNHTFA

Task	Name	Parameter	Description	Estimated Time	Task Number
1	Control Cryostat	Thermostat ON on zone cryo_cooling	Cooling to 4C	00 sec	1
2	Heat / Cool	Thermostat ON on zone electro_heating		00 sec	2
3	Sequence	Execute Once		19 hours 53 min 55 sec	3
1	MGAT1 GlcNHTFA Rea	Execute Once	Reaction	16 hours 02 min 22 sec	3.1
1	Add Reagents	Execute Once		33 sec	3.1.1
1	Transfer Volumetrically	Transfer liquid from MES Buffer pH 6.5 to Reaction1 with Needle Head #1	Add buffer	33 sec	3.1.1.1
2	Other Reagents	Execute If 'Other_Reagents = 1'	Add other reagents	00 sec	3.1.1.2
1	Transfer Volumetrically	Transfer liquid from UDP-GlcNTFA to Reaction1 with Needle Head #1	transfer sugar nucleotide solution to reaction_vial	01 min 04 sec	3.1.1.2.1
2	Transfer Volumetrically	Transfer liquid from Mn_solution to Reaction1 with Needle Head #1	transfer Mn solution to reaction_vial	01 min 01 sec	3.1.1.2.2
3	Transfer Volumetrically	Transfer liquid from Enzyme MGAT1 to Reaction1 with Needle Head #1	transfer enzyme solution to reaction_vial	01 min 21 sec	3.1.1.2.3
<insert sub tasks here>					
<insert sub tasks here>					
2	Synthesis	Execute Once		16 hours 00 min 02 sec	3.1.2
3	MS Analysis	Execute Once		01 min 47 sec	3.1.3
4	Extend Reaction?	Execute Once		00 sec	3.1.4
1	Show Dialog	Yes/No/Stop-Dialog: 'First round of MGAT1 reaction finished. Do you want to add more reagents (Select Yes) or directly go to SPE (select No)?'		00 sec	3.1.4.1
<insert sub tasks here>					
5	Extend Reaction	Execute If 'Extend_Reaction = 1'		00 sec	3.1.5
<insert sub tasks here>					
2	Continue?	Execute Once		02 sec	3.2
3	Purification	Execute Once		03 hours 06 min 26 sec	3.3
1	Stir	Agitation OFF on zone shaker		02 sec	3.3.1
2	Ni-NTA Purification	Execute Once	Ni-NTA Purification	55 min 24 sec	3.3.2
3	C18 Purification	Execute Once	C18 Purification	02 hours 11 min 00 sec	3.3.3
<insert sub tasks here>					
4	Evaporation	Execute Once		45 min 04 sec	3.4
<insert sub tasks here>					
4	Control Cryostat	Thermostat OFF on zone cryo_cooling		00 sec	4
5	Heat / Cool	Thermostat OFF on zone electro_heating		00 sec	5

Table S5. Installation of β1,2-GlcNAc or β1,2-GlcNHTFA by GnT-II.

Step	Task	Reagents and Operation
1	Transfer	i) Transfer 250 uL MES buffer (1M, pH 6.5) to the reaction vial containing starting material (2 mL water)<br>ii) Transfer UDP-GlcNAc or UDP-GlcNHTFA (1.5 eq), 25 uL $MnCl_2$ (1M), GnT-II (1% wt/wt) to the reaction tube
2	Reaction	Vortex the reaction mixture at 300 rpm for 16h at 37 °C
3	MS Sample	10 uL sample taken from the reaction mixture for ESI MS-analysis.
4	SPE1 purification	i) transfer reaction mixture to Ni-NTA cartridge (collect position) at 0.3 mL/min rate<br>ii) transfer water to rinse the reaction tube (2 mL)<br>iii) transfer the rinse solution to Ni-NTA cartridge<br>iv) transfer collected flow-through solution to holding station<br>v) transfer solution from holding station to C18 cartridge (waste position) at 0.3 mL/min rate<br>vi) transfer 4 mL water to C18 cartridge (waste position) for wash<br>vii) transfer 3 mL 40% ACN-water to C18 cartridge (collect position) and collect the released solution
5	Transfer	Transfer collected 40% ACN-water solution containing purified product to evaporation zone
6	Evaporation	Evaporate the acetonitrile from the eluted solution under controlled airflow at 37 °C with vortexing at 300 rpm for 45 min
7	MS Sample	10 uL sample taken from the reaction mixture for ESI MS-analysis, and the MS result showed that the reaction is complete.
8	Transfer	i) transfer 1.8 mL water from the evaporation zone to the next reaction tube<br>ii) transfer water to rinse the evaporation tube (200 uL)<br>iii) transfer rinsed solution to the next reaction tube

Automation program GnT-II to install β1,2-GlcNA

Task	Name	Parameter	Description	Estimated Time	Task Number
1	Control Cryostat	Thermostat ON on zone cryo_cooling	Cooling to 4C	00 sec	1
2	Heat / Cool	Thermostat ON on zone electro_heating		00 sec	2
3	Sequence	Execute Once		19 hours 53 min 55 sec	3
1	MGAT2 GlcNAc Reaction	Execute Once	Reaction	16 hours 02 min 22 sec	3.1
1	Add Reagents	Execute Once		33 sec	3.1.1
1	Transfer Volumetrically	Transfer liquid from MES Buffer pH 6.5 to Reaction1 with Needle Head #1	Add buffer	33 sec	3.1.1.1
2	Other Reagents	Execute If 'Other_Reagents = 1'	Add other reagents	00 sec	3.1.1.2
1	Transfer Volumetrically	Transfer liquid from UDP-GlcNAc to Reaction1 with Needle Head #1	transfer sugar nucleotide solution to reaction_vial	01 min 04 sec	3.1.1.2.1
2	Transfer Volumetrically	Transfer liquid from Mn_solution to Reaction1 with Needle Head #1	transfer Mn solution to reaction_vial	01 min 01 sec	3.1.1.2.2
3	Transfer Volumetrically	Transfer liquid from enzyme MGAT2 to Reaction1 with Needle Head #1	transfer enzyme solution to reaction_vial	01 min 22 sec	3.1.1.2.3
<insert sub tasks here>					
<insert sub tasks here>					
2	Synthesis	Execute Once		16 hours 00 min 02 sec	3.1.2
1	Stir	Agitation ON on zone shaker		02 sec	3.1.2.1
2	Wait	Waiting for 16:00:00 hours		16 hours 00 min 00 sec	3.1.2.2
<insert sub tasks here>					
3	MS Analysis	Execute Once		01 min 47 sec	3.1.3
4	Extend Reaction?	Execute Once		00 sec	3.1.4
1	Show Dialog	Yes/No/Stop-Dialog: 'First round of MGAT2 reaction finished. Do you want to add more reagents (Select Yes) or directly go to SPE (select No)?'		00 sec	3.1.4.1
<insert sub tasks here>					
5	Extend Reaction	Execute If 'Extend_Reaction = 1'		00 sec	3.1.5
<insert sub tasks here>					
2	Continue?	Execute Once		02 sec	3.2
3	Purification	Execute Once		03 hours 06 min 26 sec	3.3
1	Stir	Agitation OFF on zone shaker		02 sec	3.3.1
2	Ni-NTA Purification	Execute Once	Ni-NTA Purification	55 min 24 sec	3.3.2
3	C18 Purification	Execute Once	C18 Purification	02 hours 11 min 00 sec	3.3.3
<insert sub tasks here>					
4	Evaporation	Execute Once		45 min 04 sec	3.4
<insert sub tasks here>					
4	Control Cryostat	Thermostat OFF on zone cryo_cooling		00 sec	4
5	Heat / Cool	Thermostat OFF on zone electro_heating		00 sec	5

Table S6. Installation of β1,2-GlcNHTFA by GnT-V.

Step	Task	Reagents and Operation
1	Transfer	i) Transfer 250 uL sodium cacodylate buffer (1M, pH 6.5) to the reaction vial containing starting material (2 mL water)<br>ii) Transfer UDP-GlcNHTFA (1.5 eq), 25 uL $MnCl_2$ (1M), GnT-II (3% wt/wt) to the reaction tube
2	Reaction	Vortex the reaction mixture at 300 rpm for 16h at 37 °C
3	MS Sample	10 uL sample taken from the reaction mixture for ESI MS-analysis.
4	SPE1 purification	i) transfer reaction mixture to Ni-NTA cartridge (collect position) at 0.3 mL/min rate<br>ii) transfer water to rinse the reaction tube (2 mL)<br>iii) transfer the rinse solution to Ni-NTA cartridge<br>iv) transfer collected flow-through solution to holding station<br>v) transfer solution from holding station to C18 cartridge (waste position) at 0.3 mL/min rate<br>vi) transfer 4 mL water to C18 cartridge (waste position) for wash<br>vii) transfer 3 mL 40% ACN-water to C18 cartridge (collect position) and collect the released solution
5	Transfer	Transfer collected 40% ACN-water solution containing purified product to evaporation zone
6	Evaporation	Evaporate the acetonitrile from the eluted solution under controlled airflow at 37 °C with vortexing at 300 rpm for 45 min
7	MS Sample	10 uL sample taken from the reaction mixture for ESI MS-analysis, and the MS result showed that the reaction is complete.
8	Transfer	i) transfer 1.8 mL water from the evaporation zone to the next reaction tube<br>ii) transfer water to rinse the evaporation tube (200 uL)<br>iii) transfer rinsed solution to the next reaction tube

Automation program GnT-V to install β1,2-GlcNHTFA

Task	Name	Parameter	Description	Estimated Time	Task Number
1	Control Cryostat	Thermostat ON on zone cryo_cooling	Cooling to 4C	00 sec	1
2	Heat / Cool	Thermostat ON on zone electro_heating		00 sec	2
3	Sequence	Execute Once		19 hours 53 min 55 sec	3
1	MGAT5 GlcNHTFA Reactic	Execute Once	Reaction	16 hours 02 min 22 sec	3.1
1	Add Reagents	Execute Once		33 sec	3.1.1
1	Transfer Volumetrically	Transfer liquid from 1M MES Buffer pH 6.5 to Reaction1 with Needle Head #1	Add buffer	33 sec	3.1.1.1
2	Other Reagents	Execute If 'Other_Reagents = 1'	Add other reagents	00 sec	3.1.1.2
1	Transfer Volumetrically	Transfer liquid from UDP-GlcNHTFA to Reaction1 with Needle Head #1	transfer sugar nucleotide solution to reaction_	01 min 04 sec	3.1.1.2.1
2	Transfer Volumetrically	Transfer liquid from Mn_solution to Reaction1 with Needle Head #1	transfer Mn solution to reaction_vial	01 min 01 sec	3.1.1.2.2
3	Transfer Volumetrically	Transfer liquid from Enzyme MGAT5 to Reaction1 with Needle Head #1	transfer enzyme solution to reaction_vial	01 min 22 sec	3.1.1.2.3
<insert sub tasks here>					
<insert sub tasks here>					
2	Synthesis	Execute Once		16 hours 00 min 02 sec	3.1.2
1	Stir	Agitation ON on zone shaker		02 sec	3.1.2.1
2	Wait	Waiting for 16:00:00 hours	16 h wait	16 hours 00 min 00 sec	3.1.2.2
<insert sub tasks here>					
3	MS Analysis	Execute Once		01 min 47 sec	3.1.3
4	Extend Reaction?	Execute Once		00 sec	3.1.4
1	Show Dialog	Yes/No/Stop-Dialog: 'First round of MGAT5 reaction finished. Do you want to add more reagents (Select Yes) or directly go to SPE (select No)?'		00 sec	3.1.4.1
<insert sub tasks here>					
5	Extend Reaction	Execute If 'Extend_Reaction = 1'		00 sec	3.1.5
<insert sub tasks here>					
2	Continue?	Execute Once		02 sec	3.2
3	Purification	Execute Once		03 hours 06 min 26 sec	3.3
1	Stir	Agitation OFF on zone shaker		02 sec	3.3.1
2	Ni-NTA Purification	Execute Once	Ni-NTA Purification	55 min 24 sec	3.3.2
3	C18 Purification	Execute Once	C18 Purification	02 hours 11 min 00 sec	3.3.3
<insert sub tasks here>					
4	Evaporation	Execute Once		45 min 04 sec	3.4
<insert sub tasks here>					
4	Control Cryostat	Thermostat OFF on zone cryo_cooling		00 sec	4
5	Heat / Cool	Thermostat OFF on zone electro_heating		00 sec	5

Table S7. Installation of β1,3-GlcNHTFA by *H. pylori* β3GlcNAcT.

Step	Task	Reagents and Operation
1	Transfer	i) Transfer 250 uL Tris buffer (1M, pH 7) to the reaction vial containing starting material (2 mL water)<br>ii) Transfer UDP-GlcNHTFA (1.5 eq), 25 uL $MgCl_2$ (1 M), 25 uL DTT (100 mM), *H. pylori* β3GlcNAcT (1% wt/wt) to the reaction tube
2	Reaction	Vortex the reaction mixture at 300 rpm for 12 h at 30 °C
3	MS Sample	10 uL sample taken from the reaction mixture for ESI MS-analysis
4	SPE1 purification	i) transfer reaction mixture to Ni-NTA cartridge (collect position) at 0.3 mL/min rate<br>ii) transfer water to rinse the reaction tube (2 mL)<br>iii) transfer the rinse solution to Ni-NTA cartridge<br>iv) transfer collected flow-through solution to holding station<br>v) transfer solution from holding station to C18 cartridge (waste position) at 0.3 mL/min rate<br>vi) transfer 4 mL water to C18 cartridge (waste position) for wash<br>vii) transfer 3 mL 40% ACN-water to C18 cartridge (collect position) and collect the released solution
5	Transfer	Transfer collected 40% ACN-water solution containing purified product to evaporation zone
6	Evaporation	Evaporate the acetonitrile from the eluted solution under controlled airflow at 37 °C with vortexing at 300 rpm for 45 min
7	MS Sample	10 uL sample taken from the reaction mixture for ESI MS-analysis, and the MS result showed that the reaction is complete.
8	Transfer	i) transfer 1.8 mL water from the evaporation zone to the next reaction tube<br>ii) transfer water to rinse the evaporation tube (200 uL)<br>iii) transfer rinsed solution to the next reaction tube

Automation program *H. pylori* β3GlcNAcT to install β1,3-GlcNHTFA

Task	Name	Parameter	Description	Estimated Time	Task Number
1	Control Cryostat	Thermostat ON on zone cryo_cooling	Cooling to 4C	00 sec	1
2	Heat / Cool	Thermostat ON on zone electro_heating	heating at 30C	00 sec	2
3	Sequence	Execute Once		15 hours 53 min 56 sec	3
1	Hp39 GlcNHTFA-Beta-1,3-Gal Reaction	Execute Once	Reaction	12 hours 02 min 23 sec	3.1
1	Add Reagents	Execute Once		33 sec	3.1.1
1	Transfer Volumetrically	Transfer liquid from 1M Tris pH 7.5 to Reaction1 with Needle Head #1	Add buffer	33 sec	3.1.1.1
2	Other Reagents	Execute If 'Other_Reagents = 1'	Add other reagents	00 sec	3.1.1.2
1	Transfer Volumetrically	Transfer liquid from UDP-GlcNHTFA to Reaction1 with Needle Head #1	transfer sugar nucleotide solution	01 min 04 sec	3.1.1.2.1
2	Transfer Volumetrically	Transfer liquid from Mg_solution to Reaction1 with Needle Head #1	transfer Mg solution to reaction_vial	01 min 01 sec	3.1.1.2.2
3	Transfer Volumetrically	Transfer liquid from KCl_solution to Reaction1 with Needle Head #1	transfer KCl solution to reaction_vial	01 min 01 sec	3.1.1.2.3
4	Transfer Volumetrically	Transfer liquid from DTT_solution to Reaction1 with Needle Head #1	transfer DTT solution to reaction_vial	01 min 01 sec	3.1.1.2.4
5	Transfer Volumetrically	Transfer liquid from Enzyme_Hp39 to Reaction1 with Needle Head #1	transfer enzyme solution to reaction_vial	01 min 21 sec	3.1.1.2.5
<insert sub tasks here>					
<insert sub tasks here>					
2	Synthesis	Execute Once		12 hours 00 min 02 sec	3.1.2
3	MS Analysis	Execute Once		01 min 47 sec	3.1.3
4	Extend Reaction?	Execute Once		00 sec	3.1.4
1	Show Dialog	Yes/No/Stop-Dialog: 'First round of Hp39 reaction finished. Do you want to add more reagents (Select Yes) or directly go to SPE (select No)		00 sec	3.1.4.1
<insert sub tasks here>					
5	Extend Reaction	Execute If 'Extend_Reaction = 1'		00 sec	3.1.5
<insert sub tasks here>					
2	Continue?	Execute Once		02 sec	3.2
3	Purification	Execute Once		03 hours 06 min 26 sec	3.3
1	Stir	Agitation OFF on zone shaker		02 sec	3.3.1
2	Ni-NTA Purification	Execute Once	Ni-NTA Purification	55 min 24 sec	3.3.2
3	C18 Purification	Execute Once	C18 Purification	02 hours 11 min 00 sec	3.3.3
<insert sub tasks here>					
4	Evaporation	Execute Once		45 min 04 sec	3.4
<insert sub tasks here>					
4	Control Cryostat	Thermostat OFF on zone cryo_cooling		00 sec	4

Table S8. Installation of α1,3-Fuc by FUT6.

Step	Task	Reagents and Operation
1	Transfer	i) Transfer 250 uL Tris buffer (1M, pH 7.5) to the reaction vial containing starting material (2 mL water)<br>ii) Transfer GDP-Fuc (1.5 eq), 25 uL $MnCl_2$ (1 M),FUT6 (1% wt/wt) to the reaction tube
2	Reaction	Vortex the reaction mixture at 300 rpm for 12 h at 37 °C
3	MS Sample	10 uL sample taken from the reaction mixture for ESI MS-analysis
4	SPE1 purification	i) transfer reaction mixture to Ni-NTA cartridge (collect position) at 0.3 mL/min rate<br>ii) transfer water to rinse the reaction tube (2 mL)<br>iii) transfer the rinse solution to Ni-NTA cartridge<br>iv) transfer collected flow-through solution to holding station<br>v) transfer solution from holding station to C18 cartridge (waste position) at 0.3 mL/min rate<br>vi) transfer 4 mL water to C18 cartridge (waste position) for wash<br>vii) transfer 3 mL 40% ACN-water to C18 cartridge (collect position) and collect the released solution
5	Transfer	Transfer collected 40% ACN-water solution containing purified product to evaporation zone
6	Evaporation	Evaporate the acetonitrile from the eluted solution under controlled airflow at 37 °C with vortexing at 300 rpm for 45 min
7	MS Sample	10 uL sample taken from the reaction mixture for ESI MS-analysis, and the MS result showed that the reaction is complete.
8	Transfer	i) transfer 1.8 mL water from the evaporation zone to the next reaction tube<br>ii) transfer water to rinse the evaporation tube (200 uL)<br>iii) transfer rinsed solution to the next reaction tube

Automation program FUT6

Task	Name	Parameter	Description	Estimated Time	Task Number
1	Control Cryostat	Thermostat ON on zone cryo_cooling	Cooling to 4C	00 sec	1
2	Heat / Cool	Thermostat ON on zone electro_heating		00 sec	2
3	Sequence	Execute Once		15 hours 53 min 56 sec	3
1	Alpha 1-3 Fucosylation Reaction	Execute Once	Reaction	12 hours 02 min 23 sec	3.1
1	Add Reagents	Execute Once		33 sec	3.1.1
1	Transfer Volumetrically	Transfer liquid from 1M Tris pH 7.5 to Reaction1 with Needle Head #1	Add buffer	33 sec	3.1.1.1
2	Other Reagents	Execute If 'Other_Reagents = 1'	Add other reagents	00 sec	3.1.1.2
1	Transfer Volumetrically	Transfer liquid from GDP-Fuc to Reaction1 with Needle Head #1	transfer sugar nucleotide GDP-fuc solution to	01 min 04 sec	3.1.1.2.1
2	Transfer Volumetrically	Transfer liquid from Mn_solution to Reaction1 with Needle Head #1	transfer Mn solution to reaction_vial	01 min 01 sec	3.1.1.2.2
3	Transfer Volumetrically	Transfer liquid from Enzyme FUT6 to Reaction1 with Needle Head #1	transfer enzyme solution to reaction_vial	01 min 22 sec	3.1.1.2.3
<insert sub tasks here>					
<insert sub tasks here>					
2	Synthesis	Execute Once		12 hours 00 min 02 sec	3.1.2
3	MS Analysis	Execute Once		01 min 47 sec	3.1.3
4	Extend Reaction?	Execute Once		00 sec	3.1.4
1	Show Dialog	Yes/No/Stop-Dialog: 'First round of FUT6 reaction is finished. Do you want to add more reagents (Select Yes) or directly go to SPE (select No)?		00 sec	3.1.4.1
<insert sub tasks here>					
5	Extend Reaction	Execute If 'Extend_Reaction = 1'		00 sec	3.1.5
<insert sub tasks here>					
2	Continue?	Execute Once		02 sec	3.2
3	Purification	Execute Once		03 hours 06 min 26 sec	3.3
1	Stir	Agitation OFF on zone shaker		02 sec	3.3.1
2	Ni-NTA Purification	Execute Once	Ni-NTA Purification	55 min 24 sec	3.3.2
3	C18 Purification	Execute Once	C18 Purification	02 hours 11 min 00 sec	3.3.3
<insert sub tasks here>					
4	Evaporation	Execute Once		45 min 04 sec	3.4
<insert sub tasks here>					
4	Control Cryostat	Thermostat OFF on zone cryo_cooling		00 sec	4
5	Heat / Cool	Thermostat OFF on zone electro_heating		00 sec	5

Table S9. Installation of 6-O-SO^{-}_3 on GlcNAc by CHST2.

Step	Task	Reagents and Operation
1	Transfer	i) Transfer 250 uL Tris buffer (1M, pH 7.5) to the reaction vial containing starting material (2 mL water)<br>ii) Transfer PAPS solution (1.5 eq), 25 uL $MnCl_2$ (1 M), CHST2 (10% wt/wt) to the reaction tube
2	Reaction	Vortex the reaction mixture at 300 rpm for 12 h at 37 °C
3	MS Sample	10 uL sample taken from the reaction mixture for ESI MS-analysis
4	SPE1 purification	i) transfer reaction mixture to Ni-NTA cartridge (collect position) at 0.3 mL/min rate<br>ii) transfer water to rinse the reaction tube (2 mL)<br>iii) transfer the rinse solution to Ni-NTA cartridge<br>iv) transfer collected flow-through solution to holding station<br>v) transfer solution from holding station to C18 cartridge (waste position) at 0.3 mL/min rate<br>vi) transfer 4 mL water to C18 cartridge (waste position) for wash<br>vii) transfer 3 mL 40% ACN-water to C18 cartridge (collect position) and collect the released solution
5	Transfer	Transfer collected 40% ACN-water solution containing purified product to evaporation zone
6	Evaporation	Evaporate the acetonitrile from the eluted solution under controlled airflow at 37 °C with vortexing at 300 rpm for 45 min
7	MS Sample	10 uL sample taken from the reaction mixture for ESI MS-analysis, and the MS result showed that the reaction is complete.
8	Transfer	i) transfer 1.8 mL water from the evaporation zone to the next reaction tube<br>ii) transfer water to rinse the evaporation tube (200 uL)<br>iii) transfer rinsed solution to the next reaction tube

Automation program CHST2

Task	Name	Parameter	Description	Estimated Time	Task Number
1	Control Cryostat	Thermostat ON on zone cryo_cooling	Cooling to 4C	00 sec	1
2	Heat / Cool	Thermostat ON on zone electro_heating		00 sec	2
3	Sequence	Execute Once		15 hours 53 min 56 sec	3
1	CHST2 Reaction	Execute Once	Reaction	12 hours 02 min 23 sec	3.1
1	Add Reagents	Execute Once		33 sec	3.1.1
1	Transfer Volumetrically	Transfer liquid from 1M Tris pH 7.5 to Reaction1 with Needle Head #1	Add buffer	33 sec	3.1.1.1
2	Other Reagents	Execute If 'Other_Reagents = 1'	Add other reagents	00 sec	3.1.1.2
1	Transfer Volumetrically	Transfer liquid from PAPS to Reaction1 with Needle Head #1	transfer PAPS solution to reaction_vial	01 min 04 sec	3.1.1.2.1
2	Transfer Volumetrically	Transfer liquid from Mn_solution to Reaction1 with Needle Head #1	transfer Mn solution to reaction_vial	01 min 01 sec	3.1.1.2.2
3	Transfer Volumetrically	Transfer liquid from CHST2 to Reaction1 with Needle Head #1	transfer enzyme solution to reaction_vial	01 min 23 sec	3.1.1.2.3
<insert sub tasks here>					
<insert sub tasks here>					
2	Synthesis	Execute Once		12 hours 00 min 02 sec	3.1.2
1	Stir	Agitation ON on zone shaker		02 sec	3.1.2.1
2	Wait	Waiting for 12:00:00 hours	12 h wait	12 hours 00 min 00 sec	3.1.2.2
<insert sub tasks here>					
3	MS Analysis	Execute Once		01 min 47 sec	3.1.3
4	Extend Reaction?	Execute Once		00 sec	3.1.4
1	Show Dialog	Yes/No/Stop-Dialog: 'First round of CHST2 reaction finished. Do you want to add more reagents (Select Yes) or directly go to SPE (select No)?'		00 sec	3.1.4.1
<insert sub tasks here>					
5	Extend Reaction	Execute If 'Extend_Reaction = 1'		00 sec	3.1.5
<insert sub tasks here>					
2	Continue?	Execute Once		02 sec	3.2
3	Purification	Execute Once		03 hours 06 min 26 sec	3.3
1	Stir	Agitation OFF on zone shaker		02 sec	3.3.1
2	Ni-NTA Purification	Execute Once	Ni-NTA Purification	55 min 24 sec	3.3.2
3	C18 Purification	Execute Once	C18 Purification	02 hours 11 min 00 sec	3.3.3
<insert sub tasks here>					
4	Evaporation	Execute Once		45 min 04 sec	3.4
<insert sub tasks here>					
4	Control Cryostat	Thermostat OFF on zone cryo_cooling		00 sec	4
5	Heat / Cool	Thermostat OFF on zone electro_heating		00 sec	5

Table S10. Installation of 6-O-SO_3^- on Gal by CHST1.

Step	Task	Reagents and Operation
1	Transfer	i) Transfer 250 uL Tris buffer (1M, pH 7.5) to the reaction vial containing starting material (2 mL water)<br>ii) Transfer PAPS solution (1.5 eq), 25 uL $MnCl_2$ (1 M), CHST1 (10% wt/wt) to the reaction tube
2	Reaction	Vortex the reaction mixture at 300 rpm for 12 h at 37 °C
3	MS Sample	10 uL sample taken from the reaction mixture for ESI MS-analysis
4	SPE1 purification	i) transfer reaction mixture to Ni-NTA cartridge (collect position) at 0.3 mL/min rate<br>ii) transfer water to rinse the reaction tube (2 mL)<br>iii) transfer the rinse solution to Ni-NTA cartridge<br>iv) transfer collected flow-through solution to holding station<br>v) transfer solution from holding station to C18 cartridge (waste position) at 0.3 mL/min rate<br>vi) transfer 4 mL water to C18 cartridge (waste position) for wash<br>vii) transfer 3 mL 40% ACN-water to C18 cartridge (collect position) and collect the released solution
5	Transfer	Transfer collected 40% ACN-water solution containing purified product to evaporation zone
6	Evaporation	Evaporate the acetonitrile from the eluted solution under controlled airflow at 37 °C with vortexing at 300 rpm for 45 min
7	MS Sample	10 uL sample taken from the reaction mixture for ESI MS-analysis, and the MS result showed that the reaction is complete.
8	Transfer	i) transfer 1.8 mL water from the evaporation zone to the next reaction tube<br>ii) transfer water to rinse the evaporation tube (200 uL)<br>iii) transfer rinsed solution to the next reaction tube

Automation program CHST1

Task	Name	Parameter	Description	Estimated Time	Task Number
1	Control Cryostat	Thermostat ON on zone cryo_cooling	Cooling to 4C	00 sec	1
2	Heat / Cool	Thermostat ON on zone electro_heating		00 sec	2
3	Sequence	Execute Once		15 hours 53 min 56 sec	3
1	CHST1 Reaction	Execute Once	Reaction	12 hours 02 min 23 sec	3.1
1	Add Reagents	Execute Once		33 sec	3.1.1
1	Transfer Volumetrically	Transfer liquid from 1M Tris pH 7.5 to Reaction1 with Needle Head #1	Add buffer	33 sec	3.1.1.1
2	Other Reagents	Execute If 'Other_Reagents = 1'	Add other reagents	00 sec	3.1.1.2
1	Transfer Volumetrically	Transfer liquid from PAPS to Reaction1 with Needle Head #1	transfer PAPS solution to reaction_vial	01 min 04 sec	3.1.1.2.1
2	Transfer Volumetrically	Transfer liquid from Mn_solution to Reaction1 with Needle Head #1	transfer Mn solution to reaction_vial	01 min 01 sec	3.1.1.2.2
3	Transfer Volumetrically	Transfer liquid from CHST1 to Reaction1 with Needle Head #1	transfer enzyme solution to reaction_vial	01 min 23 sec	3.1.1.2.3
<insert sub tasks here>					
<insert sub tasks here>					
2	Synthesis	Execute Once		12 hours 00 min 02 sec	3.1.2
1	Stir	Agitation ON on zone shaker		02 sec	3.1.2.1
2	Wait	Waiting for 12:00:00 hours	12 h wait	12 hours 00 min 00 sec	3.1.2.2
<insert sub tasks here>					
3	MS Analysis	Execute Once		01 min 47 sec	3.1.3
4	Extend Reaction	Execute If 'Extend_Reaction = 1'		00 sec	3.1.4
5	Show Dialog	Yes/No/Stop-Dialog: 'First round of CHST1 reaction finished. Do you want to add more reagents (Select Yes) or directly go to SPE (select No)?'		00 sec	3.1.5
<insert sub tasks here>					
2	Continue?	Execute Once		02 sec	3.2
3	Purification	Execute Once		03 hours 06 min 26 sec	3.3
1	Stir	Agitation OFF on zone shaker		02 sec	3.3.1
2	Ni-NTA Purification	Execute Once	Ni-NTA Purification	55 min 24 sec	3.3.2
3	C18 Purification	Execute Once	C18 purification	02 hours 11 min 00 sec	3.3.3
<insert sub tasks here>					
4	Evaporation	Execute Once		45 min 04 sec	3.4
<insert sub tasks here>					
4	Control Cryostat	Thermostat OFF on zone cryo_cooling		00 sec	4
5	Heat / Cool	Thermostat OFF on zone electro_heating		00 sec	5

Table S11. Installation of α2,3-Neu5Ac by ST3Gal4.

Step	Task	Reagents and Operation
1	Transfer	i) Transfer 250 uL sodium cacodylate (1M, pH 6.5) to the reaction vial containing starting material (2 mL water)<br>ii) Transfer CMP-Sia (1.5 eq), ST3Gal4 (1% wt/wt) to the reaction tube
2	Reaction	Vortex the reaction mixture at 300 rpm for 16 h at 37 °C
3	MS Sample	10 uL sample taken from the reaction mixture for ESI MS-analysis
4	SPE1 purification	i) transfer reaction mixture to Ni-NTA cartridge (collect position) at 0.3 mL/min rate<br>ii) transfer water to rinse the reaction tube (2 mL)<br>iii) transfer the rinse solution to Ni-NTA cartridge<br>iv) transfer collected flow-through solution to holding station<br>v) transfer solution from holding station to C18 cartridge (waste position) at 0.3 mL/min rate<br>vi) transfer 4 mL water to C18 cartridge (waste position) for wash<br>vii) transfer 3 mL 40% ACN-water to C18 cartridge (collect position) and collect the released solution
5	Transfer	Transfer collected 40% ACN-water solution containing purified product to evaporation zone
6	Evaporation	Evaporate the acetonitrile from the eluted solution under controlled airflow at 37 °C with vortexing at 300 rpm for 45 min
7	MS Sample	10 uL sample taken from the reaction mixture for ESI MS-analysis, and the MS result showed that the reaction is complete.
8	Transfer	i) transfer 1.8 mL water from the evaporation zone to the next reaction tube<br>ii) transfer water to rinse the evaporation tube (200 uL)<br>iii) transfer rinsed solution to the next reaction tube

Automation program ST3Gal4

Task	Name	Parameter	Description	Estimated Time	Task Number
1	Control Cryostat	Thermostat ON on zone cryo_cooling	Cooling to 4C	00 sec	1
2	Heat / Cool	Thermostat ON on zone electro_heating		00 sec	2
3	Sequence	Execute Once		19 hours 53 min 53 sec	3
1	ST3Gal4 Reaction	Execute Once	Reaction	16 hours 02 min 20 sec	3.1
1	Add Reagents	Execute Once		31 sec	3.1.1
1	Transfer Volumetrically	Transfer liquid from 1M Na-Cacodylate buffer pH 6.5 to Reaction1 with Needle Head #1	Add buffer	31 sec	3.1.1.1
2	Other Reagents	Execute If 'Other_Reagents = 1'	Add other reagents	00 sec	3.1.1.2
1	Transfer Volumetrically	Transfer liquid from CMP_Neu5Ac to Reaction1 with Needle Head #1	transfer sugar nucleotide solution to reaction_vial	01 min 04 sec	3.1.1.2.1
2	Transfer Volumetrically	Transfer liquid from Enzyme _ST3Gal4 to Reaction1 with Needle Head #1	transfer enzyme solution to reaction_vial	01 min 22 sec	3.1.1.2.2
<insert sub tasks here>					
<insert sub tasks here>					
2	Synthesis	Execute Once		16 hours 00 min 02 sec	3.1.2
3	MS Analysis	Execute Once		01 min 47 sec	3.1.3
4	Extend Reaction?	Execute Once		00 sec	3.1.4
1	Show Dialog	Yes/No/Stop-Dialog: 'First round of ST3Gal4 reaction finished. Do you want to add more reagents (Select Yes) or directly go to SPE (		00 sec	3.1.4.1
<insert sub tasks here>					
5	Extend Reaction	Execute If 'Extend_Reaction = 1'		00 sec	3.1.5
<insert sub tasks here>					
2	Continue?	Execute Once		02 sec	3.2
3	Purification	Execute Once		03 hours 06 min 26 sec	3.3
1	Stir	Agitation OFF on zone shaker		02 sec	3.3.1
2	Ni-NTA Purification	Execute Once	Ni-NTA Purification	55 min 24 sec	3.3.2
3	C18 Purification	Execute Once	C18 Purification	02 hours 11 min 00 sec	3.3.3
<insert sub tasks here>					
4	Evaporation	Execute Once		45 min 04 sec	3.4
<insert sub tasks here>					
4	Control Cryostat	Thermostat OFF on zone cryo_cooling		00 sec	4
5	Heat / Cool	Thermostat OFF on zone electro_heating		00 sec	5

Table S12. Installation of α2,6-Neu5Ac by ST6Gal1.

Step	Task	Reagents and Operation
1	Transfer	i) Transfer 250 uL sodium cacodylate (1M, pH 6.5) to the reaction vial containing starting material (2 mL water)<br>ii) Transfer CMP-Sia (1.5 eq), ST6Gal1 (1% wt/wt) to the reaction tube
2	Reaction	Vortex the reaction mixture at 300 rpm for 16 h at 37 °C
3	MS Sample	10 uL sample taken from the reaction mixture for ESI MS-analysis
4	SPE1 purification	i) transfer reaction mixture to Ni-NTA cartridge (collect position) at 0.3 mL/min rate<br>ii) transfer water to rinse the reaction tube (2 mL)<br>iii) transfer the rinse solution to Ni-NTA cartridge<br>iv) transfer collected flow-through solution to holding station<br>v) transfer solution from holding station to C18 cartridge (waste position) at 0.3 mL/min rate<br>vi) transfer 4 mL water to C18 cartridge (waste position) for wash<br>vii) transfer 3 mL 40% ACN-water to C18 cartridge (collect position) and collect the released solution
5	Transfer	Transfer collected 40% ACN-water solution containing purified product to evaporation zone
6	Evaporation	Evaporate the acetonitrile from the eluted solution under controlled airflow at 37 °C with vortexing at 300 rpm for 45 min
7	MS Sample	10 uL sample taken from the reaction mixture for ESI MS-analysis, and the MS result showed that the reaction is complete.
8	Transfer	i) transfer 1.8 mL water from the evaporation zone to the next reaction tube<br>ii) transfer water to rinse the evaporation tube (200 uL)<br>iii) transfer rinsed solution to the next reaction tube

Automation program ST6Gal1

Task	Name	Parameter	Description	Estimated Time	Task Number
1	Control Cryostat	Thermostat ON on zone cryo_cooling	Cooling to 4C	00 sec	1
2	Heat / Cool	Thermostat ON on zone electro_heating		00 sec	2
3	Sequence	Execute Once		19 hours 53 min 54 sec	3
1	ST6Gal1 Reaction	Execute Once	Reaction	16 hours 02 min 22 sec	3.1
1	Add Reagents	Execute Once		32 sec	3.1.1
1	Transfer Volumetrically	Transfer liquid from 1M Na-Cacodylate buffer pH 6.5 to Reaction1 with Needle Head #1	Add buffer	32 sec	3.1.1.1
2	Other Reagents	Execute If 'Other_Reagents = 1'	Add other reagents	00 sec	3.1.1.2
1	Transfer Volumetrically	Transfer liquid from CMP_Neu5Ac to Reaction1 with Needle Head #1	transfer sugar nucleotide GDP-fuc solution to reaction_vial	01 min 04 sec	3.1.1.2.1
2	Transfer Volumetrically	Transfer liquid from enzyme_ST6Gal1 to Reaction1 with Needle Head #1	transfer enzyme solution to reaction_vial	01 min 22 sec	3.1.1.2.2
<insert sub tasks here>					
<insert sub tasks here>					
2	Synthesis	Execute Once		16 hours 00 min 02 sec	3.1.2
3	MS Analysis	Execute Once		01 min 47 sec	3.1.3
4	Extend Reaction?	Execute Once		00 sec	3.1.4
1	Show Dialog	Yes/No/Stop-Dialog: 'First round of ST6GalT1 rxn finished. Do you want to add more reagents (Select Yes) or directly go to SPE (select No)?'		00 sec	3.1.4.1
<insert sub tasks here>					
5	Extend Reaction	Execute If 'Extend_Reaction = 1'		00 sec	3.1.5
<insert sub tasks here>					
2	Continue?	Execute Once		02 sec	3.2
3	Purification	Execute Once		03 hours 06 min 26 sec	3.3
1	Stir	Agitation OFF on zone shaker		02 sec	3.3.1
2	Ni-NTA Purification	Execute Once	Ni-NTA Purification	55 min 24 sec	3.3.2
3	C18 Purification	Execute Once	C18 Purification	02 hours 11 min 00 sec	3.3.3
<insert sub tasks here>					
4	Evaporation	Execute Once		45 min 04 sec	3.4
<insert sub tasks here>					
4	Control Cryostat	Thermostat OFF on zone cryo_cooling		00 sec	4
5	Heat / Cool	Thermostat OFF on zone electro_heating		00 sec	5

Table S13. One-pot three enzyme glycosidases.

Step	Task	Reagents and Operation
1	Transfer	i) Transfer 250 uL glycobuffer-1 (1M, pH 5.0) to the reaction vial containing starting material (2 mL water)<br>ii) Transfer neuraminidase, galactosidase, glucosaminindase (1% wt/wt) to the reaction tube
2	Reaction	Vortex the reaction mixture at 300 rpm for 16 h at 37 °C
3	MS Sample	10 uL sample taken from the reaction mixture for ESI MS-analysis
4	SPE1 purification	i) transfer reaction mixture to Ni-NTA cartridge (collect position) at 0.3 mL/min rate<br>ii) transfer water to rinse the reaction tube (2 mL)<br>iii) transfer the rinse solution to Ni-NTA cartridge<br>iv) transfer collected flow-through solution to holding station<br>v) transfer solution from holding station to C18 cartridge (waste position) at 0.3 mL/min rate<br>vi) transfer 4 mL water to C18 cartridge (waste position) for wash<br>vii) transfer 3 mL 40% ACN-water to C18 cartridge (collect position) and collect the released solution
5	Transfer	Transfer collected 40% ACN-water solution containing purified product to evaporation zone
6	Evaporation	Evaporate the acetonitrile from the eluted solution under controlled airflow at 37 °C with vortexing at 300 rpm for 45 min
7	MS Sample	10 uL sample taken from the reaction mixture for ESI MS-analysis, and the MS result showed that the reaction is complete.
8	Transfer	i) transfer 1.8 mL water from the evaporation zone to the next reaction tube<br>ii) transfer water to rinse the evaporation tube (200 uL)<br>iii) transfer rinsed solution to the next reaction tube

Automation program glycosidases

Task	Name	Parameter	Description	Estimated Time	Task Number
1	Control Cryostat	Thermostat ON on zone cryo_cooling	Cooling to 4C	00 sec	1
2	Heat / Cool	Thermostat ON on zone electro_heating		00 sec	2
3	Sequence	Execute Once		15 hours 53 min 55 sec	3
1	SpNaNa-BgaA-StrH Reaction	Execute Once	Reaction	12 hours 02 min 23 sec	3.1
1	Add Reagents	Execute Once		33 sec	3.1.1
1	Transfer Volumetrically	Transfer liquid from GlycoBuffer 1 to Reaction1 with Needle Head #1	Add buffer	33 sec	3.1.1.1
2	Other Reagents	Execute If 'Other_Reagents = 1'	Add other reagents	00 sec	3.1.1.2
1	Transfer Volumetrically	Transfer liquid from Enzyme_SpNaNa to Reaction1 with Needle Head #1	transfer SpNaNa to reaction_vial	01 min 22 sec	3.1.1.2.1
2	Transfer Volumetrically	Transfer liquid from Enzyme_BgaA to Reaction1 with Needle Head #1	transfer BgaA to reaction_vial	01 min 23 sec	3.1.1.2.2
3	Transfer Volumetrically	Transfer liquid from enzyme StrH to Reaction1 with Needle Head #1	transfer StrH to reaction_vial	01 min 34 sec	3.1.1.2.3
<insert sub tasks here>					
<insert sub tasks here>					
2	Synthesis	Execute Once		12 hours 00 min 02 sec	3.1.2
3	MS Analysis	Execute Once		01 min 47 sec	3.1.3
4	Extend Reaction?	Execute Once		00 sec	3.1.4
1	Show Dialog	Yes/No/Stop-Dialog: 'First round of SpNaNa-BgaA-StrH rxn finished. Do you want to add more reagents (Select Yes) or directly go to SPE (select No)?'		00 sec	3.1.4.1
<insert sub tasks here>					
5	Extend Reaction	Execute If 'Extend_Reaction = 1'		00 sec	3.1.5
<insert sub tasks here>					
2	Stir	Agitation ON on zone shaker		02 sec	3.2
3	Continue?	Execute Once		00 sec	3.3
4	Purification	Execute Once		03 hours 06 min 26 sec	3.4
1	Stir	Agitation OFF on zone shaker		02 sec	3.4.1
2	Ni-NTA Purification	Execute Once	Ni-NTA Purification	55 min 24 sec	3.4.2
3	C18 Purification	Execute Once	C18 Purification	02 hours 11 min 00 sec	3.4.3
<insert sub tasks here>					
5	Evaporation	Execute Once		45 min 04 sec	3.5
<insert sub tasks here>					
4	Control Cryostat	Thermostat OFF on zone cryo_cooling		00 sec	4
5	Heat / Cool	Thermostat OFF on zone electro_heating		00 sec	5

Table S14. Removal of TFA group by Na_2CO_3.

Step	Task	Reagents and Operation
1	Transfer	i) Transfer 250 uL sodium carbonate (1M, pH 6.5) to the reaction vial containing starting material (2 mL water)
2	Reaction	Vortex the reaction mixture at 300 rpm for 12 h at 37 °C
3	MS Sample	10 uL sample taken from the reaction mixture for ESI MS-analysis
4	SPE2 purification	i) transfer reaction mixture to C18 cartridge (waste position) at 0.3 mL/min rate<br>ii) transfer 4 mL water to C18 cartridge (waste position) for wash<br>iii) transfer 3 mL 40% ACN-water to C18 cartridge (collect position) and collect the released solution
5	Transfer	Transfer collected 40% ACN-water solution containing purified product to evaporation zone
6	Evaporation	Evaporate the acetonitrile from the eluted solution under controlled airflow at 37 °C with vortexing at 300 rpm for 45 min
7	MS Sample	10 uL sample taken from the reaction mixture for ESI MS-analysis, and the MS result showed that the reaction is complete.
8	Transfer	i) transfer 1.8 mL water from the evaporation zone to the next reaction tube<br>ii) transfer water to rinse the evaporation tube (200 uL)<br>iii) transfer rinsed solution to the next reaction tube

Automation program TFA removal

Task	Name	Parameter	Description	Estimated Time	Task Number
1	Control Cryostat	Thermostat ON on zone cryo_cooling	Cooling to 4C	00 sec	1
2	Heat / Cool	Thermostat ON on zone electro_heating		00 sec	2
3	Sequence	Execute Once		14 hours 58 min 27 sec	3
1	NHTFA to NH2 Reaction	Execute Once	Reaction	12 hours 02 min 21 sec	3.1
1	Add Reagents	Execute Once		32 sec	3.1.1
1	Transfer Volumetrically	Transfer liquid from 1M NaHCO3 to Reaction1 with Needle Head #1	Add Reagent	32 sec	3.1.1.1
<insert sub tasks here>					
2	Synthesis	Execute Once		12 hours 00 min 02 sec	3.1.2
3	MS Analysis	Execute Once		01 min 47 sec	3.1.3
4	Extend Reaction?	Execute Once		00 sec	3.1.4
1	Show Dialog	Yes/No/Stop-Dialog: 'First round of NHTFA to NH2 Reaction finished. Do you want to add more reagents (Select Yes) or directly go to SPE (select No)		00 sec	3.1.4.1
<insert sub tasks here>					
5	Extend Reaction	Execute If 'Extend_Reaction = 1'		00 sec	3.1.5
<insert sub tasks here>					
2	Continue?	Execute Once		02 sec	3.2
3	Purification	Execute Once		02 hours 10 min 59 sec	3.3
1	Stir	Agitation OFF on zone shaker		02 sec	3.3.1
2	C18 Purification	Execute Once	C18 Purification	02 hours 10 min 57 sec	3.3.2
<insert sub tasks here>					
4	Evaporation	Execute Once		45 min 04 sec	3.4
<insert sub tasks here>					
4	Control Cryostat	Thermostat OFF on zone cryo_cooling		00 sec	4
5	Heat / Cool	Thermostat OFF on zone electro_heating		00 sec	5

Table S15. Conversion of amine to azido-functional group by Imidazole-1-sulfonyl azide.

Step	Task	Reagents and Operation
1	Transfer	i) Transfer 200 uL potassium carbonate (1M) to the reaction vial containing starting material (2 mL water)<br>ii) Transfer $CuSO_4$.5 H_2O solution (100mM), Imidazole-1-sulfonyl azide (10mg/mL in water) to the reaction tube
2	Reaction	Vortex the reaction mixture at 300 rpm for 12 h at 37 °C
3	MS Sample	10 uL sample taken from the reaction mixture for ESI MS-analysis
4	SPE2 purification	i) transfer reaction mixture to C18 cartridge (waste position) at 0.3 mL/min rate<br>ii) transfer 4 mL water to C18 cartridge (waste position) for wash<br>iii) transfer 3 mL 40% ACN-water to C18 cartridge (collect position) and collect the released solution
5	Transfer	Transfer collected 40% ACN-water solution containing purified product to evaporation zone
6	Evaporation	Evaporate the acetonitrile from the eluted solution under controlled airflow at 37 °C with vortexing at 300 rpm for 45 min
7	MS Sample	10 uL sample taken from the reaction mixture for ESI MS-analysis, and the MS result showed that the reaction is complete.
8	Transfer	i) transfer 1.8 mL water from the evaporation zone to the next reaction tube<br>ii) transfer water to rinse the evaporation tube (200 uL)<br>iii) transfer rinsed solution to the next reaction tube

Automation program azido installation

Task	Name	Parameter	Description	Estimated Time	Task Number
1	Control Cryostat	Thermostat ON on zone cryo_cooling	Cooling to 4C	00 sec	1
2	Heat / Cool	Thermostat ON on zone electro_heating		00 sec	2
3	Sequence	Execute Once		14 hours 58 min 26 sec	3
1	NH2 to N3 Reaction	Execute Once	Reaction	12 hours 02 min 21 sec	3.1
1	Add Reagents	Execute Once		31 sec	3.1.1
1	Transfer Volumetrically	Transfer liquid from 1M K2CO3 to Reaction1 with Needle Head #1	Add K2CO3 solution to reaction vial	31 sec	3.1.1.1
2	Other Reagents	Execute If 'Other_Reagents = 1'	Add other reagents	00 sec	3.1.1.2
1	Transfer Volumetrically	Transfer liquid from CuSO4 Solution 10mg/mL to Reaction1 with Needle Head #1	transfer CuSO4 solution to reaction_vial	01 min 25 sec	3.1.1.2.1
2	Transfer Volumetrically	Transfer liquid from Imidazole-1-sulfonyl azide solution to Reaction1 with Needle Head #1	transfer Stick's reagent solution to reaction_vial	01 min 21 sec	3.1.1.2.2
<insert sub tasks here>					
<insert sub tasks here>					
2	Synthesis	Execute Once		12 hours 00 min 02 sec	3.1.2
1	Stir	Agitation ON on zone shaker		02 sec	3.1.2.1
2	Wait	Waiting for 12:00:00 hours		12 hours 00 min 00 sec	3.1.2.2
<insert sub tasks here>					
3	MS Analysis	Execute Once		01 min 47 sec	3.1.3
4	Extend Reaction?	Execute Once		00 sec	3.1.4
1	Show Dialog	Yes/No/Stop-Dialog: 'First round of NH2 to N3 Reaction finished. Do you want to add more reagents (Sel		00 sec	3.1.4.1
<insert sub tasks here>					
5	Extend Reaction	Execute If 'Extend_Reaction = 1'		00 sec	3.1.5
<insert sub tasks here>					
2	Continue?	Execute Once		02 sec	3.2
3	Purification	Execute Once		02 hours 10 min 59 sec	3.3
1	Stir	Agitation OFF on zone shaker		02 sec	3.3.1
2	C18 Purification	Execute Once	C18 Purification	02 hours 10 min 57 sec	3.3.2
<insert sub tasks here>					
4	Evaporation	Execute Once		45 min 04 sec	3.4
<insert sub tasks here>					
4	Control Cryostat	Thermostat OFF on zone cryo_cooling		00 sec	4
5	Heat / Cool	Thermostat OFF on zone electro_heating		00 sec	5

Table S16. Conversion of amine to N-acetyl functional group by NHS-acetate.

Step	Task	Reagents and Operation
1	Transfer	i) Transfer 200 uL sodium bicarbonate (1M) to the reaction vial containing starting material (2 mL water)<br>ii) Transfer NHS-acetate solution (10mg/mL) to the reaction tube
2	Reaction	Vortex the reaction mixture at 300 rpm for 12 h at 37 °C
3	MS Sample	10 uL sample taken from the reaction mixture for ESI MS-analysis
4	SPE2 purification	i) transfer reaction mixture to C18 cartridge (waste position) at 0.3 mL/min rate<br>ii) transfer 4 mL water to C18 cartridge (waste position) for wash<br>iii) transfer 3 mL 40% ACN-water to C18 cartridge (collect position) and collect the released solution
5	Transfer	Transfer collected 40% ACN-water solution containing purified product to evaporation zone
6	Evaporation	Evaporate the acetonitrile from the eluted solution under controlled airflow at 37 °C with vortexing at 300 rpm for 45 min
7	MS Sample	10 uL sample taken from the reaction mixture for ESI MS-analysis, and the MS result showed that the reaction is complete.
8	Transfer	i) transfer 1.8 mL water from the evaporation zone to the next reaction tube<br>ii) transfer water to rinse the evaporation tube (200 uL)<br>iii) transfer rinsed solution to the next reaction tube

Automation program acetylation of amine

Task	Name	Parameter	Description	Estimated Time	Task Number
1	Control Cryostat	Thermostat ON on zone cryo_cooling	Cooling to 4C	00 sec	1
2	Heat / Cool	Thermostat ON on zone electro_heating		00 sec	2
3	Sequence	Execute Once		14 hours 58 min 28 sec	3
1	NH2 to NHAc Reaction	Execute Once	Reaction	12 hours 02 min 21 sec	3.1
1	Add Reagents	Execute Once		32 sec	3.1.1
1	Transfer Volumetrically	Transfer liquid from 1M NaHCO3 to Reaction1 with Needle Head #1	Add NaHCO3 solution to reaction vial	32 sec	3.1.1.1
2	Other Reagents	Execute If 'Other_Reagents = 1'	Add other reagents	00 sec	3.1.1.2
1	Transfer Volumetrically	Transfer liquid from NHS-Acetate solution to Reaction1 with Needle Head #1	transfer NHS-Acetate solution to reaction_vial	01 min 23 sec	3.1.1.2.1
<insert sub tasks here>					
<insert sub tasks here>					
2	Synthesis	Execute Once		12 hours 00 min 02 sec	3.1.2
3	MS Analysis	Execute Once		01 min 47 sec	3.1.3
4	Extend Reaction?	Execute Once		00 sec	3.1.4
1	Show Dialog	Yes/No/Stop-Dialog: 'First round of NH2 to NHAc Reaction finished. Do you want to add more reagents (Select Yes) or directly go to SPE (select No)?		00 sec	3.1.4.1
<insert sub tasks here>					
5	Extend Reaction	Execute If 'Extend_Reaction = 1'		00 sec	3.1.5
<insert sub tasks here>					
2	Continue?	Execute Once		02 sec	3.2
3	Purification	Execute Once		02 hours 11 min 00 sec	3.3
1	Stir	Agitation OFF on zone shaker		02 sec	3.3.1
2	C18 Purification	Execute Once	C18 Purification	02 hours 10 min 58 sec	3.3.2
<insert sub tasks here>					
4	Evaporation	Execute Once		45 min 04 sec	3.4
1	Stir	Agitation ON on zone shaker		02 sec	3.4.1
2	Wait	Waiting for 45:00 minutes		45 min 00 sec	3.4.2
3	Stir	Agitation OFF on zone shaker		02 sec	3.4.3
<insert sub tasks here>					
<insert sub tasks here>					
4	Control Cryostat	Thermostat OFF on zone cryo_cooling		00 sec	4
5	Heat / Cool	Thermostat OFF on zone electro_heating		00 sec	5

Table S17. Deprotection of Boc- group by trifluoroacetic acid.

Step	Task	Reagents and Operation
1	Transfer	i) Transfer 2% TFA solution to the reaction tube containing starting material (in 2 mL water)
2	Reaction	Vortex the reaction mixture at 300 rpm for 3 h at 37 °C
3	MS Sample	10 uL sample taken from the reaction mixture for ESI MS-analysis
4	SPE2 purification	i) transfer reaction mixture to C18 cartridge (waste position) at 0.3 mL/min rate<br>ii) transfer 4 mL water to C18 cartridge (waste position) for wash<br>iii) transfer 3 mL 40% ACN-water to C18 cartridge (collect position) and collect the released solution
5	Transfer	Transfer collected 40% ACN-water solution containing purified product to evaporation zone
6	Evaporation	Evaporate the acetonitrile from the eluted solution under controlled airflow at 37 °C with vortexing at 300 rpm for 45 min
7	MS Sample	10 uL sample taken from the reaction mixture for ESI MS-analysis, and the MS result showed that the reaction is complete.
8	Transfer	i) transfer 1.8 mL water from the evaporation zone to the next reaction tube<br>ii) transfer water to rinse the evaporation tube (200 uL)<br>iii) transfer rinsed solution to the next reaction tube

Automation program Boc- deprotection

Task	Name	Parameter	Description	Estimated Time	Task Number
1	Control Cryostat	Thermostat ON on zone cryo_cooling	Cooling to 4C	00 sec	1
2	Heat / Cool	Thermostat ON on zone electro_heating		00 sec	2
3	Sequence	Execute Once		05 hours 58 min 29 sec	3
1	NHBoc to NH2 Reactic	Execute Once	Reaction	03 hours 02 min 24 sec	3.1
1	Add Reagents	Execute Once		34 sec	3.1.1
1	Transfer Volumetrically	Transfer liquid from 2% TFA solution to Reaction1 with Needle Head #1	Add TFA solution	34 sec	3.1.1.1
<insert sub tasks here>					
2	Synthesis	Execute Once		03 hours 00 min 02 sec	3.1.2
1	Stir	Agitation ON on zone shaker		02 sec	3.1.2.1
2	Wait	Waiting for 3:00:00 hours		03 hours 00 min 00 sec	3.1.2.2
<insert sub tasks here>					
3	MS Analysis	Execute Once		01 min 47 sec	3.1.3
4	Extend Reaction?	Execute Once		00 sec	3.1.4
1	Show Dialog	Yes/No/Stop-Dialog: 'First round of NHBoc to NH2 Reaction finished. Do you want to add more reagents (Select Yes) or directly go to SPE (select No)?'		00 sec	3.1.4.1
<insert sub tasks here>					
5	Extend Reaction	Execute If 'Extend_Reaction = 1'		00 sec	3.1.5
<insert sub tasks here>					
2	Continue?	Execute Once		02 sec	3.2
3	Purification	Execute Once		02 hours 10 min 59 sec	3.3
1	Stir	Agitation OFF on zone shaker		02 sec	3.3.1
2	C18 Purification	Execute Once	C18 Purification	02 hours 10 min 57 sec	3.3.2
<insert sub tasks here>					
4	Evaporation	Execute Once		45 min 04 sec	3.4
<insert sub tasks here>					
4	Control Cryostat	Thermostat OFF on zone cryo_cooling		00 sec	4
5	Heat / Cool	Thermostat OFF on zone electro_heating		00 sec	5

Table S18. Protection of amine with di-tert-butyl decarbonate.

Step	Task	Reagents and Operation
1	Transfer	i) Transfer 200 uL sodium carbonate (1M) to the reaction vial containing starting material (2 mL water)<br>ii) Transfer Boc-anhydride solution (3 eq) (10mg/mL in 1,4-dioxane) to the reaction tube
2	Reaction	Vortex the reaction mixture at 300 rpm for 12 h at 37 °C
3	MS Sample	10 uL sample taken from the reaction mixture for ESI MS-analysis
4	SPE3 purification	i) transfer reaction mixture to evaporation zone at 0.3 mL/min rate<br>ii) Evaporate the volatile organic component from the reaction mixture (Boc_2O, Dioxane) under controlled airflow at 45 °C with vortexing at 300 rpm for 1 h<br>iii) transfer solution to C18 cartridge (waste position) from evaporation zone at 0.3 mL/min rate<br>iv) transfer 4 mL water to C18 cartridge (waste position) for wash<br>v) transfer 3 mL 40% ACN-water to C18 cartridge (collect position) and collect the released solution
5	Transfer	Transfer collected 40% ACN-water solution containing purified product to evaporation zone
6	Evaporation	Evaporate the acetonitrile from the eluted solution under controlled airflow at 37 °C with vortexing at 300 rpm for 45 min
7	MS Sample	10 uL sample taken from the reaction mixture for ESI MS-analysis, and the MS result showed that the reaction is complete.
8	Transfer	i) transfer 1.8 mL water from the evaporation zone to the next reaction tube<br>ii) transfer water to rinse the evaporation tube (200 uL)<br>iii) transfer rinsed solution to the next reaction tube

Automation program Boc- installation

Task	Name	Parameter	Description	Estimated Time	Task Nu
1	Control Cryostat	Thermostat ON on zone cryo_cooling	Cooling to 4C	00 sec	1
2	Heat / Cool	Thermostat ON on zone electro_heating		00 sec	2
3	Sequence	Execute Once		15 hours 55 min 04 sec	3
1	NH2 to NHBoc Reaction	Execute Once	Reaction	12 hours 02 min 20 sec	3.1
1	Add Reagents	Execute Once		31 sec	3.1.1
1	Transfer Volumetrically	Transfer liquid from 1M Na2CO3 to Reaction1 with Needle Head #1	Add Na2CO3 solution to reaction vial	31 sec	3.1.1.1
2	Other Reagents	Execute If 'Other_Reagents = 1'	Add other reagents	00 sec	3.1.1.2
1	Transfer Volumetrically	Transfer liquid from Boc-anhydride solution in Dioxane to Reaction1 with Needle Head #1	transfer Boc-anhydride solution to reaction_vial	01 min 25 sec	3.1.1.2.1
<insert sub tasks here>					
<insert sub tasks here>					
2	Synthesis	Execute Once		12 hours 00 min 02 sec	3.1.2
1	Stir	Agitation ON on zone shaker		02 sec	3.1.2.1
2	Wait	Waiting for 12:00:00 hours		12 hours 00 min 00 sec	3.1.2.2
<insert sub tasks here>					
3	MS Analysis	Execute Once		01 min 47 sec	3.1.3
4	Extend Reaction?	Execute Once		00 sec	3.1.4
1	Show Dialog	Yes/No/Stop-Dialog: 'First round of NH2 to NHAc Reaction finished. Do you want to add more reagents (Select Yes) or directly go to Evaporation (select No)?'		00 sec	3.1.4.1
<insert sub tasks here>					
5	Extend Reaction	Execute If 'Extend_Reaction = 1'		00 sec	3.1.5
<insert sub tasks here>					
2	Continue?	Execute Once		02 sec	3.2
3	Transfer to evaporation	Execute Once		56 min 32 sec	3.3
4	Purification	Execute Once		02 hours 11 min 05 sec	3.4
1	Stir	Agitation OFF on zone shaker		02 sec	3.4.1
2	C18 Purification	Execute Once	C18 Purification	02 hours 11 min 03 sec	3.4.2
<insert sub tasks here>					
5	Evaporation	Execute Once		45 min 04 sec	3.5
<insert sub tasks here>					
4	Control Cryostat	Thermostat OFF on zone cryo_cooling		00 sec	4
5	Heat / Cool	Thermostat OFF on zone electro_heating		00 sec	5

Table S19. Reduction of azide with trimethylphosphene.

Step	Task	Reagents and Operation
1	Transfer	i) Transfer 200 uL sodium hydroxide (1M) to the reaction vial containing starting material (2 mL water)<br>ii) Transfer PMe_3 solution (15 eq) (1 M in THF) to the reaction tube
2	Reaction	Vortex the reaction mixture at 300 rpm for 12 h at 37 °C
3	MS Sample	10 uL sample taken from the reaction mixture for ESI MS-analysis
4	SPE3 purification	i) transfer reaction mixture to evaporation zone at 0.3 mL/min rate<br>ii) Evaporate the volatile organic component from the reaction mixture (PMe_3, THF) under controlled airflow at 45 °C with vortexing at 300 rpm for 1 h<br>iii) transfer solution to C18 cartridge (waste position) from evaporation zone at 0.3 mL/min rate<br>iv) transfer 4 mL water to C18 cartridge (waste position) for wash<br>v) transfer 3 mL 40% ACN-water to C18 cartridge (collect position) and collect the released solution
5	Transfer	Transfer collected 40% ACN-water solution containing purified product to evaporation zone
6	Evaporation	Evaporate the acetonitrile from the eluted solution under controlled airflow at 37 °C with vortexing at 300 rpm for 45 min
7	MS Sample	10 uL sample taken from the reaction mixture for ESI MS-analysis, and the MS result showed that the reaction is complete.
8	Transfer	i) transfer 1.8 mL water from the evaporation zone to the next reaction tube<br>ii) transfer water to rinse the evaporation tube (200 uL)<br>iii) transfer rinsed solution to the next reaction tube

Automation program azide reduction

Task	Name	Parameter	Description	Estimated Time	Task Number
1	Control Cryostat	Thermostat ON on zone cryo_cooling	Cooling to 4C	00 sec	1
2	Heat / Cool	Thermostat ON on zone electro_heating		00 sec	2
3	Sequence	Execute Once		16 hours 06 min 32 sec	3
1	N3 Reduction Reaction	Execute Once	Reaction	12 hours 02 min 22 sec	3.1
1	Add Reagents	Execute Once		32 sec	3.1.1
1	Transfer Volumetrically	Transfer liquid from 1M NaOH to Reaction1 with Needle Head #1	Add NaOH solution	32 sec	3.1.1.1
2	Other Reagents	Execute If 'Other_Reagents = 1'	Add other reagents	00 sec	3.1.1.2
1	Transfer Volumetrically	Transfer liquid from 1M PMe3 in THF to Reaction1 with Needle Head #1	transfer PMe3 solution to reaction_vial	01 min 08 sec	3.1.1.2.1
<insert sub tasks here>					
<insert sub tasks here>					
2	Synthesis	Execute Once		12 hours 00 min 02 sec	3.1.2
3	MS Analysis	Execute Once		01 min 47 sec	3.1.3
4	Extend Reaction?	Execute Once		00 sec	3.1.4
1	Show Dialog	Yes/No/Stop-Dialog: 'N3 reduction rxn finished. Do you want to add more reagents (Select Yes) or directly		00 sec	3.1.4.1
<insert sub tasks here>					
5	Extend Reaction	Execute If 'Extend_Reaction = 1'		00 sec	3.1.5
<insert sub tasks here>					
2	Transfer to evaporation	Execute Once		56 min 32 sec	3.2
3	Purification	Execute Once		02 hours 11 min 05 sec	3.3
1	Stir	Agitation OFF on zone shaker		02 sec	3.3.1
2	C18 Purification	Execute Once	C18 Purification	02 hours 11 min 03 sec	3.3.2
<insert sub tasks here>					
4	Evaporation	Execute Once		45 min 04 sec	3.4
5	Transfer to reaction 1	Execute Once		11 min 28 sec	3.5
<insert sub tasks here>					
4	Control Cryostat	Thermostat OFF on zone cryo_cooling		00 sec	4
5	Heat / Cool	Thermostat OFF on zone electro_heating		00 sec	5

Table S20. Sequential B3GnT2-B4GalT1 consecutive reaction cycles.

Step	Task	Reagents and Operation
1	Transfer	i) Transfer 250 uL HEPES buffer (1M, pH 7) to the reaction vial containing starting material (2 mL water)<br>ii) Transfer UDP-GlcNAc (1.5 eq), 25 uL $MgCl_2$ (1 M), 25 uL DTT (100 mM), 10 uL KCl (1 M), B3GNT2 (1% wt/wt) to the reaction tube
2	Reaction	Vortex the reaction mixture at 300 rpm for 16 h at 37 °C
3	MS Sample	10 uL sample taken from the reaction mixture for ESI MS-analysis
4	SPE1 purification	i) transfer reaction mixture to Ni-NTA cartridge (collect position) at 0.3 mL/min rate<br>ii) transfer water to rinse the reaction tube (2 mL)<br>iii) transfer the rinse solution to Ni-NTA cartridge<br>iv) transfer collected flow-through solution to holding station<br>v) transfer solution from holding station to C18 cartridge (waste position) at 0.3 mL/min rate<br>vi) transfer 4 mL water to C18 cartridge (waste position) for wash<br>vii) transfer 3 mL 40% ACN-water to C18 cartridge (collect position) and collect the released solution
5	Transfer	Transfer collected 40% ACN-water solution containing purified product to evaporation zone
6	Evaporation	Evaporate the acetonitrile from the eluted solution under controlled airflow at 37 °C with vortexing at 300 rpm for 45 min
7	MS Sample	10 uL sample taken from the reaction mixture for ESI MS-analysis, and the MS result showed that the reaction is complete.
8	Transfer	i) transfer 1.8 mL water from the evaporation zone to the next reaction tube<br>ii) transfer water to rinse the evaporation tube (200 uL)<br>iii) transfer rinsed solution to the next reaction tube for subsequent galactosylation

9	Transfer	i) Transfer 250 uL Tris-HCL buffer (1M, pH 7.5) to the reaction vial containing starting material (2 mL water)<br>ii) Transfer UDP-Gal (1.5 eq), 25 uL $MnCl_2$ (1M), B4GalT4 (1% wt/wt) to the reaction tube
10	Reaction	Vortex the reaction mixture at 300 rpm for 12 h at 37 °C
11	MS Sample	10 uL sample taken from the reaction mixture for ESI MS-analysis.
12	SPE1 purification	i) transfer reaction mixture to Ni-NTA cartridge (collect position) at 0.3 mL/min rate<br>ii) transfer water to rinse the reaction tube (2 mL)<br>iii) transfer the rinse solution to Ni-NTA cartridge<br>iv) transfer collected flow-through solution to holding station<br>v) transfer solution from holding station to C18 cartridge (waste position) at 0.3 mL/min rate<br>vi) transfer 4 mL water to C18 cartridge (waste position) for wash<br>vii) transfer 3 mL 40% ACN-water to C18 cartridge (collect position) and collect the released solution
13	Transfer	Transfer collected 40% ACN-water solution containing purified product to evaporation zone
14	Evaporation	Evaporate the acetonitrile from the eluted solution under controlled airflow at 37 °C with vortexing at 300 rpm for 45 min
15	MS Sample	10 uL sample taken from the reaction mixture for ESI MS-analysis, and the MS result showed that the reaction is complete.
16	Transfer	i) transfer 1.8 mL water from the evaporation zone to the next reaction tube<br>ii) transfer water to rinse the evaporation tube (200 uL)<br>iii) transfer rinsed solution to the next reaction tube

Automation program

Task	Name	Parameter	Description	Estimated Time	Task Number
1	Control Cryostat	Thermostat ON on zone cryo_cooling	Cooling to 4C	00 sec	1
2	Heat / Cool	Thermostat ON on zone electro_heating		00 sec	2
3	Sequence B3GNT2 -> B4GalT1	Execute Once		32 hours 07 min 50 sec	3
1	B3GNT2 Reaction	Execute Once	Reaction	12 hours 02 min 22 sec	3.1
1	Add Reagents	Execute Once		32 sec	3.1.1
1	Transfer Volumetrically	Transfer liquid from 1M HEPES pH 7.5 to Reaction1 with Needle Head #1	Add 250 uL bufffer	32 sec	3.1.1.1
2	Other Reagents	Execute If 'Other_Reagents = 1'	Add other reagents	00 sec	3.1.1.2
1	Transfer Volumetrically	Transfer liquid from UDP-GlcNAc to Reaction1 with Needle Head #1	transfer sugar nucleotide solution to reaction_	01 min 04 sec	3.1.1.2.1
2	Transfer Volumetrically	Transfer liquid from Mg_solution to Reaction1 with Needle Head #1	transfer Mg solution to reaction_vial	01 min 01 sec	3.1.1.2.2
3	Transfer Volumetrically	Transfer liquid from KCl_solution to Reaction1 with Needle Head #1	transfer KCl solution to reaction_vial	01 min 01 sec	3.1.1.2.3
4	Transfer Volumetrically	Transfer liquid from DTT_solution to Reaction1 with Needle Head #1	transfer DTT solution to reaction_vial	01 min 01 sec	3.1.1.2.4
5	Transfer Volumetrically	Transfer liquid from enzyme_B3GNT2 to Reaction1 with Needle Head #1	transfer enzyme solution to reaction_vial	01 min 21 sec	3.1.1.2.5
<insert sub tasks here>					
<insert sub tasks here>					
2	Synthesis	Execute Once		12 hours 00 min 02 sec	3.1.2
1	Stir	Agitation ON on zone shaker		02 sec	3.1.2.1
2	Wait	Waiting for 12:00:00 hours		12 hours 00 min 00 sec	3.1.2.2
<insert sub tasks here>					
3	MS Analysis	Execute Once		01 min 47 sec	3.1.3
4	Extend Reaction?	Execute Once		00 sec	3.1.4
1	Show Dialog	Yes/No/Stop-Dialog: 'First round of B3GNT2 finished. Do you want to add more reagent		00 sec	3.1.4.1
<insert sub tasks here>					
5	Extend Reaction	Execute If 'Extend_Reaction = 1'		00 sec	3.1.5
<insert sub tasks here>					
2	Continue?	Execute Once		02 sec	3.2
3	Purification	Execute Once		03 hours 06 min 26 sec	3.3
1	Stir	Agitation OFF on zone shaker		02 sec	3.3.1
2	Ni-NTA Purification	Execute Once	Ni-NTA Purification	55 min 24 sec	3.3.2
3	C18 Purification	Execute Once		02 hours 11 min 00 sec	3.3.3

Task	Name	Parameter	Description	Estimated Time	Task Number
<insert sub tasks here>					
4	Evaporation	Execute Once		45 min 04 sec	3.4
5	Transfer to Reaction 2	Execute Once		19 min 58 sec	3.5
6	Sequence B4GalT1	Execute Once		15 hours 53 min 56 sec	3.6
1	B4GalT1 Reaction	Execute Once	Reaction	12 hours 02 min 23 sec	3.6.1
1	Add Reagents	Execute Once		34 sec	3.6.1.1
1	Transfer Volumetrically	Transfer liquid from 1M Tris pH 7.5 to Reaction2 with Needle Head #1	Add 300 uL bufffer	34 sec	3.6.1.1.1
2	Other Reagents	Execute If 'Other_Reagents = 1'	Add other reagents	00 sec	3.6.1.1.2
1	Transfer Volumetrically	Transfer liquid from variable 'Sugar_donor' to Reaction2 with Needle Head #1	transfer sugar nucleotide solution to reaction_	01 min 04 sec	3.6.1.1.2.1
2	Transfer Volumetrically	Transfer liquid from variable 'metal_cation' to Reaction2 with Needle Head #1	transfer ALP solution to reaction_vial	01 min 01 sec	3.6.1.1.2.2
3	Transfer Volumetrically	Transfer liquid from variable 'enzyme_solution' to Reaction2 with Needle Head #1	transfer enzyme solution to reaction_vial	01 min 23 sec	3.6.1.1.2.3
<insert sub tasks here>					
<insert sub tasks here>					
2	Synthesis	Execute Once		12 hours 00 min 02 sec	3.6.1.2
3	MS Analysis	Execute Once		01 min 47 sec	3.6.1.3
4	Extend Reaction?	Execute Once		00 sec	3.6.1.4
5	Extend Reaction	Execute If 'Extend_Reaction = 1'		00 sec	3.6.1.5
<insert sub tasks here>					
2	Continue?	Execute Once		02 sec	3.6.2
3	Purification	Execute Once		03 hours 06 min 27 sec	3.6.3
1	Stir	Agitation OFF on zone shaker		02 sec	3.6.3.1
2	Ni-NTA Purification	Execute Once	Ni-NTA Purification	55 min 24 sec	3.6.3.2
3	C18 Purification	Execute Once		02 hours 11 min 00 sec	3.6.3.3
<insert sub tasks here>					
4	Evaporation	Execute Once		45 min 04 sec	3.6.4
<insert sub tasks here>					

Table S21. Sequential β1,2-GlcNAc(GnT-I)-β1,2-GlcNHTFA(GnT-II) consecutive reaction cycles.

Step	Task	Reagents and Operation
1	Transfer	i) Transfer 250 uL MES buffer (1M, pH 6.5) to the reaction vial containing starting material (2 mL water)<br>ii) Transfer UDP-GlcNAc (1.5 eq), 25 uL $MnCl_2$ (1M), GnT-I (1% wt/wt) to the reaction tube
2	Reaction	Vortex the reaction mixture at 300 rpm for 16 h at 37 °C
3	MS Sample	10 uL sample taken from the reaction mixture for ESI MS-analysis
4	SPE1 purification	i) transfer reaction mixture to Ni-NTA cartridge (collect position) at 0.3 mL/min rate<br>ii) transfer water to rinse the reaction tube (2 mL)<br>iii) transfer the rinse solution to Ni-NTA cartridge<br>iv) transfer collected flow-through solution to holding station<br>v) transfer solution from holding station to C18 cartridge (waste position) at 0.3 mL/min rate<br>vi) transfer 4 mL water to C18 cartridge (waste position) for wash<br>vii) transfer 3 mL 40% ACN-water to C18 cartridge (collect position) and collect the released solution
5	Transfer	Transfer collected 40% ACN-water solution containing purified product to evaporation zone
6	Evaporation	Evaporate the acetonitrile from the eluted solution under controlled airflow at 37 °C with vortexing at 300 rpm for 45 min
7	MS Sample	10 uL sample taken from the reaction mixture for ESI MS-analysis, and the MS result showed that the reaction is complete.
8	Transfer	i) transfer 1.8 mL water from the evaporation zone to the next reaction tube<br>ii) transfer water to rinse the evaporation tube (200 uL)<br>iii) transfer rinsed solution to the next reaction tube for subsequent galactosylation

9	Transfer	i) Transfer 250 uL MES buffer (1M, pH 6.5) to the reaction vial containing starting material (2 mL water)<br>ii) Transfer UDP-GlcNHTFA (1.5 eq), 25 uL $MnCl_2$ (1M), GnT-II (1% wt/wt) to the reaction tube
10	Reaction	Vortex the reaction mixture at 300 rpm for 12 h at 37 °C
11	MS Sample	10 uL sample taken from the reaction mixture for ESI MS-analysis.
12	SPE1 purification	i) transfer reaction mixture to Ni-NTA cartridge (collect position) at 0.3 mL/min rate<br>ii) transfer water to rinse the reaction tube (2 mL)<br>iii) transfer the rinse solution to Ni-NTA cartridge<br>iv) transfer collected flow-through solution to holding station<br>v) transfer solution from holding station to C18 cartridge (waste position) at 0.3 mL/min rate<br>vi) transfer 4 mL water to C18 cartridge (waste position) for wash<br>vii) transfer 3 mL 40% ACN-water to C18 cartridge (collect position) and collect the released solution
13	Transfer	Transfer collected 40% ACN-water solution containing purified product to evaporation zone
14	Evaporation	Evaporate the acetonitrile from the eluted solution under controlled airflow at 37 °C with vortexing at 300 rpm for 45 min
15	MS Sample	10 uL sample taken from the reaction mixture for ESI MS-analysis, and the MS result showed that the reaction is complete.
16	Transfer	i) transfer 1.8 mL water from the evaporation zone to the next reaction tube<br>ii) transfer water to rinse the evaporation tube (200 uL)<br>iii) transfer rinsed solution to the next reaction tube

Automation program

Task	Name	Parameter	Description	Estimated Time	Task Number
1	Control Cryostat	Thermostat ON on zone cryo_cooling	Cooling to 4C	00 sec	1
2	Heat / Cool	Thermostat ON on zone electro_heating		00 sec	2
3	Sequence	Execute Once		40 hours 04 min 13 sec	3
1	MGAT1 Reaction	Execute Once	Reaction	16 hours 02 min 22 sec	3.1
1	Add Reagents	Execute Once		33 sec	3.1.1
1	Transfer Volumetrically	Transfer liquid from MES Buffer pH 6.5 to Reaction1 with Needle Head #1	Add bufffer	33 sec	3.1.1.1
2	Other Reagents	Execute If 'Other_Reagents = 1'	Add other reagents	00 sec	3.1.1.2
1	Transfer Volumetrically	Transfer liquid from UDP-GlcNAc to Reaction1 with Needle Head #1	transfer sugar nucleotide solution to reaction_vial	01 min 04 sec	3.1.1.2.1
2	Transfer Volumetrically	Transfer liquid from Mn_solution to Reaction1 with Needle Head #1	transfer Mn solution to reaction_vial	01 min 01 sec	3.1.1.2.2
3	Transfer Volumetrically	Transfer liquid from Enzyme MGAT1 to Reaction1 with Needle Head #1	transfer enzyme solution to reaction_vial	01 min 21 sec	3.1.1.2.3
<insert sub tasks here>					
<insert sub tasks here>					
2	Synthesis	Execute Once		16 hours 00 min 02 sec	3.1.2
1	Stir	Agitation ON on zone shaker		02 sec	3.1.2.1
2	Wait	Waiting for 16:00:00 hours		16 hours 00 min 00 sec	3.1.2.2
<insert sub tasks here>					
3	MS Analysis	Execute Once		01 min 47 sec	3.1.3
4	Extend Reaction?	Execute Once		00 sec	3.1.4
1	Show Dialog	Yes/No/Stop-Dialog: 'First round of MGAT1 reaction finished. Do you want to add more		00 sec	3.1.4.1
<insert sub tasks here>					
5	Extend Reaction	Execute If 'Extend_Reaction = 1'		00 sec	3.1.5
<insert sub tasks here>					
2	Continue?	Execute Once		02 sec	3.2
3	Purification	Execute Once		03 hours 06 min 26 sec	3.3
4	Evaporation	Execute Once		45 min 04 sec	3.4
5	Transfer to Reaction 2	Execute Once		18 min 08 sec	3.5

Task	Name	Parameter	Description	Estimated Time	Task Number
<insert sub tasks here>					
6	Sequence	Execute Once		19 hours 52 min 09 sec	3.6
1	MGAT2 Reaction	Execute Once	Reaction	16 hours 02 min 23 sec	3.6.1
1	Add Reagents	Execute Once		33 sec	3.6.1.1
1	Transfer Volumetrically	Transfer liquid from MES Buffer pH 6.5 to Reaction2 with Needle Head #1	Add 300 uL bufffer	33 sec	3.6.1.1.1
2	Other Reagents	Execute If 'Other_Reagents = 1'	Add other reagents	00 sec	3.6.1.1.2
1	Transfer Volumetrically	Transfer liquid from UDP-GlcNHTFA to Reaction2 with Needle Head #1	transfer sugar nucleotide solution to reaction_vial	01 min 04 sec	3.6.1.1.2.1
2	Transfer Volumetrically	Transfer liquid from Mn_solution to Reaction2 with Needle Head #1	transfer Mn solution to reaction_vial	01 min 01 sec	3.6.1.1.2.2
3	Transfer Volumetrically	Transfer liquid from Enzyme MGAT2 to Reaction2 with Needle Head #1	transfer enzyme solution to reaction_vial	01 min 22 sec	3.6.1.1.2.3
<insert sub tasks here>					
<insert sub tasks here>					
2	Synthesis	Execute Once		16 hours 00 min 02 sec	3.6.1.2
3	MS Analysis	Execute Once		01 min 47 sec	3.6.1.3
4	Extend Reaction?	Execute Once		00 sec	3.6.1.4
1	Show Dialog	Yes/No/Stop-Dialog: 'First round of MGAT2 rxn finished. Do you want to add more reag		00 sec	3.6.1.4.1
<insert sub tasks here>					
5	Extend Reaction	Execute If 'Extend_Reaction = 1'		00 sec	3.6.1.5
<insert sub tasks here>					
2	Continue?	Execute Once		02 sec	3.6.2
3	Purification	Execute Once		03 hours 04 min 40 sec	3.6.3
1	Stir	Agitation OFF on zone shaker		02 sec	3.6.3.1
2	Ni-NTA Purification	Execute Once	Ni-NTA Purification	55 min 24 sec	3.6.3.2
3	C18 Purification	Execute Once		02 hours 09 min 14 sec	3.6.3.3
<insert sub tasks here>					
4	Evaporation	Execute Once		45 min 04 sec	3.6.4
<insert sub tasks here>					

Table S22. Sequential β1,2-GlcNHTFA(GnT-I)-β1,2-GlcNAc(GnT-II) consecutive reaction cycles.

Step	Task	Reagents and Operation
1	Transfer	i) Transfer 250 uL MES buffer (1M, pH 6.5) to the reaction vial containing starting material (2 mL water)<br>ii) Transfer UDP-GlcNHTFA (1.5 eq), 25 uL $MnCl_2$ (1M), GnT-I (1% wt/wt) to the reaction tube
2	Reaction	Vortex the reaction mixture at 300 rpm for 16 h at 37 °C
3	MS Sample	10 uL sample taken from the reaction mixture for ESI MS-analysis
4	SPE1 purification	i) transfer reaction mixture to Ni-NTA cartridge (collect position) at 0.3 mL/min rate<br>ii) transfer water to rinse the reaction tube (2 mL)<br>iii) transfer the rinse solution to Ni-NTA cartridge<br>iv) transfer collected flow-through solution to holding station<br>v) transfer solution from holding station to C18 cartridge (waste position) at 0.3 mL/min rate<br>vi) transfer 4 mL water to C18 cartridge (waste position) for wash<br>vii) transfer 3 mL 40% ACN-water to C18 cartridge (collect position) and collect the released solution
5	Transfer	Transfer collected 40% ACN-water solution containing purified product to evaporation zone
6	Evaporation	Evaporate the acetonitrile from the eluted solution under controlled airflow at 37 °C with vortexing at 300 rpm for 45 min
7	MS Sample	10 uL sample taken from the reaction mixture for ESI MS-analysis, and the MS result showed that the reaction is complete.
8	Transfer	i) transfer 1.8 mL water from the evaporation zone to the next reaction tube<br>ii) transfer water to rinse the evaporation tube (200 uL)<br>iii) transfer rinsed solution to the next reaction tube for subsequent galactosylation

9	Transfer	i) Transfer 250 uL MES buffer (1M, pH 6.5) to the reaction vial containing starting material (2 mL water)<br>ii) Transfer UDP-GlcNAc (1.5 eq), 25 uL $MnCl_2$ (1M), GnT-II (1% wt/wt) to the reaction tube
10	Reaction	Vortex the reaction mixture at 300 rpm for 12 h at 37 °C
11	MS Sample	10 uL sample taken from the reaction mixture for ESI MS-analysis.
12	SPE1 purification	i) transfer reaction mixture to Ni-NTA cartridge (collect position) at 0.3 mL/min rate<br>ii) transfer water to rinse the reaction tube (2 mL)<br>iii) transfer the rinse solution to Ni-NTA cartridge<br>iv) transfer collected flow-through solution to holding station<br>v) transfer solution from holding station to C18 cartridge (waste position) at 0.3 mL/min rate<br>vi) transfer 4 mL water to C18 cartridge (waste position) for wash<br>vii) transfer 3 mL 40% ACN-water to C18 cartridge (collect position) and collect the released solution
13	Transfer	Transfer collected 40% ACN-water solution containing purified product to evaporation zone
14	Evaporation	Evaporate the acetonitrile from the eluted solution under controlled airflow at 37 °C with vortexing at 300 rpm for 45 min
15	MS Sample	10 uL sample taken from the reaction mixture for ESI MS-analysis, and the MS result showed that the reaction is complete.
16	Transfer	i) transfer 1.8 mL water from the evaporation zone to the next reaction tube<br>ii) transfer water to rinse the evaporation tube (200 uL)<br>iii) transfer rinsed solution to the next reaction tube

Automation program

Task	Name	Parameter	Description	Estimated Time	Task Number
1	Control Cryostat	Thermostat ON on zone cryo_cooling	Cooling to 4C	00 sec	1
2	Heat / Cool	Thermostat ON on zone electro_heating		00 sec	2
3	Sequence	Execute Once		40 hours 24 min 07 sec	3
1	MGAT1 Reaction	Execute Once	Reaction	16 hours 02 min 22 sec	3.1
1	Add Reagents	Execute Once		33 sec	3.1.1
1	Transfer Volumetrically	Transfer liquid from MES Buffer pH 6.5 to Reaction1 with Needle Head #1	Add 250 uL bufffer	33 sec	3.1.1.1
2	Other Reagents	Execute If 'Other_Reagents = 1'	Add other reagents	00 sec	3.1.1.2
1	Transfer Volumetrically	Transfer liquid from UDP-GlcNHTFA to Reaction1 with Needle Head #1	transfer sugar nucleotide solution to reaction_	01 min 04 sec	3.1.1.2.1
2	Transfer Volumetrically	Transfer liquid from Mn_solution to Reaction1 with Needle Head #1	transfer Mn solution to reaction_vial	01 min 01 sec	3.1.1.2.2
3	Transfer Volumetrically	Transfer liquid from Enzyme MGAT1 to Reaction1 with Needle Head #1	transfer enzyme solution to reaction_vial	01 min 21 sec	3.1.1.2.3
<insert sub tasks here>					
<insert sub tasks here>					
2	Synthesis	Execute Once		16 hours 00 min 02 sec	3.1.2
3	MS Analysis	Execute Once		01 min 47 sec	3.1.3
4	Extend Reaction?	Execute Once		00 sec	3.1.4
1	Show Dialog	Yes/No/Stop-Dialog: 'First round of MGAT1 rxn finished. Do you want to add more reage		00 sec	3.1.4.1
<insert sub tasks here>					
5	Extend Reaction	Execute If 'Extend_Reaction = 1'		00 sec	3.1.5
<insert sub tasks here>					
2	Continue?	Execute Once		02 sec	3.2
3	Purification	Execute Once		03 hours 06 min 26 sec	3.3
1	Stir	Agitation OFF on zone shaker		02 sec	3.3.1
2	Ni-NTA Purification	Execute Once	Ni-NTA Purification	55 min 24 sec	3.3.2
3	C18 Purification	Execute Once		02 hours 11 min 00 sec	3.3.3
<insert sub tasks here>					
4	Evaporation	Execute Once		45 min 04 sec	3.4

5	Transfer to Reaction 2	Execute Once		18 min 08 sec	3.5
6	Sequence	Execute Once		20 hours 12 min 03 sec	3.6
1	MGAT2 Reaction	Execute Once	Reaction	16 hours 02 min 23 sec	3.6.1
1	Add Reagents	Execute Once		33 sec	3.6.1.1
1	Transfer Volumetrically	Transfer liquid from MES Buffer pH 6.5 to Reaction2 with Needle Head #1	Add 250 uL bufffer	33 sec	3.6.1.1.1
2	Other Reagents	Execute If 'Other_Reagents = 1'	Add other reagents	00 sec	3.6.1.1.2
1	Transfer Volumetrically	Transfer liquid from UDP-GlcNAc to Reaction2 with Needle Head #1	transfer sugar nucleotide solution to reaction	01 min 04 sec	3.6.1.1.2.1
2	Transfer Volumetrically	Transfer liquid from Mn_solution to Reaction2 with Needle Head #1	transfer Mn solution to reaction_vial	01 min 01 sec	3.6.1.1.2.2
3	Transfer Volumetrically	Transfer liquid from Enzyme MGAT2 to Reaction2 with Needle Head #1	transfer enzyme solution to reaction_vial	01 min 22 sec	3.6.1.1.2.3
	<insert sub tasks here>				
	<insert sub tasks here>				
2	Synthesis	Execute Once		16 hours 00 min 02 sec	3.6.1.2
3	MS Analysis	Execute Once		01 min 47 sec	3.6.1.3
4	Extend Reaction?	Execute Once		00 sec	3.6.1.4
5	Extend Reaction	Execute If 'Extend_Reaction = 1'		00 sec	3.6.1.5
	<insert sub tasks here>				
2	Continue?	Execute Once		02 sec	3.6.2
3	Purification	Execute Once		03 hours 06 min 26 sec	3.6.3
1	Stir	Agitation OFF on zone shaker		02 sec	3.6.3.1
2	Ni-NTA Purification	Execute Once	Ni-NTA Purification	55 min 24 sec	3.6.3.2
3	C18 Purification	Execute Once		02 hours 11 min 00 sec	3.6.3.3
	<insert sub tasks here>				
4	Evaporation	Execute Once		45 min 04 sec	3.6.4

13. Automated reaction cycles and LC-MS traces

13a. Chemical transformation on the automation platform

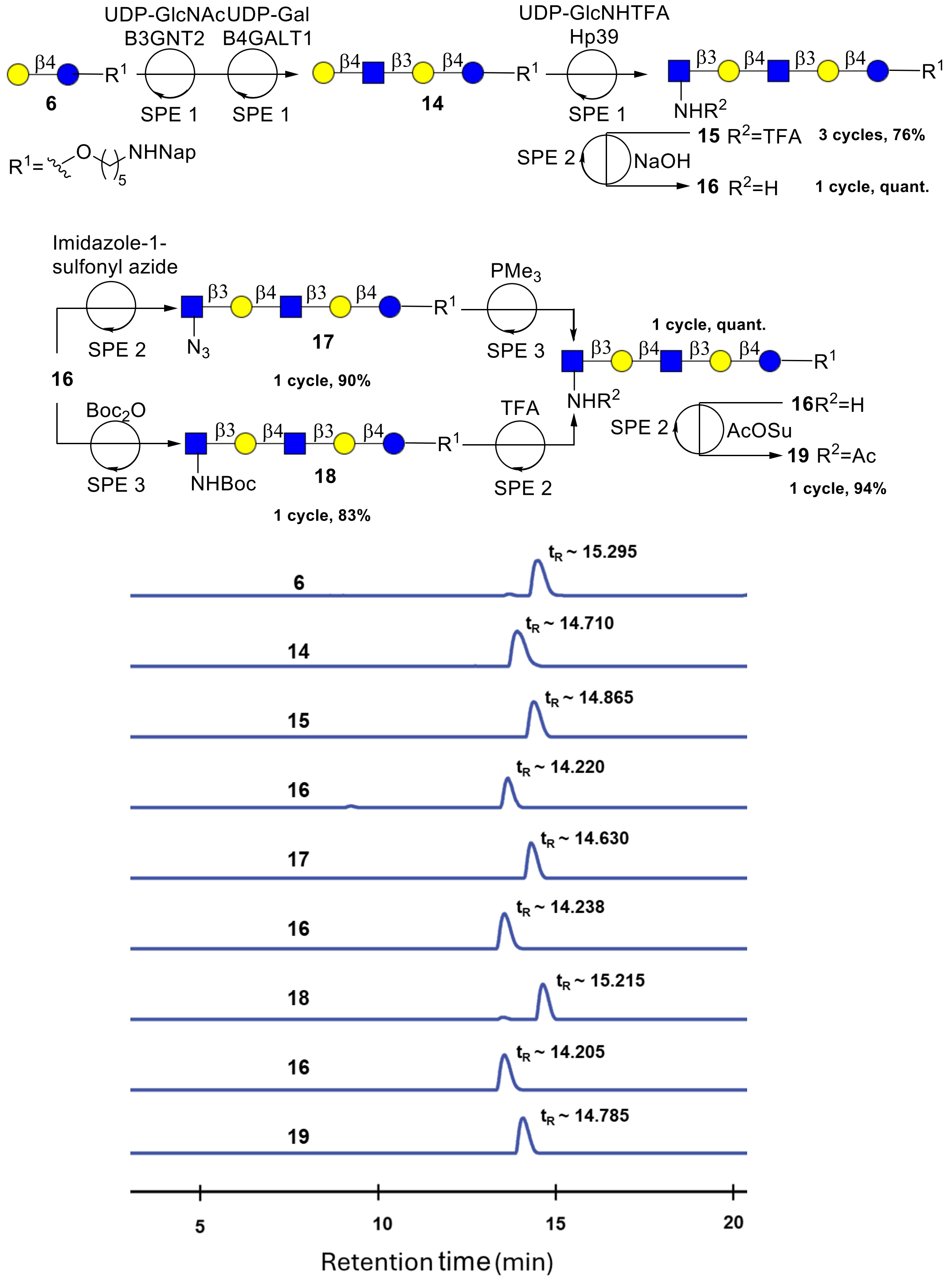

Figure S6. LC-MS traces for chemical transformation.

ESI-MS for intermediate **6**

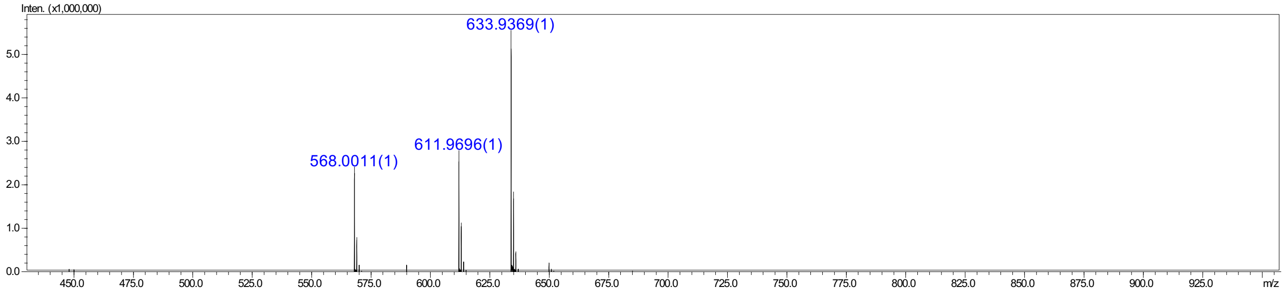

ESI-MS for intermediate **14**

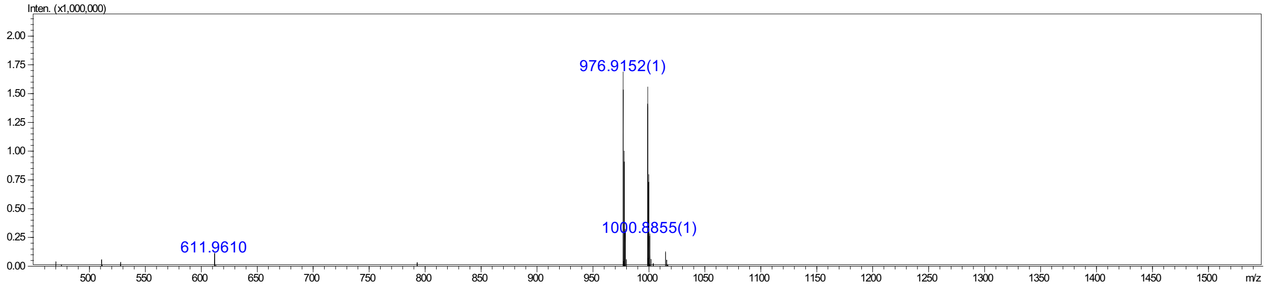

ESI-MS for compound **15**

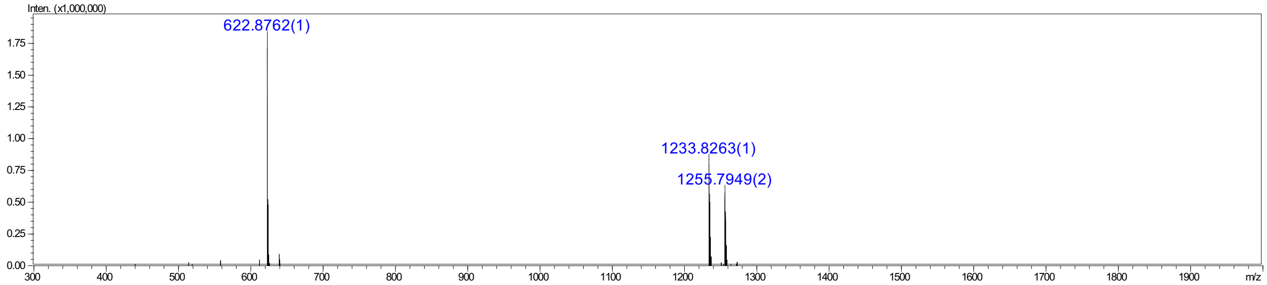

ESI-MS for compound **16**

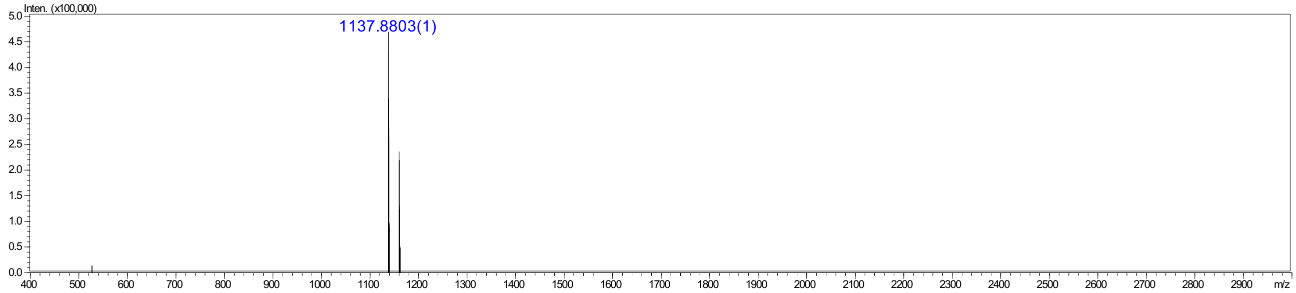

ESI-MS for compound **17**

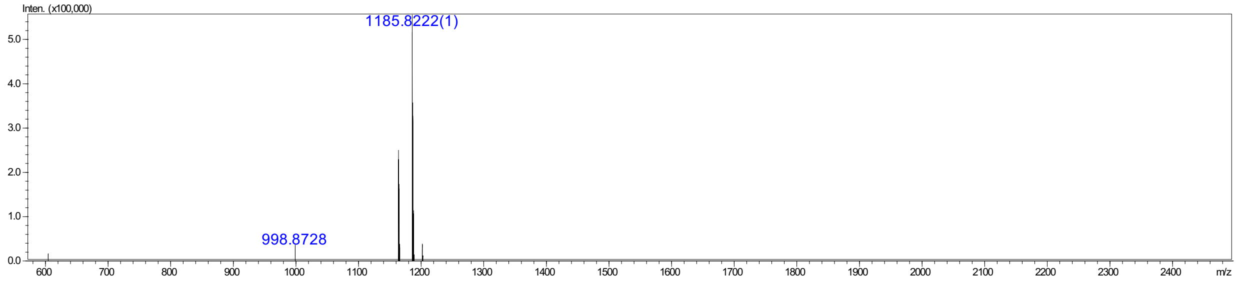

ESI-MS for compound **18**

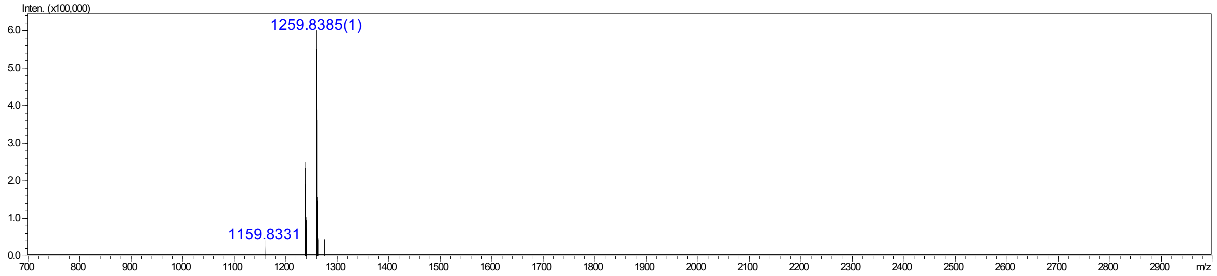

ESI-MS for compound **19**

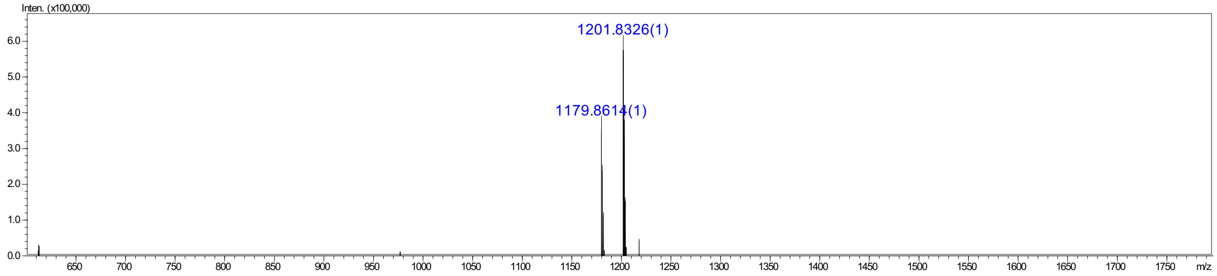

LCMS traces were recorded with method 6a and in positive mode. Washing step artifacts are discarded.

13b. Sulfated glycan synthesis on the automation platform

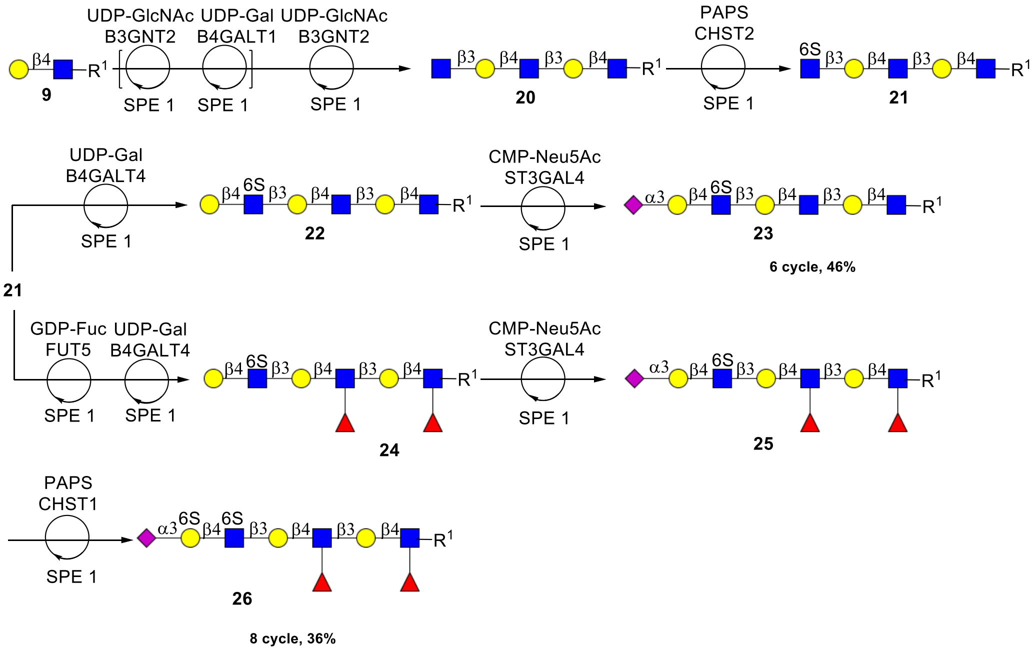

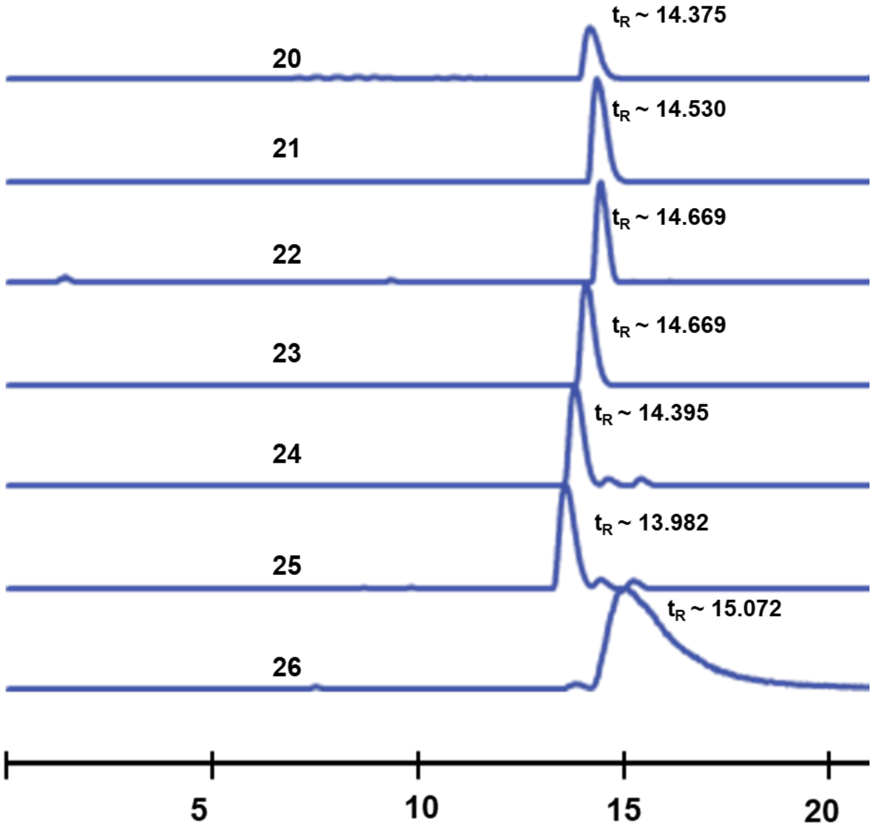

Figure S7. LC-MS traces for automated sulfated glycans synthesis.

ESI-MS for intermediate **20**

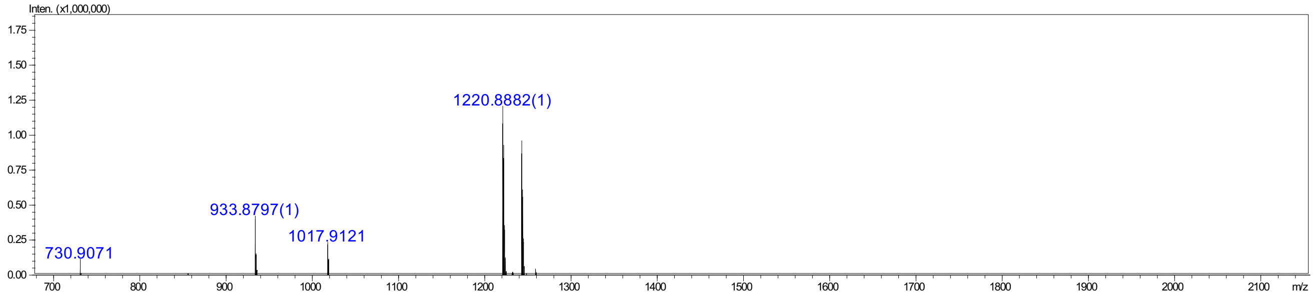

ESI-MS for intermediate **21**

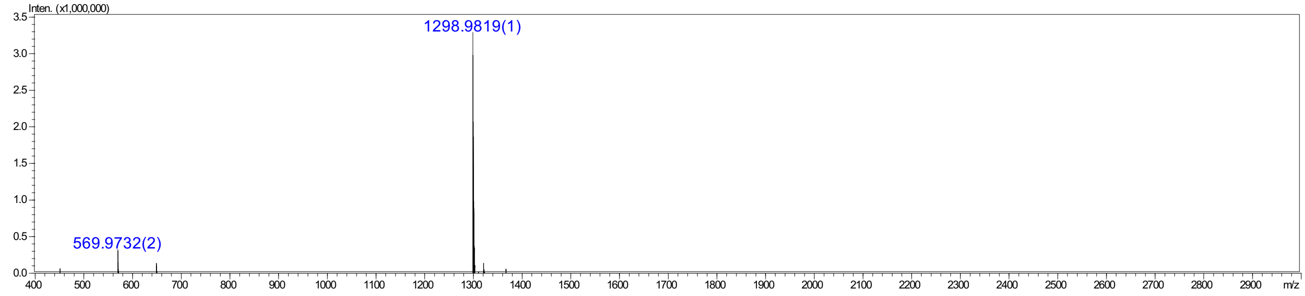

ESI-MS for intermediate **22**

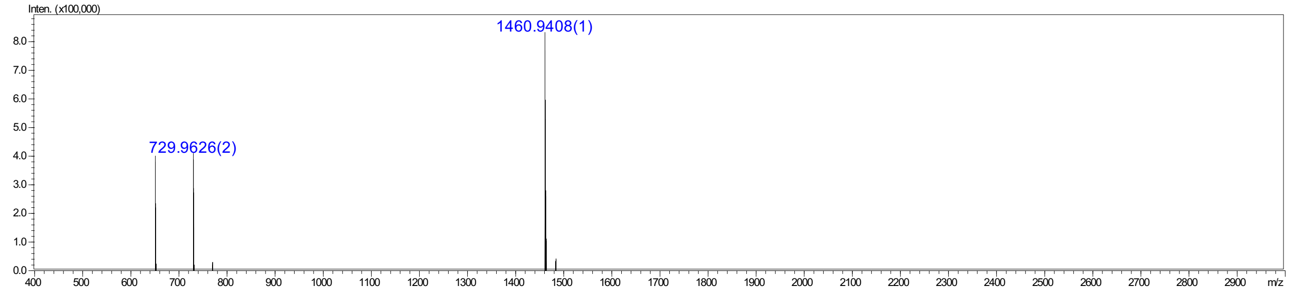

ESI-MS for compound **23**

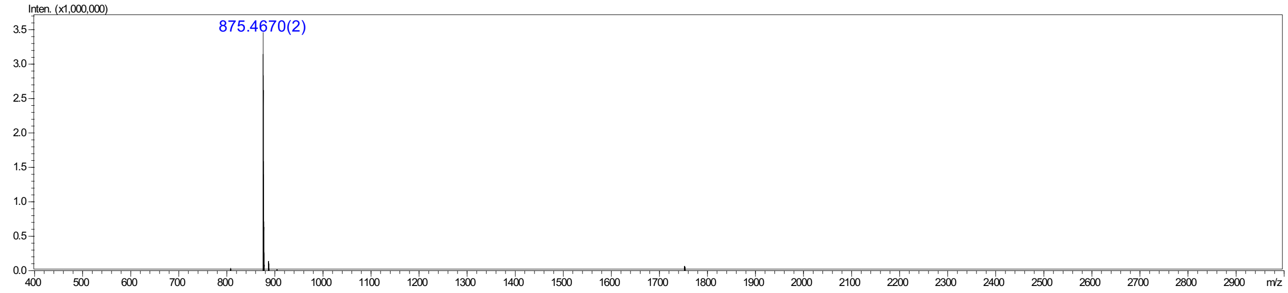

ESI-MS for intermediate **24**

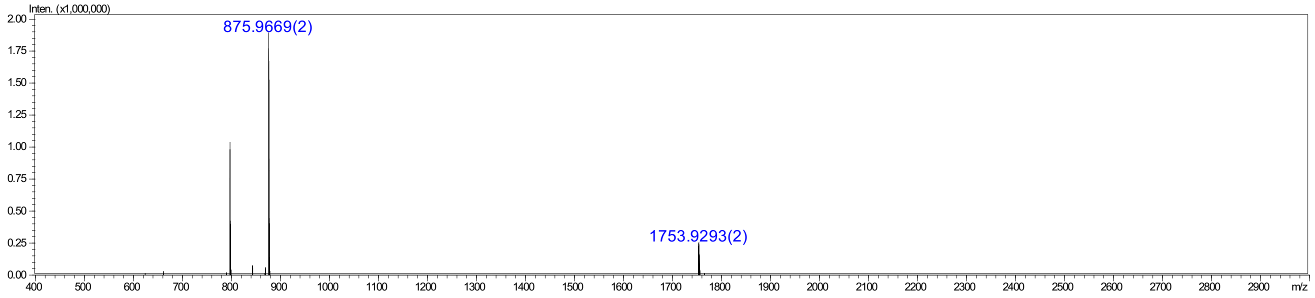

ESI-MS for intermediate **25**

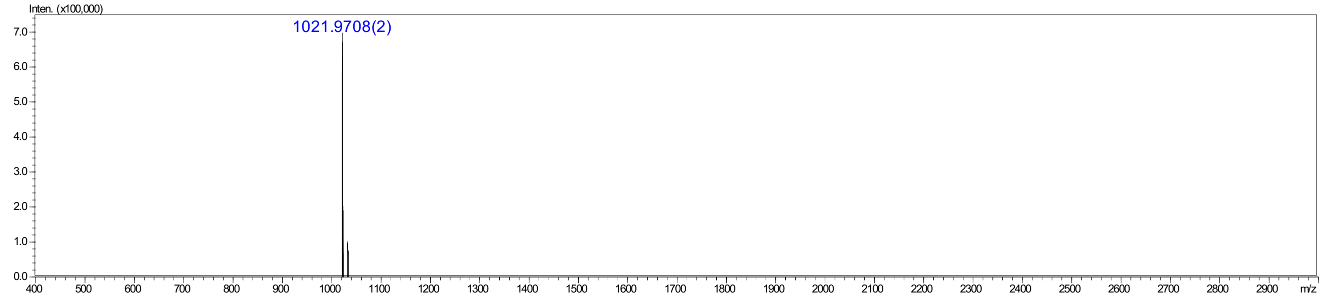

ESI-MS for compound **26**

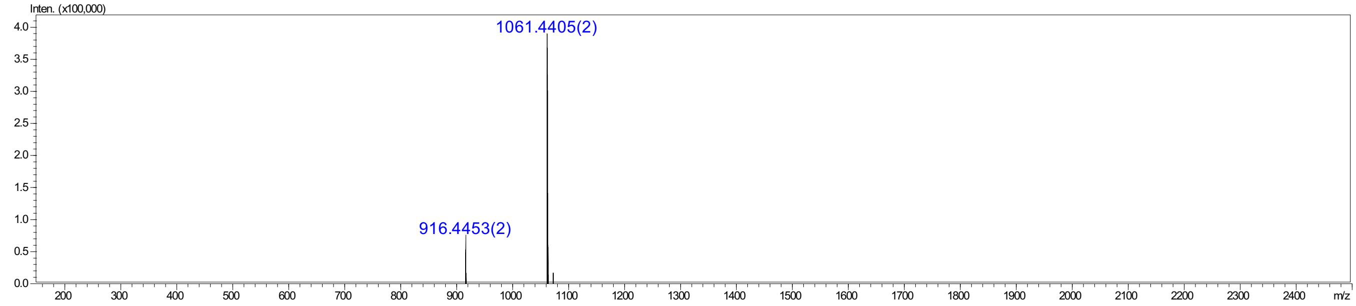

LCMS traces were recorded with method 6a and in positive mode (**9-20)** and negative mode (**21-26)**. Washing step artifacts are discarded. 5% dimethyl sulfoxide was added in the reaction mixture for the first two cycles for better solubility.

13c. Preparation of compound 24 as the starting material for N glycan synthesis

Isolated sialylglycopeptide (SGP) was further trimmed down to a single amino acid (Asn) at the reducing end of the heterogeneous N-glycan (section **3**). Next, the free amine group of the asparagine moiety was converted into its Nap tagged analogue following general procedure **4b** followed by a semi-preparative HPLC purification on Agilent 1100 LC system with a Halo PentaHILIC semi preparative column (2.7 µm, 2.1 mm × 100 mm). Mobile phase A was 10 mM ammonium formate in water, adjusted to pH 3.5 with formic acid; mobile phase B was 100% acetonitrile. The general condition using a linear gradient is as follows:

Time (min)	%A	%B
0	20	80
30	75	25
31	80	20
24	80	20
26	20	80
30	20	80

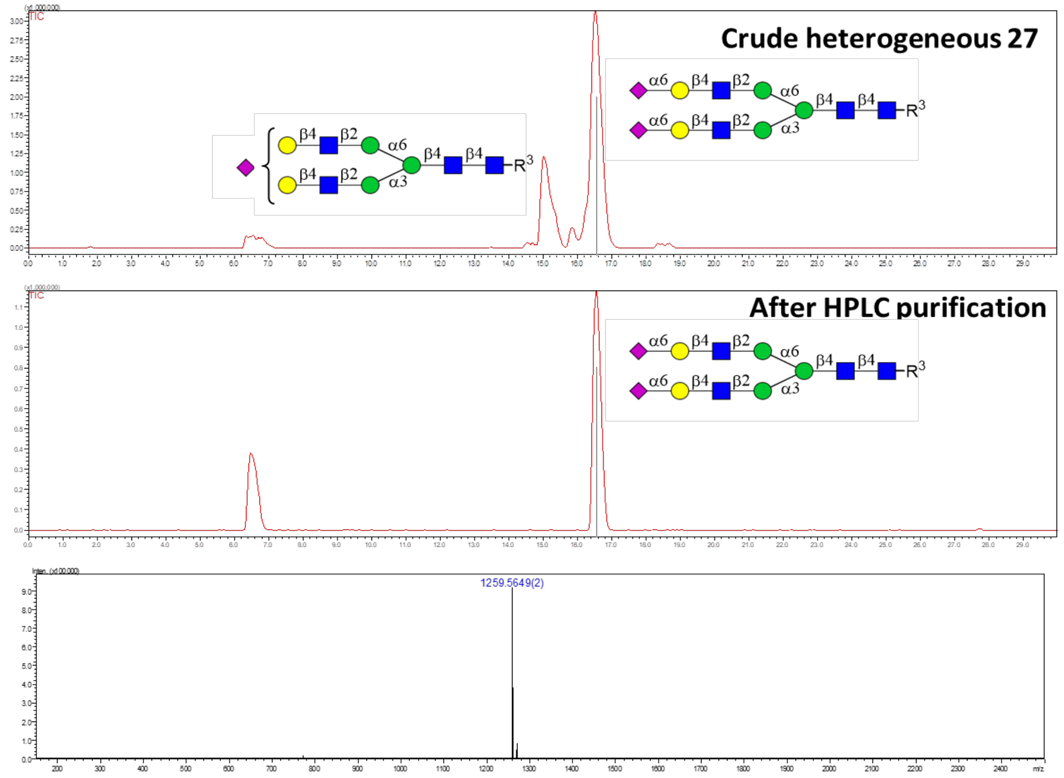

Figure S8. LC-MS traces for compound **27** before and after HPLC purification. The common peak at 6-7 min is sodium and ammonium formate salt artifact.

13c.1. Automated synthesis of N-glycan 34

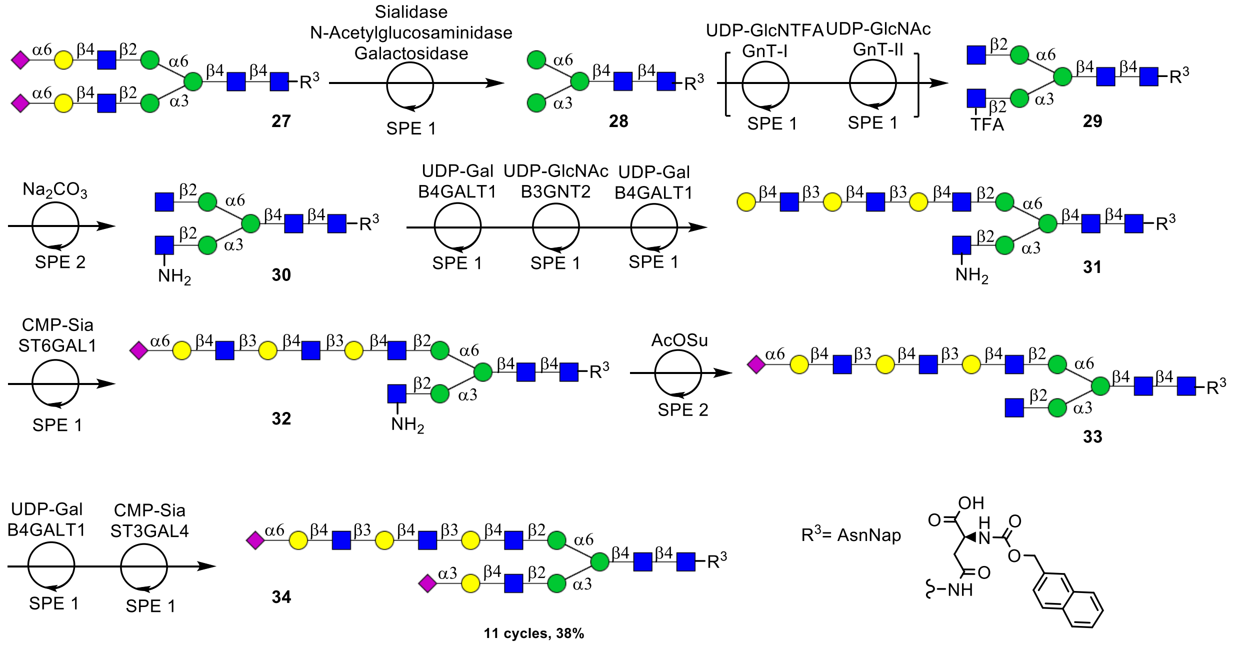

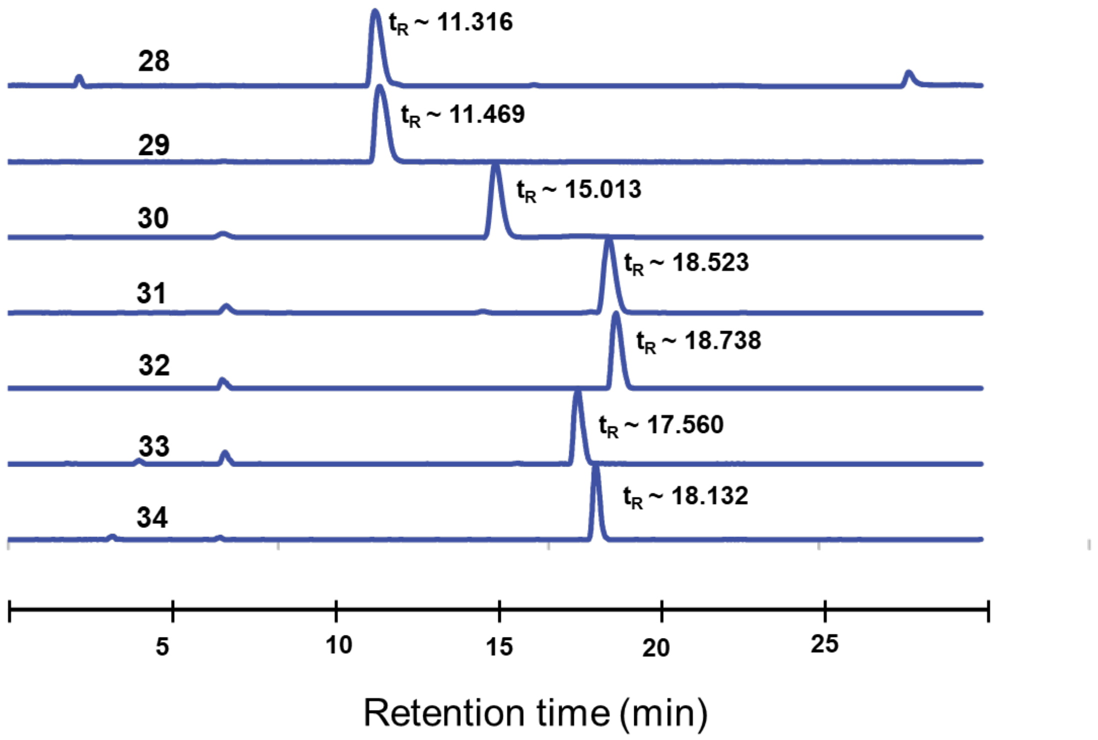

Figure S9. LC-MS traces for automated synthesis of N-glycan **34**.

ESI-MS for compound **28**

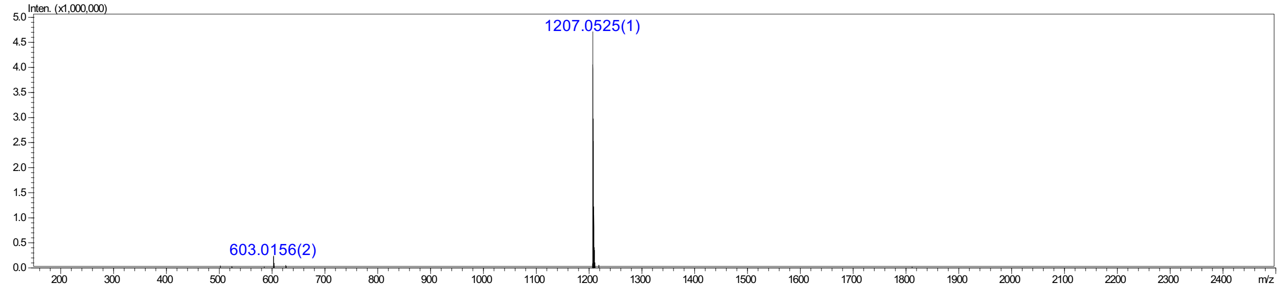

ESI-MS for intermediate **29**

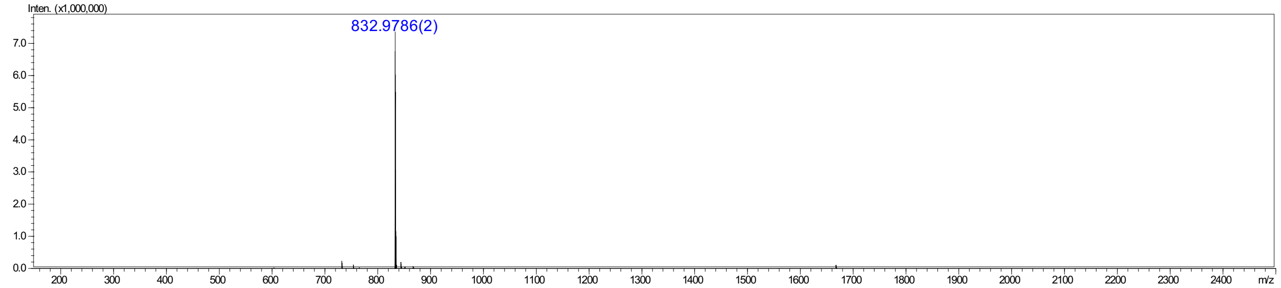

ESI-MS for intermediate **30**

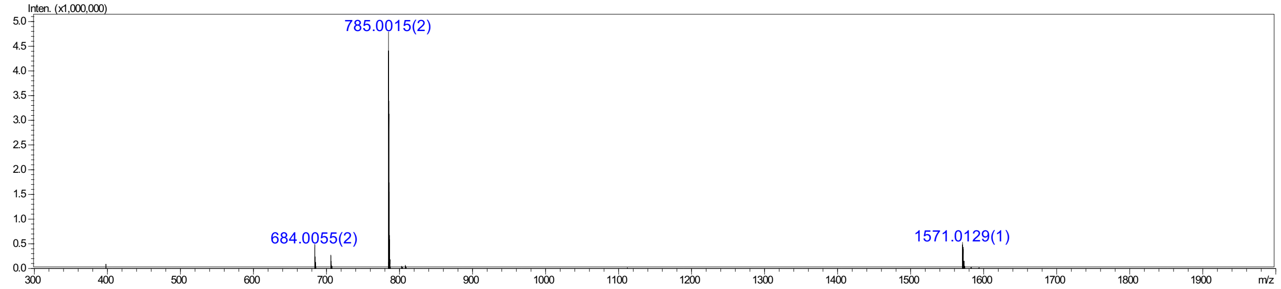

ESI-MS for intermediate **31**

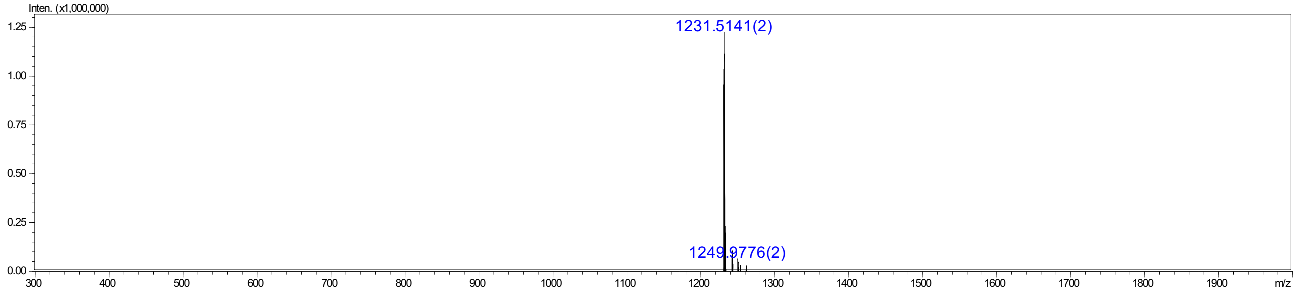

ESI-MS for intermediate **32**

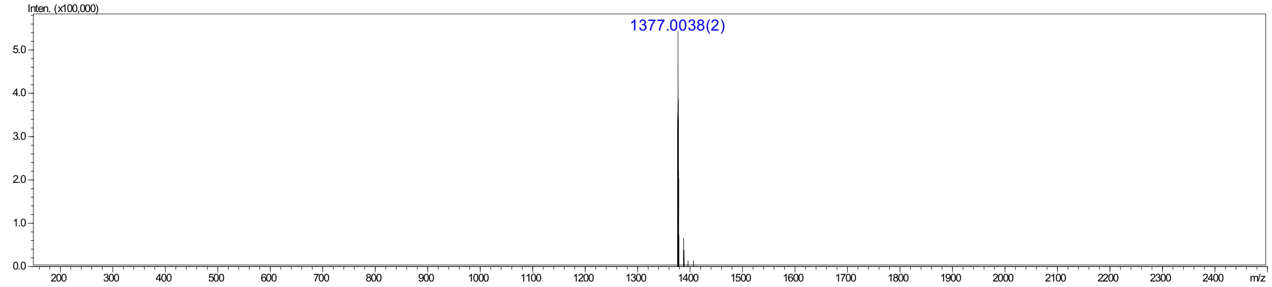

ESI-MS for intermediate **33**

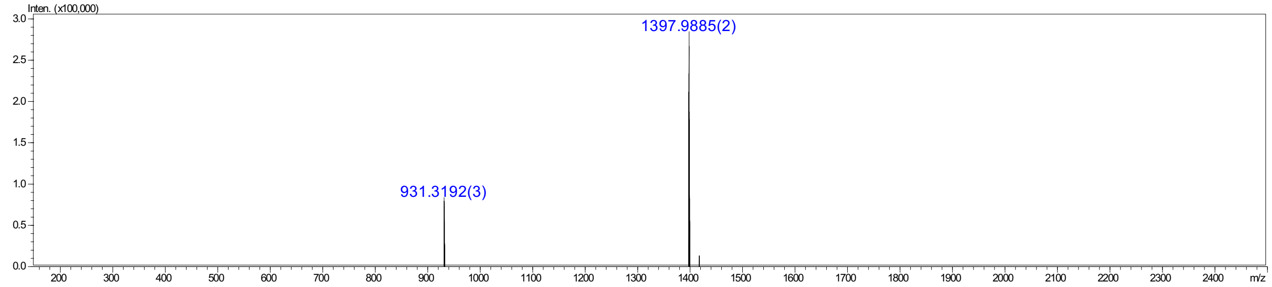

ESI-MS for compound **34**

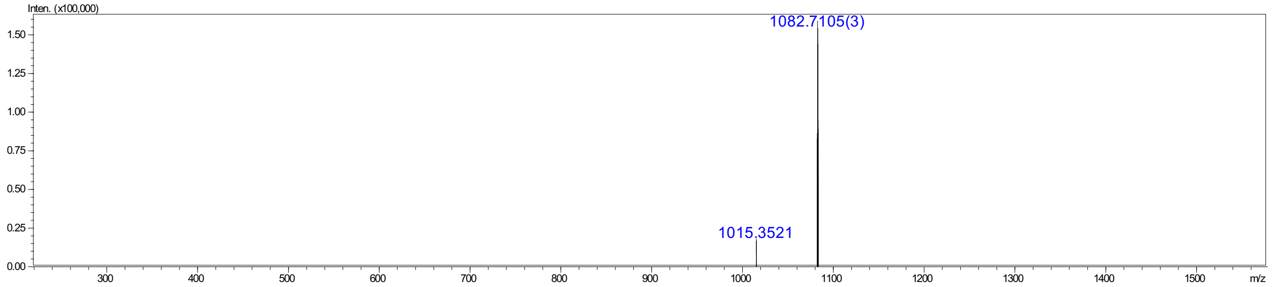

LCMS traces were recorded with method 6b and in negative mode. The common peak at 6-7 min is sodium and ammonium formate salt artifact.

13c.2. Automated synthesis of N-glycan 44

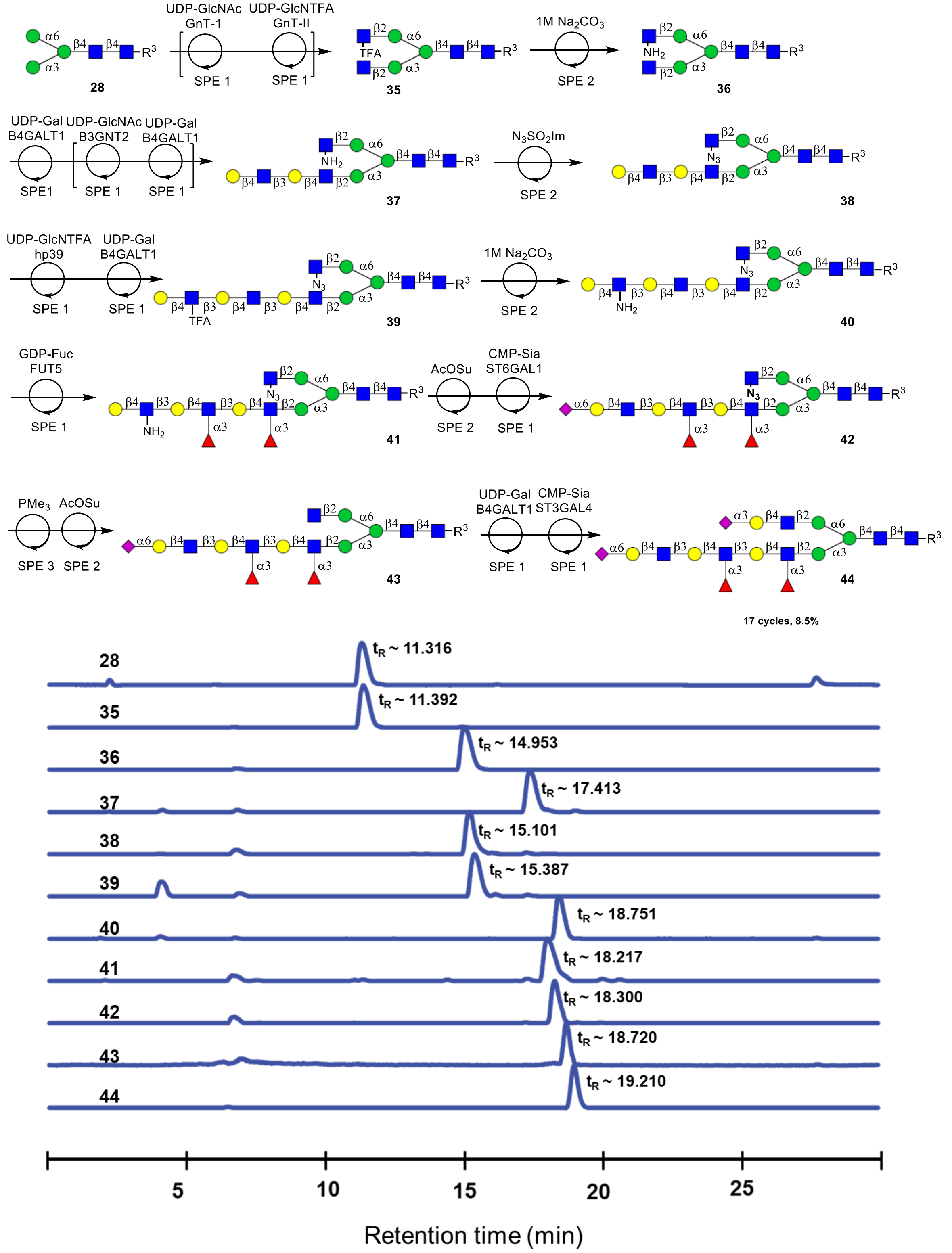

Figure S10. LC-MS traces for automated synthesis of N-glycan **44**.

ESI-MS for intermediate **35**

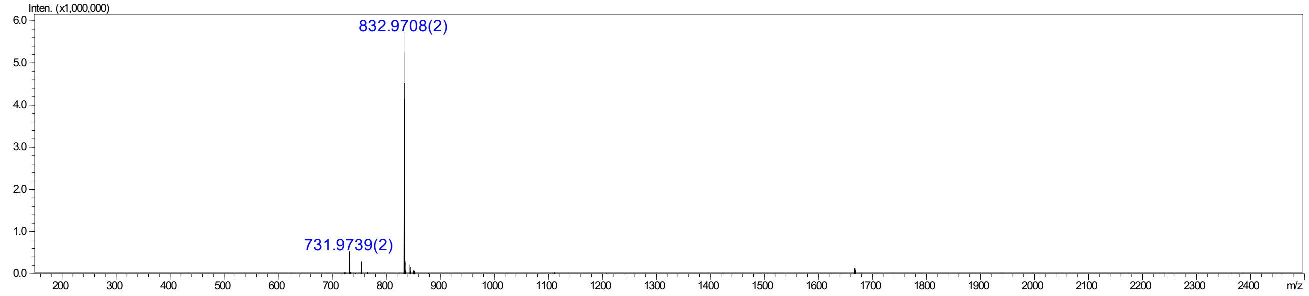

ESI-MS for intermediate **36**

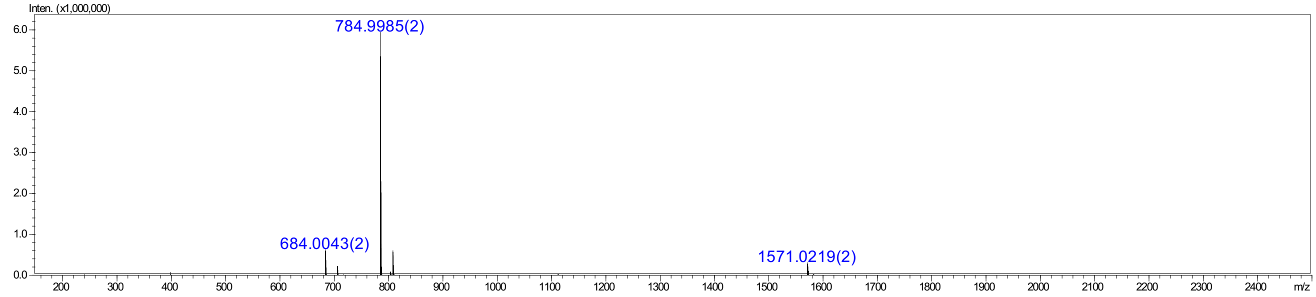

ESI-MS for intermediate **37**

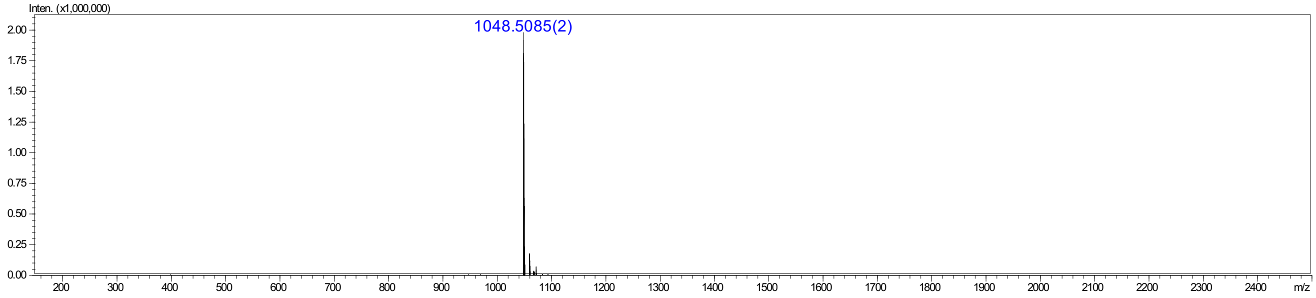

ESI-MS for intermediate **38**

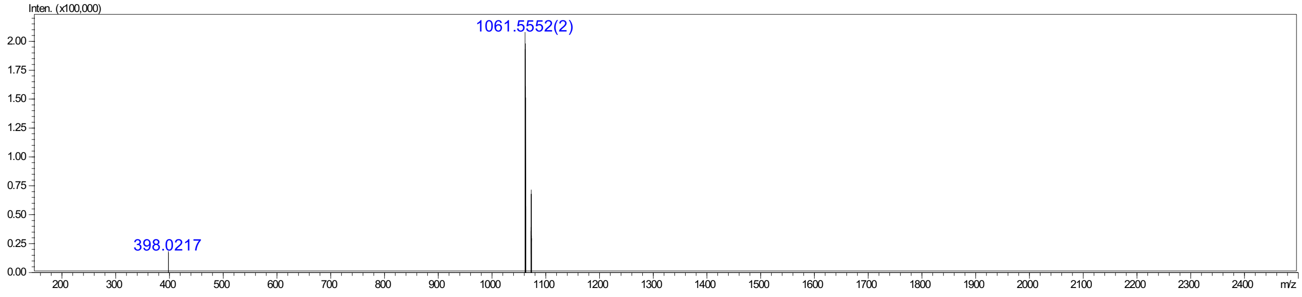

ESI-MS for intermediate **39**

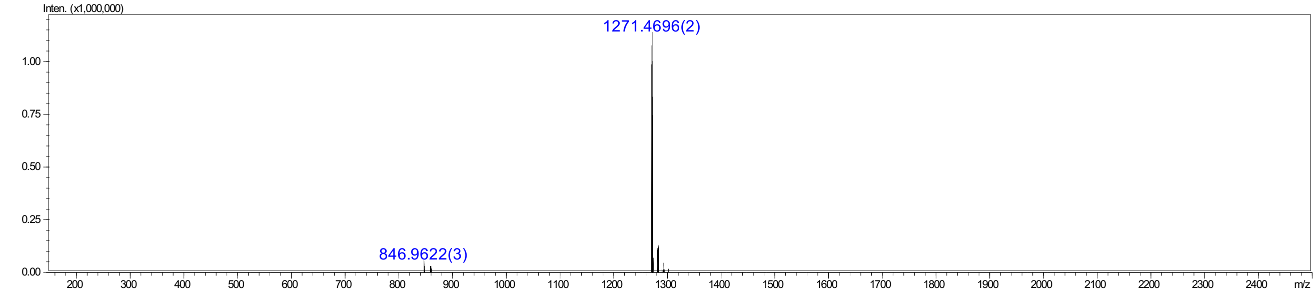

ESI-MS for intermediate **40**

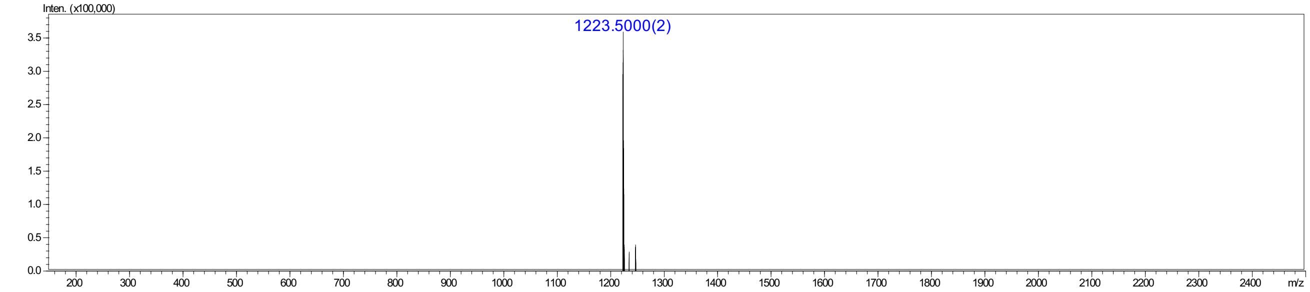

ESI-MS for intermediate **41**

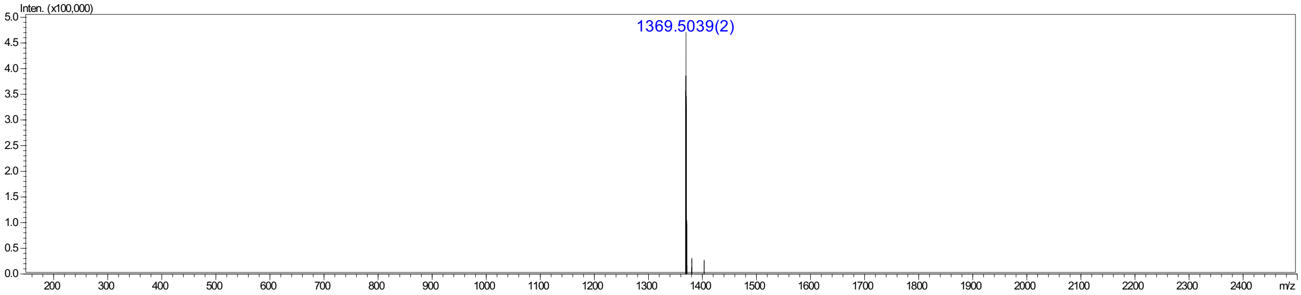

ESI-MS for intermediate **42**

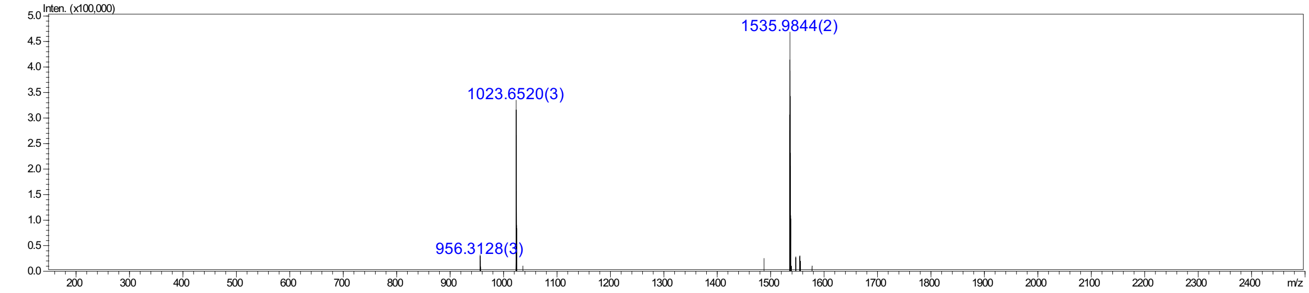

ESI-MS for intermediate **43**

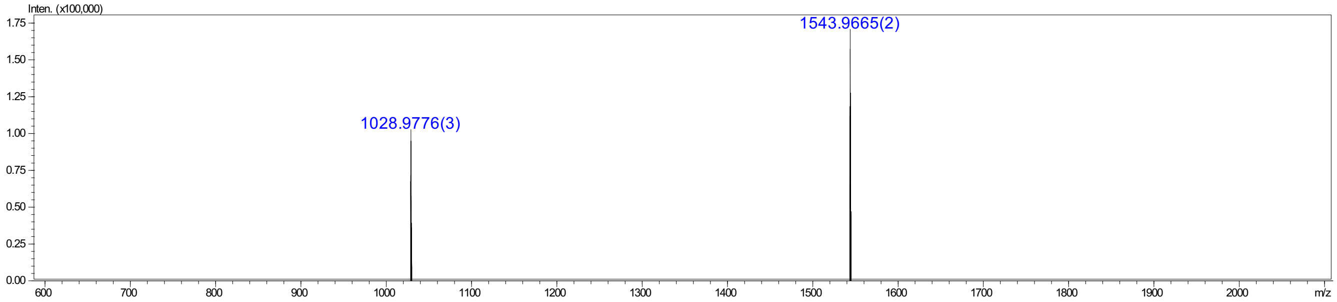

ESI-MS for compound **44**

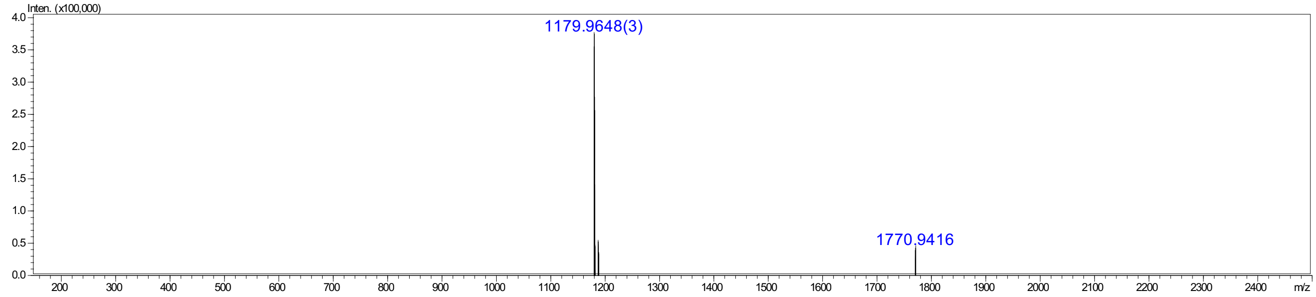

LCMS traces were recorded with method 6b and in negative mode.

13c.3. Automated synthesis of N-glycan 52

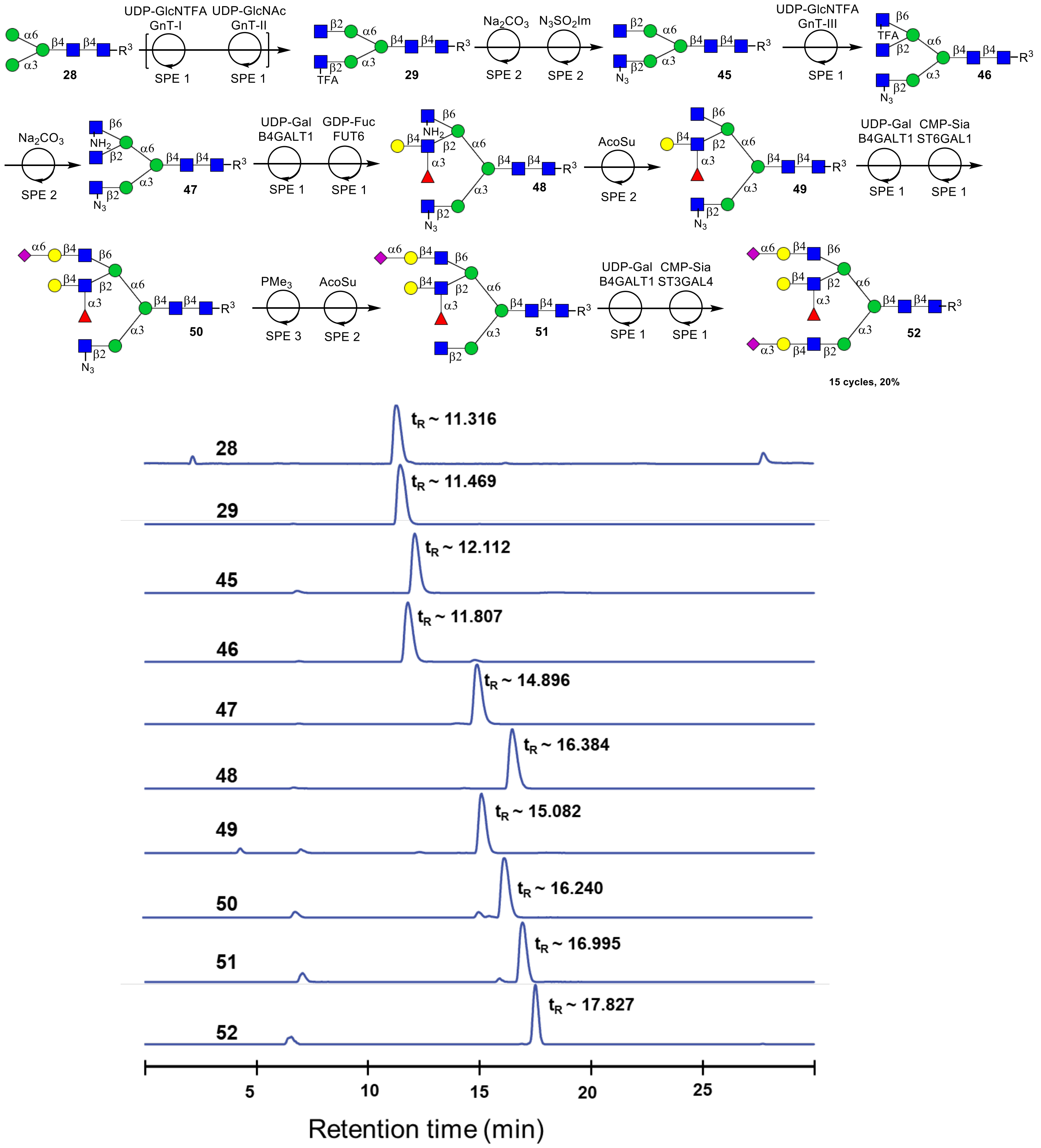

Figure S11. LC-MS traces for automated synthesis of N-glycan **52**.

ESI-MS for compound **45**

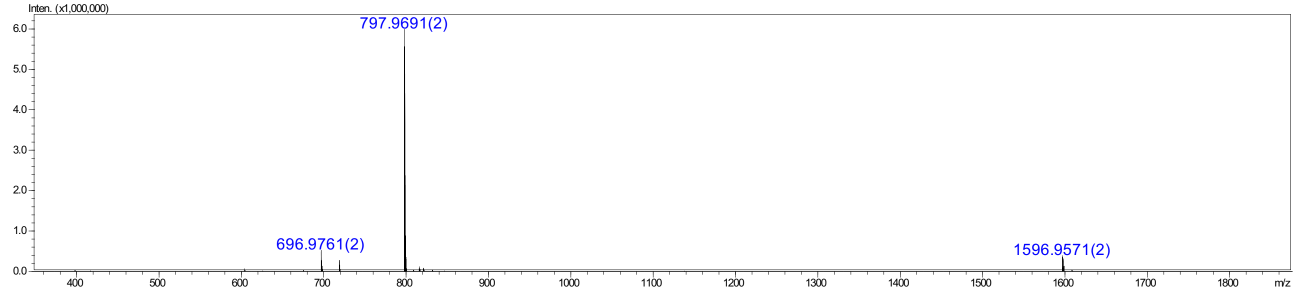

ESI-MS for compound **46**

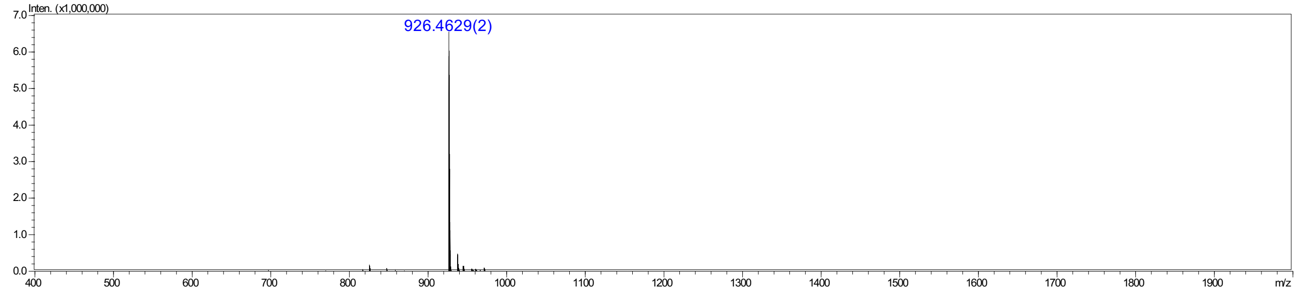

ESI-MS for compound **47**

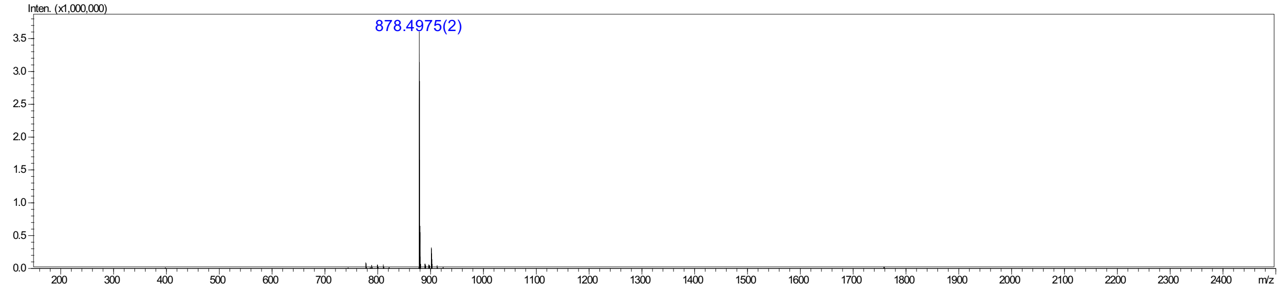

ESI-MS for compound **48**

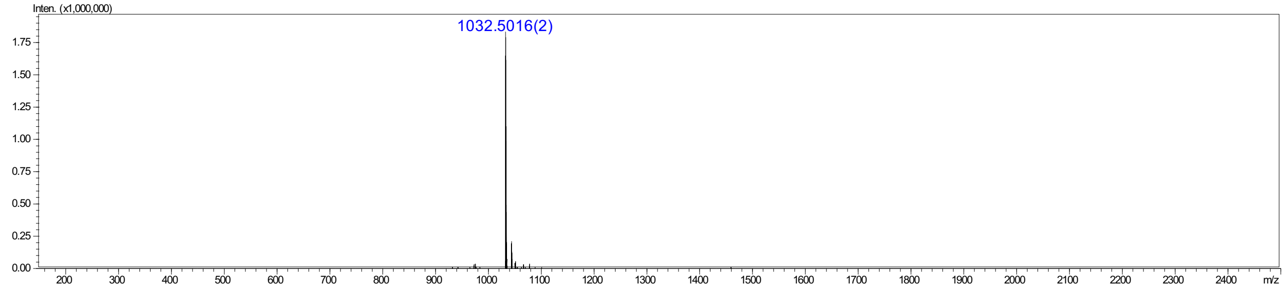

ESI-MS for compound **49**

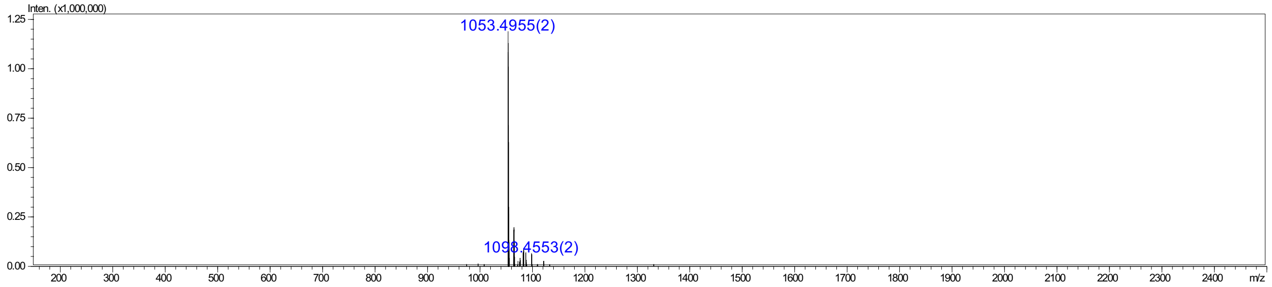

ESI-MS for compound **50**

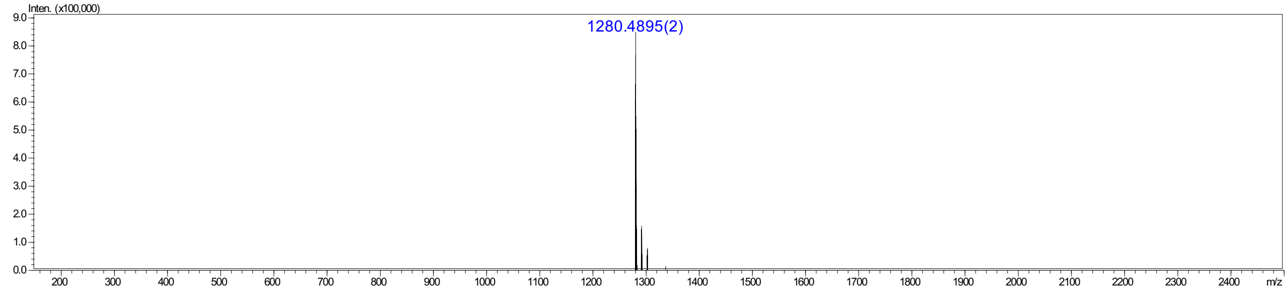

ESI-MS for compound **51**

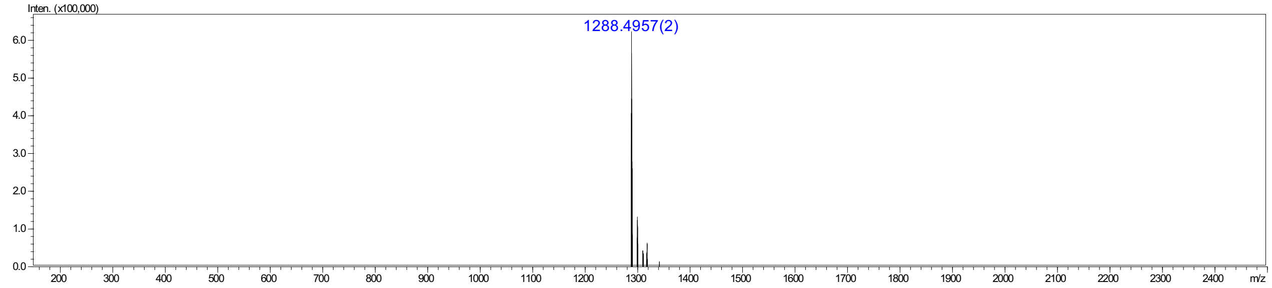

ESI-MS for compound **52**

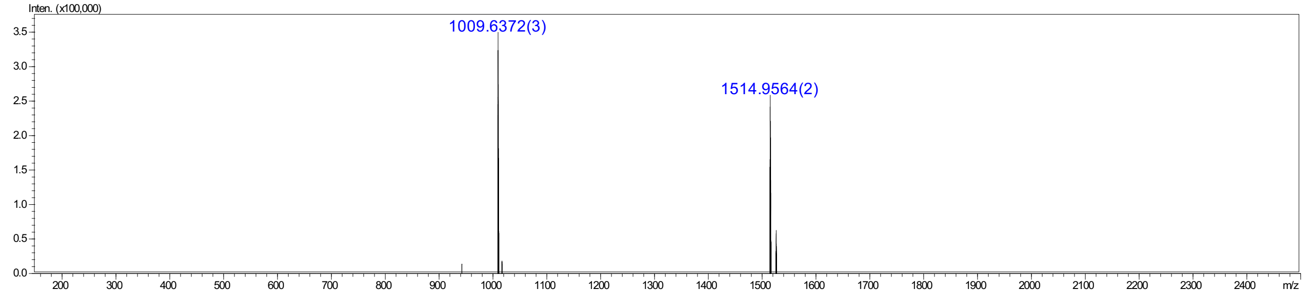

LCMS traces were recorded with method 6b and in negative mode. Cycle 5 (compound **46**) was repeated twice to push the reaction to completion.

14. NMR nomenclature

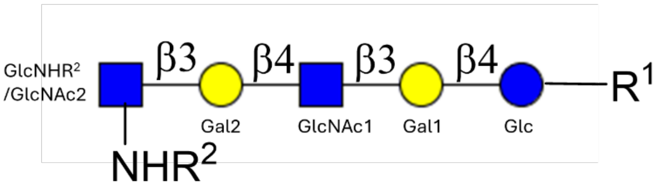

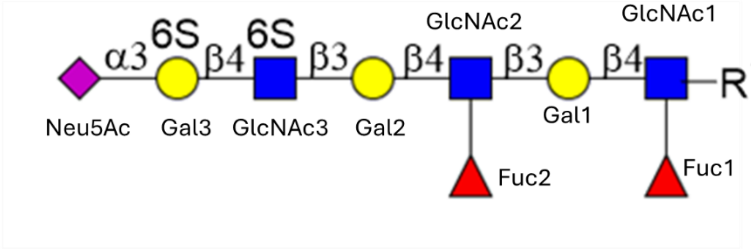

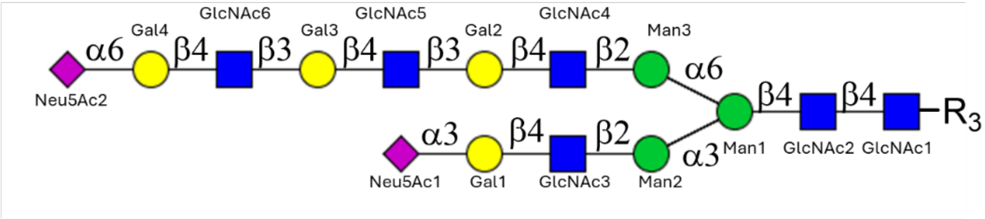

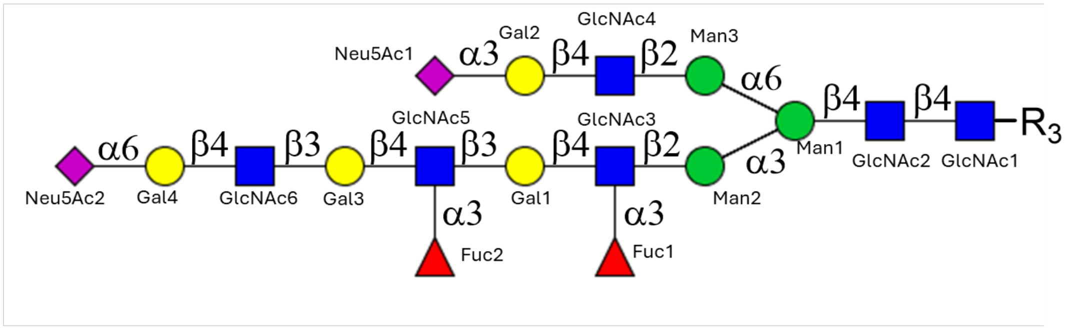

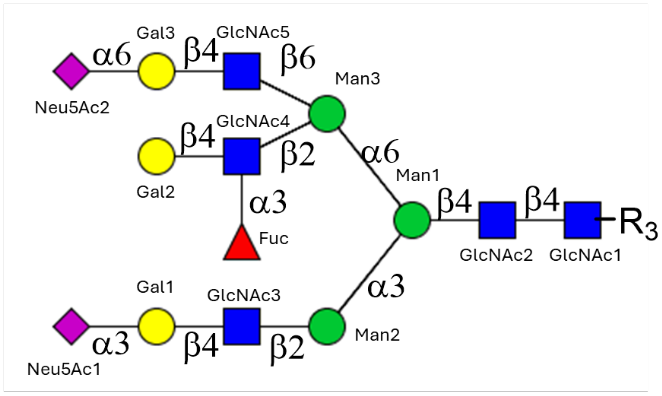

15. Characterization data

15.1. Compound 6: Compound **6** was prepared according to general procedure 4b from compound **5** (25 mg, 0.059 mmol). Crude reaction mixture was purified through flash silica gel chromatography to obtain pure **2** as a white solid (32 mg, 91%).

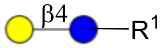

^{1}H NMR (Methanol-d4, 600 MHz): δ (ppm)

	H1	H2	H3	H4	H5	H6
**Glc**	4.28 (d, J = 7.8 Hz)	3.23	3.51	3.54	3.47	3.79, 3.71
**Gal**	4.34 (d, J = 7.7Hz	3.54	3.39	3.84	3.58	3.88,3.85

^{1}H NMR (non-carbohydrate) **(Methanol-d4, 600 MHz): δ (ppm)** 1.44-1.40 (m, 2H, linker C*H$_2$*), 1.57-1.51 (m, 2H, linker C*H$_2$*), 1.65-1.63 (m, 2H, linker C*H$_2$*), 3.15-3.13 (m, 2H, linker -NHC*H$_2$*),3.88,3.55 (m, 2H, linker -O-*CH$_2$*-), 5.22 (s, 2H, *CH$_2$*-Nap), 7.86-7.45 (m, 7H, aromatic)

ESI TOF-MS m/z calcd for $C_{29}H_{41}NO_{13}$ $[M+H]^+$: 612.2656, found 611.9696.

15.2. Compound 7: Compound **7** was prepared according to Table S2 automation program from compound **6** (5 mg, 0.082 mmol). Fraction containing products were pooled and lyophilized to give **7** as a white fluffy solid (6.2 mg, 94%).

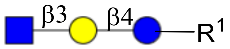

^{1}H NMR (D_2O, 600 MHz): δ (ppm)

	H1	H2	H3	H4	H5	H6
**Glc**	4.32 (d, J = 7.9 Hz)	3.24	3.59	3.54	3.47	3.70-3.60
**Gal**	4.40 (d, J = 7.8 Hz)	3.61	3.70	4.14	3.56	3.68-3.64
**GlcNAc**	4.69 (d, J = 8.4 Hz)	3.76	3.90	3.58	-	3.82-3.76

^{1}H NMR (non-carbohydrate) **(D_2O, 600 MHz): δ (ppm)** 1.25-1.20 (m, 2H, linker C*H$_2$*), 1.42-1.35 (m, 2H, linker C*H$_2$*), 1.53-1.45 (m, 2H, linker C*H$_2$*), 3.05-2.99 (m, 2H, linker -NHC*H$_2$*),3.73,3.46 (m, 2H, linker -O-*CH$_2$*-), 5.1 (s, 2H, *CH$_2$*-Nap), 7.71-7.25 (m, 7H, aromatic)

ESI TOF-MS m/z calcd for $C_{37}H_{54}N_2O_{18}$ $[M+H]^+$: 815.3450, found 814.9368.

15.3. Compound 9: Compound **9** was prepared according to general procedure 4b from compound **8** (25 mg, 0.054 mmol). Crude reaction mixture was purified through flash silica gel chromatography to obtain pure **9** as a white solid (33 mg, 95%).

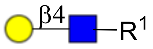

^{1}H NMR (DMSO-d6, 600 MHz): δ (ppm)

	**H1**	**H2**	**H3**	**H4**	**H5**	**H6**	NH**Ac**
**GlcNAc**	4.32(d, J = 7.4 Hz)	3.47	3.25	3.52	3.44	3.75, 3.62	1.77
**Gal**	4.23(d, J = 5.8 Hz)	3.32	3.62	3.84	3.58	3.51, 3.45	-

^{1}H NMR (non-carbohydrate) **(DMSO-d6, 600 MHz): δ (ppm)** 1.44-1.40 (m, 2H, linker C*H₂*), 1.57-1.51 (m, 2H, linker C*H₂*), 1.65-1.63 (m, 2H, linker C*H₂*), 3.15-3.13 (m, 2H, linker -NHC*H₂*),3.88,3.55 (m, 2H, linker -O-*CH₂*-), 5.22 (s, 2H, *CH₂*-Nap), 7.86-7.45 (m, 7H, aromatic)

ESI TOF-MS m/z calcd for $C_{31}H_{44}N_2O_{13}$ $[M+H]^+$: 654.2922, found 653.9717

15.4. Compound 10: Compound **10** was prepared according to Table S2 automation program from compound **9** (5 mg, 0.076 mmol). Fraction containing products were pooled and lyophilized to give **10** as a white fluffy solid (6 mg, 92%).

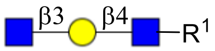

^{1}H NMR (DMSO-d6, 600 MHz): δ (ppm)

	**H1**	**H2**	**H3**	**H4**	**H5**	**H6**	NH**Ac**
**GlcNAc1**	4.30	3.45	3.09	3.32	3.43	3.76, 3.63	1.82-1.77 (6H)
**Gal**	4.26	3.42	3.46	3.84	3.48	3.51, 3.45	-
**GlcNAc2**	4.63	3.35	3.10	3.47	3.43	3.75-3.63	1.82-1.77 (6H)

^{1}H NMR (non-carbohydrate) **(DMSO-d6, 600 MHz): δ (ppm)** 1.44-1.40 (m, 2H, linker C*H₂*), 1.57-1.51 (m, 2H, linker C*H₂*), 1.65-1.63 (m, 2H, linker C*H₂*), 2.95-2.93 (m, 2H,

linker -NHCH_2),3.88,3.55 (m, 2H, linker -O-CH_2-), 5.22 (s, 2H, CH_2-Nap), 7.86-7.45 (m, 7H, aromatic).

ESI TOF-MS m/z calcd for $C_{39}H_{57}N_3O_{18}$ $[M+H]^+$: 856.3715, found 855.9476.

15.5. Compound 12: Compound **12** was prepared according to according to general procedure 4b from compound **11** (20 mg, 13.9 μmol). Crude reaction mixture was purified through P2 biogel size exclusion chromatography in 100mM Ammonium bicarbonate solution. Fraction containing products were pooled and lyophilized to give **12** as a white fluffy solid (21 mg, 93%).

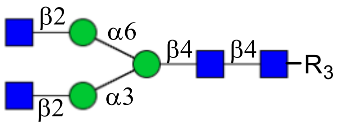

^{1}H NMR (D_2O, 600 MHz): δ (ppm)

	H1	H2	H3	H4	H5	H6	NH**Ac**
**GlcNAc1**	4.95	3.77	3.74	3.62	N/A	N/A[a]	2.12-2.06 12H
**GlcNAc2**	4.57	3.80	3.74	N/A	N/A	N/A	2.12-2.06 12H
**GlcNAc3**	4.58	3.75	3.58	3.48	N/A	N/A	2.12-2.06 12H
**GlcNAc4**	4.58	3.73	3.58	3.47	N/A	N/A	2.12-2.06 12H
**Man1**	4.79	4.27	3.78	3.65	3.54	N/A	-[b]
**Man2**	5.14	4.20	3.93	3.58	3.76	N/A	-
**Man3**	4.94	4.13	3.91	3.58	3.64	N/A	-

a=Not assigned,b=Not applicable

**Signal**	**Proton**	**Carbon**
Aromatic (Naphthyl)	8.04-7.94 (m, 4H)<br>7.64-7.56 (m, 3H)	128.1-125.3
-**CH_2**Ph	5.40 (d, J = 12.7 Hz)<br>5.24 (d, J = 12.7 Hz)	67.0
-NH-**CH**-COOH	4.44 (dd, J = 9.6, 4.1 Hz)	52.8
-C(O)-**CH_2**-CH	2.87 (dd, J = 15.3, 4.2 Hz)<br>2.62 (dd, J = 15.2, 9.6 Hz)	38.7

^{13}C NMR (D_2O, 150 MHz): δ (ppm)

	**Carbon (C1)**
**GlcNAc 1**	78.1
**GlcNAc 2**	101.2
**GlcNAc 3**	99.5
**GlcNAc 4**	99.5
**Man1**	100.5
**Man2**	99.5
**Man3**	96.9

ESI TOF-MS m/z calcd for $C_{66}H_{96}N_6O_{40}^{2-}$: 806.2836, found 806.0382

15.6. Compound 13: Compound **13** was prepared according to Table S1 automation program from compound **12** (5 mg, 3.09 µmol). Fraction containing products were pooled and lyophilized to give **13** as a white fluffy solid (5.7 mg, 95%).

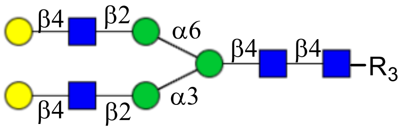

^{1}H NMR (D_2O, 600 MHz): δ (ppm)

	**H1**	**H2**	**H3**	**H4**	**H5**	**H6**	NH**Ac**
**GlcNAc1**	4.95	3.77	3.74	3.62	N/A	N/A[a]	2.12-2.06 12H
**GlcNAc2**	4.57	3.80	3.74	N/A	N/A	N/A	2.12-2.06 12H
**GlcNAc3**	4.58	3.75	3.58	3.48	N/A	N/A	2.12-2.06 12H
**GlcNAc4**	4.58	3.71	3.58	3.47	N/A	N/A	2.12-2.06 12H
**Man1**	4.79	4.27	3.78	3.65	3.54	N/A	-[b]
**Man2**	5.14	4.20	3.93	3.58	3.76	N/A	-
**Man3**	4.94	4.13	3.91	3.58	3.64	N/A	-
**Gal1**	4.47	3.54	3.67	3.92	N/A	N/A	-
**Gal2**	4.47	3.54	3.67	3.92	N/A	N/A	-

a=Not assigned, b=Not applicable

**Signal**	**Proton**	**Carbon**
Aromatic (Naphthyl)	8.04-7.94 (m, 4H)<br>7.64-7.56 (m, 3H)	128.1-125.3
-**CH_2**Ph	5.40 (d, J = 12.7 Hz)<br>5.24 (d, J = 12.7 Hz)	67.0
-NH-**CH**-COOH	4.44 (dd, J = 9.6, 4.1 Hz)	52.8

-C(O)-**CH2**-CH	2.87 (dd, J = 15.3, 4.2 Hz)<br>2.62 (dd, J = 15.2, 9.6 Hz)	38.7

^{13}C NMR (D_2O, 150 MHz): δ (ppm)

	**Carbon (C1)**
**GlcNAc 1**	78.1
**GlcNAc 2**	101.2
**GlcNAc 3**	99.5
**GlcNAc 4**	99.5
**Man1**	100.5
**Man2**	99.5
**Man3**	96.9
**Gal1**	102.7
**Gal2**	102.7

ESI TOF-MS m/z calcd for $C_{78}H_{116}N_6O_{50}^{2-}$: 968.3365, found 968.0293

15.7. Compound 15: Compound **15** was prepared from compound **6** (5mg, 8.17 μmol). Fraction containing products were pooled and lyophilized to give **13** as a white fluffy solid (7.6 mg, 76%).

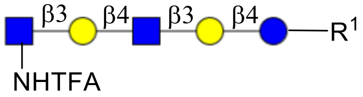

^{1}H NMR (D_2O, 600 MHz): δ (ppm)

	**H1**	**H2**	**H3**	**H4**	**H5**	**H6**	NH**Ac**
**Glc**	4.36	3.26	3.59	3.56	N/A	3.79	-[b]
**Gal1**	4.40	3.59	3.74	4.18(d, *J*=3.3 Hz)	N/A	N/A[a]	-[b]
**GlcNAc1**	4.71	3.80	3.49	3.62	N/A	N/A	2.04 (s)
**Gal2**	4.47	3.60	3.74	4.15 (d, *J*=3.3 Hz)	N/A	N/A	-[b]
**GlcNHTFA**	4.80	3.86	3.51	3.65	N/A	N/A	-[b]

a=Not assigned, b=Not applicable

**Signal**	**Proton**	**Carbon**
Aromatic (Naphthyl)	7.98-7.91 (m, 4H)<br>7.60-7.54 (m, 3H)	128.1-125.0
-**CH2**Ph	5.26 (s)	66.7
-NH-**CH2**- (linker)	3.15-3.13(m)	40.1

-O-**CH₂**- (linker)	3.79,3.54	74.2
**C-CH₂-C** (linker)	1.60-1.57 (m, 2H),1.53-1.48 (m, 2H), 1.34-1.32 (m, 2H)	28.4, 28.1,22.5

^{13}C NMR (D_2O, 150 MHz): δ (ppm)

	**Carbon (C1)**
**Glc**	101.9
**Gal1**	102.8
**GlcNAc1**	102.5
**Gal2**	102.8
**GlcNHTFA**	101.9

ESI TOF-MS m/z calcd for $C_{51}H_{74}F_3N_3O_{28}$ $[M+H]^+$: 1234.4489, found 1233.8263

15.8. Compound 16: Compound **16** was prepared from compound **15** (3mg, 8.17 μmol). Fraction containing products were pooled and lyophilized to give **16** as a white fluffy solid (2.5 mg, quantitative)

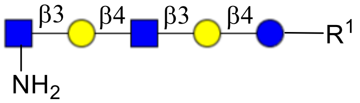

^{1}H NMR (D_2O, 600 MHz): δ (ppm)

	**H1**	**H2**	**H3**	**H4**	**H5**	**H6**	NH**Ac**
**Glc**	4.45	3.32	3.67	3.56	N/A	3.79	-[b]
**Gal1**	4.41	3.67	3.71	4.25	N/A	N/A[a]	-[b]
**GlcNAc1**	4.76	3.86	3.49	3.62	N/A	N/A	2.10 (s)
**Gal2**	4.60	3.76	3.74	4.21	N/A	N/A	-[b]
**GlcNH₂**	4.66	2.77	3.17	3.64	N/A	N/A	-[b]

a=Not assigned, b=Not applicable

**Signal**	**Proton**	**Carbon**
Aromatic (Naphthyl)	8.08-7.94 (m, 4H) 7.71-7.59 (m, 3H)	128.1-125.0
-**CH₂**Ph	5.34 (s)	66.7
-NH-**CH₂**- (linker)	3.21-3.19(m)	41.1
-O-**CH₂**- (linker)	3.84,3.60	74.2
**C-CH₂-C** (linker)	1.67-1.57 (m, 2H),1.53-1.48 (m, 2H), 1.44-1.39 (m, 2H)	28.7, 28.4,22.2

^{13}C NMR (D_2O, 150 MHz): δ (ppm)

	**Carbon (C1)**
**Glc**	101.7
**Gal1**	102.7
**GlcNAc1**	102.7
**Gal2**	102.5
**GlcNH$_2$**	104.6

ESI TOF-MS m/z calcd for $C_{49}H_{75}N_3O_{27}$ $[M+H]^+$: 1137.4588, found 1137.8803

15.9. Compound 17: Compound **17** was prepared from compound **16** (3 mg, 8.17 µmol). Fraction containing products were pooled and lyophilized to give **17** as a white fluffy solid (2.8 mg, 90%)

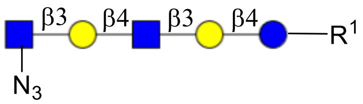

^{1}H NMR (D_2O, 600 MHz): δ (ppm)

	**H1**	**H2**	**H3**	**H4**	**H5**	**H6**	NH**Ac**
**Glc**	4.36	3.23	3.60	3.56	N/A	3.78	-[b]
**Gal1**	4.41	3.59	3.72	4.18(d, *J*=3.3 Hz)	N/A	N/A[a]	-[b]
**GlcNAc1**	4.71	3.82	3.49	3.58	N/A	N/A	2.04 (s)
**Gal2**	4.51	3.73	3.71	4.15 (d, *J*=3.3 Hz)	N/A	N/A	-[b]
**GlcN$_3$**	4.66	3.38	3.52	3.65	N/A	N/A	-[b]

a=Not assigned, b=Not applicable

**Signal**	**Proton**	**Carbon**
Aromatic (Naphthyl)	8.08-7.94 (m, 4H) 7.71-7.59 (m, 3H)	128.1-125.0
-**CH$_2$**Ph	5.28 (s)	66.8
-NH-**CH$_2$**- (linker)	3.21-3.19(m)	41.1
-O-**CH$_2$**- (linker)	3.84,3.60	74.2
**C-CH$_2$-C** (linker)	1.60-1.57 (m, 2H),1.53-1.48 (m, 2H), 1.34-1.32 (m, 2H)	28.7, 28.4,22.2

^{13}C NMR (D_2O, 150 MHz): δ (ppm)

	Carbon (C1)
**Glc**	102.1
**Gal1**	102.9
**GlcNAc1**	102.7
**Gal2**	102.9
**$GlcN_3$**	103.4

ESI TOF-MS m/z calcd for $C_{49}H_{73}N_5O_{27}$ $[M+Na]^+$: 1186.4391 found: 1185.88

15.10. Compound 18: Compound **18** was prepared from compound **16** (3 mg, 8.17 μmol). Fraction containing products were pooled and lyophilized to give **18** as a white fluffy solid (2.7 mg, 83%)

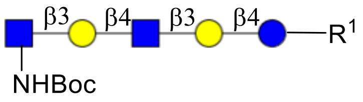

^1H NMR (D_2O, 600 MHz): δ (ppm)

	H1	H2	H3	H4	H5	H6	NH**Ac**
**Glc**	4.36	3.24	3.60	3.56	N/A	3.75	-[b]
**Gal1**	4.37	3.59	3.72	4.14(d, *J*=3.3 Hz)	N/A	N/A[a]	-[b]
**GlcNAc1**	4.70	3.82	3.49	3.58	N/A	N/A	2.04 (s)
**Gal2**	4.47	3.63	3.71	4.12 (d, *J*=3.3 Hz)	N/A	N/A	-[b]
**GlcNHBoc**	4.71	3.40	3.75	3.60	N/A	N/A	1.45 (s,9H) **$(CH_3)_3$ ^{t}Butyl**

a=Not assigned, b=Not applicable

**Signal**	**Proton**	**Carbon**
Aromatic (Naphthyl)	8.08-7.94 (m, 4H) 7.71-7.59 (m, 3H)	128.1-125.0
-**CH_2**Ph	5.25 (s)	66.8
-NH-**CH_2**- (linker)	3.21-3.19(m)	41.1
-O-**CH_2**- (linker)	3.84,3.60	74.2
**C-CH_2-C** (linker)	1.60-1.57 (m, 2H),1.53-1.48 (m, 2H), 1.34-1.32 (m, 2H)	28.7, 28.4,22.2

^{13}C NMR (D_2O, 150 MHz): δ (ppm)

	**Carbon (C1)**
**Glc**	102.1
**Gal1**	102.8
**GlcNAc1**	102.6
**Gal2**	102.7
**GlcNHBoc**	102.7

ESI TOF-MS m/z calcd for $C_{54}H_{83}N_3O_{29}$ $[M+Na]^+$: 1260.5010 found: 1259.8385

15.11. Compound 19: Compound **19** was prepared from compound **16** (3 mg, 8.17 μmol). Fraction containing products were pooled and lyophilized to give **19** as a white fluffy solid (2.9 mg, 94%)

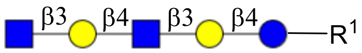

^{1}H NMR (D_2O, 600 MHz): δ (ppm)

	**H1**	**H2**	**H3**	**H4**	**H5**	**H6**	NH**Ac**
**Glc**	4.37	3.25	3.60	3.56	N/A	N/A[a]	-[b]
**Gal1**	4.37	3.59	3.72	4.15(d, *J*=3.3 Hz)	N/A	N/A	-[b]
**GlcNAc1**	4.70	3.82	3.7	3.58	N/A	N/A	2.05 (s)
**Gal2**	4.47	3.58	3.71	4.15 (d, *J*=3.3 Hz)	N/A	N/A	-[b]
**GlcNAc2**	4.73	3.76	3.75	3.60	N/A	N/A	2.05 (s)

a=Not assigned, b=Not applicable

**Signal**	**Proton**	**Carbon**
Aromatic (Naphthyl)	8.08-7.94 (m, 4H)<br>7.71-7.59 (m, 3H)	128.1-125.0
-**CH_2**Ph	5.25 (s)	66.8
-NH-**CH_2**- (linker)	3.21-3.19(m)	41.1
-O-**CH_2**- (linker)	3.84,3.60	74.2
**C-CH_2-C** (linker)	1.60-1.57 (m, 2H),1.53-1.48 (m, 2H), 1.34-1.32 (m, 2H)	28.7, 28.4,22.2

^{13}C NMR (D_2O, 150 MHz): δ (ppm)

	**Carbon (C1)**
**Glc**	102.1
**Gal1**	102.9
**GlcNAc1**	102.7
**Gal2**	102.9
**GlcNAc1**	102.7

ESI TOF-MS m/z calcd for $C_{51}H_{77}N_3O_{28}$ $[M+H]^+$: 1180.4772 found: 1179.8614

15.12. Compound 23: Compound **23** was prepared from compound **9** (4 mg, 6.13 μmol). Final product was purified using ultrafine P4 biogel with 100mM Ammonium bicarbonate (1.5 cm* 115 cm column) as eluant. Fraction containing products were pooled and lyophilized to give **23** as a white fluffy solid (4.9 mg, 46% overall yield after six automated reaction cycle)

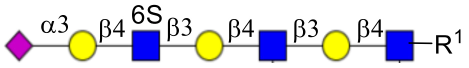

^{1}H NMR (D_2O, 600 MHz): δ (ppm)

	**H1**	**H2**	**H3**	**H4**	**H5**	**H6**	**H7**	**H8**	**H9**	NH**Ac**
**GlcNAc1**	4.43	3.70	3.65	3.71	N/A[a]	N/A	-[b]	-	-	2.05-1.95 (12H)
**Gal1**	4.45	3.59	3.73	4.16 (d, J=3.3 Hz)	N/A	N/A	-	-	-	-
**GlcNAc2**	4.71	3.82	3.74	3.74	N/A	N/A	-	-	-	2.05-1.95 (12H)
**Gal2**	4.48	3.57	3.73	4.20 (d, J=3.3 Hz)	N/A	N/A	-	-	-	-
**GlcNAc3 6S**	4.72	3.84	3.75	3.8	N/A	4.41,4.33	-	-	-	2.05-1.95 (12H)
**Gal3**	4.61	3.57	4.14 (dd, J = 9.8, 3.1 Hz)	3.97	N/A	N/A	-	-	-	-

**Neu5Ac**			2.76 (H_{eq}) (dd, *J* = 12.4, 4.6 Hz) 1.82 (H_{ax}) (t, *J* = 12.1)	3.65	3.84	N/A	N/A	N/A	N/A	2.05-1.95 (12H)

a=Not assigned, b=Not applicable

**Signal**	**Proton**	**Carbon**
Aromatic (Naphthyl)	8.01-7.91 (m, 4H)<br>7.63-7.55 (m, 3H)	128.1-125.0
-**CH_2**Ph	5.29 (s)	66.8
-NH-**CH_2**- (linker)	3.14-3.10(m)	41.1
-O-**CH_2**- (linker)	3.83,3.47	72.2
**C-CH_2-C** (linker)	1.55 -1.45 (m, 4H), 1.35-1.25 (m, 2H)	28.3,22.1

^{13}C NMR (D_2O, 150 MHz): δ (ppm)

	**Carbon (C1)**
**GlcNAc1**	100.9
**Gal1**	102.7
**GlcNAc2**	102.7
**Gal2**	102.7
**GlcNAc3 6S**	102.8
**Gal3**	102.2

ESI TOF-MS m/z calcd for $C_{70}H_{105}N_5O_{44}S$ $[M-2H]^{2-}$: 875.7932 found: 875.4670

15.13. Compound 26: Compound **26** was prepared from compound **9** (4 mg, 6.13 μmol). Final product was purified using ultrafine P4 biogel with 100mM Ammonium bicarbonate (1.5 cm * 115 cm column) as eluant. Fraction containing products were pooled and lyophilized to give **26** as a white fluffy solid (4.7 mg, 36% overall yield after eight automated reaction cycles).

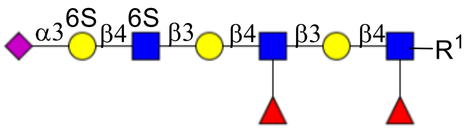

^{1}H NMR (D_2O, 600 MHz): δ (ppm)

	H1	H2	H3	H4	H5	H6	H7	H8	H9	NHAc
**GlcNAc1**	4.42	3.84	N/A	N/A	N/A[a]	N/A	-[b]	-	-	2.05-1.95 (12H)
**Gal1**	4.43	3.50	3.71	4.10	N/A	N/A	-	-	-	-
**GlcNAc2**	4.71	3.96	N/A	3.74	N/A	N/A	-	-	-	2.05-1.95 (12H)
**Gal2**	4.45	3.53	3.71	4.13	N/A	N/A	-	-	-	-
**GlcNAc3 6S**	4.71	3.82	3.75	3.8	N/A	4.45,4.31	-	-	-	2.05-1.95 (12H)
**Gal36S**	4.62	3.58	4.14	4.02	N/A	4.20	-	-	-	-
**Fuc1**	5.06	3.68	N/A	N/A	4.79	1.17-1.15 (6 H)	-	-	-	-
**Fuc2**	5.13	3.70	N/A	N/A	4.79	.17-1.15 (6 H)	-	-	-	-
**Neu5Ac**	-	-	2.76 (H_{eq}) (dd, J = 12.4, 4.6 Hz) 1.82 (H_{ax}) (t, J = 12.1)	3.68	3.85	N/A	N/A	N/A	N/A	2.05-1.95 (12H)

a=Not assigned, b=Not applicable

**Signal**	**Proton**	**Carbon**
Aromatic (Naphthyl)	8.01-7.91 (m, 4H)<br>7.63-7.55 (m, 3H)	128.1-125.0

-**CH_2**Ph	5.29 (s)	66.8
-NH-**CH_2**- (linker)	3.14-3.10(m)	41.1
-O-**CH_2**- (linker)	3.83,3.47	72.2
**C-CH_2-C** (linker)	1.55 -1.45 (m, 4H), 1.35-1.25 (m, 2H)	28.3,22.1

^{13}C NMR (D_2O, 150 MHz): δ (ppm)

	**Carbon (C1)**
**GlcNAc1**	100.8
**Gal1**	101.6
**GlcNAc2**	102.5
**Gal2**	101.6
**GlcNAc3 6S**	102.5
**Gal36S**	102.2
**Fuc1**	98.5
**Fuc2**	98.5

ESI TOF-MS m/z calcd for $C_{82}H_{124}N_5O_{55}S$_2 $[M-2H]^{2-}$: 1061.3259 found: 1061.4405

15.14. Compound 27: Compound **27** was prepared according to previous protocol. HPLC purification and desalting gave **27** as a white fluffy solid.

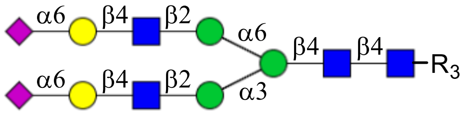

^{1}H NMR (D_2O, 600 MHz): δ (ppm)

	H1	H2	H3	H4	H5	H6	NH**Ac**
**GlcNAc1**	4.97	3.81	3.74	3.66	N/A	N/A[a]	2.02-1.9 18H
**GlcNAc2**	4.58	3.77	3.74	N/A	N/A	N/A	2.02-1.9 18H
**GlcNAc3**	4.62	3.76	3.58	3.48	N/A	N/A	2.02-1.9 18H
**GlcNAc4**	4.62	3.76	3.58	3.52	N/A	N/A	2.02-1.9 18H
**Man1**	4.78	4.27	3.78	3.65	3.54	N/A	-[b]
**Man2**	5.14	4.20	3.93	3.58	3.76	N/A	-
**Man3**	4.96	4.13	3.91	3.58	3.64	N/A	-
**Gal1**	4.45	3.54	3.67	3.94	N/A	N/A	-
**Gal2**	4.45	3.54	3.67	3.94	N/A	N/A	-
**Neu5Ac1**	-	-	2.69 – H_{eq} (dd, J = 12.4, 4.7 Hz, 1H) 1.75 – H_{ax} (t, J = 12.3 Hz, 1H)	3.68	3.82		2.02-1.9 18H
**Neu5Ac2**	-	-	2.69 – H_{eq} (dd, J = 12.4, 4.7 Hz, 1H) 1.75 – H_{ax} (t, J = 12.3 Hz, 1H)	3.68	3.82		2.02-1.9 18H

a=Not assigned, b=Not applicable

**Signal**	**Proton**	**Carbon**
Aromatic (Naphthyl)	8.03-7.94 (m, 4H) 7.64-7.52 (m, 3H)	128.1-125.3
-**CH_2**Ph	5.37 (d, J = 12.7 Hz) 5.26 (d, J = 12.7 Hz)	67.0

-NH-**CH**-COOH	4.55 (dd, J = 9.6, 4.1 Hz)	51.4
-C(O)-**CH**$_2$-CH	2.87 (dd, J = 15.3, 4.2 Hz)<br>2.73 (dd, J = 15.2, 9.6 Hz)	38.1

^{13}C NMR (D_2O, 150 MHz): δ (ppm)

	**Carbon (C1)**
**GlcNAc 1**	78.06
**GlcNAc 2**	101.1
**GlcNAc 3**	99.1
**GlcNAc 4**	99.1
**Man1**	100.3
**Man2**	99.3
**Man3**	96.7
**Gal1**	103.3
**Gal2**	103.3

ESI TOF-MS m/z calcd for $C_{100}H_{150}N_8O_{66}$ $[M-2H]^{2-}$: 1259.9336 found: 1259.5420

15.15. Compound 28: Compound **28** was prepared according to Table S13 automation program from compound **27** (30 mg, 12 µmol). Fraction containing products were pooled and lyophilized to give **12** as a white fluffy solid (14.2 mg, 97%).

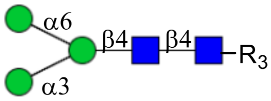

This reaction also worked as a proof of concept for scalability of the reaction on the recently developed automation program as this current setup can easily handle up to 30 mg scale (1-50 µmol) chemoenzymatic reaction. Compound **27** is used as the common precursor for asymmetrically branched N-Glycan synthesis.

^{1}H NMR (D_2O, 600 MHz): δ (ppm)

	**H1**	**H2**	**H3**	**H4**	**H5**	**H6**	NH**Ac**
**GlcNAc1**	4.93	3.85	3.74	3.63	3.56	N/A[a]	2.02-1.9 6H
**GlcNAc2**	4.57	3.80	3.73	3.72	3.59	N/A	2.02-1.9 6H
**Man1**	4.79	4.27	3.78	3.65	3.54	N/A	-[b]
**Man2**	5.11	4.08	3.93	3.58	3.76	N/A	-
**Man3**	4.93	3.99	3.91	3.58	3.64	N/A	-

a=Not assigned, b=Not applicable

**Signal**	**Proton**	**Carbon**
Aromatic (Naphthyl)	8.03-7.94 (m, 4H)<br>7.64-7.52 (m, 3H)	128.1-125.3
-**CH_2**Ph	5.37 (d, J = 12.7 Hz)<br>5.26 (d, J = 12.7 Hz)	67.0
-NH-**CH**-COOH	4.55 (dd, J = 9.6, 4.1 Hz)	51.4
-C(O)-**CH_2**-CH	2.87 (dd, J = 15.3, 4.2 Hz)<br>2.73 (dd, J = 15.2, 9.6 Hz)	38.1

^{13}C NMR (D_2O, 150 MHz): δ (ppm)

	**Carbon (C1)**
**GlcNAc 1**	76.1
**GlcNAc 2**	99.2
**Man1**	100.5
**Man2**	99.3
**Man3**	96.7

ESI TOF-MS m/z calcd for $C_{50}H_{72}N_4O_{30}$ $[M-H]^-$: 1207.4167 found: 1207.0556

15.16. Compound 34: Compound **34** was prepared from compound **27** (5 mg, 4.14 μmol). Final product was purified using ultrafine P4 biogel with 100mM Ammonium bicarbonate (1.5 cm * 115 cm column) as eluant. Fraction containing products were pooled and lyophilized to give **34** as a white fluffy solid after 11 consecutive automated reaction cycle (5.1 mg, 38% overall yield).

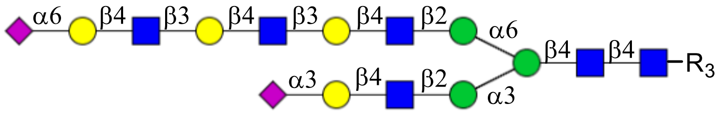

^{1}H NMR (D_2O, 600 MHz): δ (ppm)

	H1	H2	H3	H4	H5	H6	NHAc
**GlcNAc1**	4.92	3.76	3.74	3.66	N/A	N/A[a]	2.08-1.82 24H
**GlcNAc2**	4.60-4.54 (m, 3H)	3.80	3.76	N/A	N/A	N/A	2.08-1.82 24H
**Man1**	4.78	4.27	3.78	3.65	N/A	N/A	-[b]
**Man2**	5.12	4.20	3.93	3.58	3.76	N/A	-
**Man3**	4.93	4.12	3.91	3.58	3.64	N/A	-
**GlcNAc3**	4.60-4.54 (m, 3H)	3.76	3.58	3.48	N/A	N/A	2.08-1.82 24H
**GlcNAc4**	4.60-4.54 (m, 3H)	3.76	3.58	3.52	N/A	N/A	2.08-1.82 24H
**Gal1**	4.56-4.46 (4H)	3.60	4.12	4.17	N/A	N/A	-
**Gal2**	4.56-4.46 (4H)	3.56	3.73	4.17	N/A	N/A	-
**GlcNAc5**	4.74-4.70 (2H)	3.76	3.58	3.48	N/A	N/A	2.08-1.82 24H
**GlcNAc6**	4.74-4.70 (2H)	3.76	3.58	3.52	N/A	N/A	2.08-1.82 24H

**Gal3**	4.56-4.46 (4H)	3.56	3.73	4.17	N/A	N/A	-
**Gal4**	4.56-4.46 (4H)	3.56	3.73	4.17	N/A	N/A	-
**Neu5Ac1(α3)**	-	-	1.80 H_{ax} 2.77-H_{eq} (dd, J = 12.5, 4.5 Hz, 1H)	3.69	N3.86	N/A	2.08-1.82 24H
**Neu5Ac2(α6)**	-	-	2.66 – H_{eq} (dd, J = 12.5, 4.5 Hz, 1H) 1.73 – H_{ax} (t, J = 12.1 Hz, 1H)	3.64	3.81	N/A	2.08-1.82 24H

a=Not assigned, b=Not applicable

**Signal**	**Proton**	**Carbon**
Aromatic (Naphthyl)	8.03-7.94 (m, 4H)<br>7.64-7.52 (m, 3H)	128.1-125.3
-**CH_2**Ph	5.40 (d, J = 12.7 Hz)<br>5.21(d, J = 12.7 Hz)	67.0
-NH-**CH**-COOH	4.55 (dd, J = 9.6, 4.1 Hz)	51.4
-C(O)-**CH_2**-CH	2.86 (dd, J = 15.3, 4.2 Hz)<br>2.56 (dd, J = 15.2, 9.6 Hz)	38.1

^{13}C NMR (D_2O, 150 MHz): δ (ppm)

	**Carbon (C1)**
**GlcNAc1**	78.06
**GlcNAc2**	101.3
**GlcNAc3**	99.6
**GlcNAc4**	99.6
**GlcNAc5**	102.5
**GlcNAc6**	102.5
**Man1**	100.3
**Man2**	99.5
**Man3**	96.9
**Gal1**	103.2
**Gal2**	103.2
**Gal3**	103.2

Gal4	102.5

ESI TOF-MS m/z calcd for $C_{116}H_{188}N_{10}O_{84}$ $[M-3H]^{3-}$: 1083.0414 found: 1082.7105

15.17. Compound 44: Compound **44** was prepared from compound **27** (5 mg, 4.14 μmol). Final product was purified using ultrafine P4 biogel with 100mM Ammonium bicarbonate (1.5 cm * 115 cm column) as eluant. Fraction containing products were pooled and lyophilized to give **44** as a white fluffy solid after 17 consecutive automated reaction cycle (1.25 mg, 8.5% overall yield).

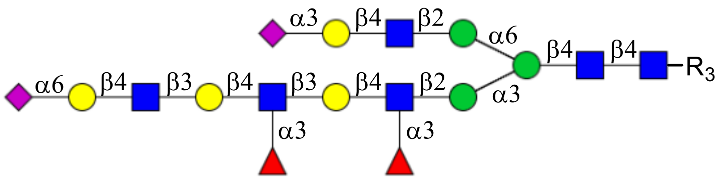

^{1}H NMR (D_2O, 600 MHz): δ (ppm)

	H1	H2	H3	H4	H5	H6	NH**Ac**
**GlcNAc1**	4.92	3.76	N/A	N/A	N/A	N/A[a]	2.12-1.86 24H
**GlcNAc2**	4.60-4.54 (m, 3H)	3.91	N/A	N/A	N/A	N/A	2.12-1.86 24H
**Man1**	4.78	4.27	3.78	N/A	N/A	N/A	-[b]
**Man2**	5.12	4.20	3.93	N/A	N/A	N/A	-
**Man3**	4.93	4.12	3.91	N/A	N/A	N/A	-
**GlcNAc3**	4.60-4.54 (m, 3H)	3.77	N/A	N/A	N/A	N/A	2.12-1.86 24H
**GlcNAc4**	4.60-4.54 (m, 3H)	3.76	N/A	N/A	N/A	N/A	2.12-1.86 24H
**Gal1**	4.56-4.46 (4H)	3.54	3.67	4.10	N/A	N/A	-
**Gal2**	4.56-4.46 (4H)	3.54	4.12	4.12	N/A	N/A	-

**GlcNAc5**	4.74-4.70 (2H)	3.92	N/A	N/A	N/A	N/A	2.12-1.86 24H
**GlcNAc6**	4.74-4.70 (2H)	3.81	N/A	N/A	N/A	N/A	2.12-1.86 24H
**Gal3**	4.56-4.46 (4H)	3.54	3.67	4.10	N/A	N/A	-
**Gal4**	4.56-4.46 (4H)	3.54	3.67	4.10	N/A	N/A	-
**Fuc1**	5.13	3.70	3.89	N/A	4.83	1.15 (**CH_3**)	-
**Fuc2**	5.13	3.70	3.89	N/A	4.83	1.15 (**CH_3**)	-
**Neu5Ac1(α3)**	-	-	1.81 H_{ax} (t, J = 12.3 Hz, 1H) 2.77-H_{eq} (dd, J = 12.5, 4.5 Hz, 1H)	3.67	3.96	N/A	2.12-1.86 24H
**Neu5Ac2(α6)**	-	-	2.68 – H_{eq} (dd, J = 12.4, 4.7 Hz, 1H) 1.73 – H_{ax} (t, J = 12.3 Hz, 1H)	3.71	3.93	N/A	2.12-1.86 24H

a=Not assigned, b=Not applicable

**Signal**	**Proton**	**Carbon**
Aromatic (Naphthyl)	8.03-7.94 (m, 4H)<br>7.64-7.52 (m, 3H)	128.1-125.3
-**CH_2**Ph	5.37 (d, J = 12.7 Hz)<br>5.26 (d, J = 12.7 Hz)	67.0
-NH-**CH**-COOH	4.55 (dd, J = 9.6, 4.1 Hz)	51.4
-C(O)-**CH_2**-CH	2.87 (dd, J = 15.3, 4.2 Hz)<br>2.73 (dd, J = 15.2, 9.6 Hz)	38.1

^{13}C NMR (D_2O, 150 MHz): δ (ppm)

	**Carbon (C1)**

**GlcNAc1**	78.06
**GlcNAc2**	100.2
**GlcNAc3**	99.3
**GlcNAc4**	99.3
**GlcNAc5**	102.5
**GlcNAc6**	102.5
**Man1**	100.3
**Man2**	99.5
**Man3**	97.1
**Gal1**	102.1
**Gal2**	102.1
**Gal3**	103.6
**Gal4**	102.7
**Fuc1**	98.7
**Fuc2**	98.7

ESI TOF-MS m/z calcd for $C_{140}H_{215}N_{10}O_{94}$ $[M-3H]^{3-}$: 1180.4134 found: 1179.9648

15.17. Compound 52: Compound **52** was prepared from compound **27** (5 mg, 4.14 μmol). Final product was purified using ultrafine P4 biogel with 100mM Ammonium bicarbonate (1.5 cm * 115 cm column) as eluant. Fraction containing products were pooled and lyophilized to give **52** as a white fluffy solid after 17 consecutive automated reaction cycles (2.5 mg, 20% overall yield).

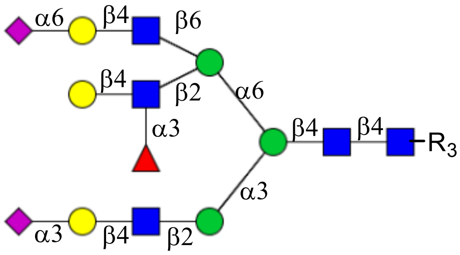

^{1}H NMR (D_2O, 600 MHz): δ (ppm)

	H1	H2	H3	H4	H5	H6	NH**Ac**
**GlcNAc1**	4.92	3.76	N/A	N/A	N/A	N/A[a]	2.12-1.98 21H
**GlcNAc2**	4.58	3.81	N/A	N/A	N/A	N/A	2.12-1.98 21H
**Man1**	4.79	4.27	3.78	N/A	N/A	N/A	-[b]
**Man2**	5.12	4.20	3.93	N/A	N/A	N/A	-
**Man3**	4.89	4.12	3.91	N/A	N/A	N/A	-

**GlcNAc3**	4.59	3.78	N/A	N/A	N/A	N/A	2.12-1.98 21H
**GlcNAc4**	4.59	3.78	N/A	N/A	N/A	N/A	2.12-1.98 21H
**GlcNAc5**	4.58	3.78	N/A	N/A	N/A	N/A	2.12-1.98 21H
**Gal1**	4.55-4.45 (3H)	3.54	N/A	3.92	N/A	N/A	-
**Gal2**	4.55-4.45 (3H)	3.54	3.67	3.92	N/A	N/A	-
**Gal3**	4.55-4.45 (3H)	3.53	3.66	3.92	N/A	N/A	-
**Fuc**	5.14 (1H)	3.62	3.72	3.99	4.85	1.18(**CH_3**)	-
**Neu5Ac1(α3)**	-	-	2.77 – H_{eq} (dd, J = 12.3, 4.7 Hz, 1H) 1.81 – H_{ax} (t, J = 12.3 Hz, 1H)	3.66	N/A	N/A	2.12-1.98 21H
**Neu5Ac2(α6)**	-	-	2.68 – H_{eq} (dd, J = 12.1, 4.7 Hz, 1H) 1.72 – H_{ax} (t, J = 12.2 Hz, 1H)	3.64	3.83	N/A	2.12-1.98 21H

a=Not assigned, b=Not applicable

**Signal**	**Proton**	**Carbon**
Aromatic (Naphthyl)	8.03-7.94 (m, 4H)<br>7.64-7.52 (m, 3H)	128.1-125.3
-**CH_2**Ph	5.37 (d, J = 12.7 Hz)<br>5.26 (d, J = 12.7 Hz)	67.0
-NH-**CH**-COOH	4.55 (dd, J = 9.6, 4.1 Hz)	51.4
-C(O)-**CH_2**-CH	2.87 (dd, J = 15.3, 4.2 Hz)	38.1

	2.73 (dd, J = 15.2, 9.6 Hz)	

^{13}C NMR (D_2O, 150 MHz): δ (ppm)

	Carbon (C1)
**GlcNAc1**	78.06
**GlcNAc2**	101.2
**GlcNAc3**	99.7
**GlcNAc4**	99.7
**GlcNAc5**	101.6
**Man1**	100.4
**Man2**	98.7
**Man3**	97.0
**Gal1**	102.7
**Gal2**	102.7
**Gal3**	103.5
**Fuc**	98.6

ESI TOF-MS m/z calcd for $C_{120}H_{182}N_9O_{80}$ $[M-3H]^{3-}$: 1010.0166 found: 1009.6372

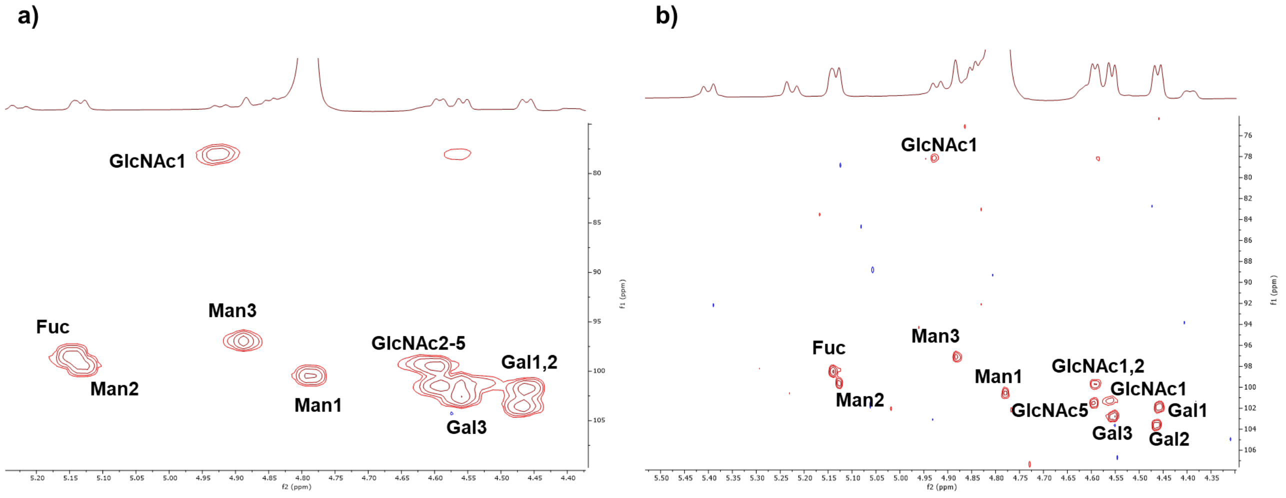

Figure S12. Zoomed in anomeric region in ^1H-^{13}C HSQC NMR of compound **52** in (a) 600 MHz (b) 1.1 GHz NMR.

16. General procedure for removal of 2-naphthylmethyl carbamate (Nap)-tag

To a stirring solution of the Nap-tagged saccharide in 1:1 ^tBuOH:Water mixture (20 mM) was added 10% wt/wt Pd/C under H_2 atmosphere. Upon completion of reaction as adjudged by LC-MS trace, the reaction mixture was filtered using a 0.2 μm PTFE syringe filter and the filtrate was lyophilized to give a saccharide with free amine group in

quantitative yield. For example, Nap tag of compound **34** was deprotected to give compound **SI2** (Scheme S1).

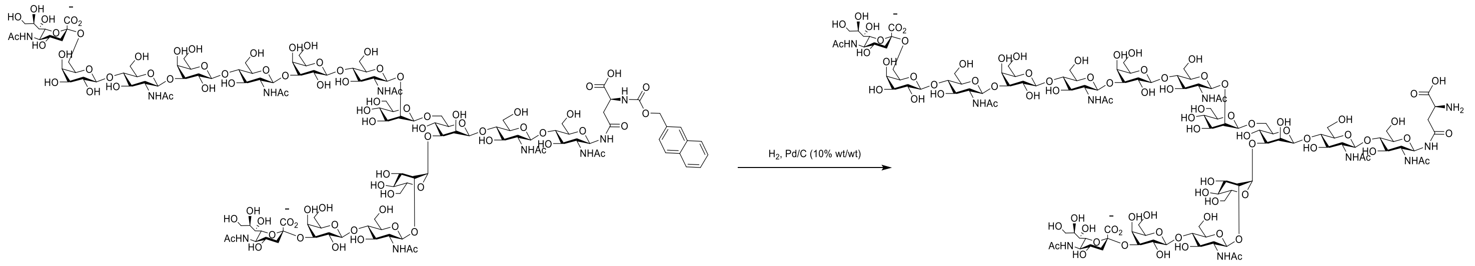

Scheme S1. Removal of Nap-tag by hydrogenation.

17. References

(1) Meng, L.; Forouhar, F.; Thieker, D.; Gao, Z.; Ramiah, A.; Moniz, H.; Xiang, Y.; Seetharaman, J.; Milaninia, S.; Su, M.; Bridger, R.; Veillon, L.; Azadi, P.; Kornhaber, G.; Wells, L.; Montelione, G. T.; Woods, R. J.; Tong, L.; Moremen, K. W. Enzymatic basis for *N*-glycan sialylation: structure of rat alpha2,6-sialyltransferase (ST6GAL1) reveals conserved and unique features for glycan sialylation. *J. Biol. Chem*. **2013**, *288*, 34680-34698.

(2) Kadirvelraj, R.; Yang, J. Y.; Sanders, J. H.; Liu, L.; Ramiah, A.; Prabhakar, P. K.; Boons, G. J.; Wood, Z. A.; Moremen, K. W. Human *N*-acetylglucosaminyltransferase II substrate recognition uses a modular architecture that includes a convergent exosite. *Proc. Natl. Acad. Sci. U. S. A.* **2018**, *115*, 4637-4642.

(3) Prudden, A. R.; Liu, L.; Capicciotti, C. J.; Wolfert, M. A.; Wang, S.; Gao, Z.; Meng, L.; Moremen, K. W.; Boons, G. J. Synthesis of asymmetrical multiantennary human milk oligosaccharides. *Proc. Natl. Acad. Sci. U. S. A.* **2017**, *114*, 6954-6959.

(4) Liu, L.; Prudden, A. R.; Capicciotti, C. J.; Bosman, G. P.; Yang, J. Y.; Chapla, D. G.; Moremen, K. W.; Boons, G. J. Streamlining the chemoenzymatic synthesis of complex *N*-glycans by a stop and go strategy. *Nat. Chem*. **2019**, *11*, 161-169.

(5) Liu, L.; Prudden, A. R.; Bosman, G. P.; Boons, G. J. Improved isolation and characterization procedure of sialylglycopeptide from egg yolk powder. *Carbohydr. Res*. **2017**, *452*, 122-128.

18. Copies of NMR spectra

^{1}H NMR of Compound **3**

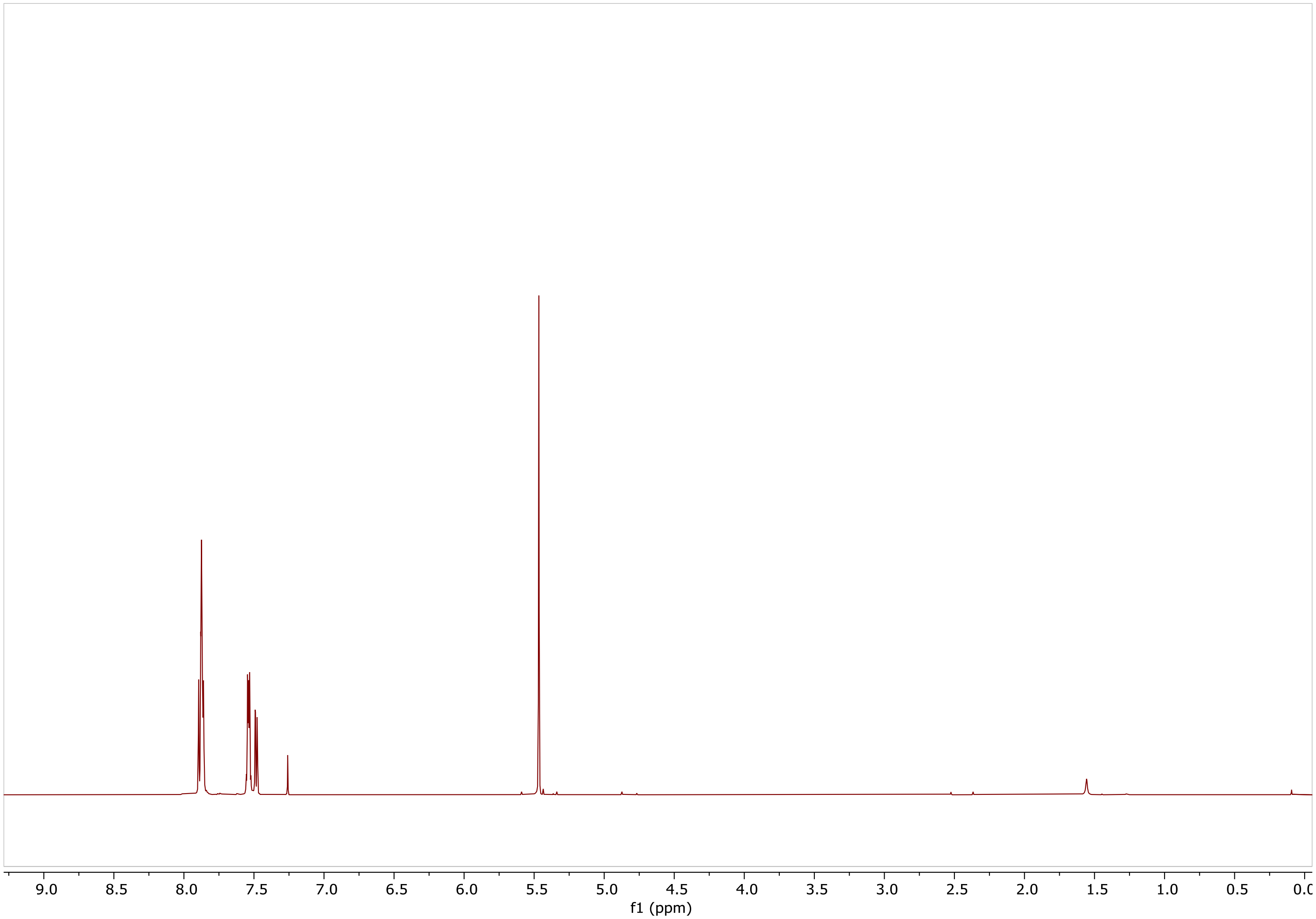

^{13}C NMR of Compound **3**

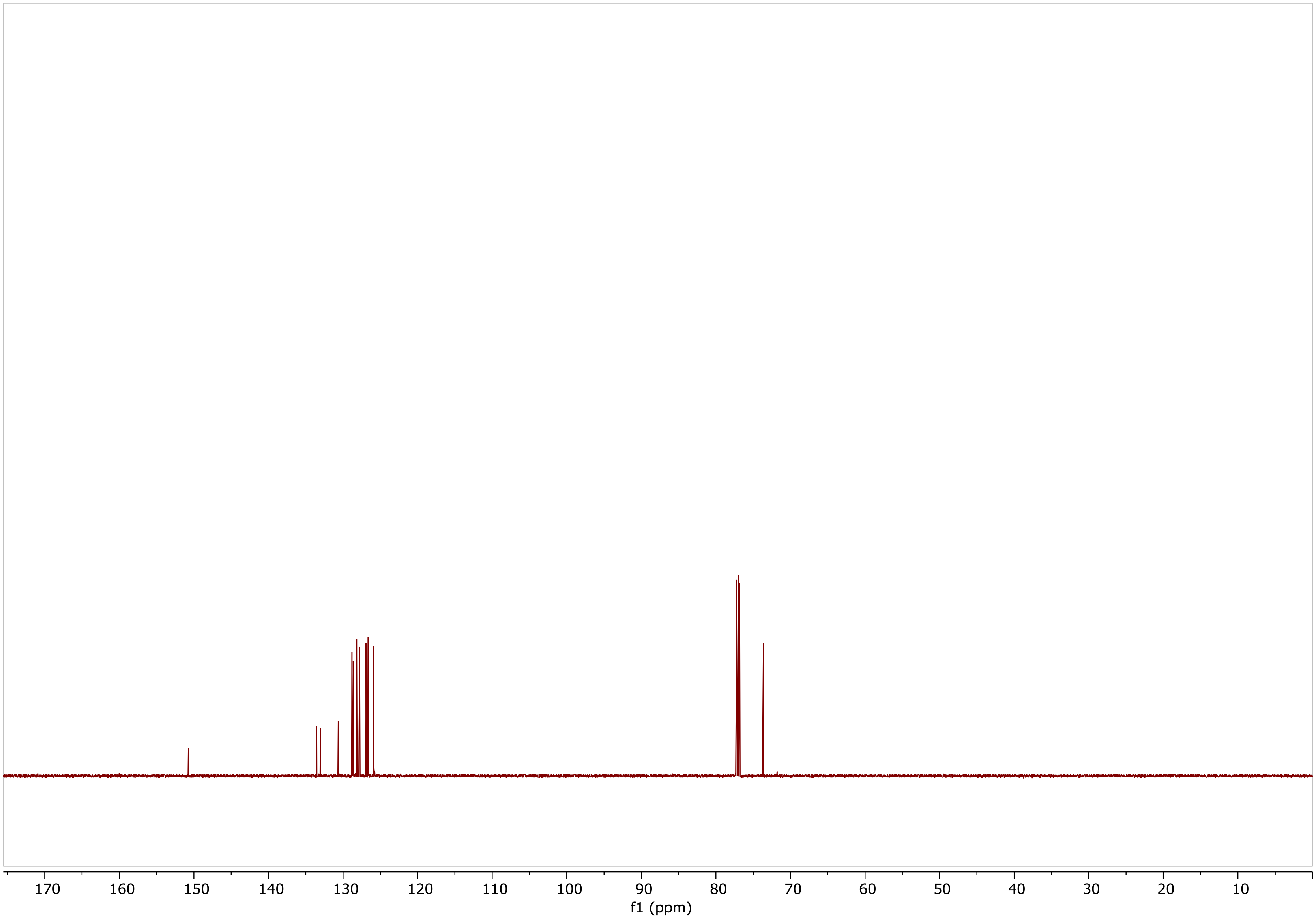

^{1}H NMR of Compound **6**

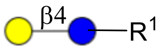

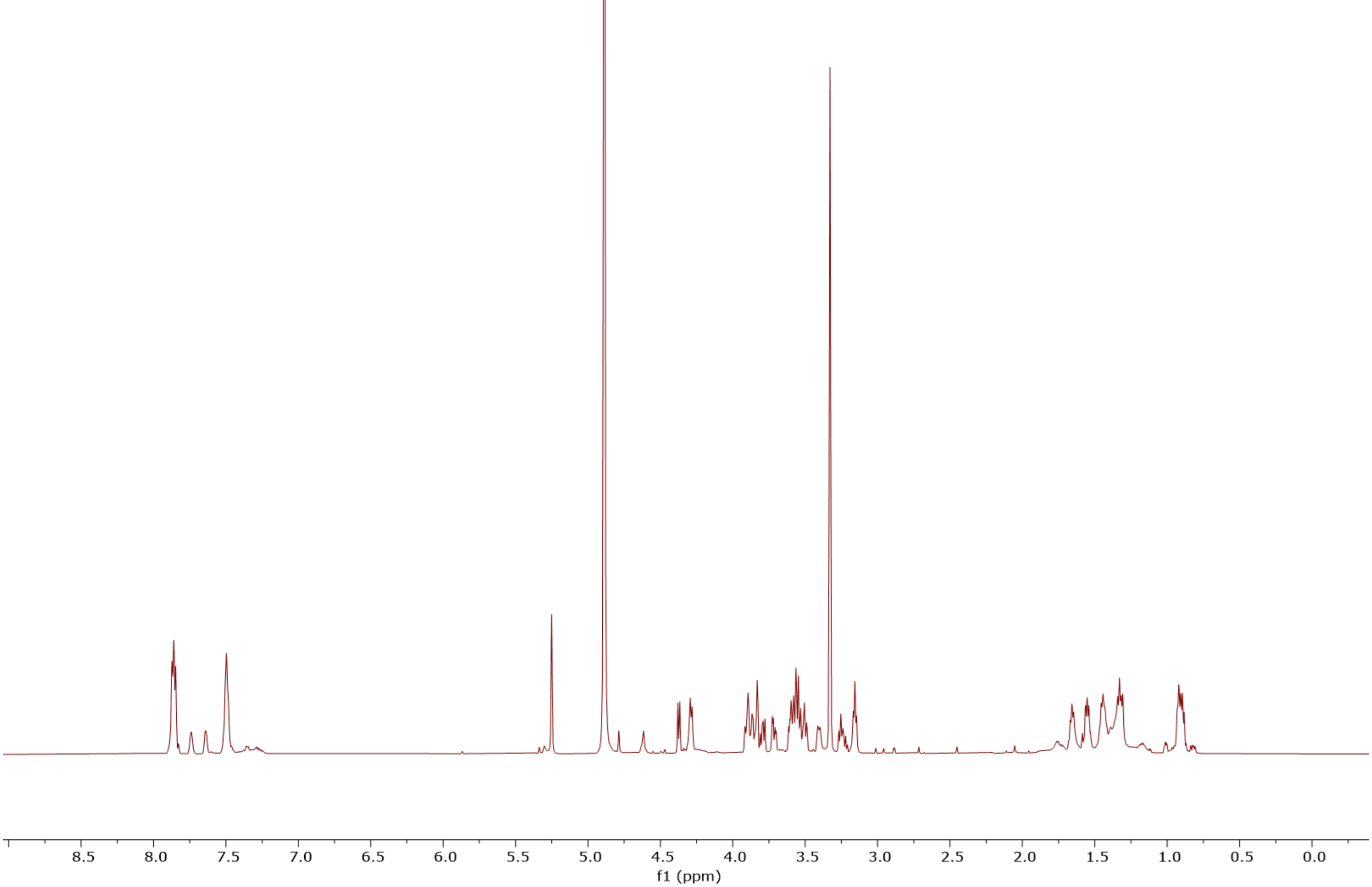

^{13}C NMR of Compound **6**

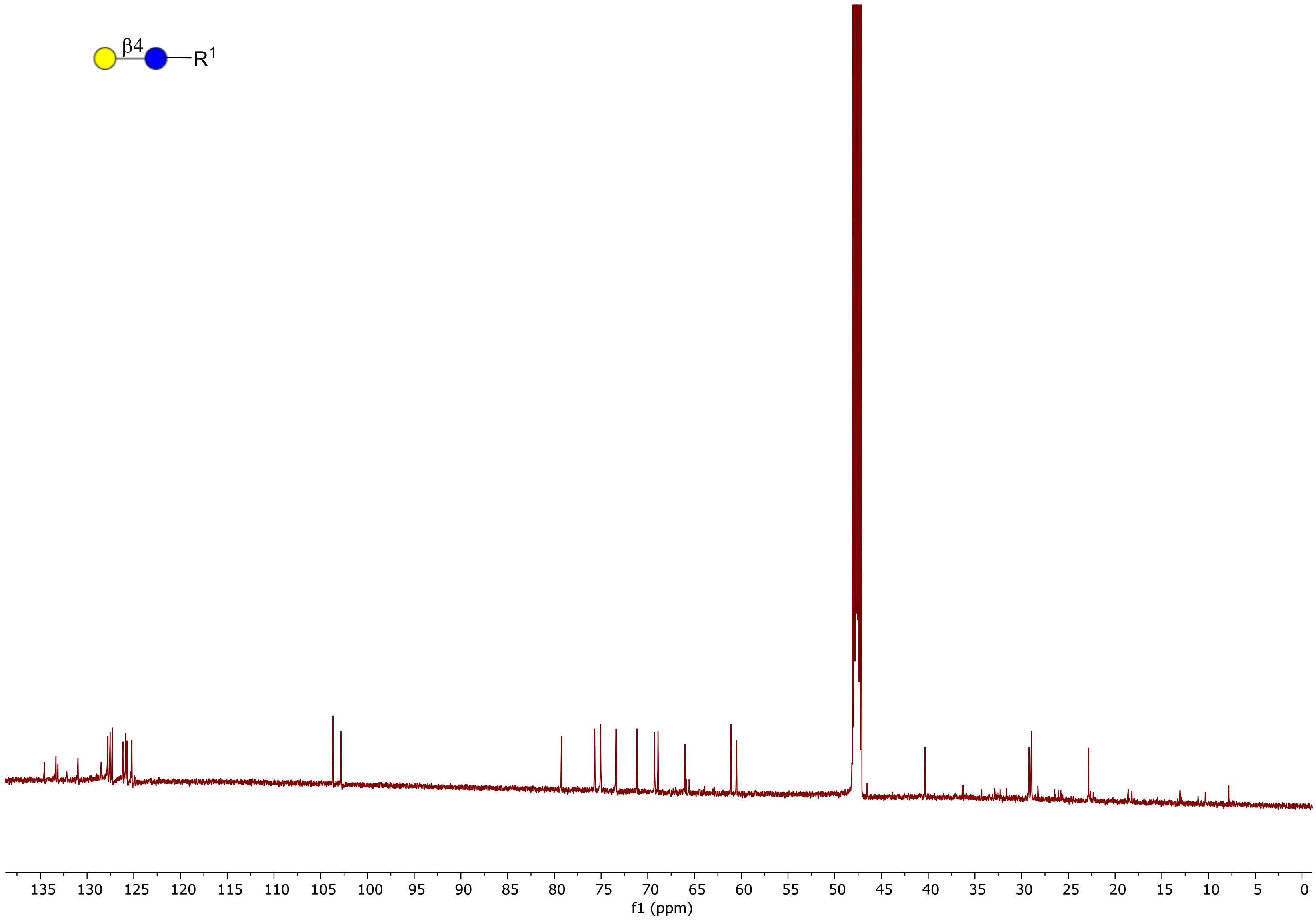

gCOSY NMR of Compound **6**

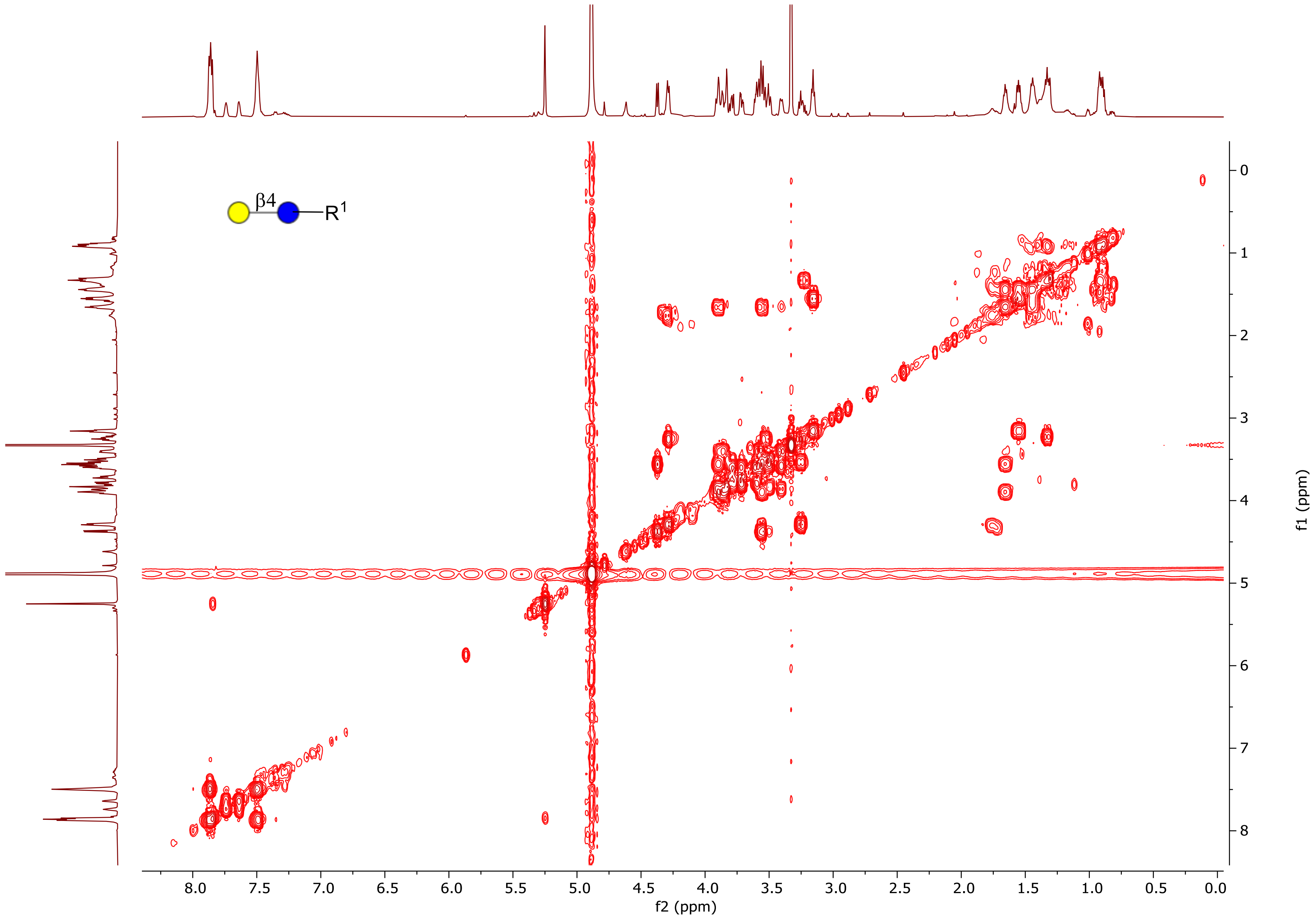

Multiplicity edited gHSQC NMR of Compound **6**

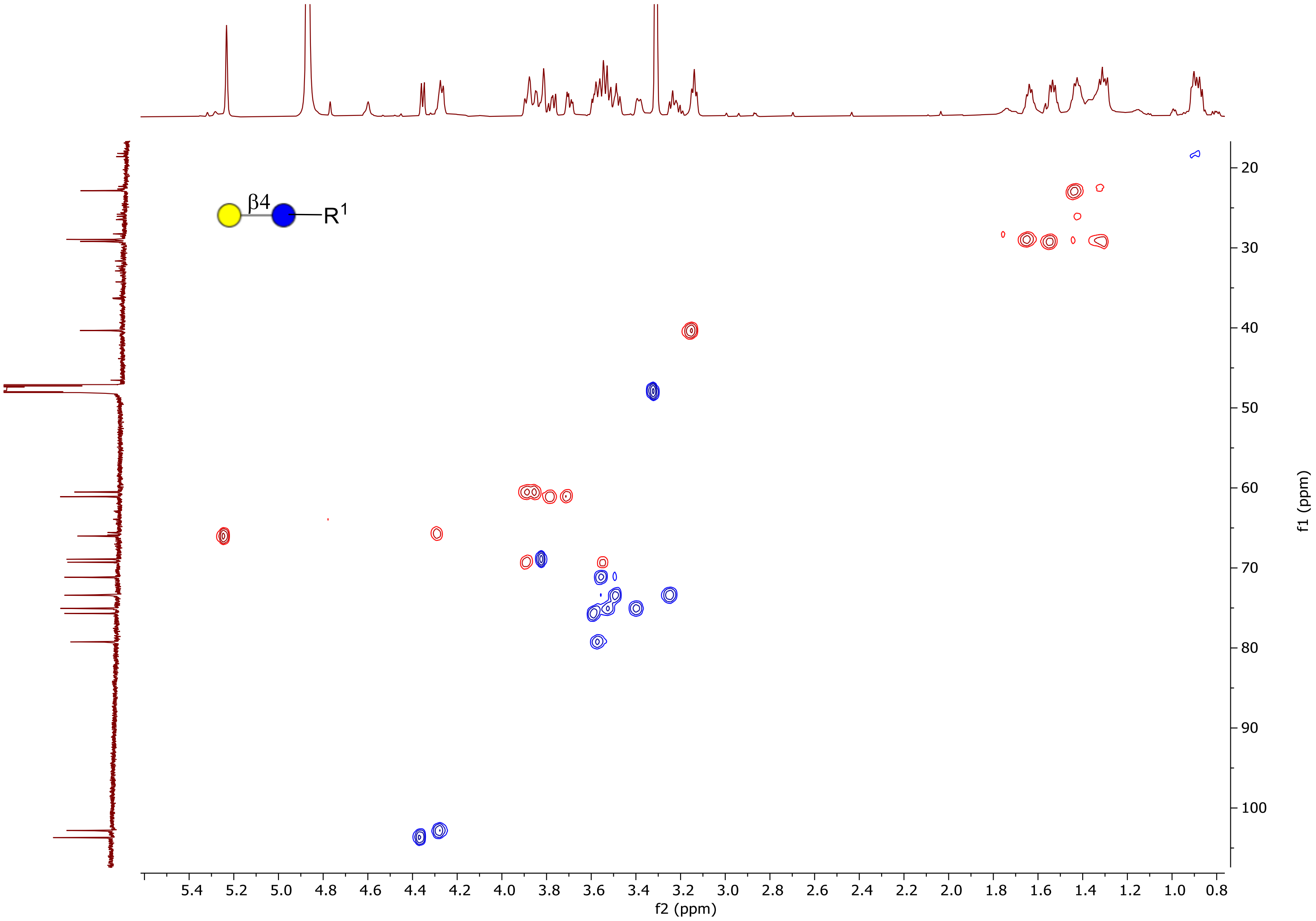

^{1}H NMR of Compound **7**

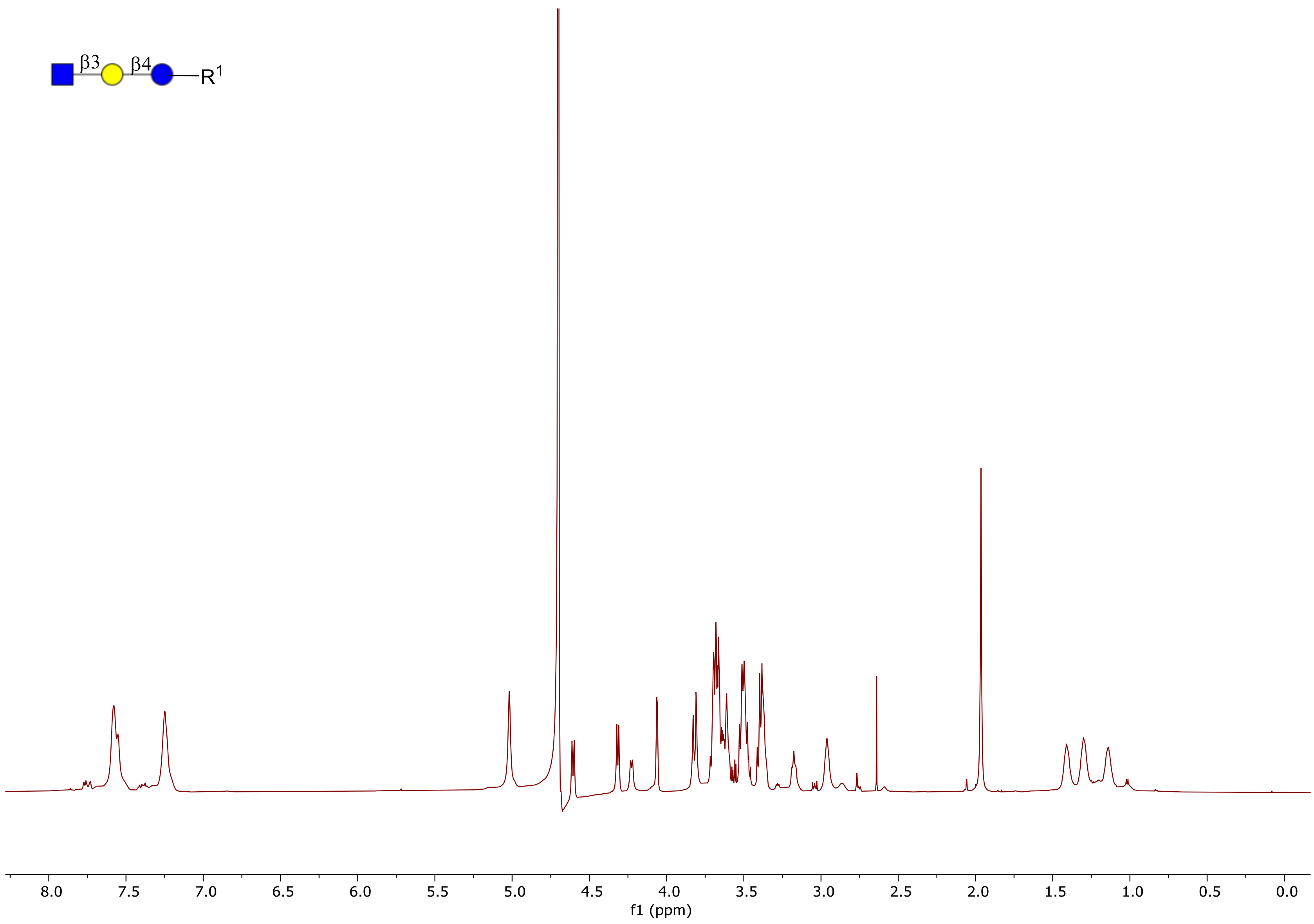

gCOSY NMR of Compound **7**

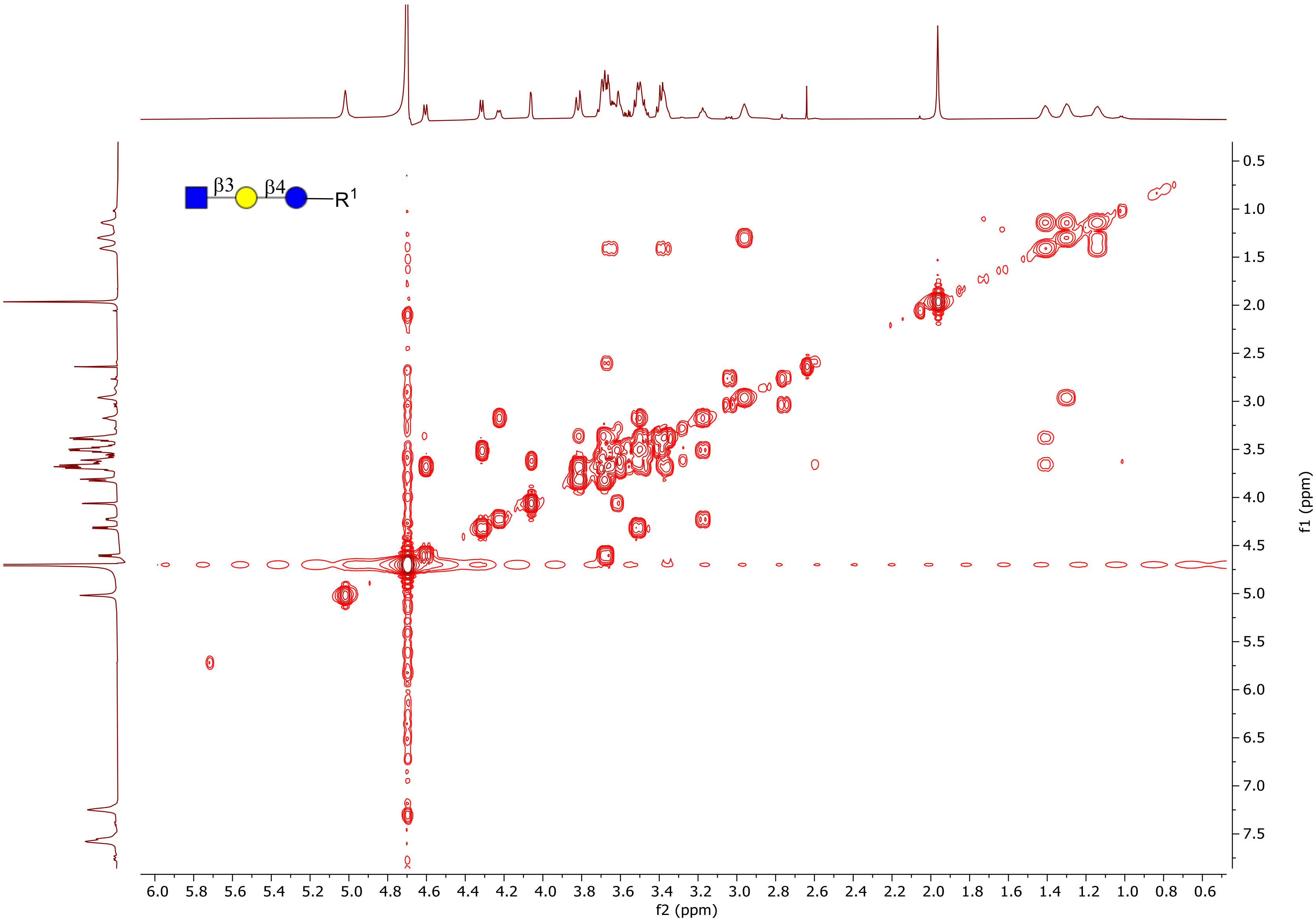

Multiplicity edited gHSQC NMR of Compound **7**

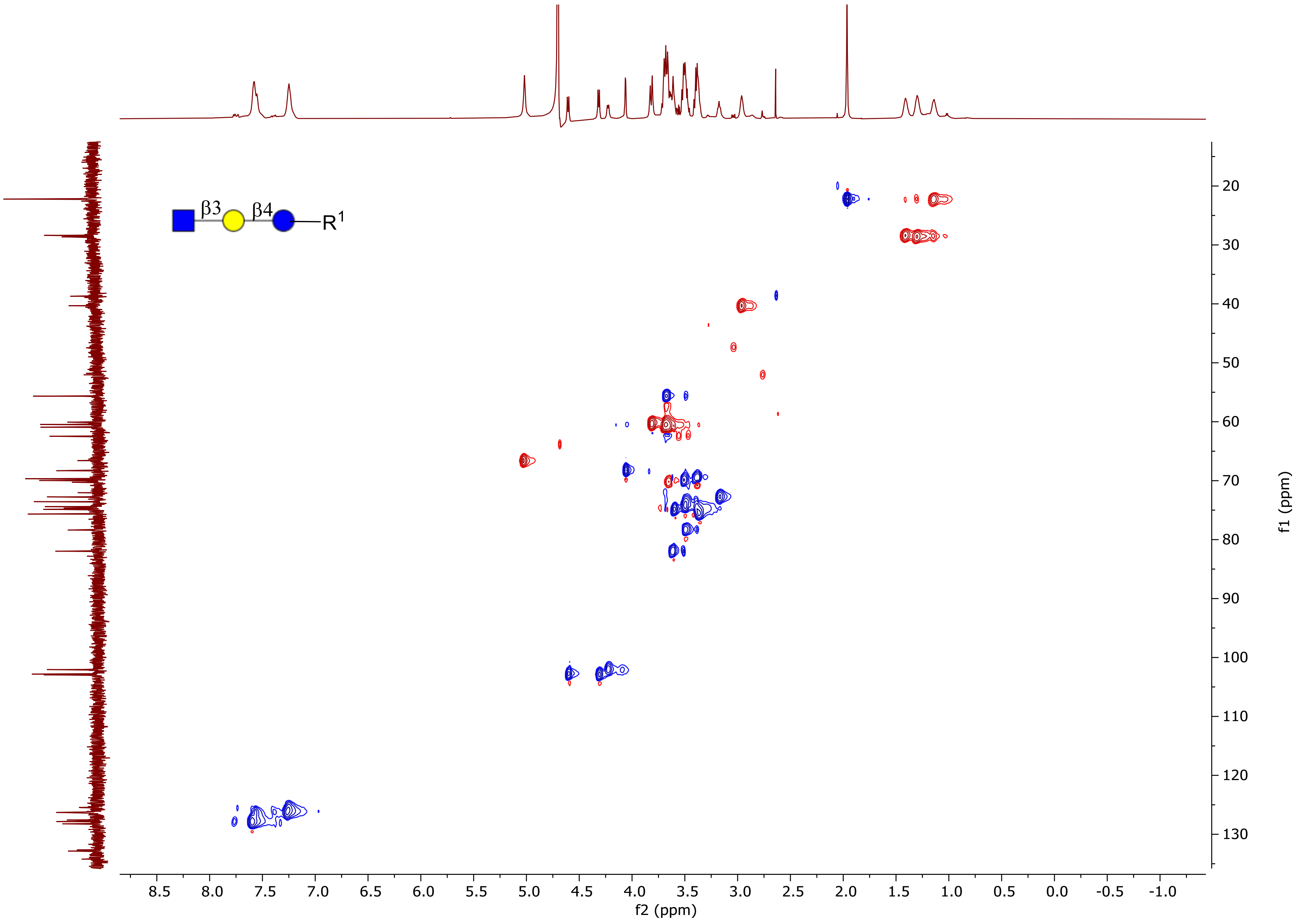

^{1}H NMR of Compound **9**

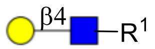

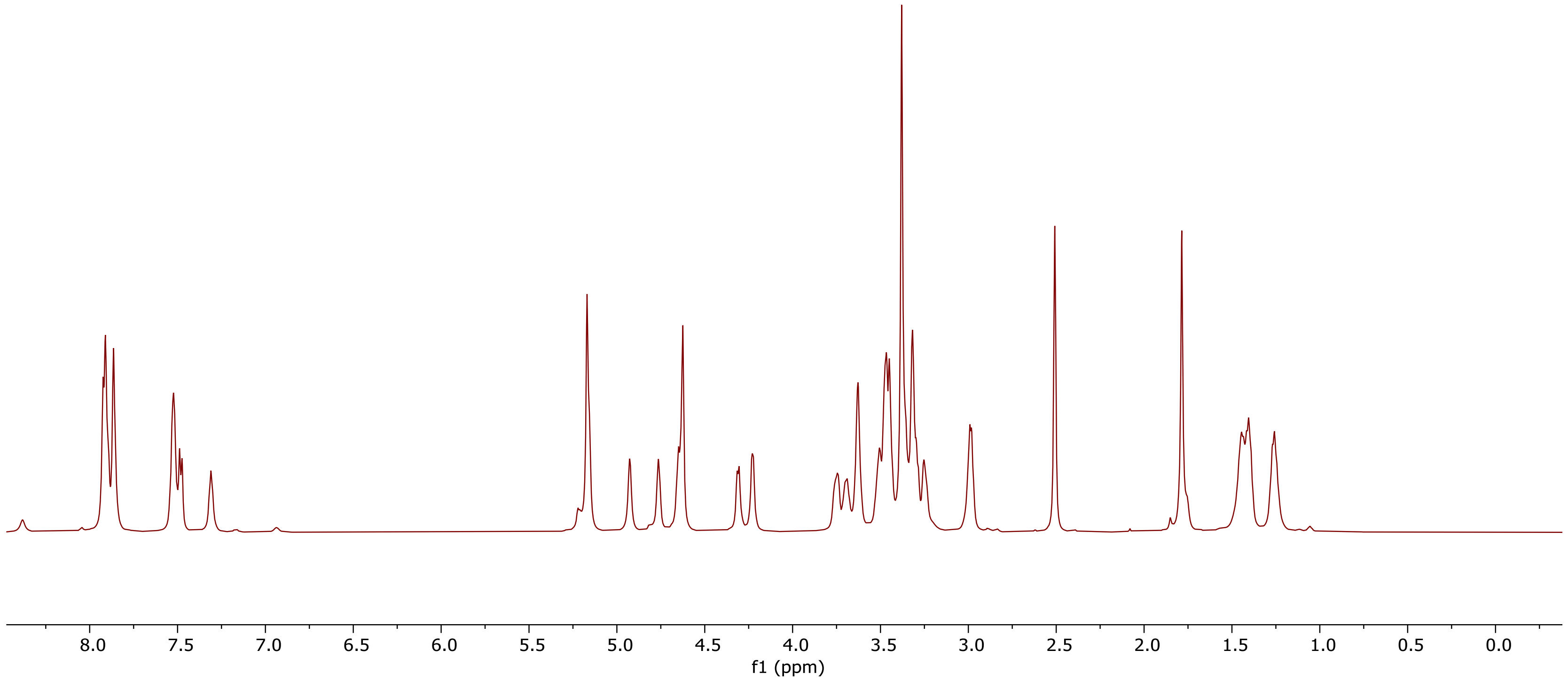

gCOSY NMR of Compound **9**

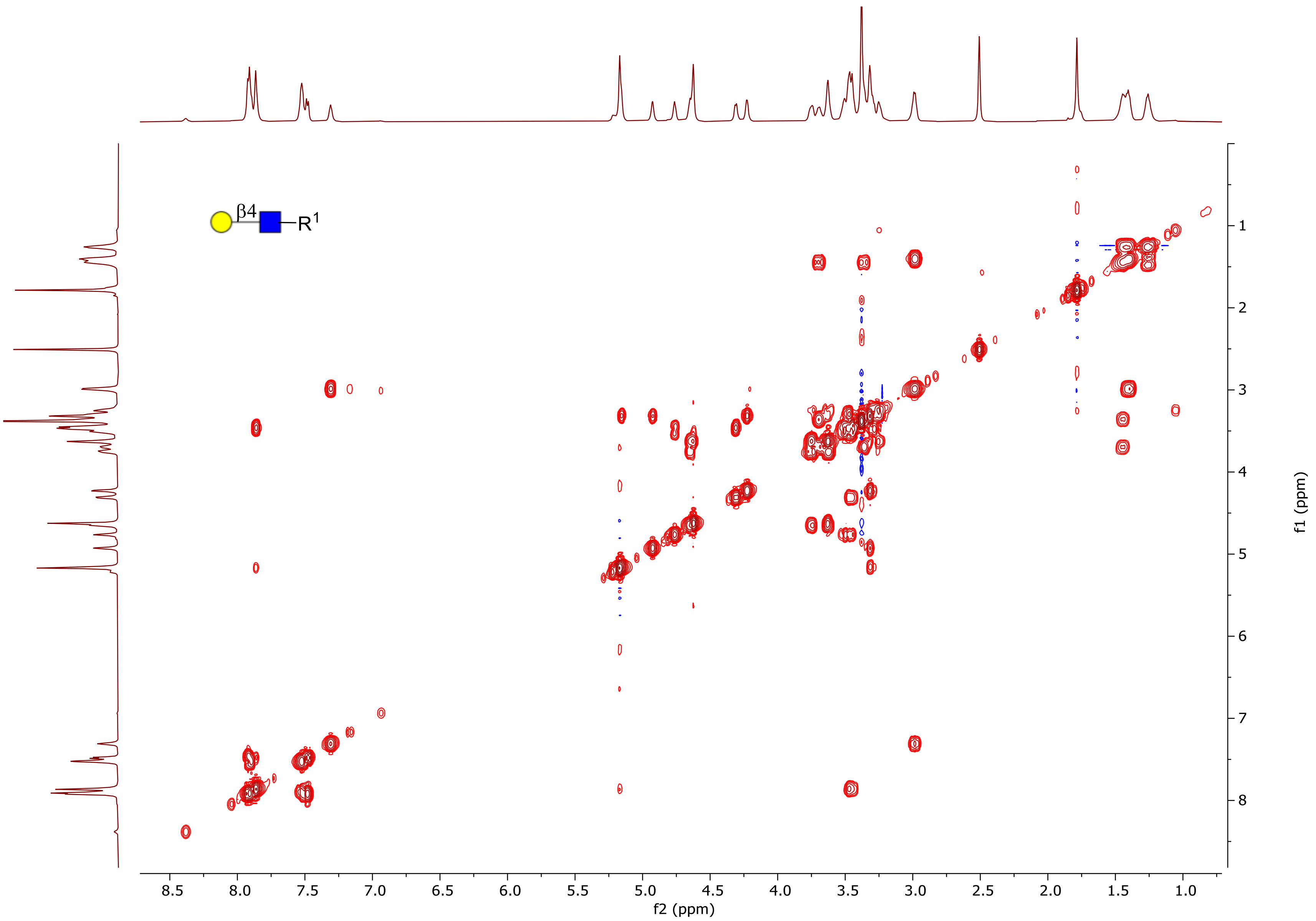

Multiplicity edited gHSQC NMR of Compound **9**

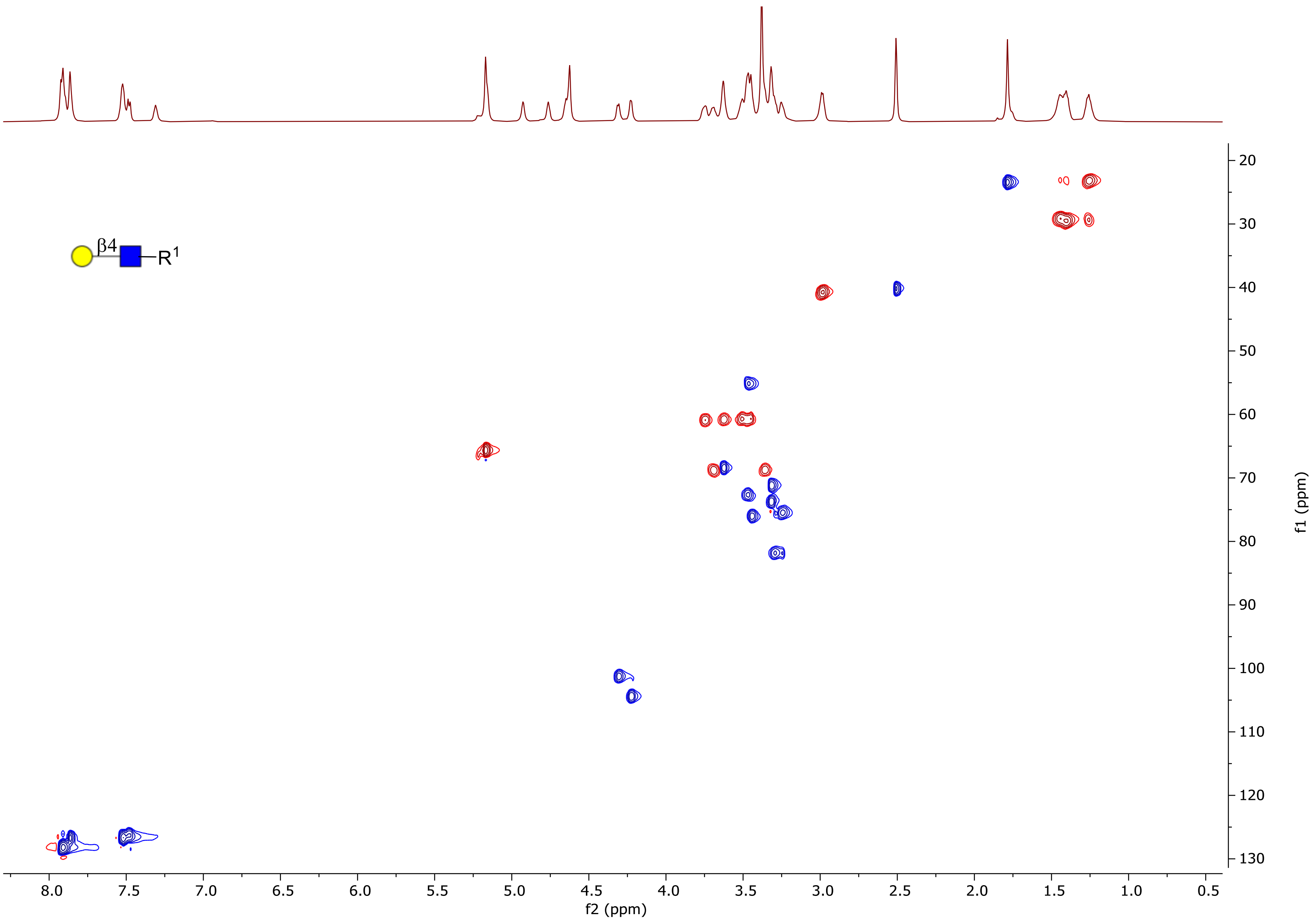

^{1}H NMR of Compound **10**

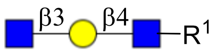

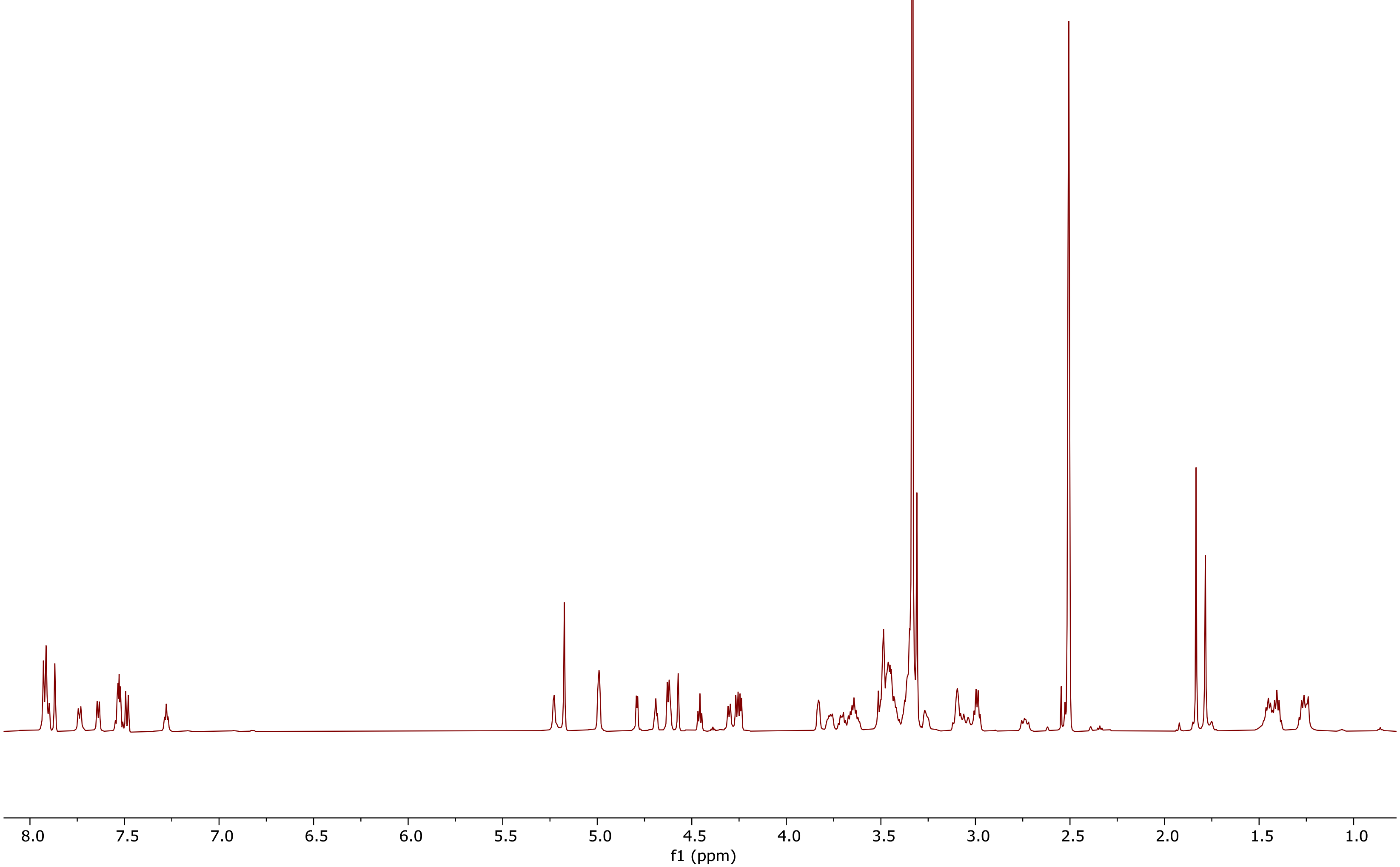

^{13}C NMR of Compound **10**

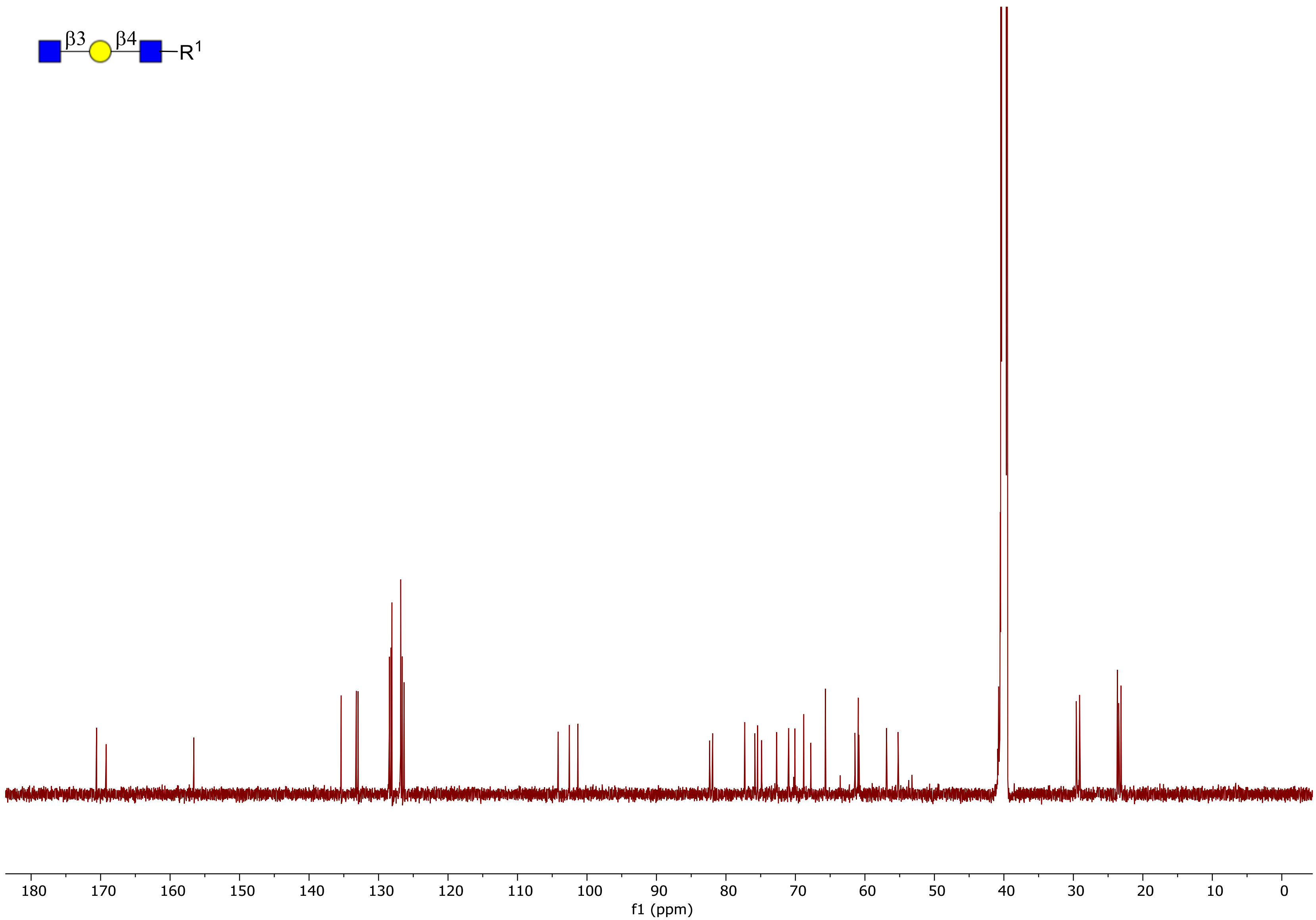

gCOSY NMR of Compound **10**

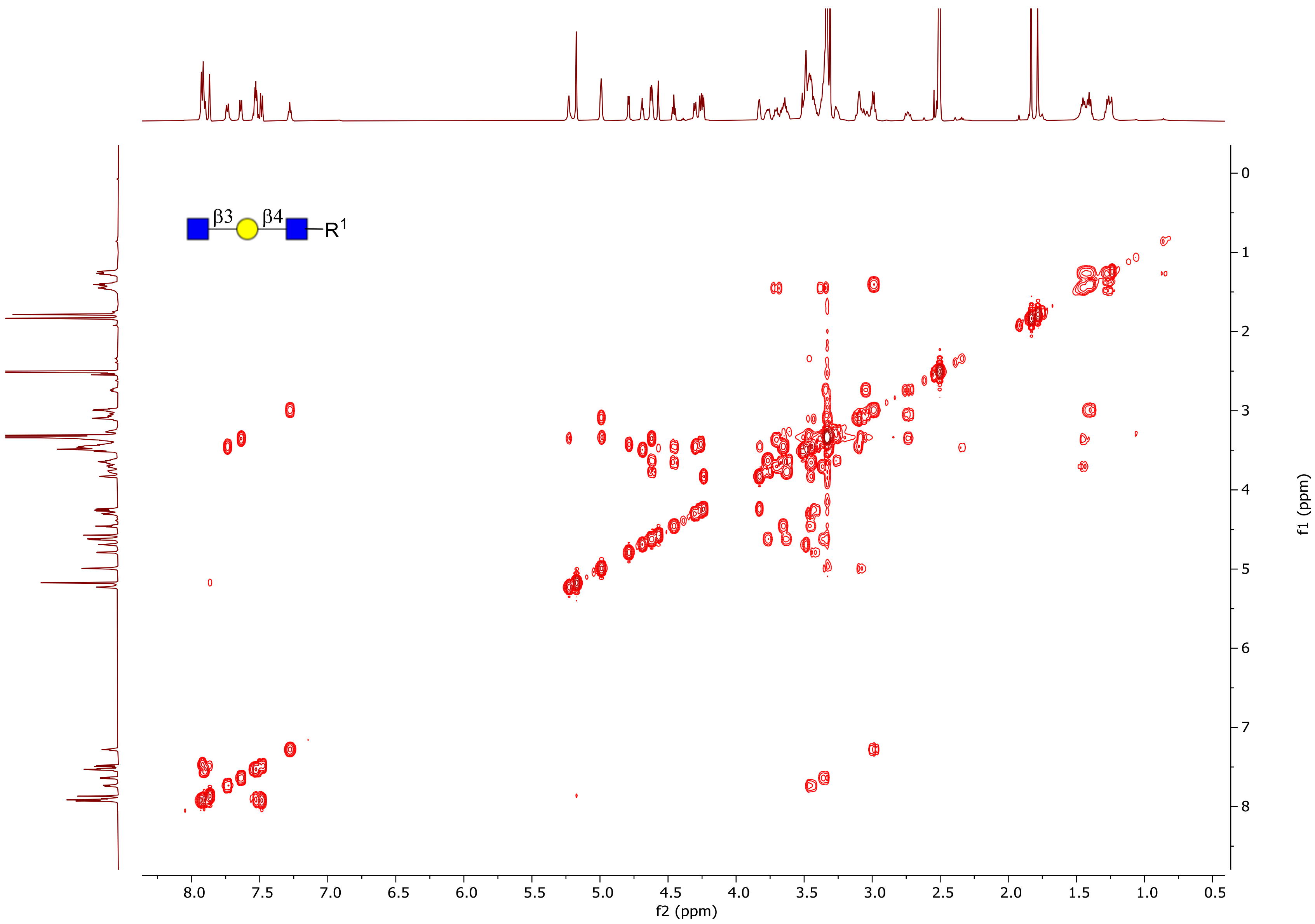

Multiplicity edited gHSQC NMR of Compound **10**

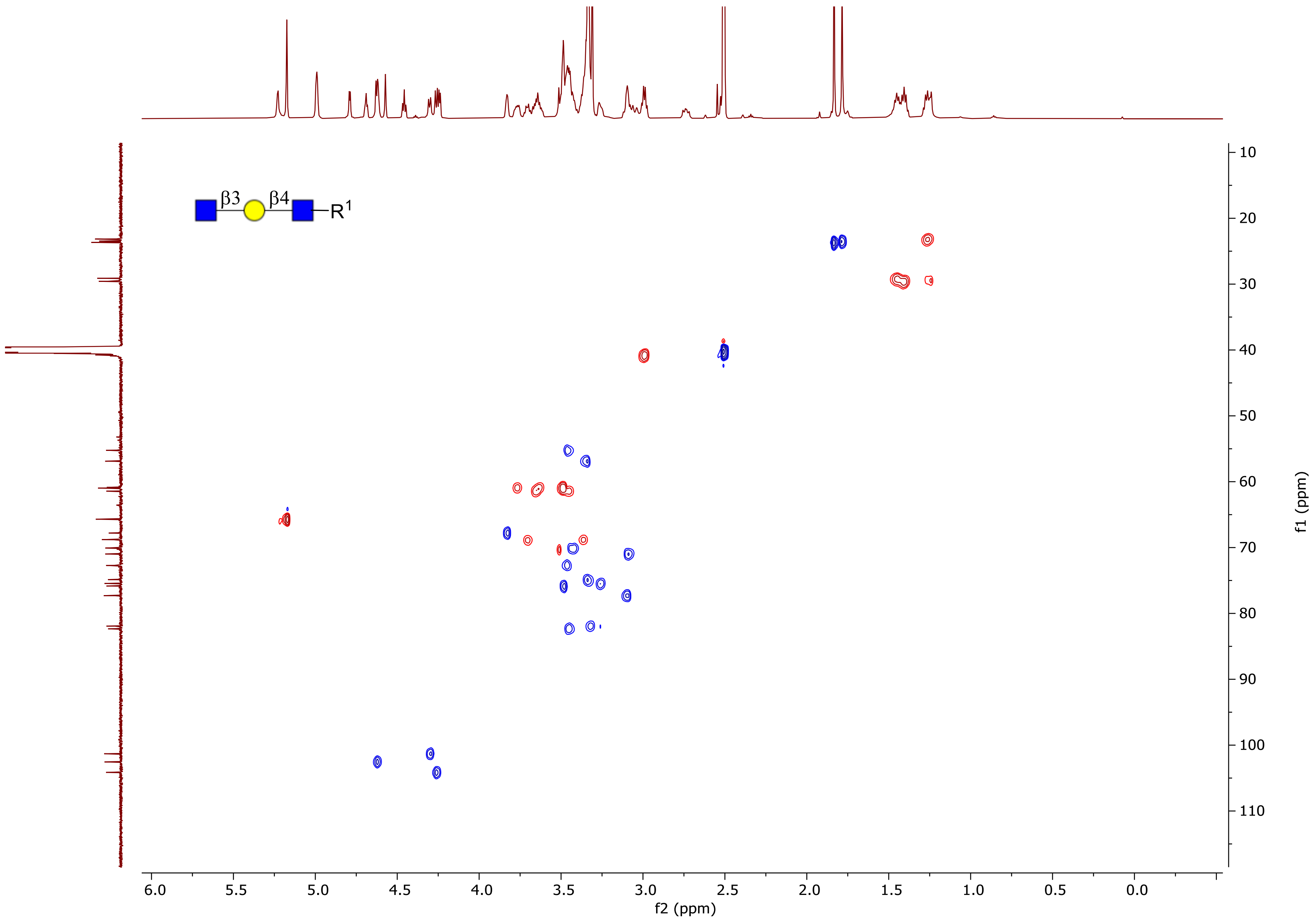

^{1}H NMR of Compound **12**

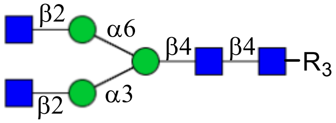

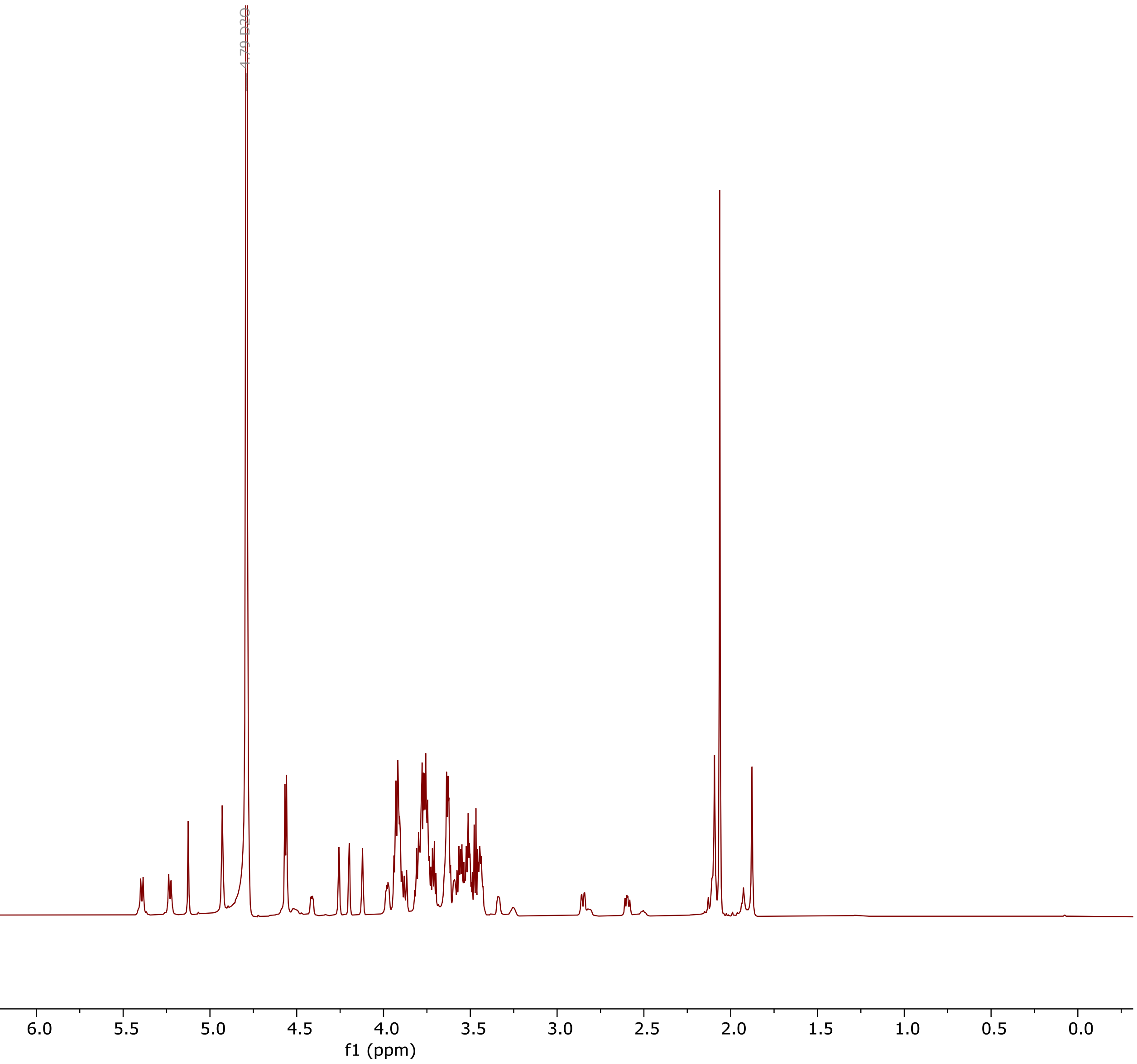

gCOSY NMR of Compound **12**

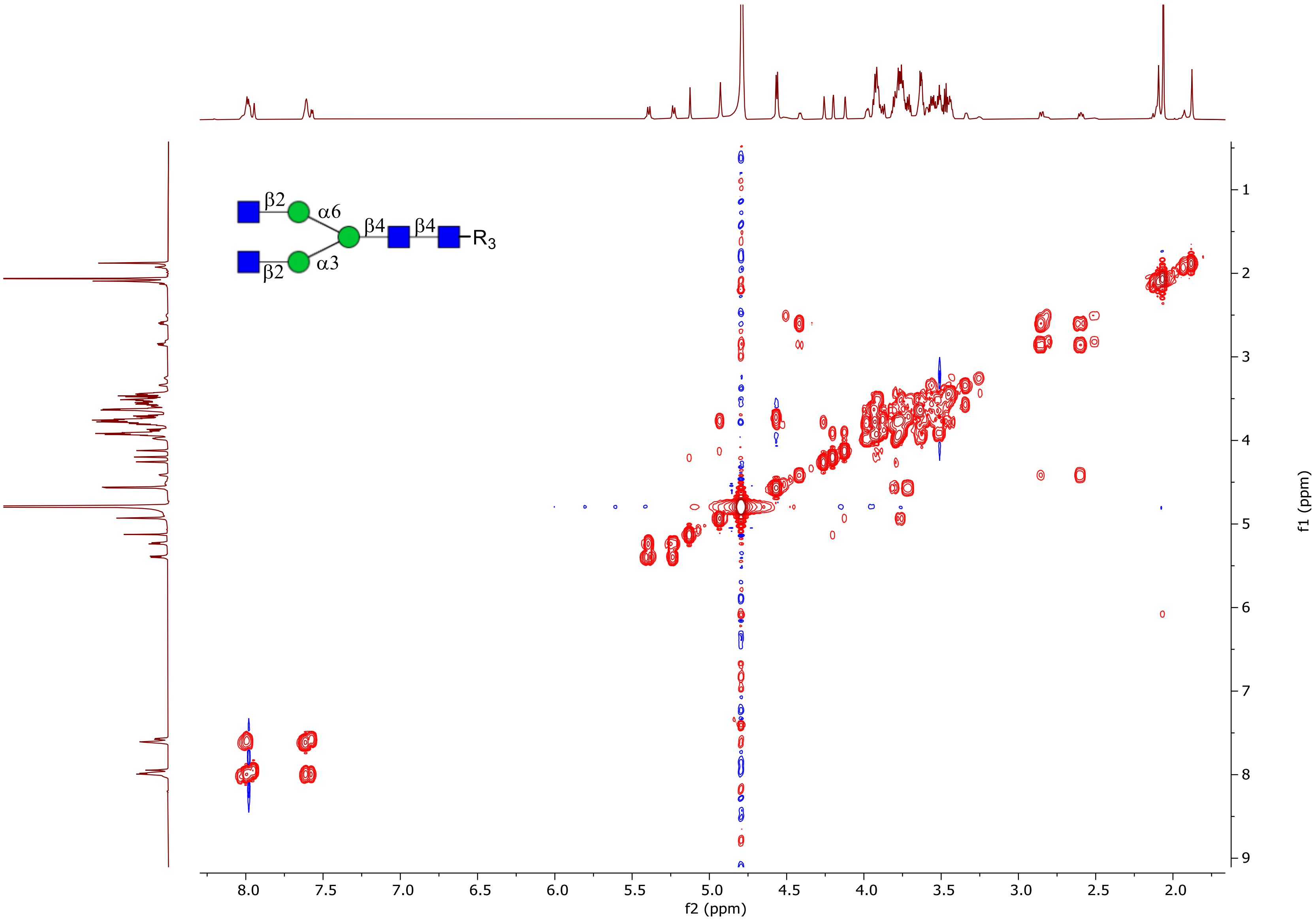

Multiplicity edited gHSQC NMR of Compound **12**

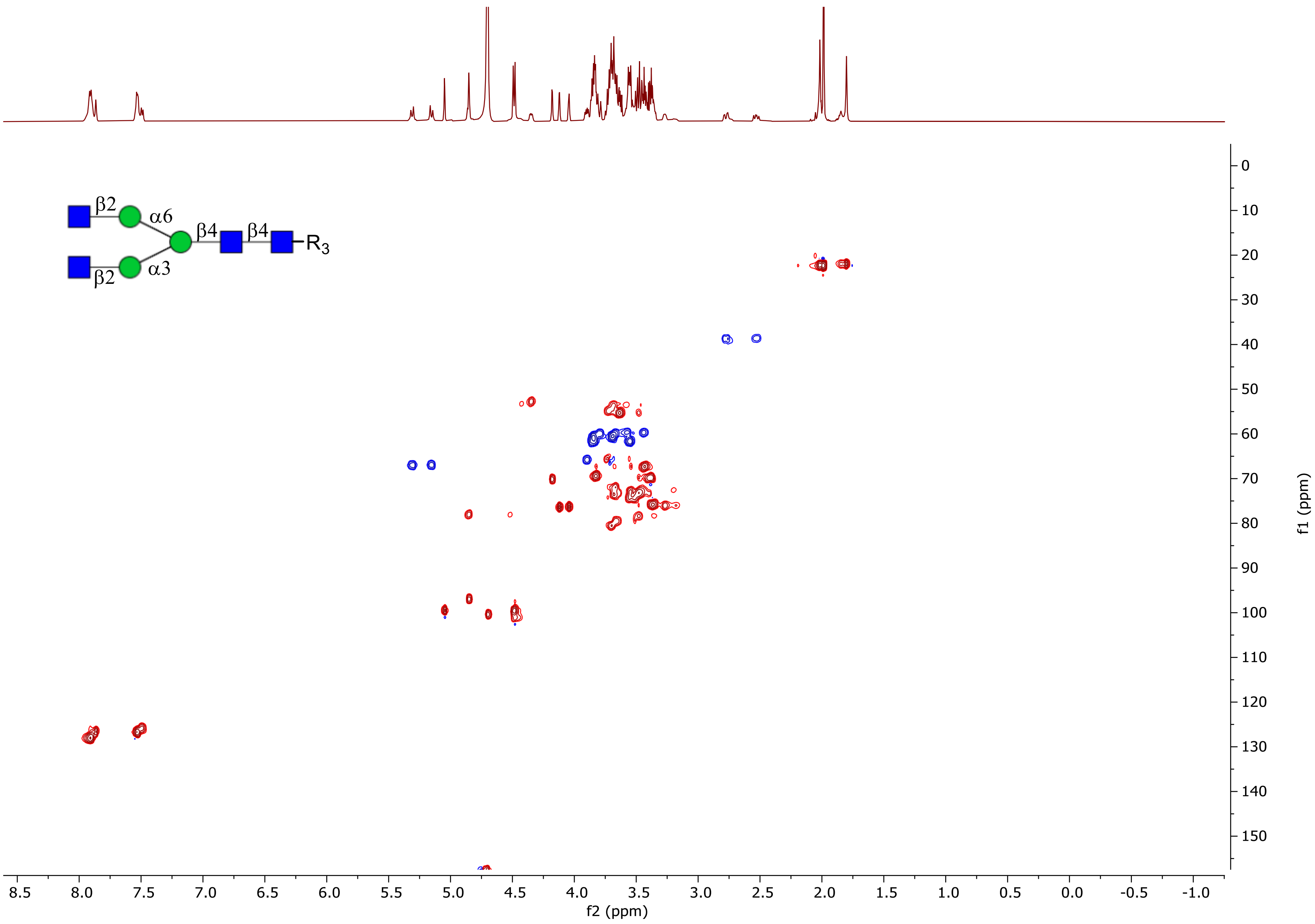

TOCSY-DIPSI NMR of Compound **12**

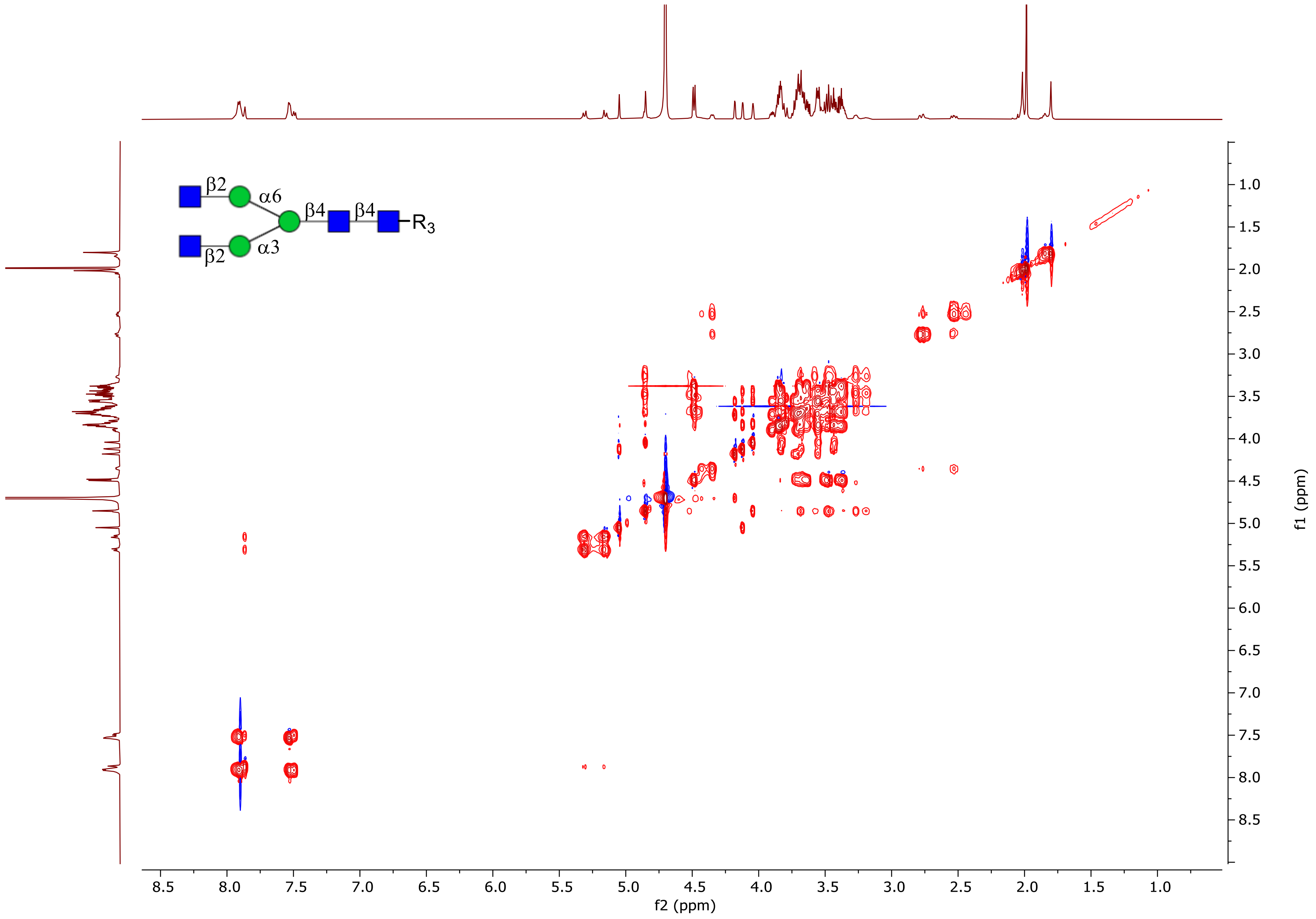

NOESY NMR of Compound **12**

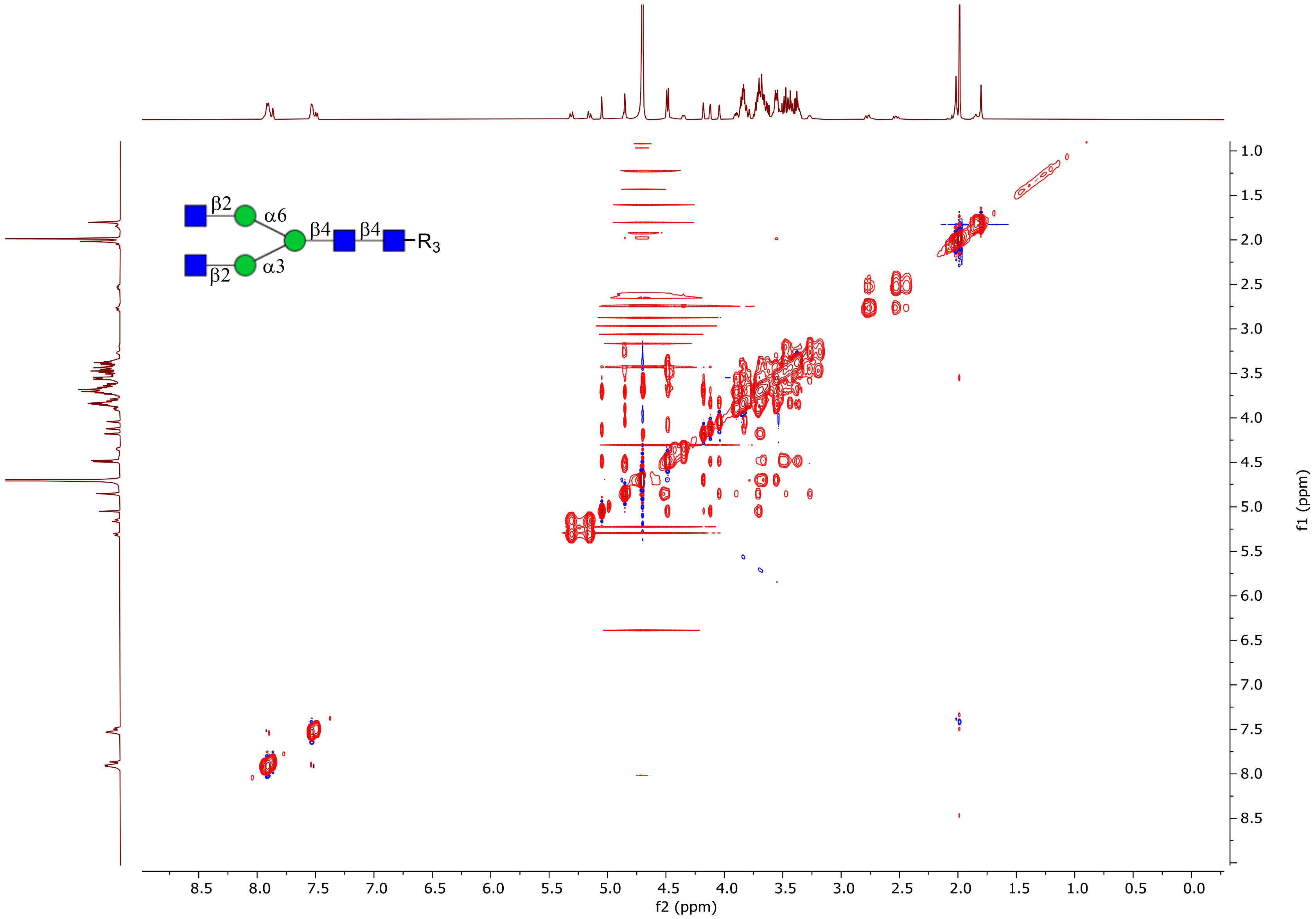

^{1}H NMR of Compound **13**

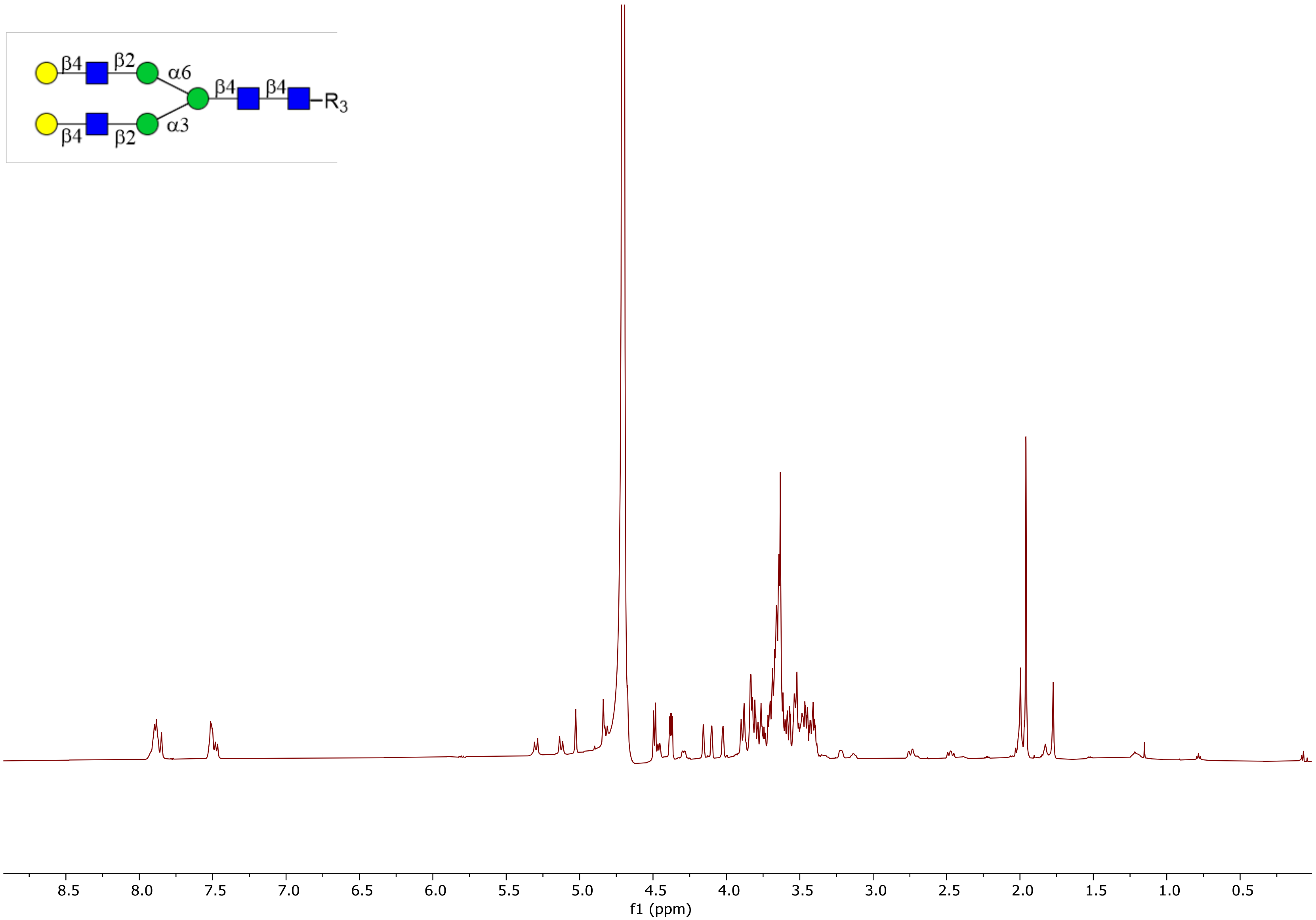

gCOSY NMR of Compound **13**

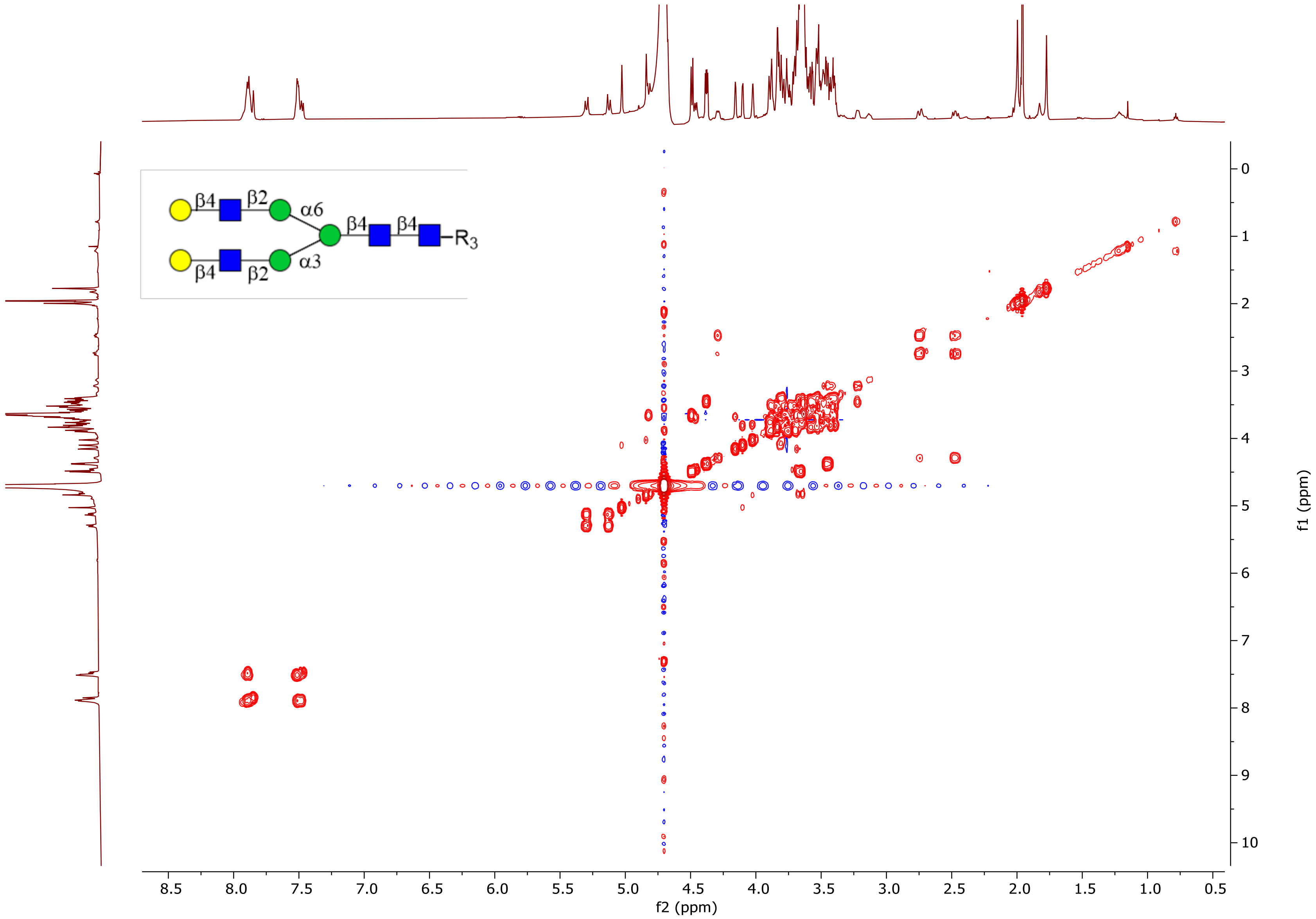

Multiplicity edited gHSQC NMR of Compound **13**

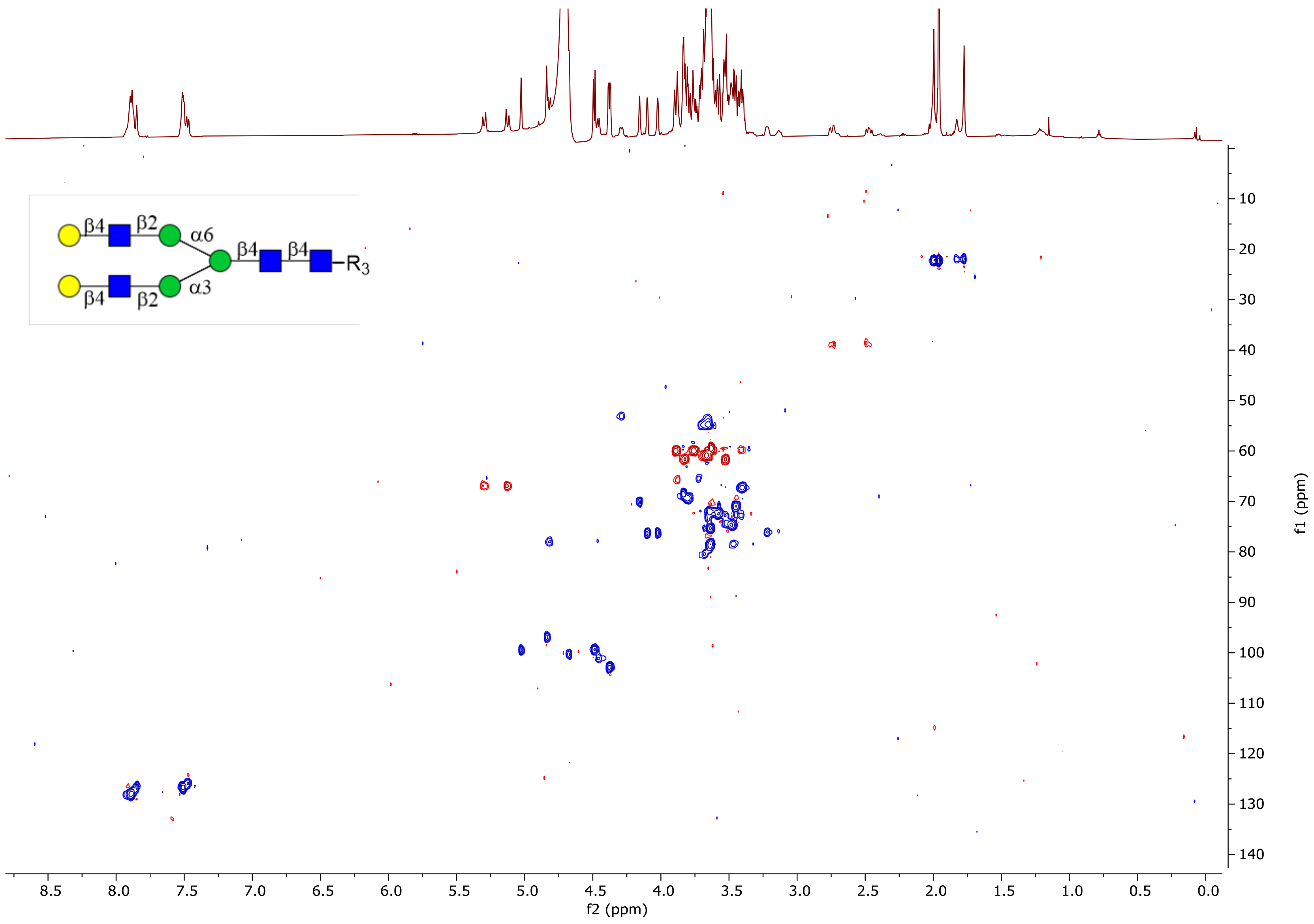

NOESY NMR of Compound **13**

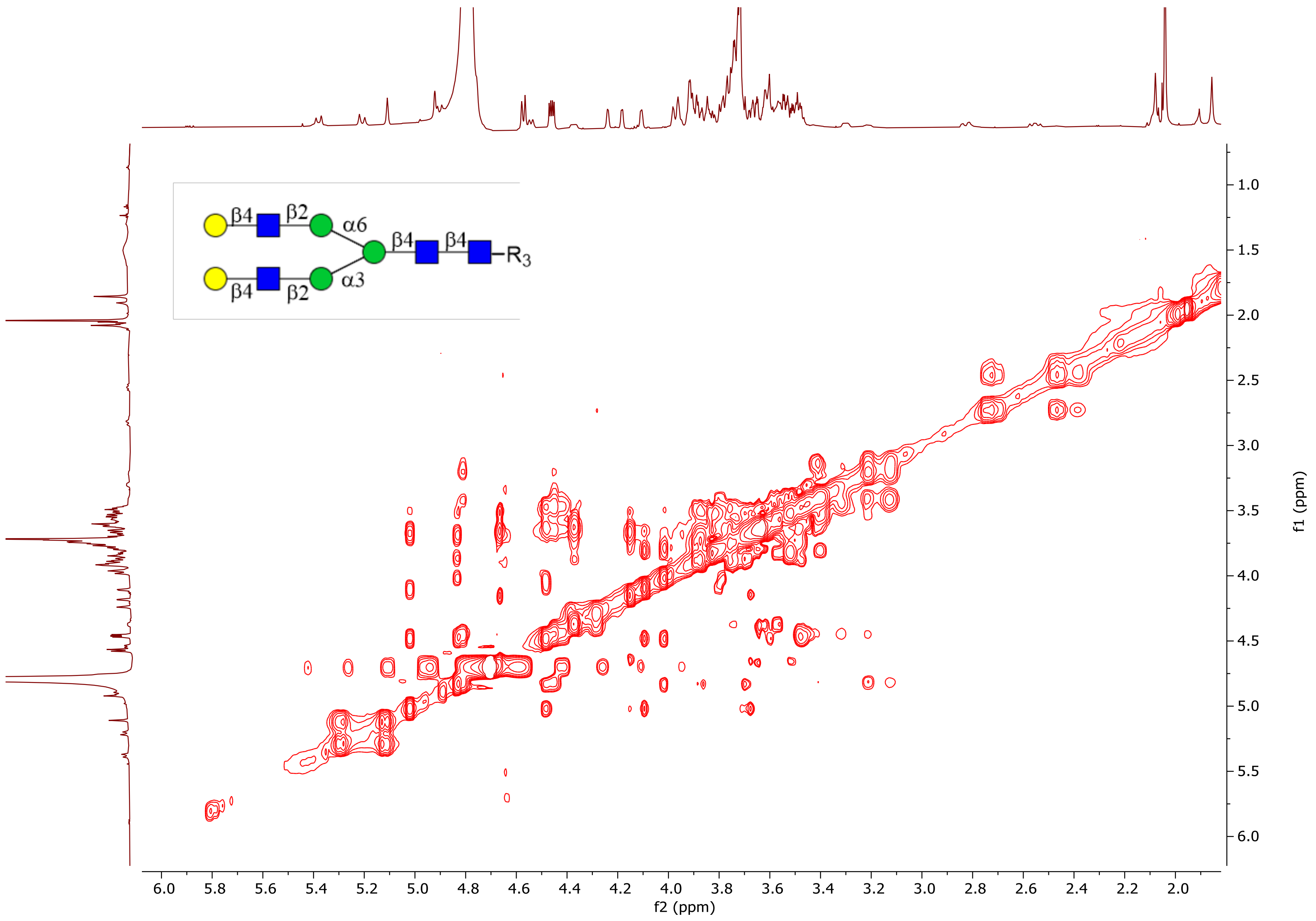

TOCSY-DIPSI NMR of Compound **13**

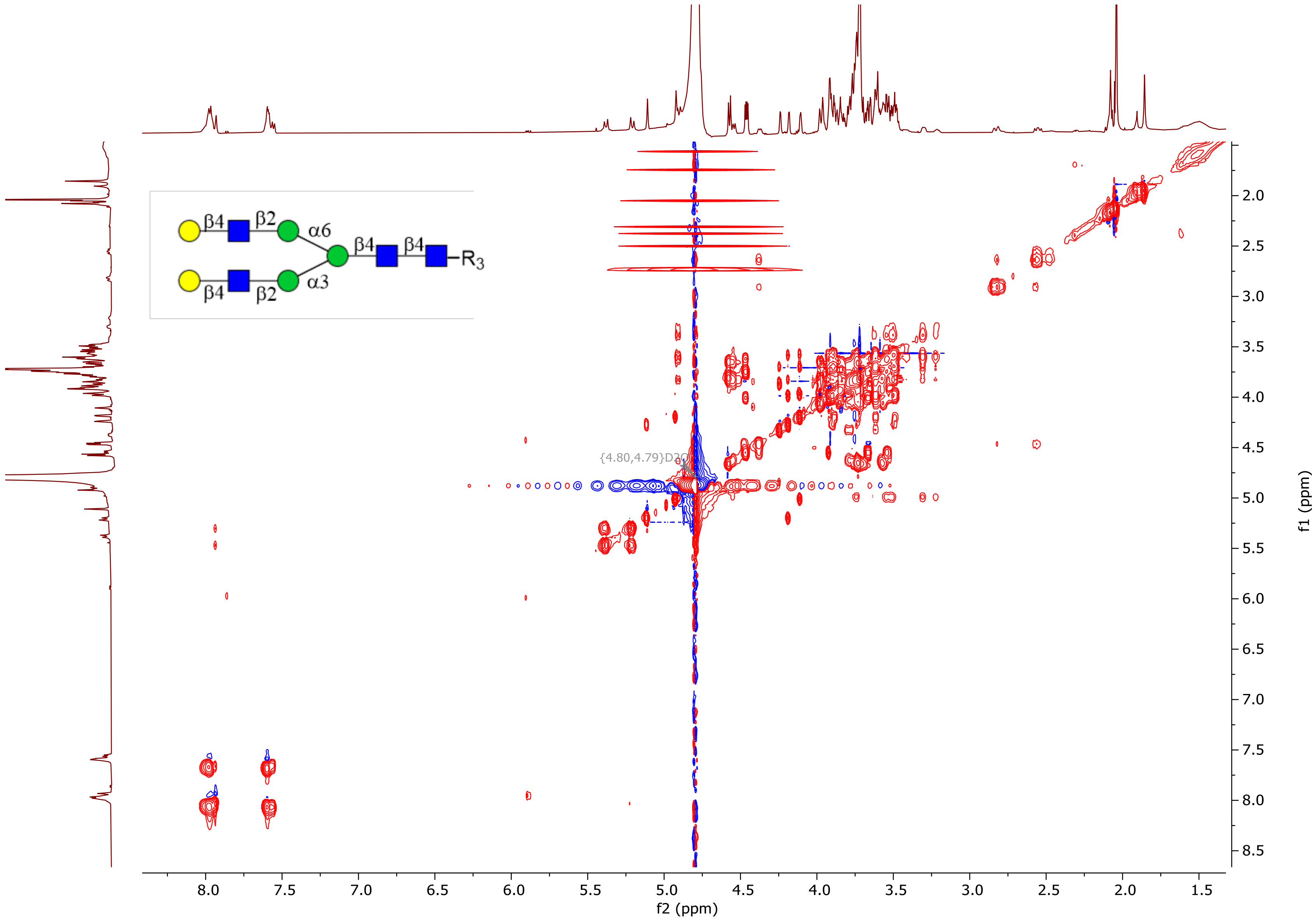

^{1}H NMR of Compound **15**

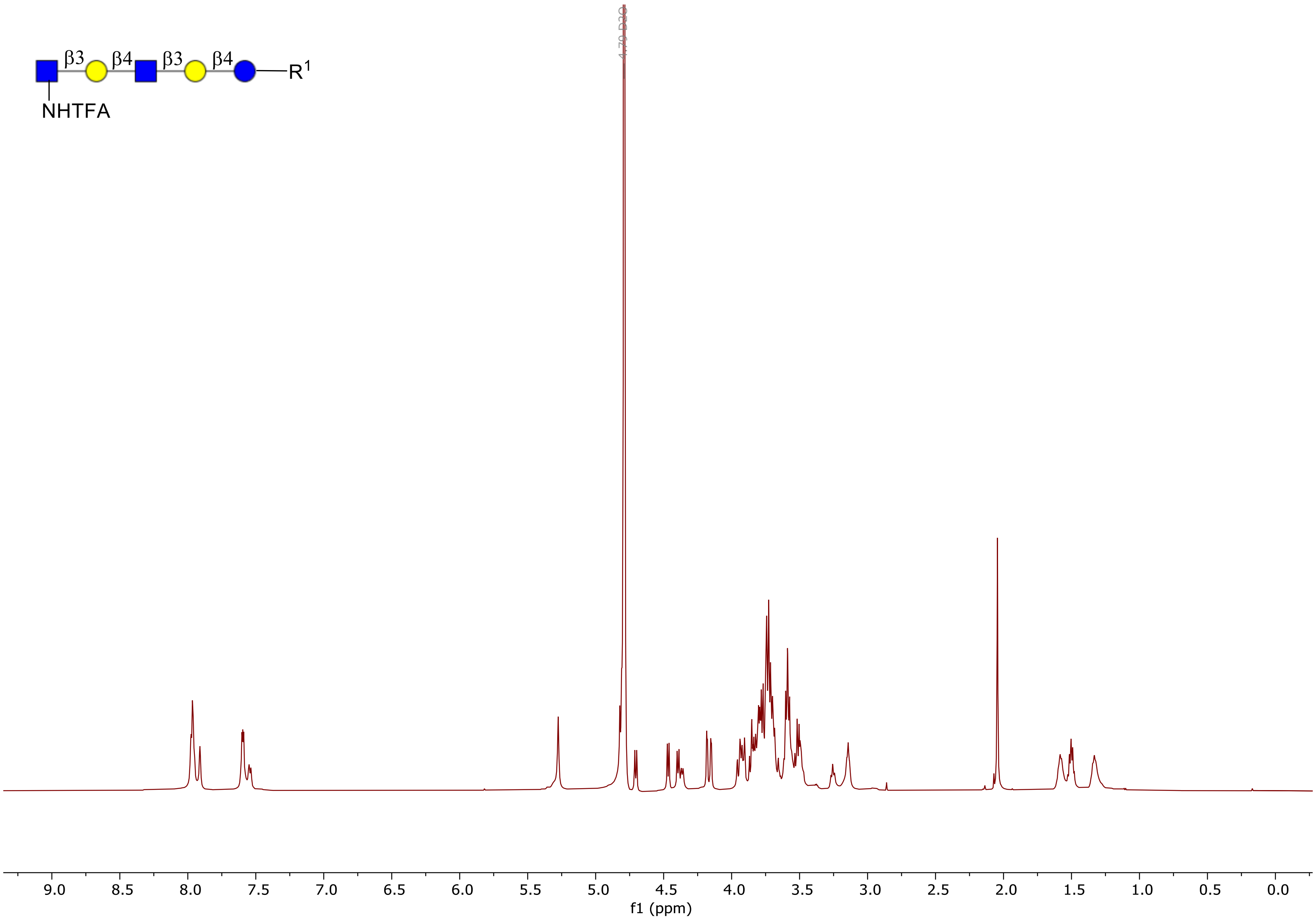

gCOSY NMR of Compound **15**

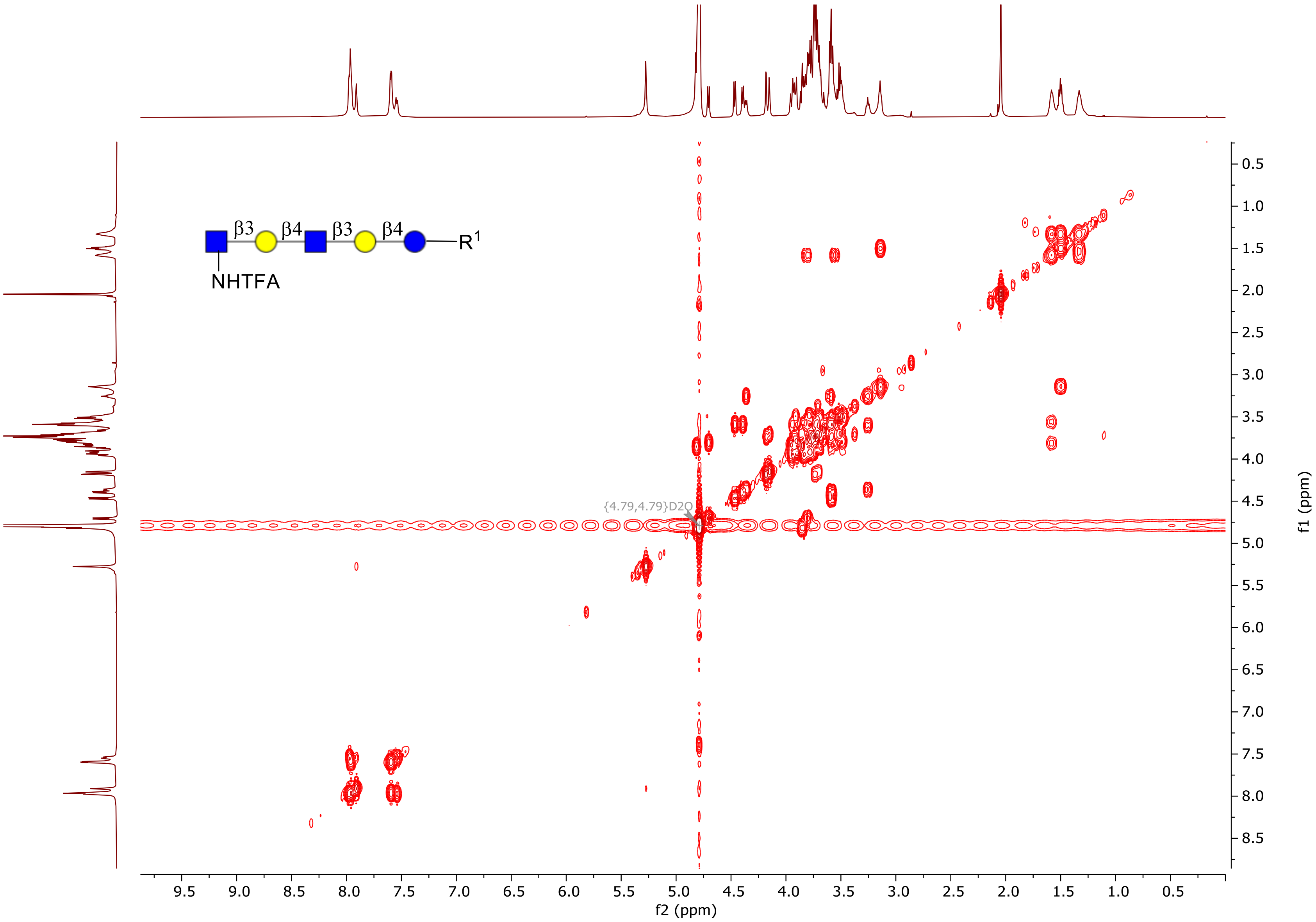

Multiplicity edited gHSQC NMR of Compound **15**

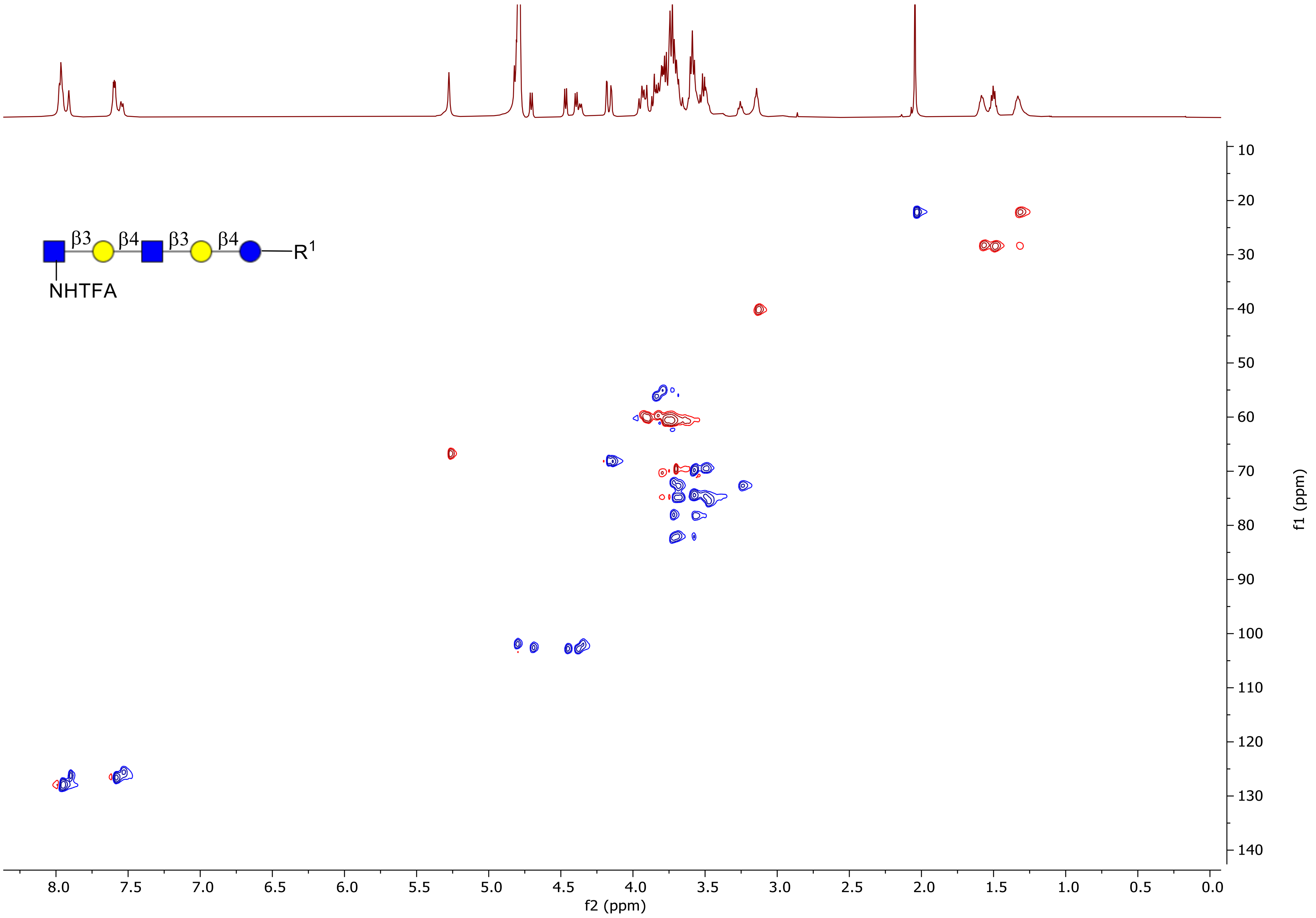

TOCSY-DIPSI NMR of Compound **15**

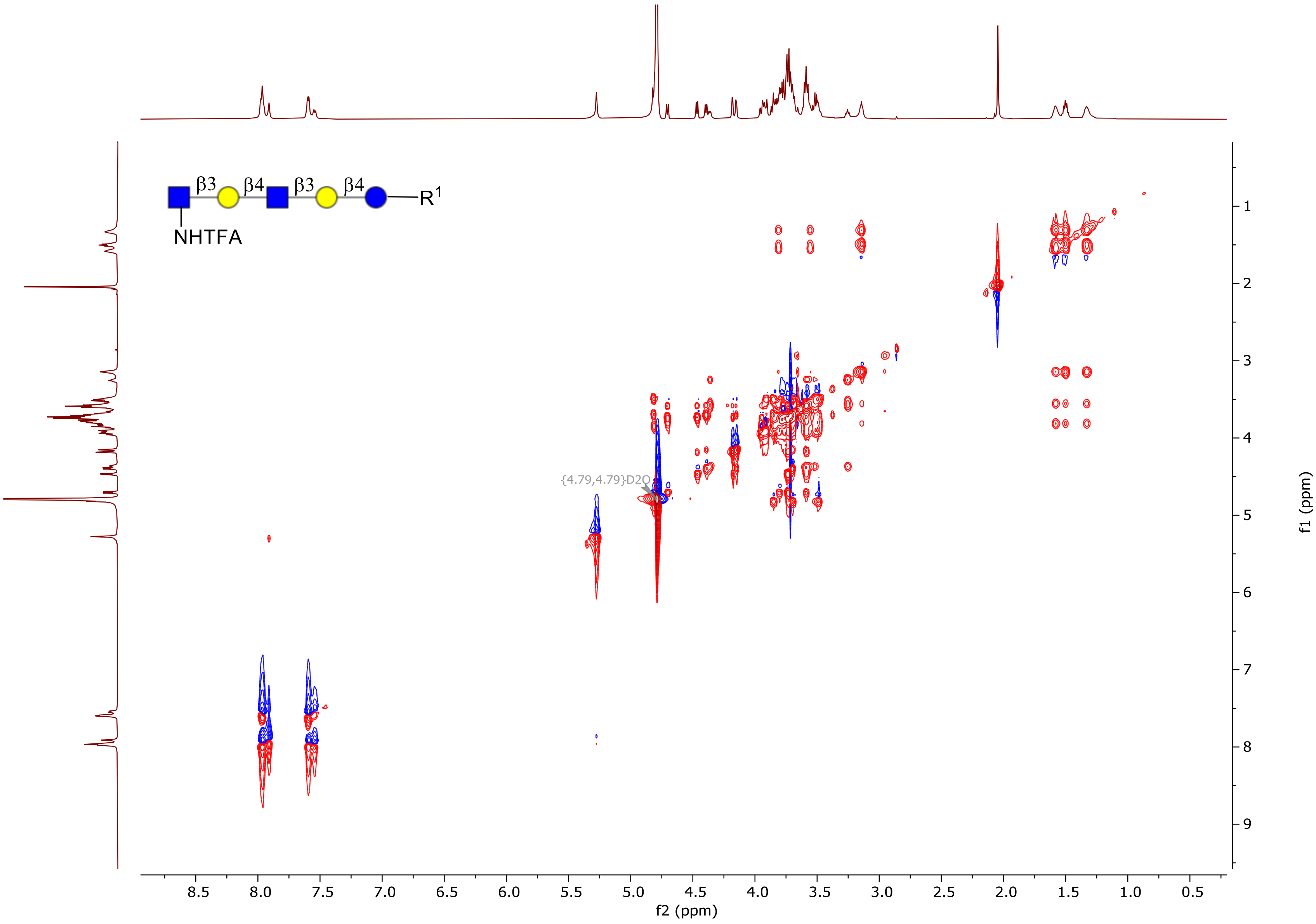

NOESY NMR of Compound **15**

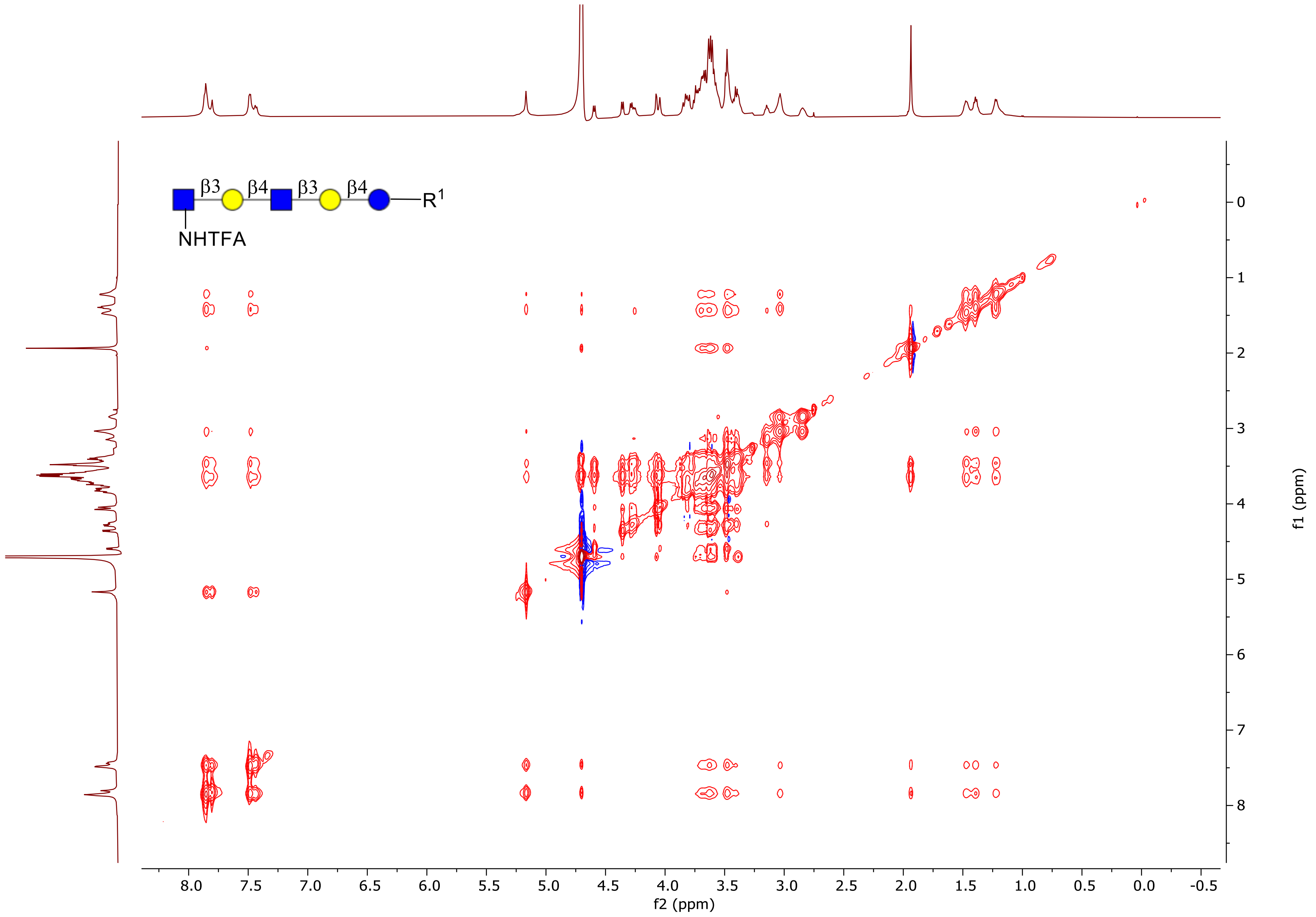

^{1}H NMR of Compound **16**

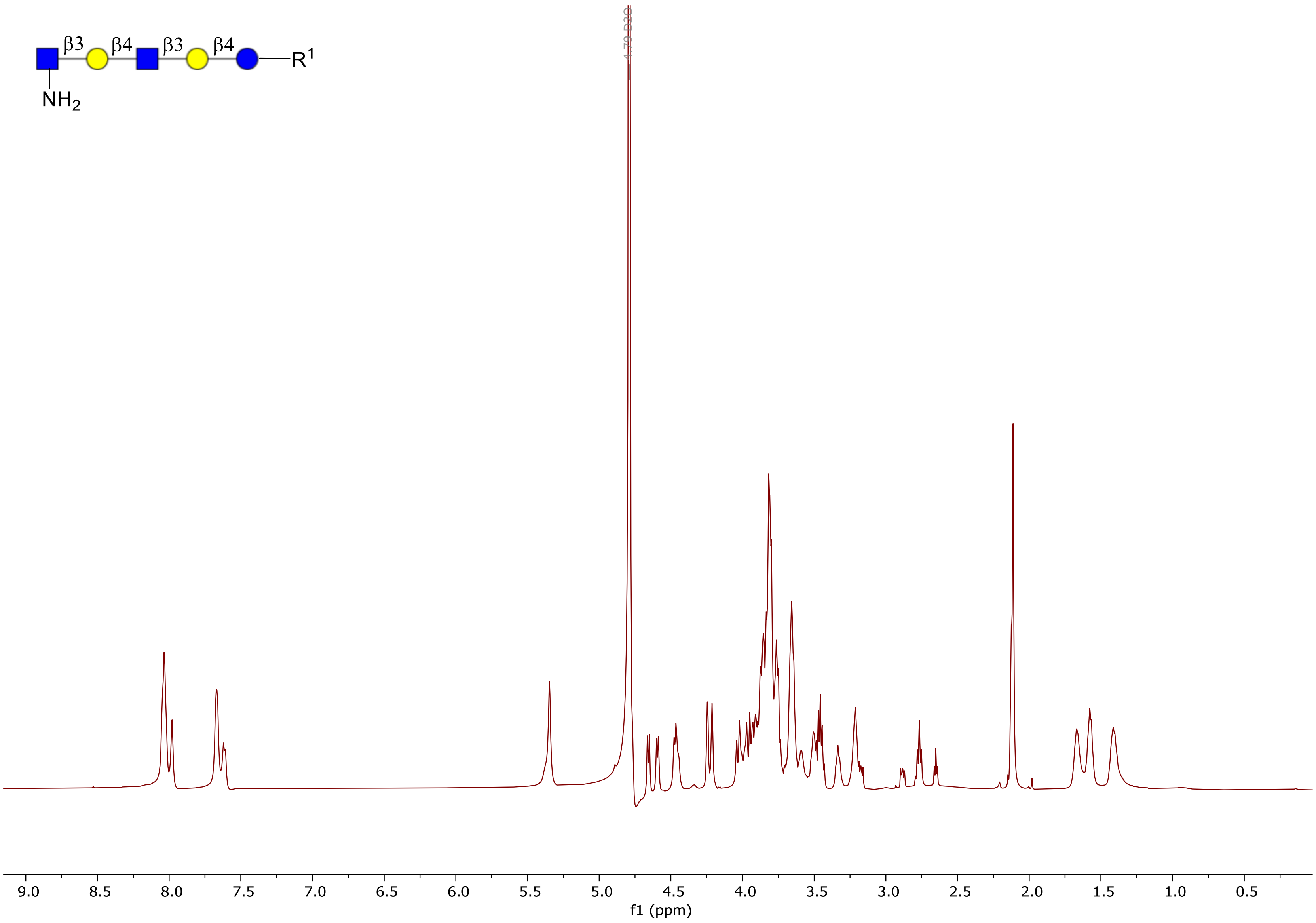

gCOSY NMR of Compound **16**

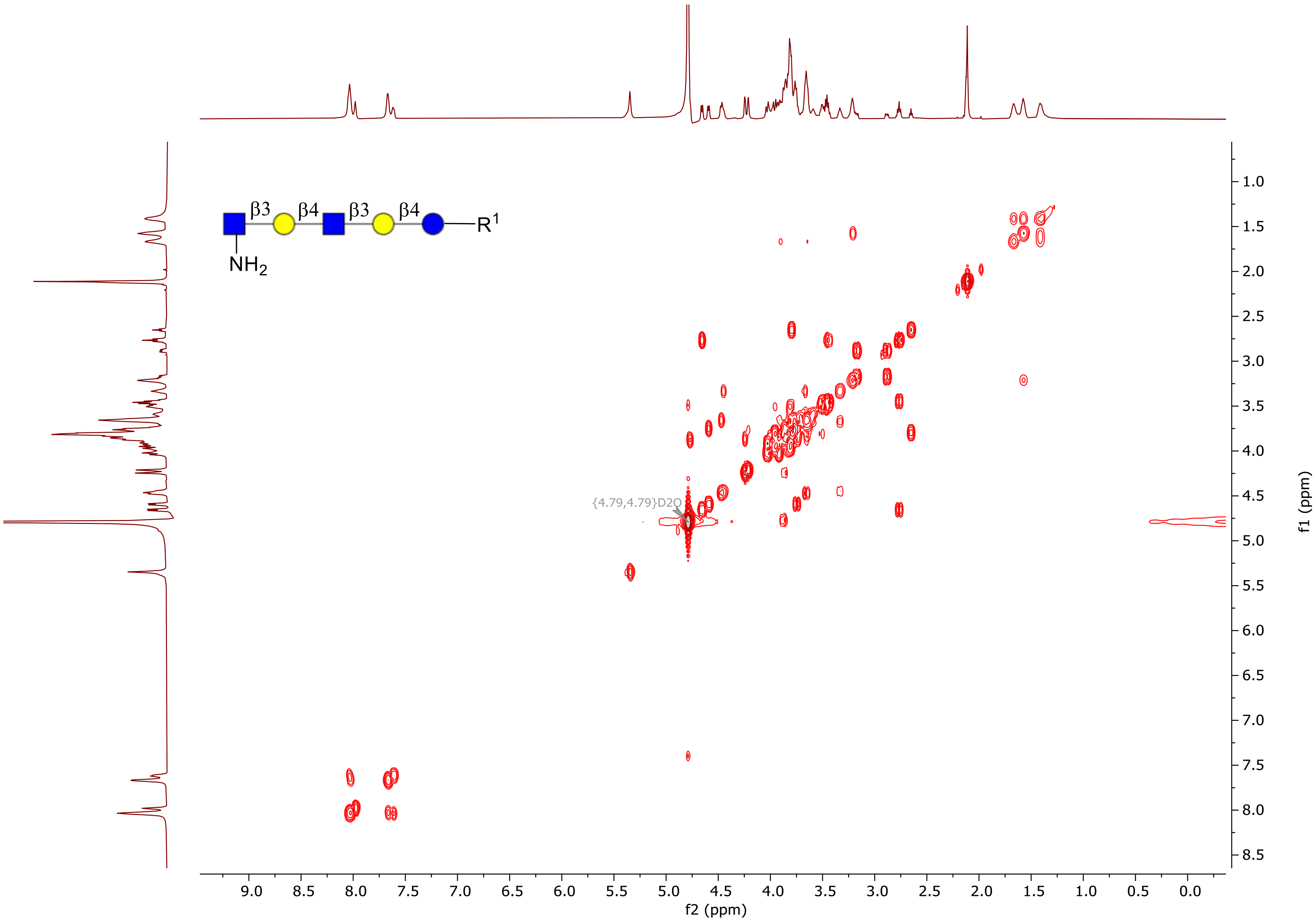

Multiplicity edited gHSQC NMR of Compound **16**

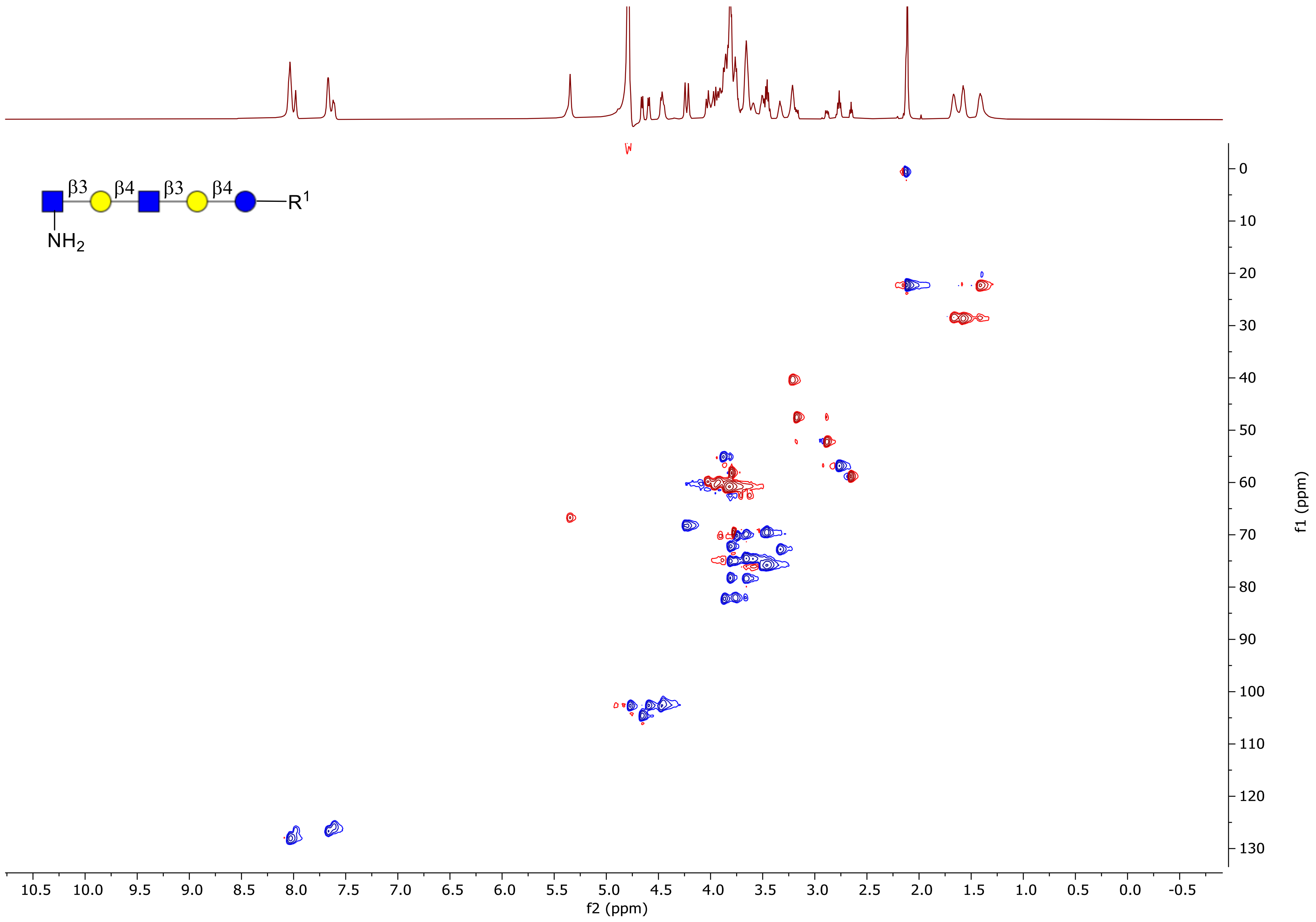

NOESY NMR of Compound **16**

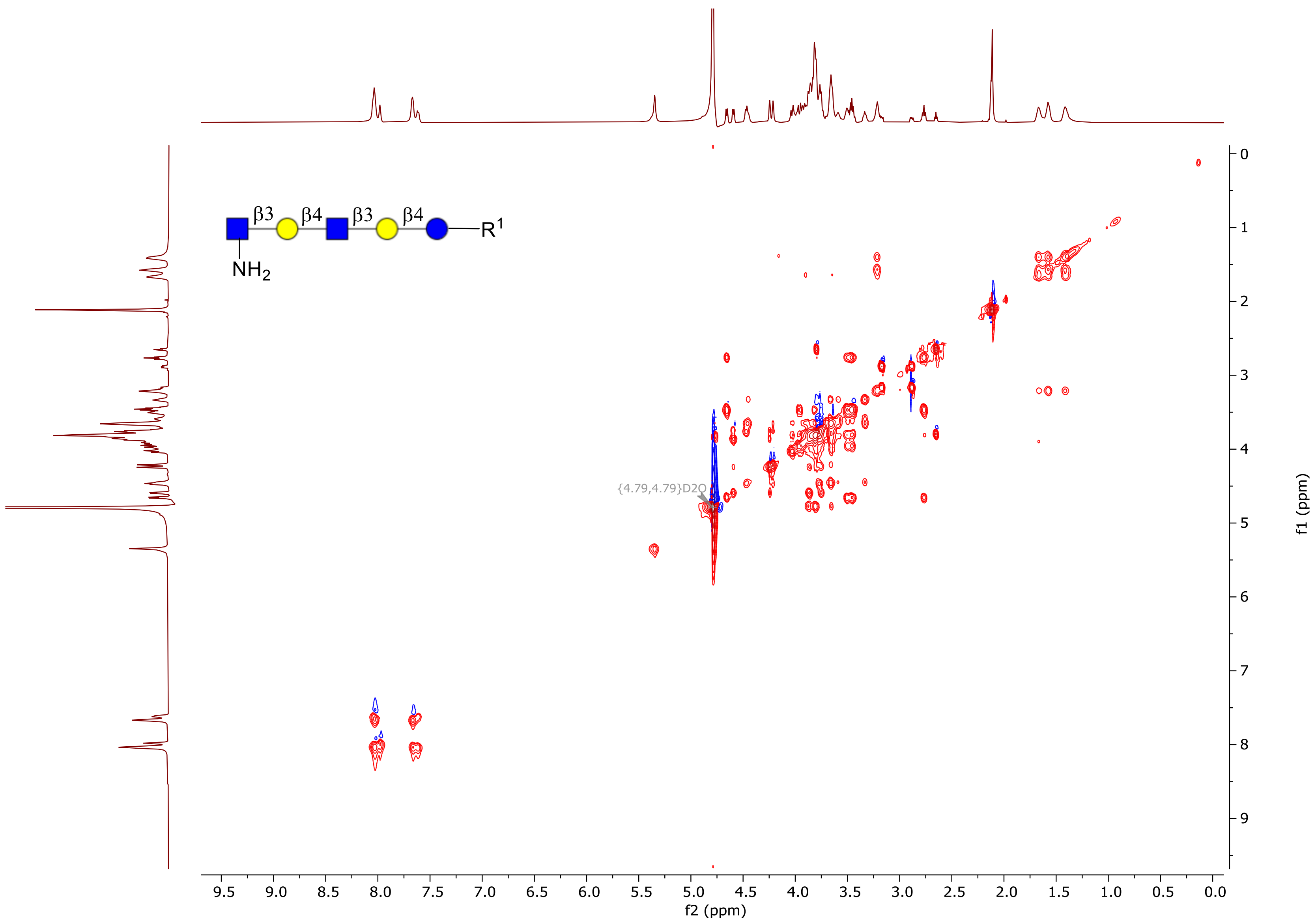

TOCSY-DIPSI NMR of Compound **16**

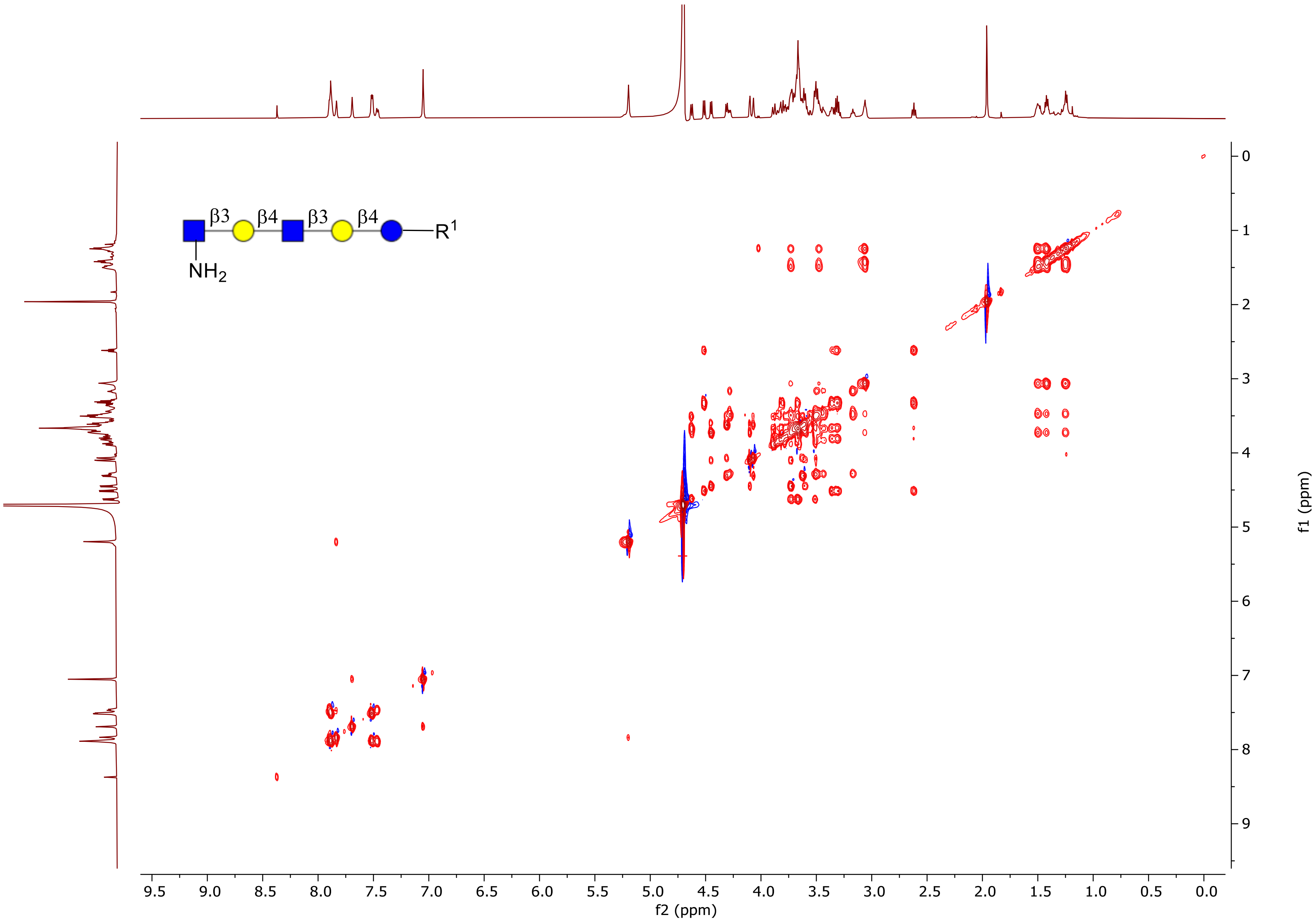

^{1}H NMR of Compound **17**

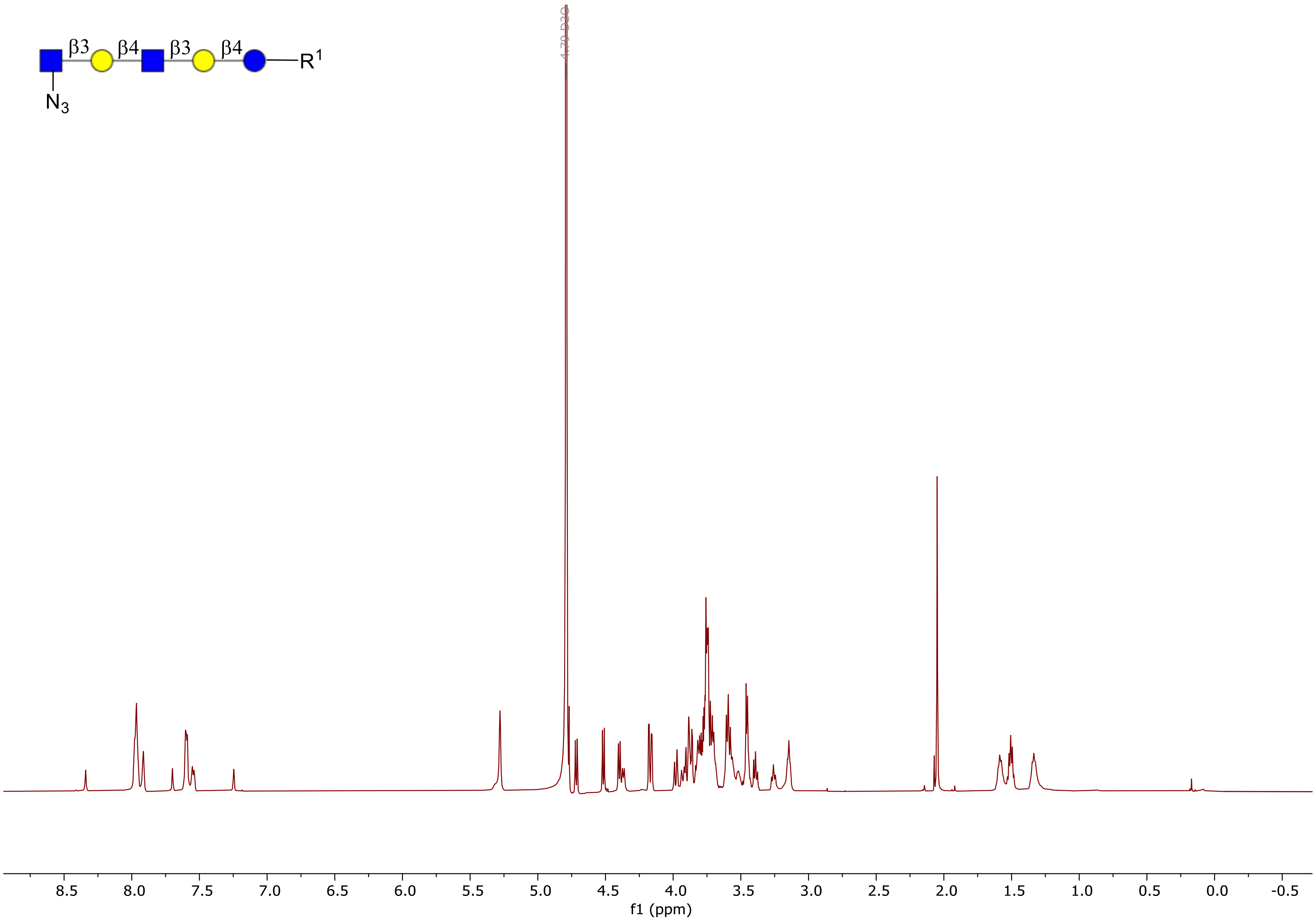

gCOSY NMR of Compound **17**

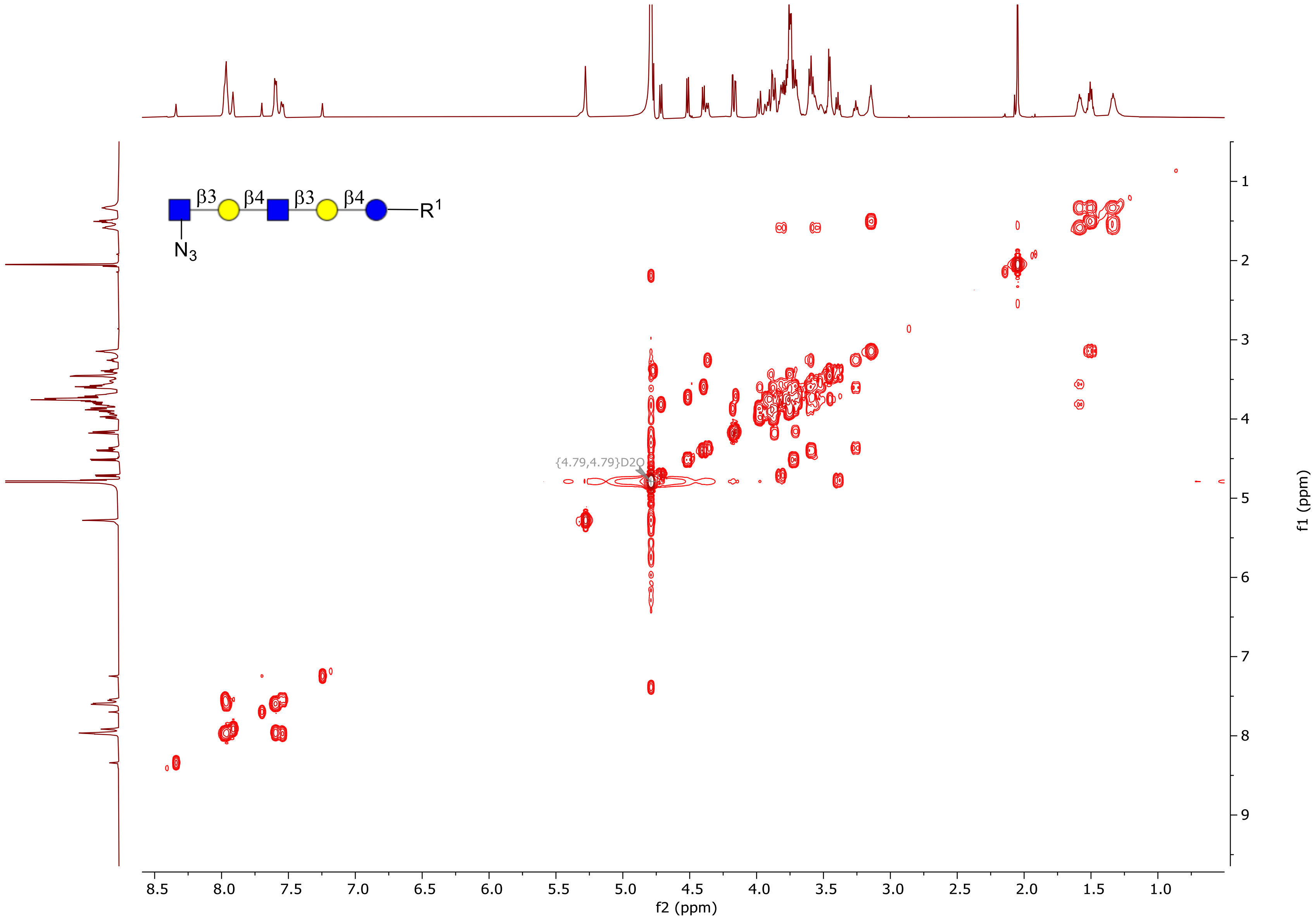

Multiplicity edited gHSQC NMR of Compound **17**

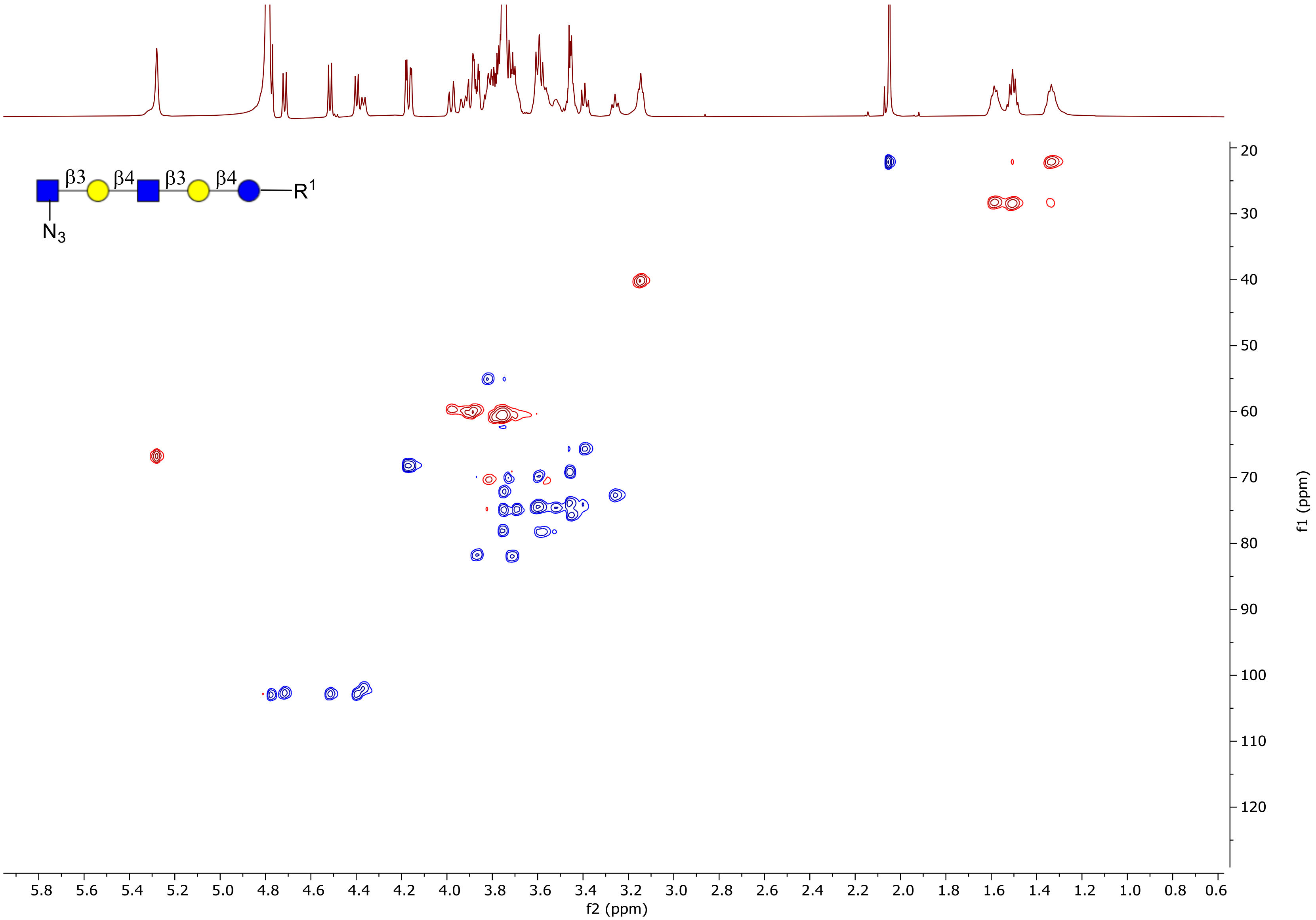

TOCSY-DIPSI NMR of Compound **17**

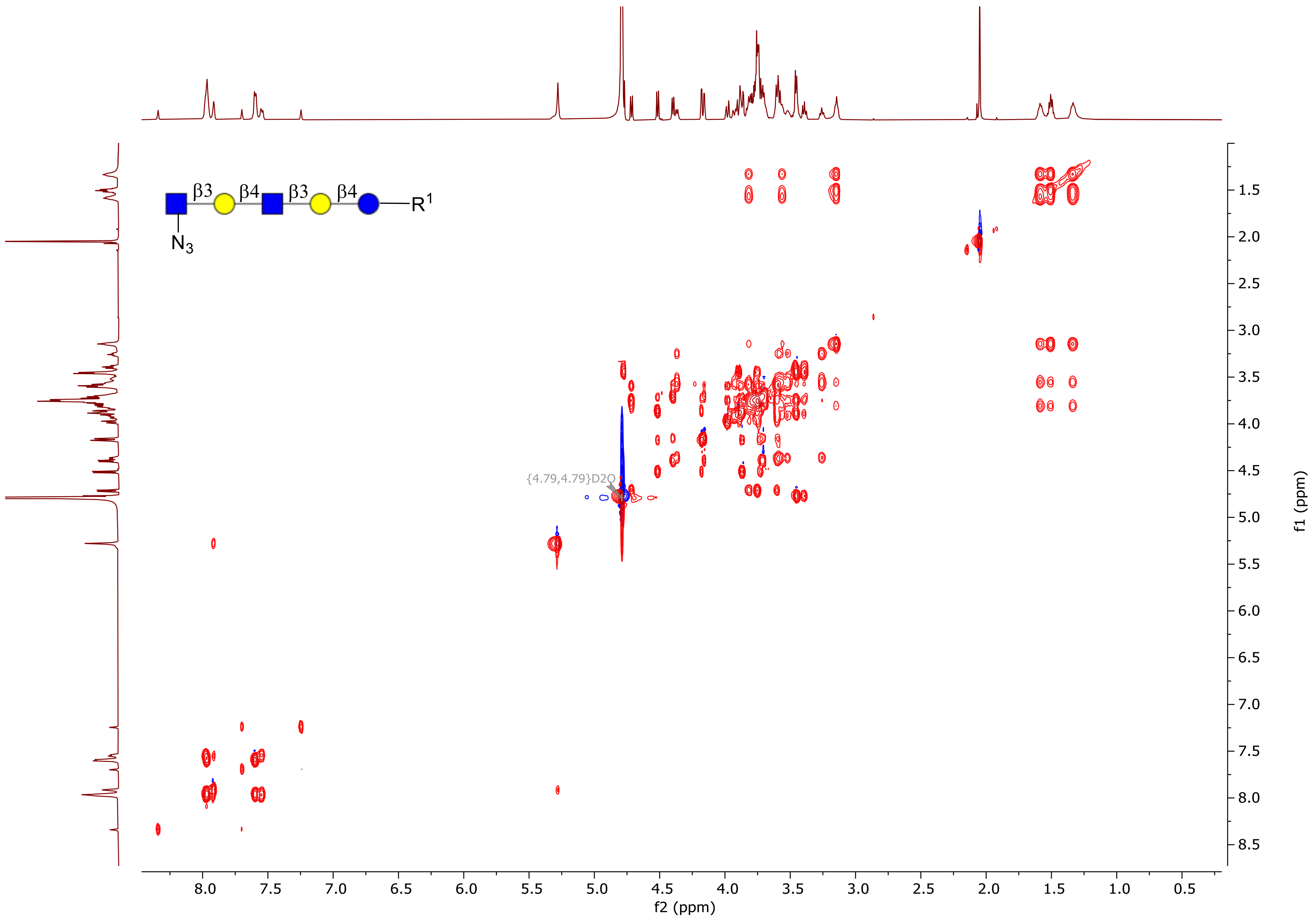

NOESY NMR of Compound **17**

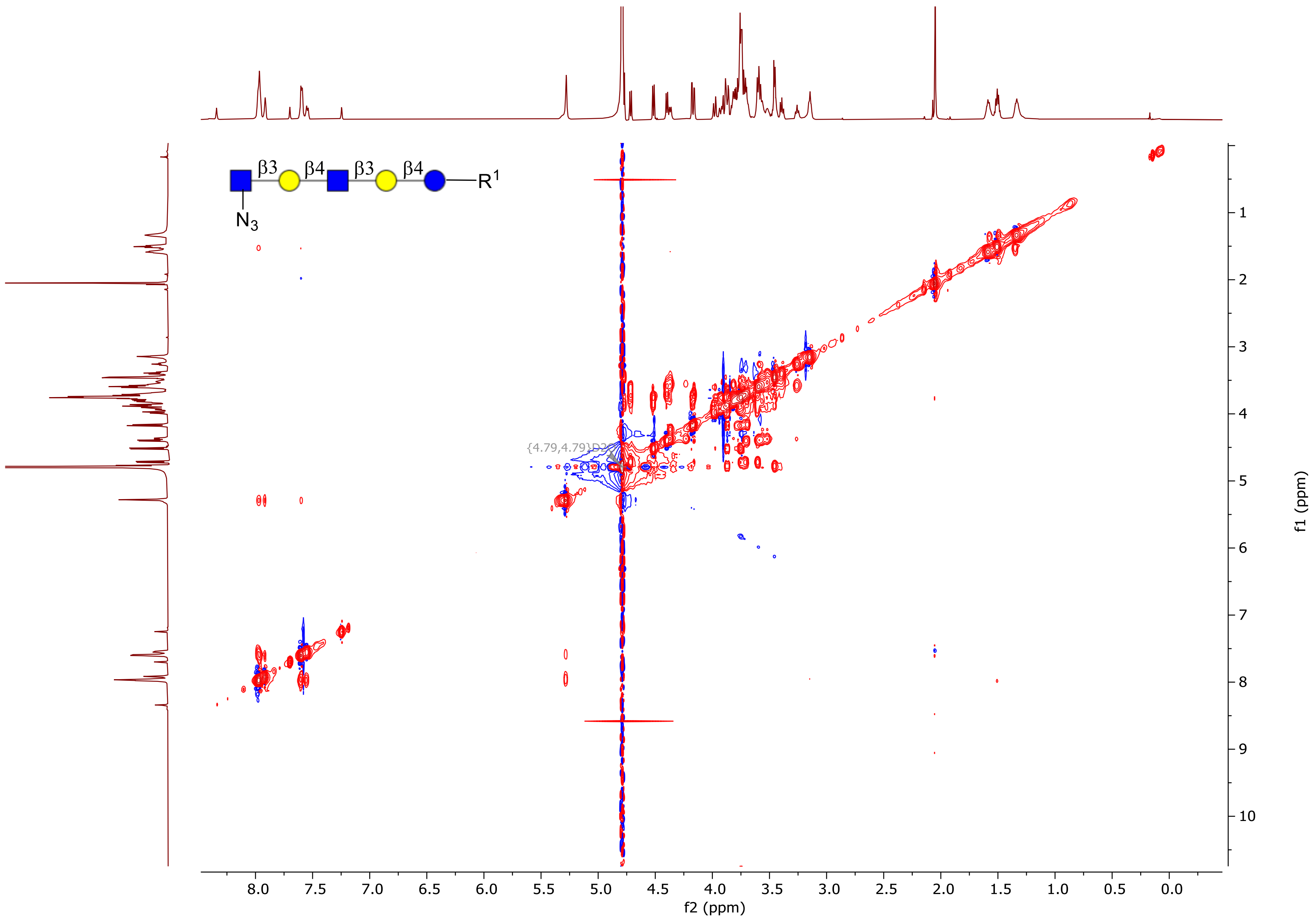

^{1}H NMR of Compound **18**

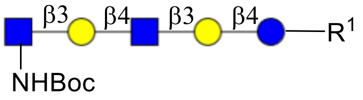

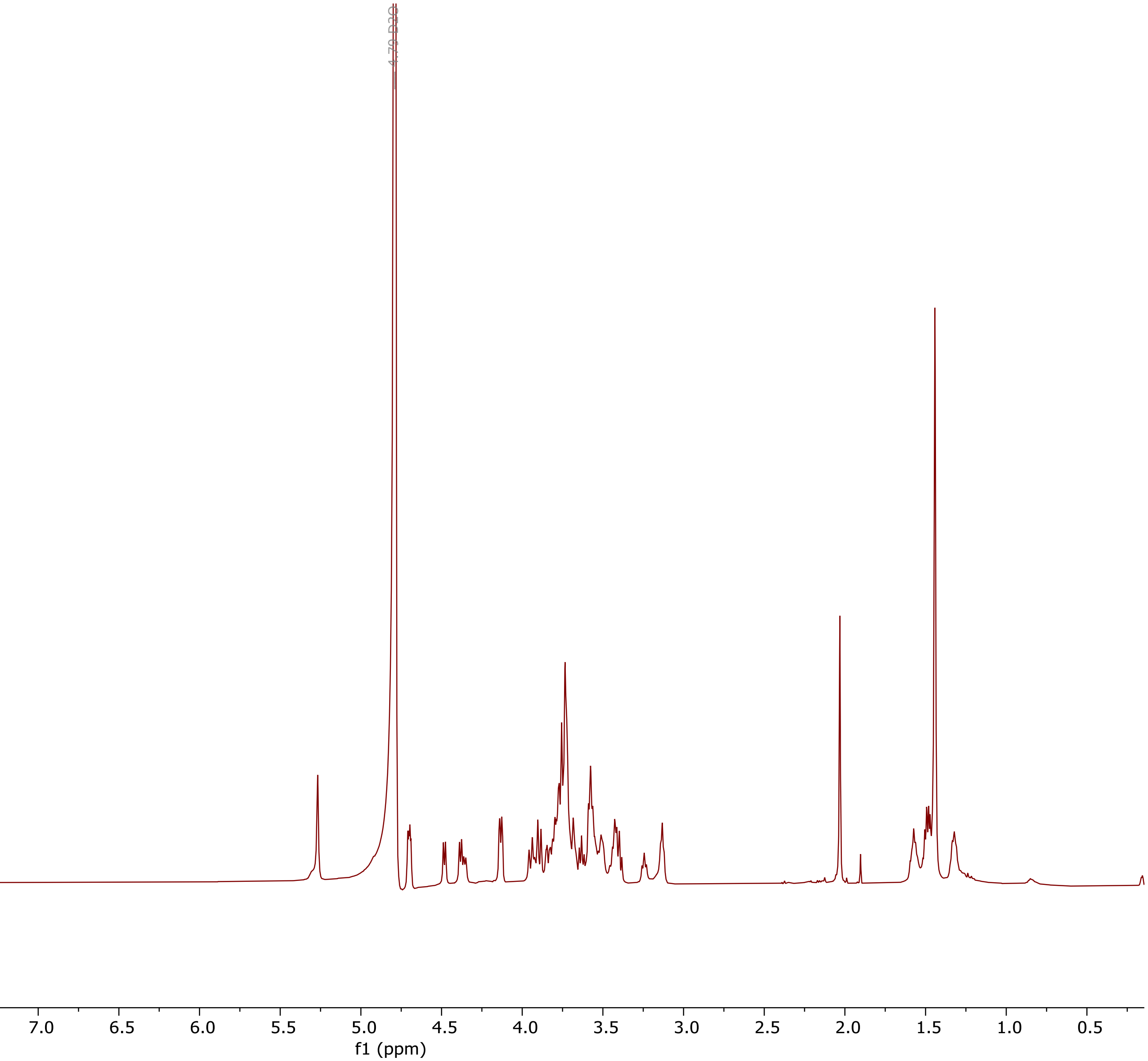

gCOSY NMR of Compound **18**

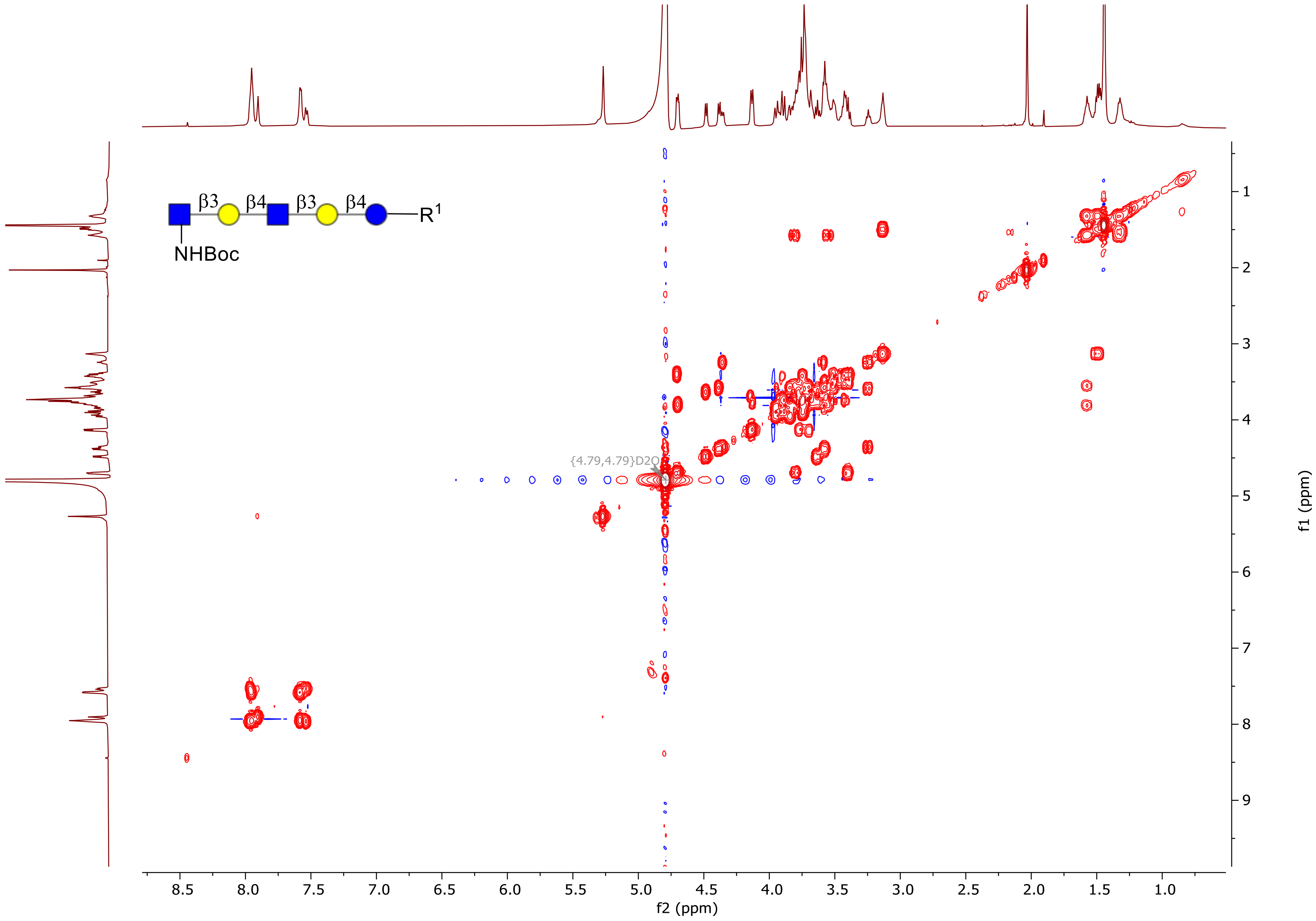

Multiplicity edited gHSQC NMR of Compound **18**

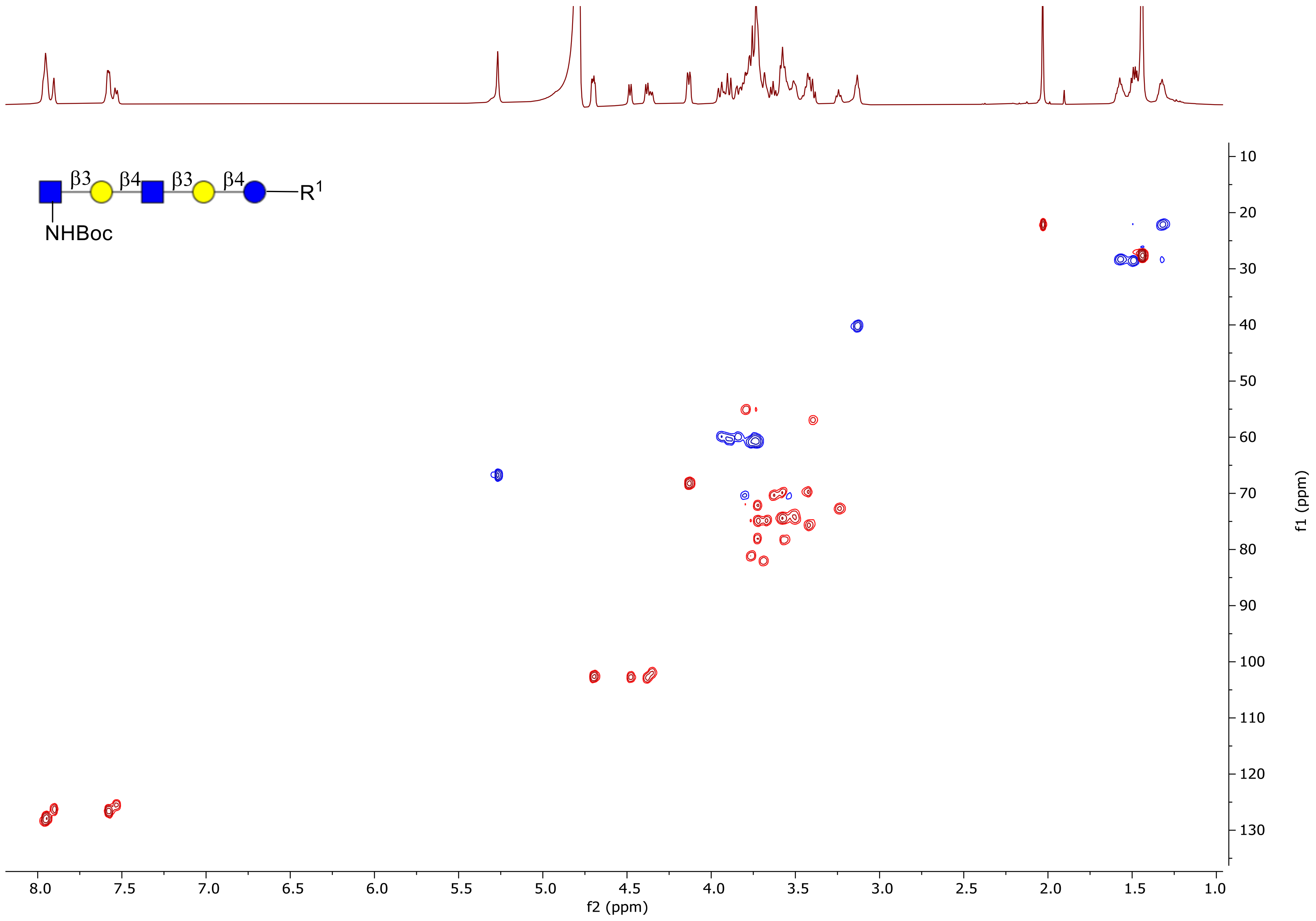

TOCSY-DIPSI NMR of Compound **18**

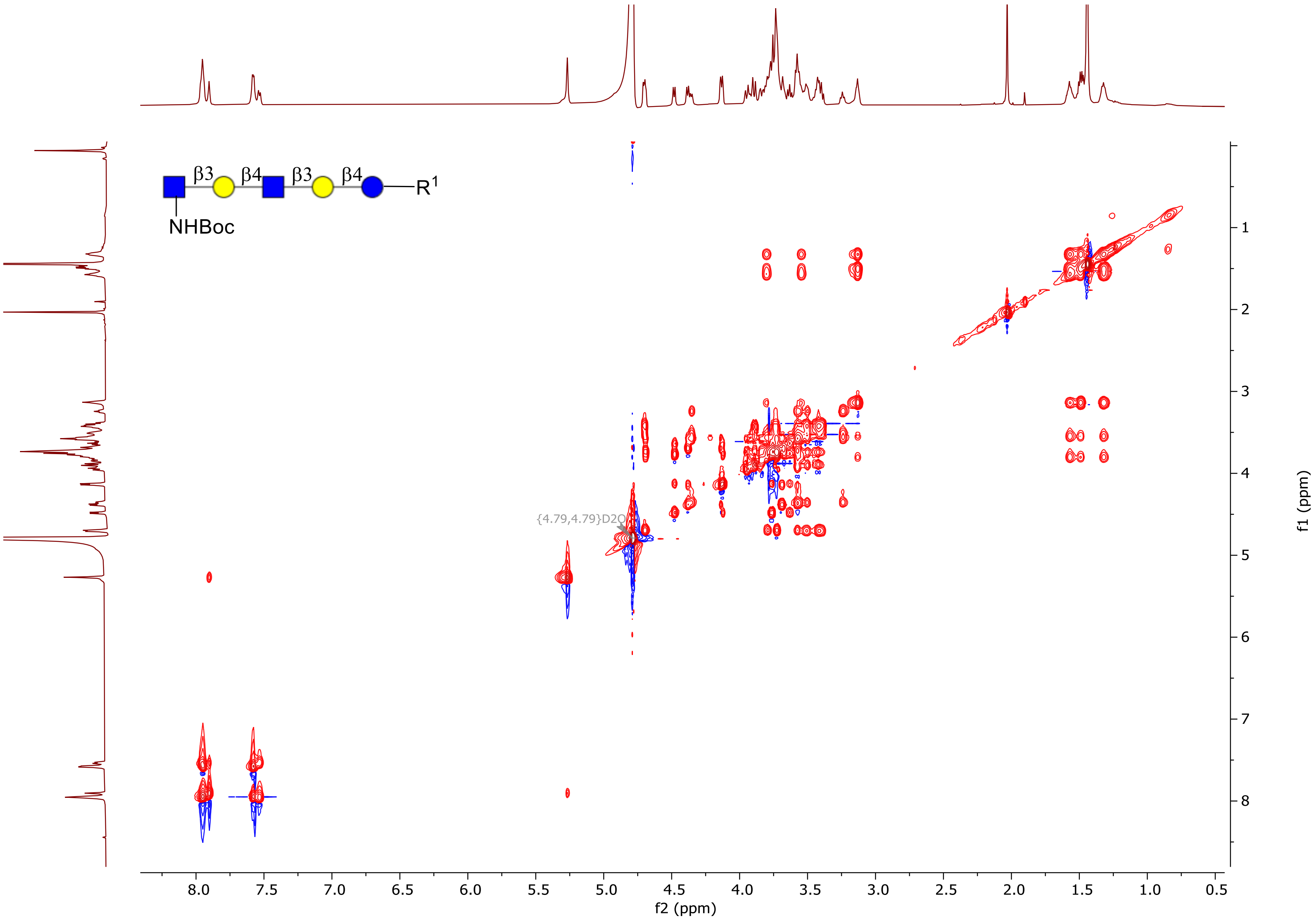

^{1}H NMR of Compound **19**

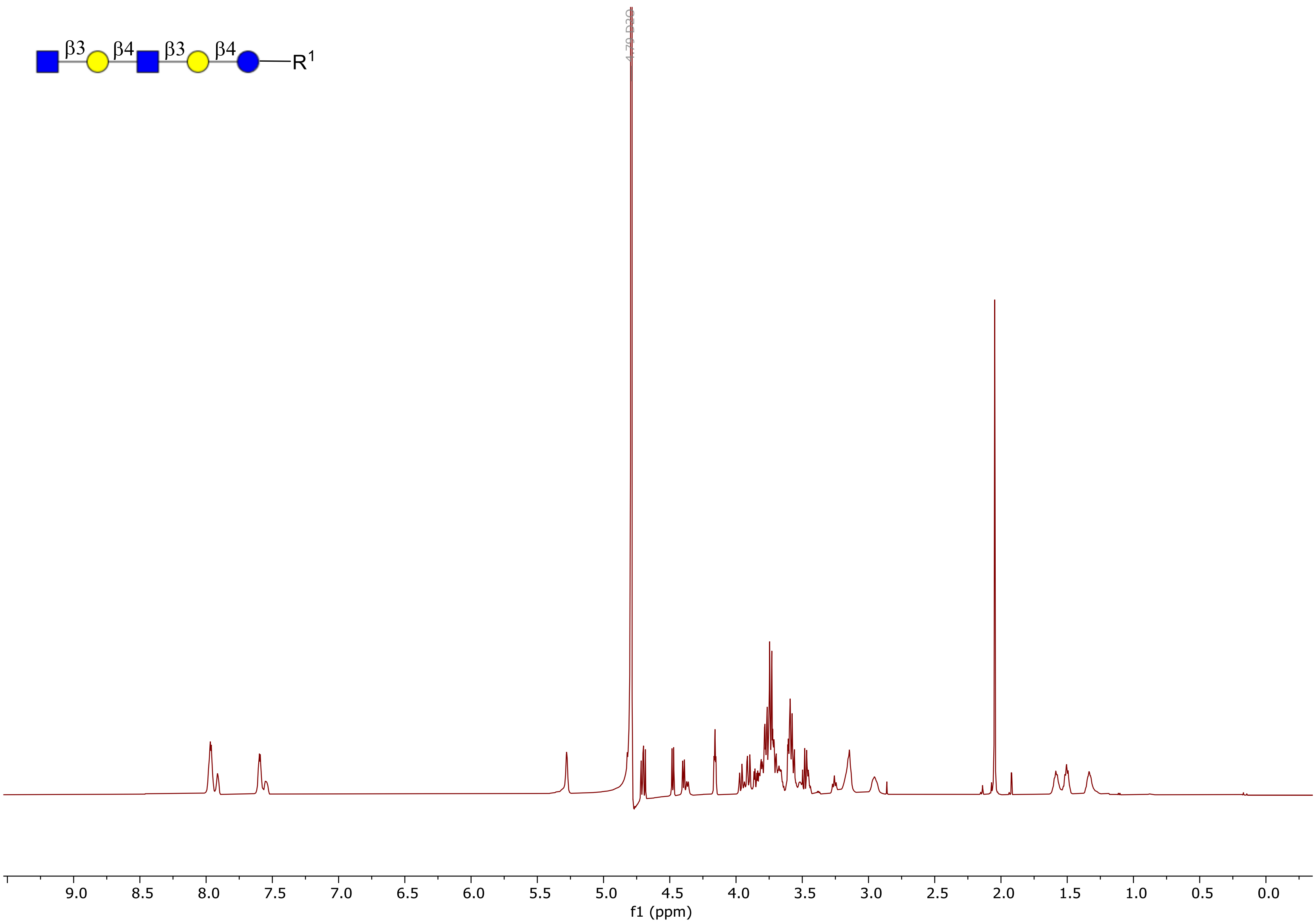

gCOSY NMR of Compound **19**

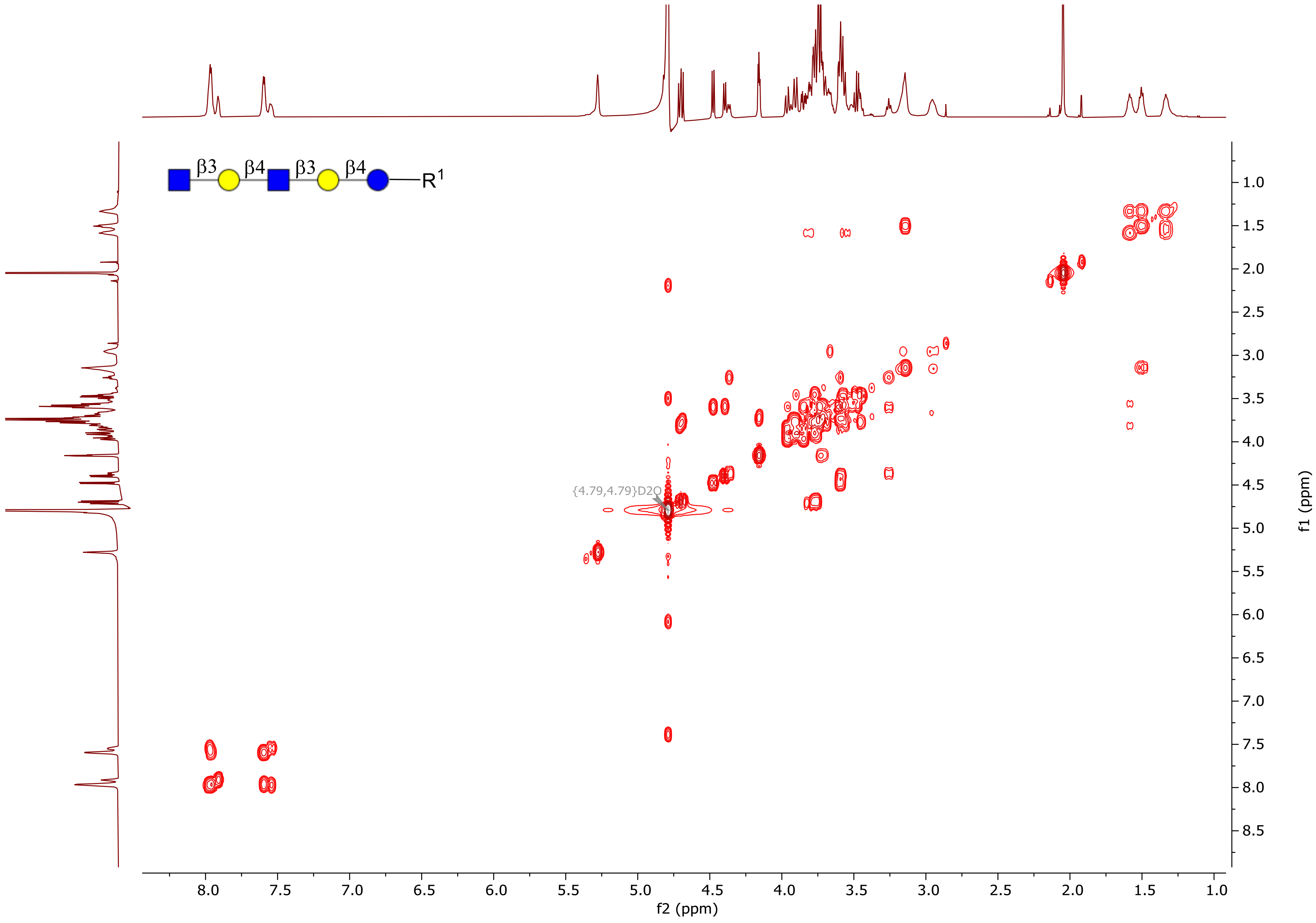

Multiplicity edited gHSQC NMR of Compound **19**

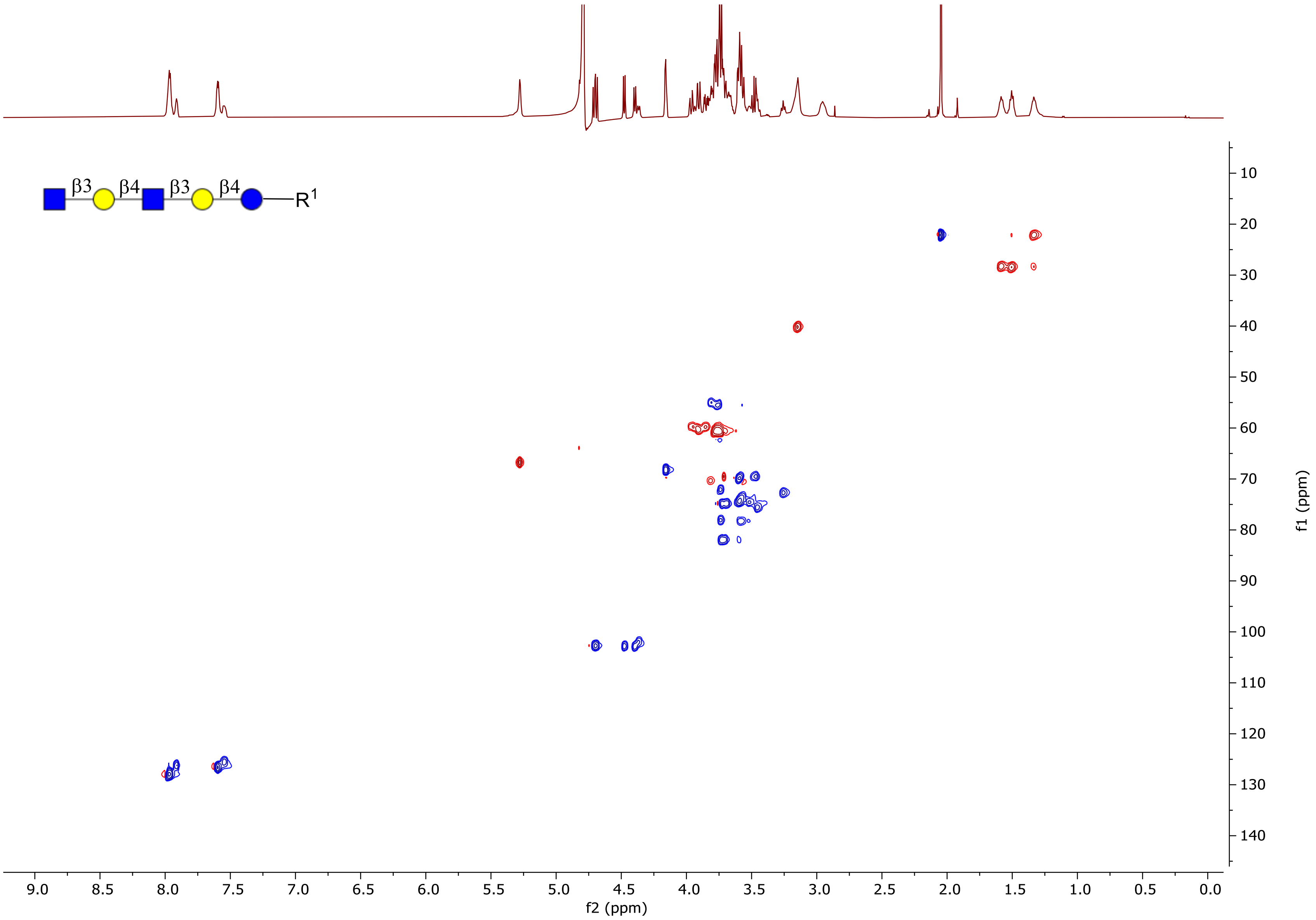

TOCSY-DIPSI NMR of Compound **19**

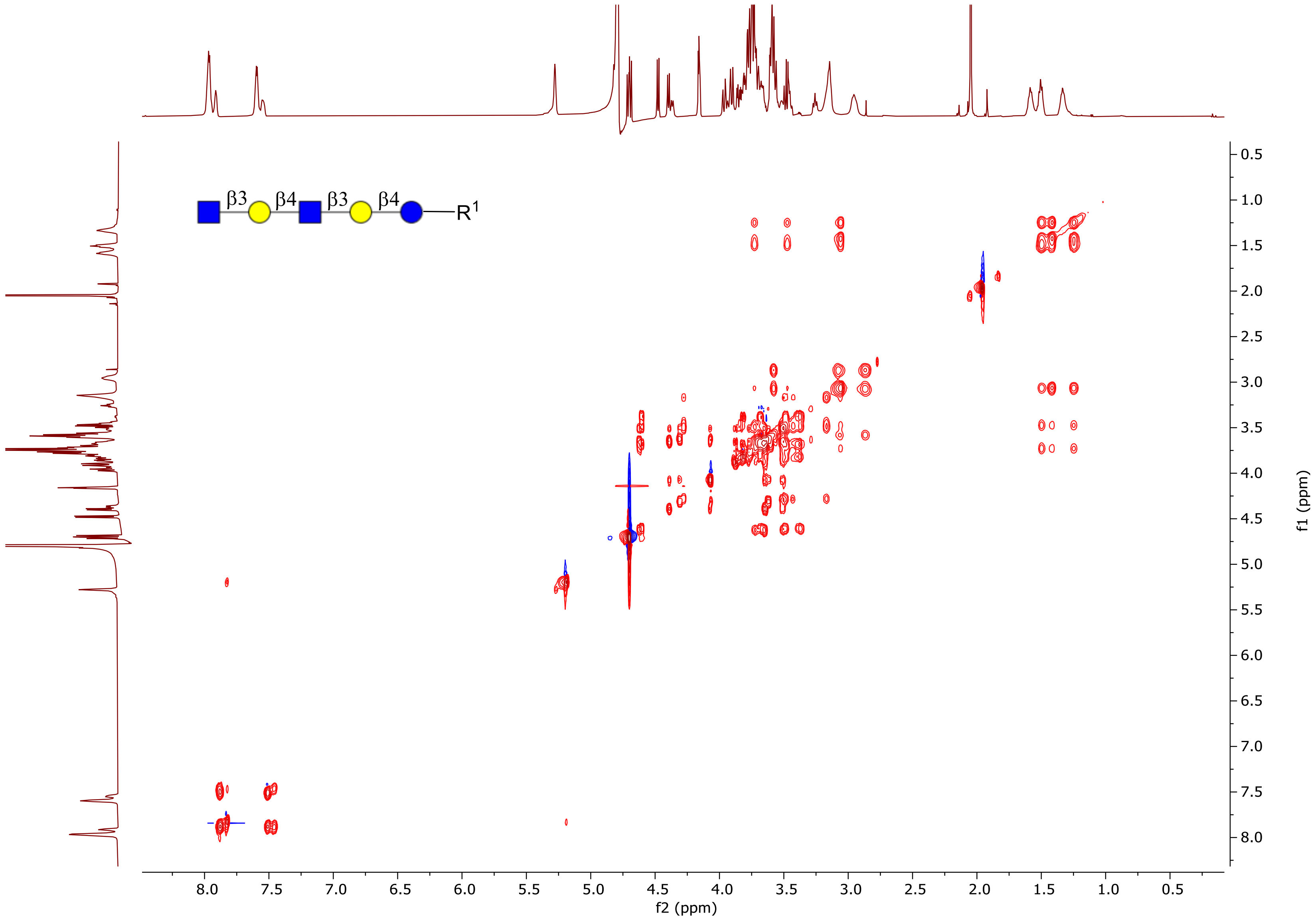

NOESY NMR of Compound **19**

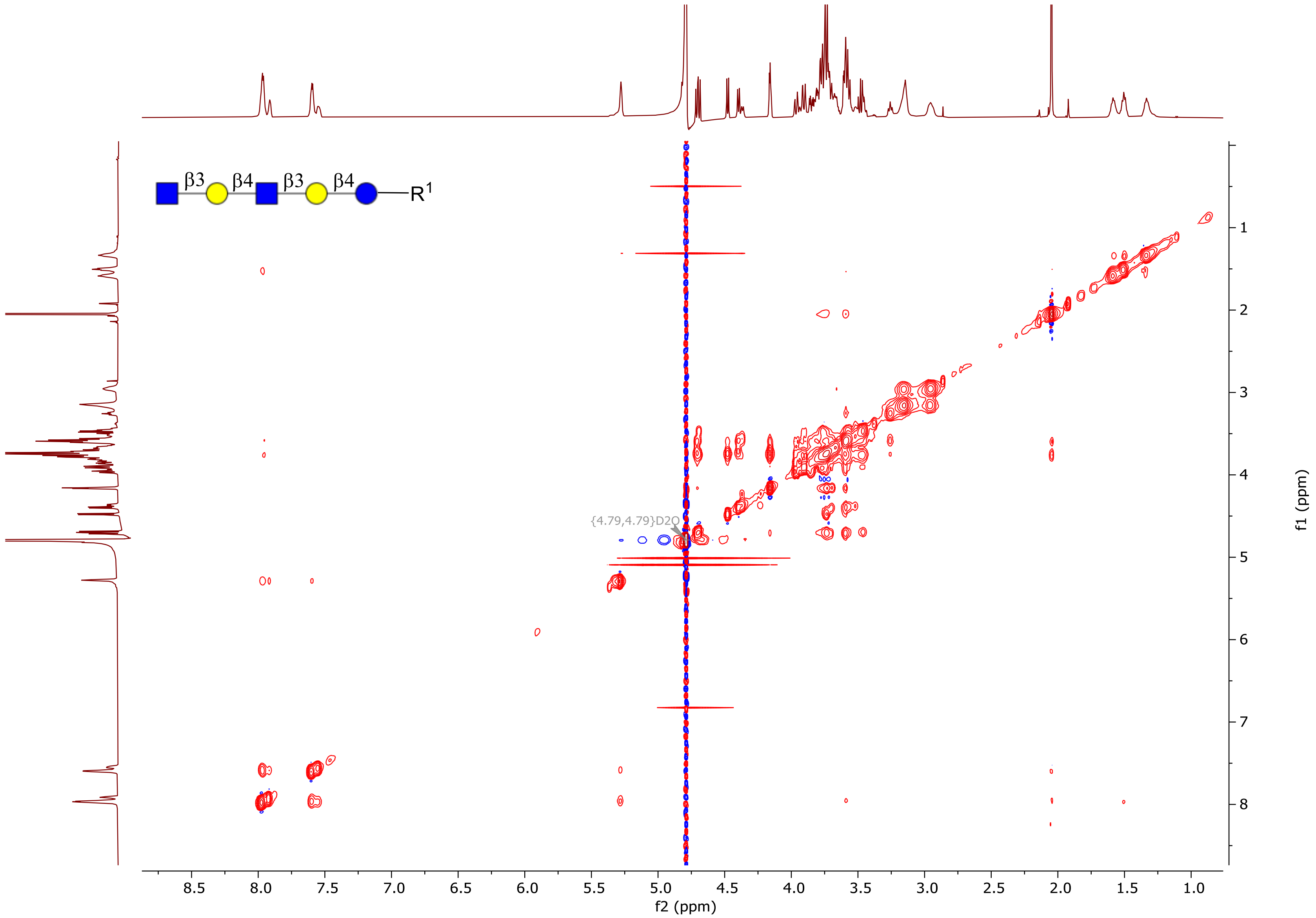

HMBC NMR of Compound **19**

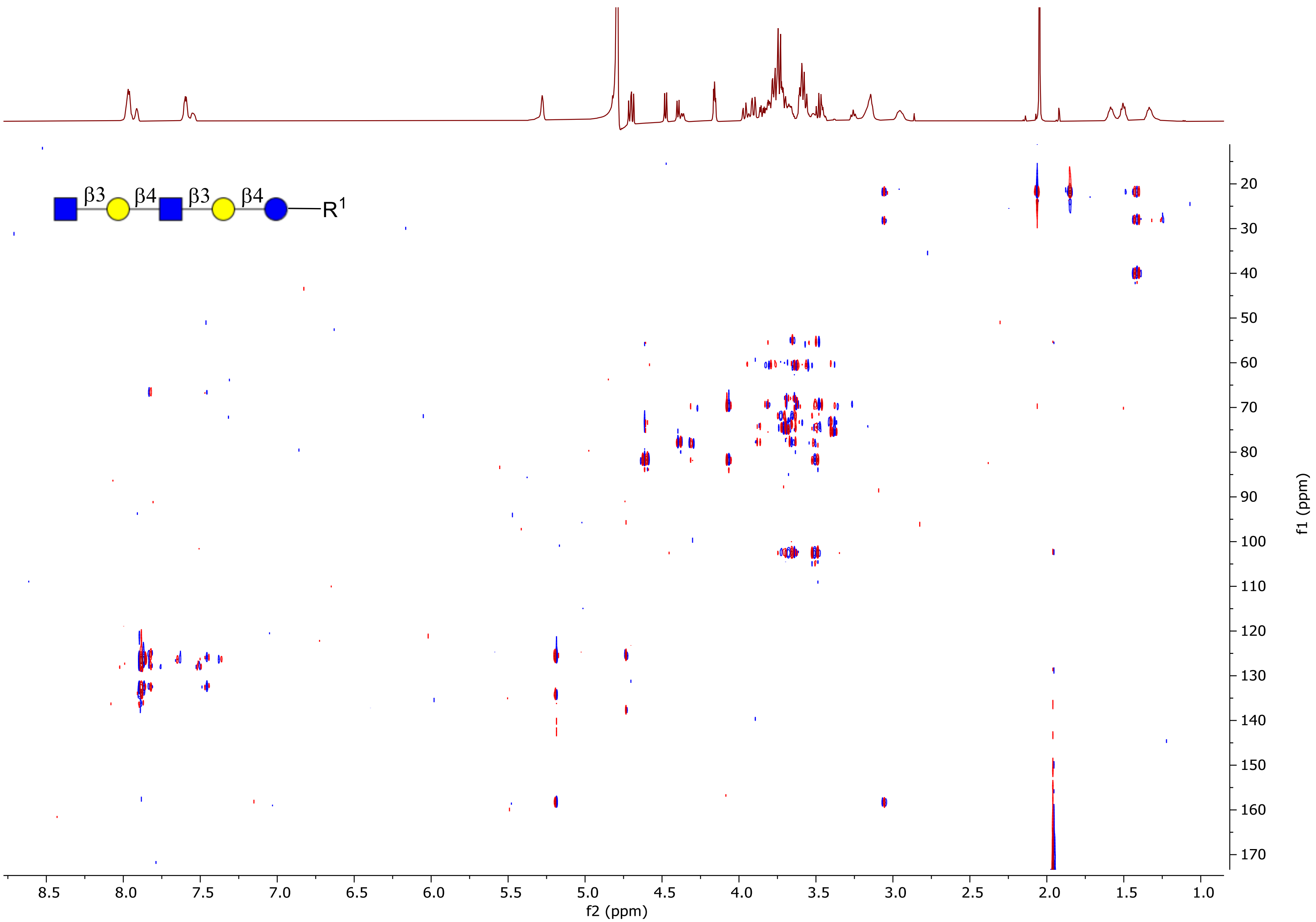

^{1}H NMR of Compound **23**

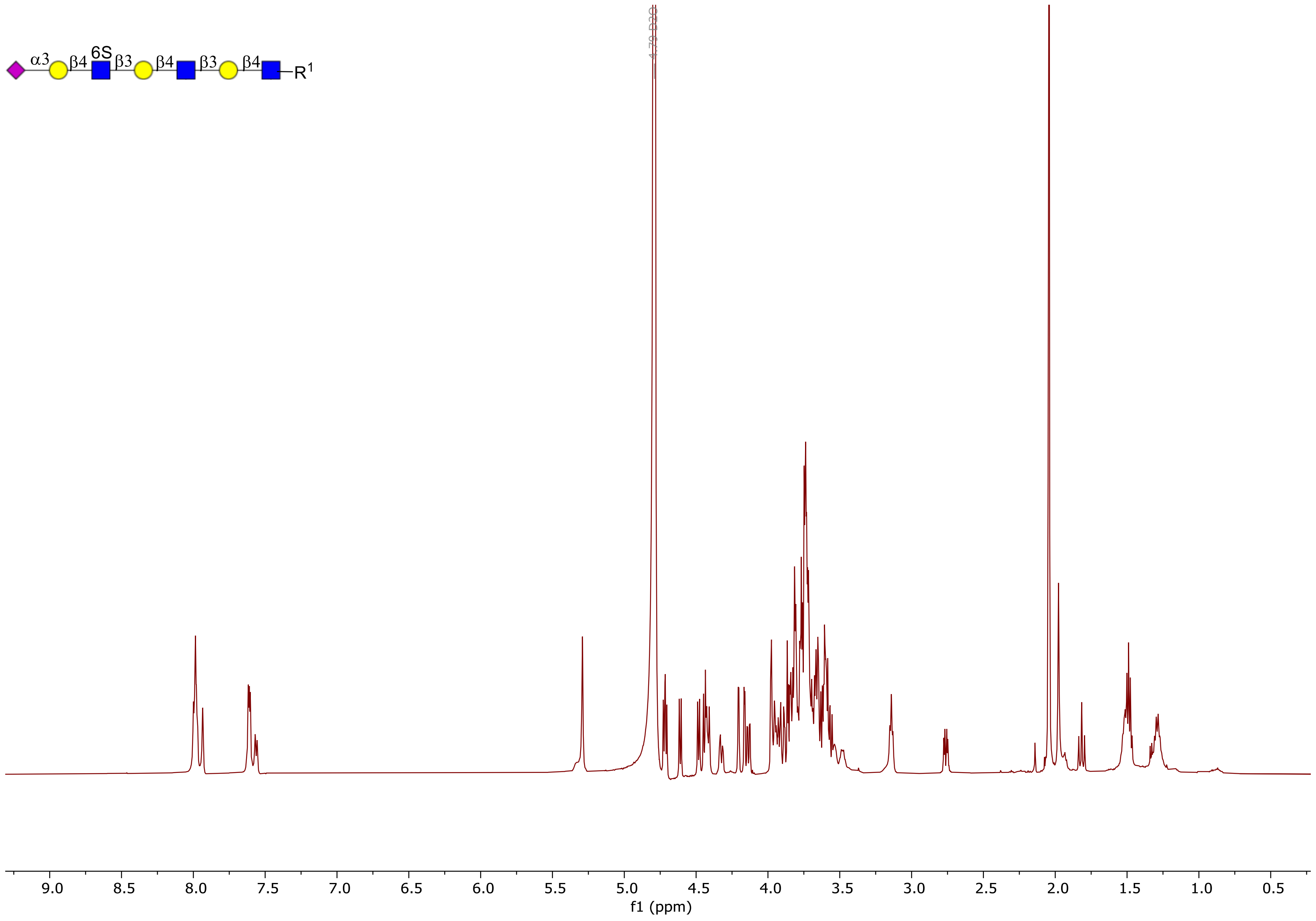

gCOSY NMR of Compound **23**

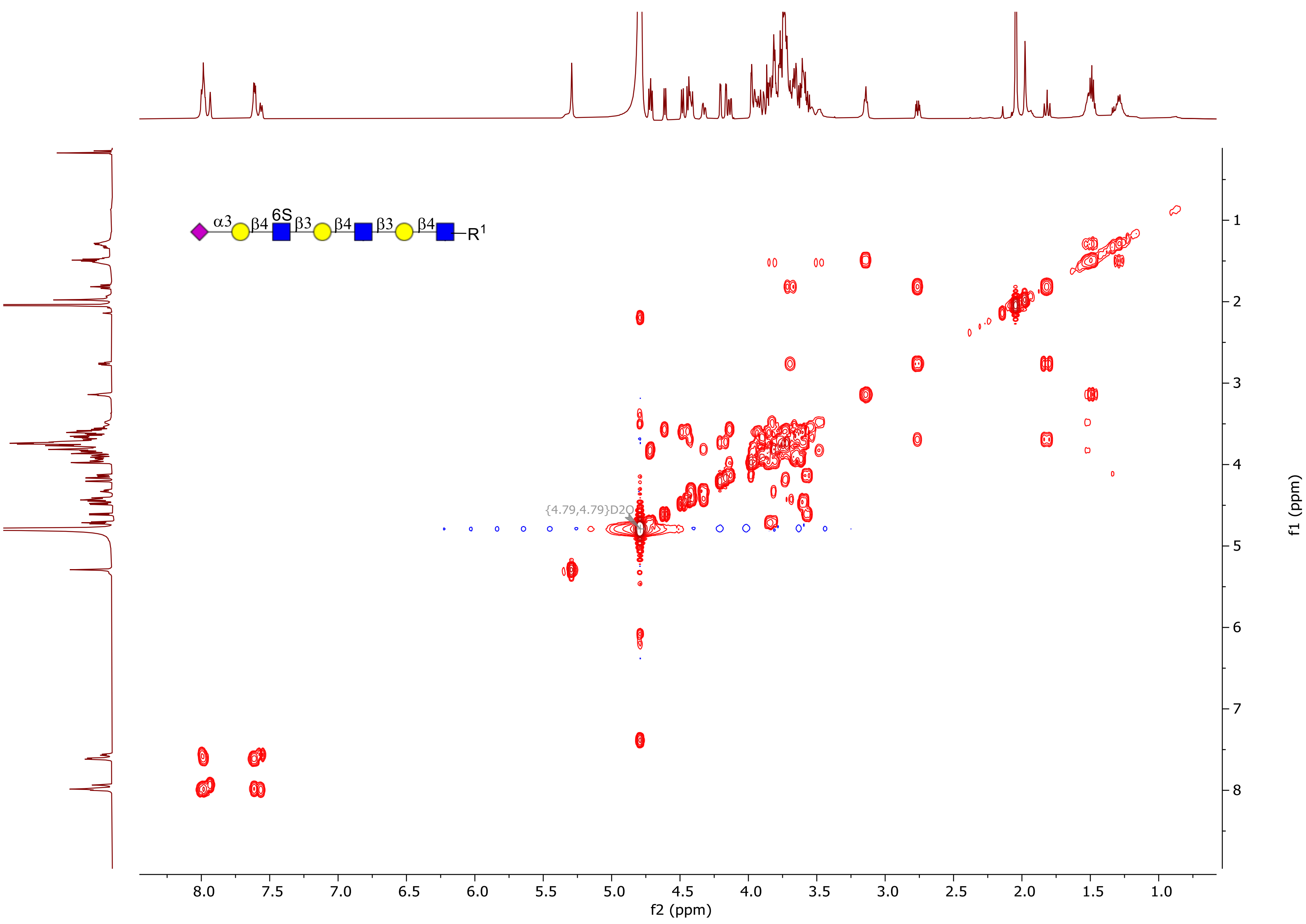

Multiplicity edited gHSQC NMR of Compound **23**

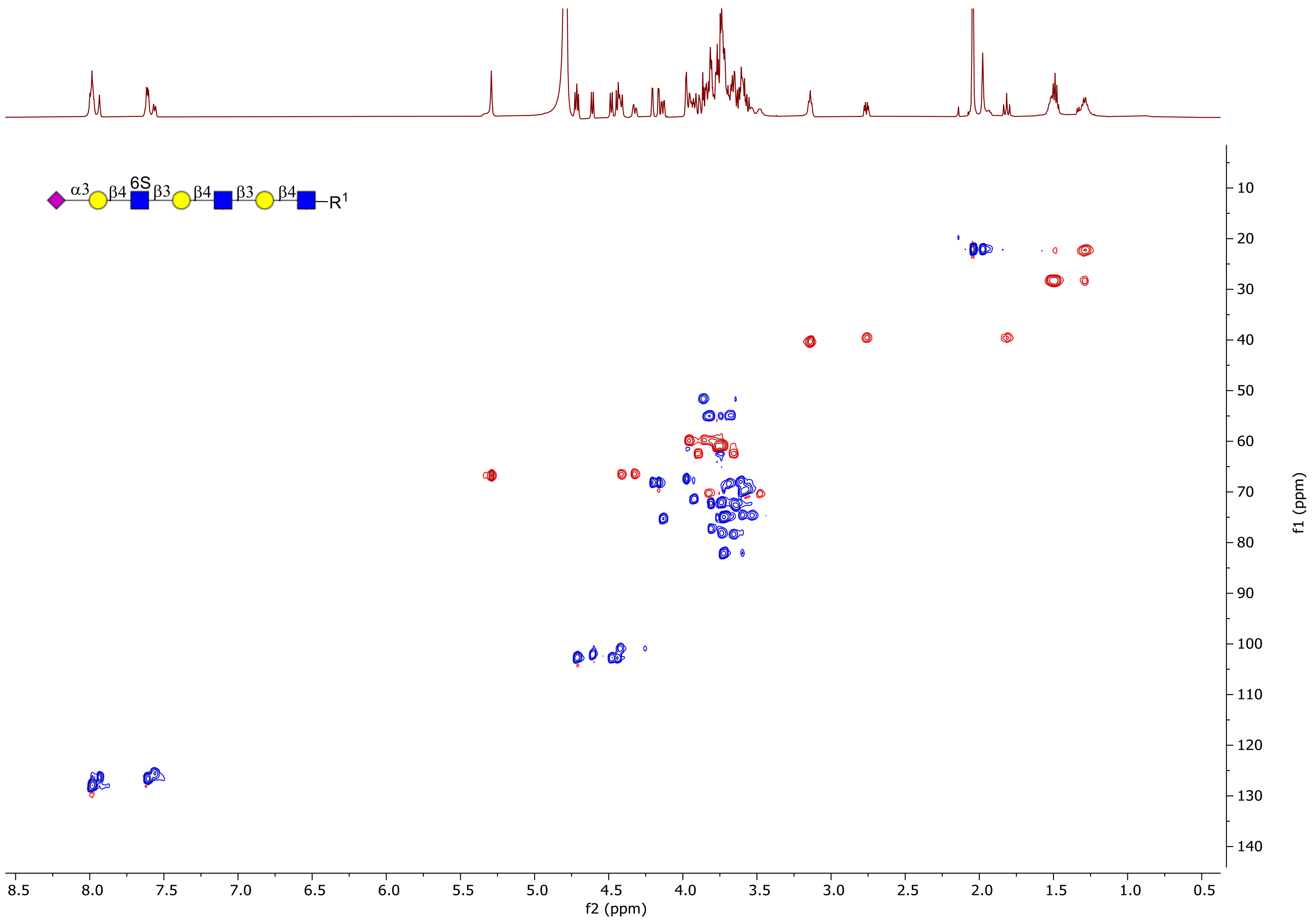

TOCSY-DIPSI NMR of Compound **23**

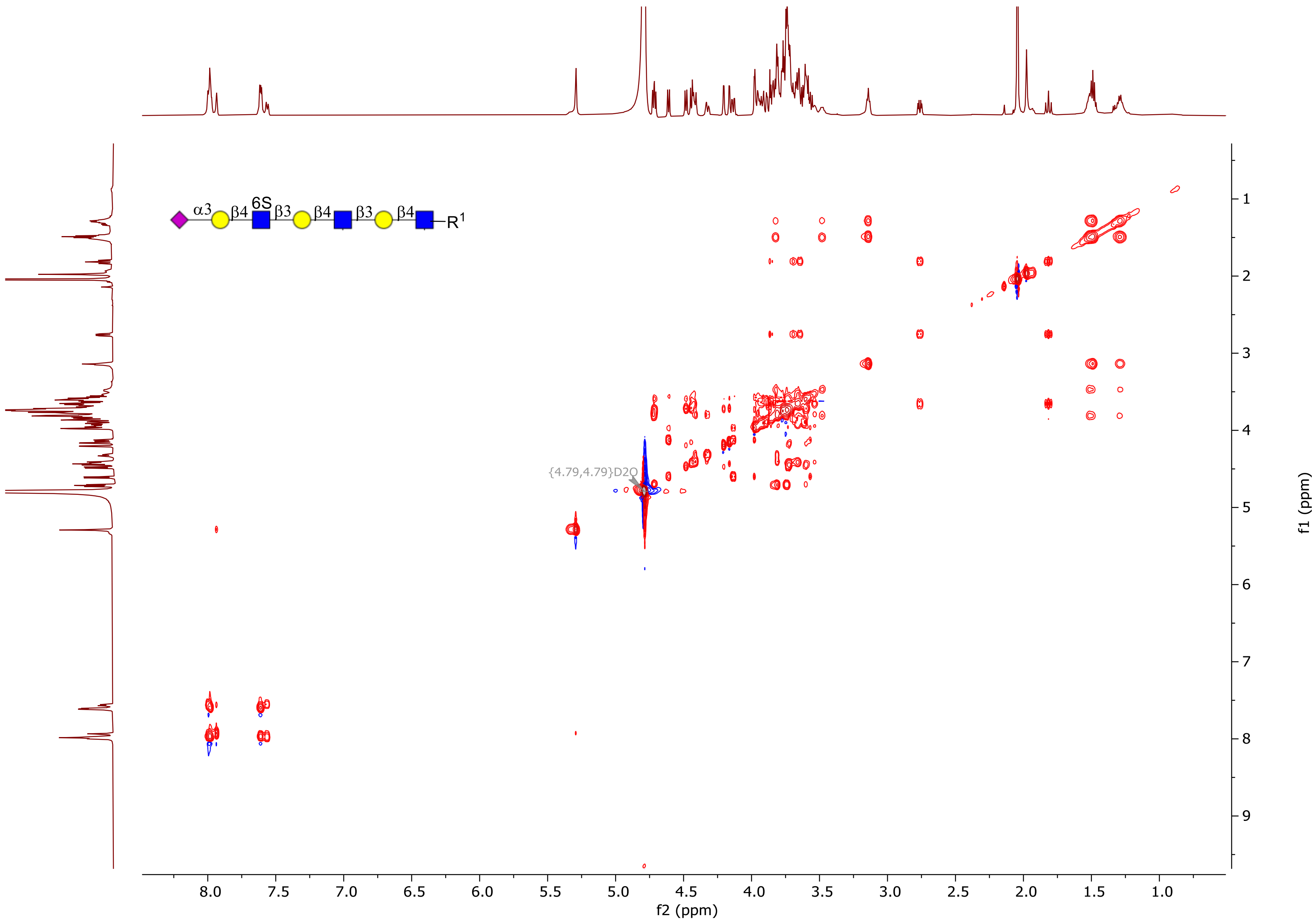

NOESY NMR of Compound **23**

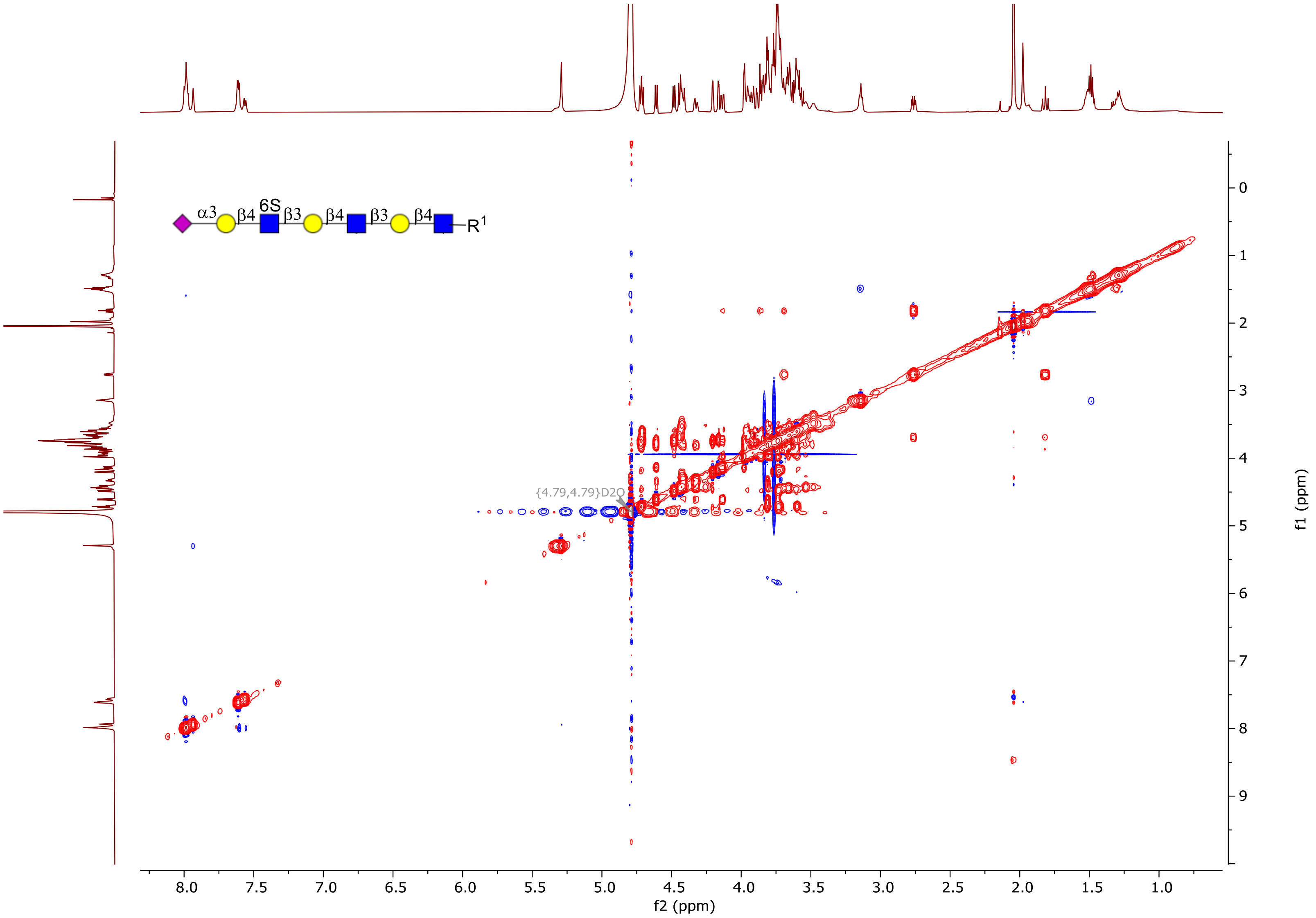

HMBC NMR of Compound **23**

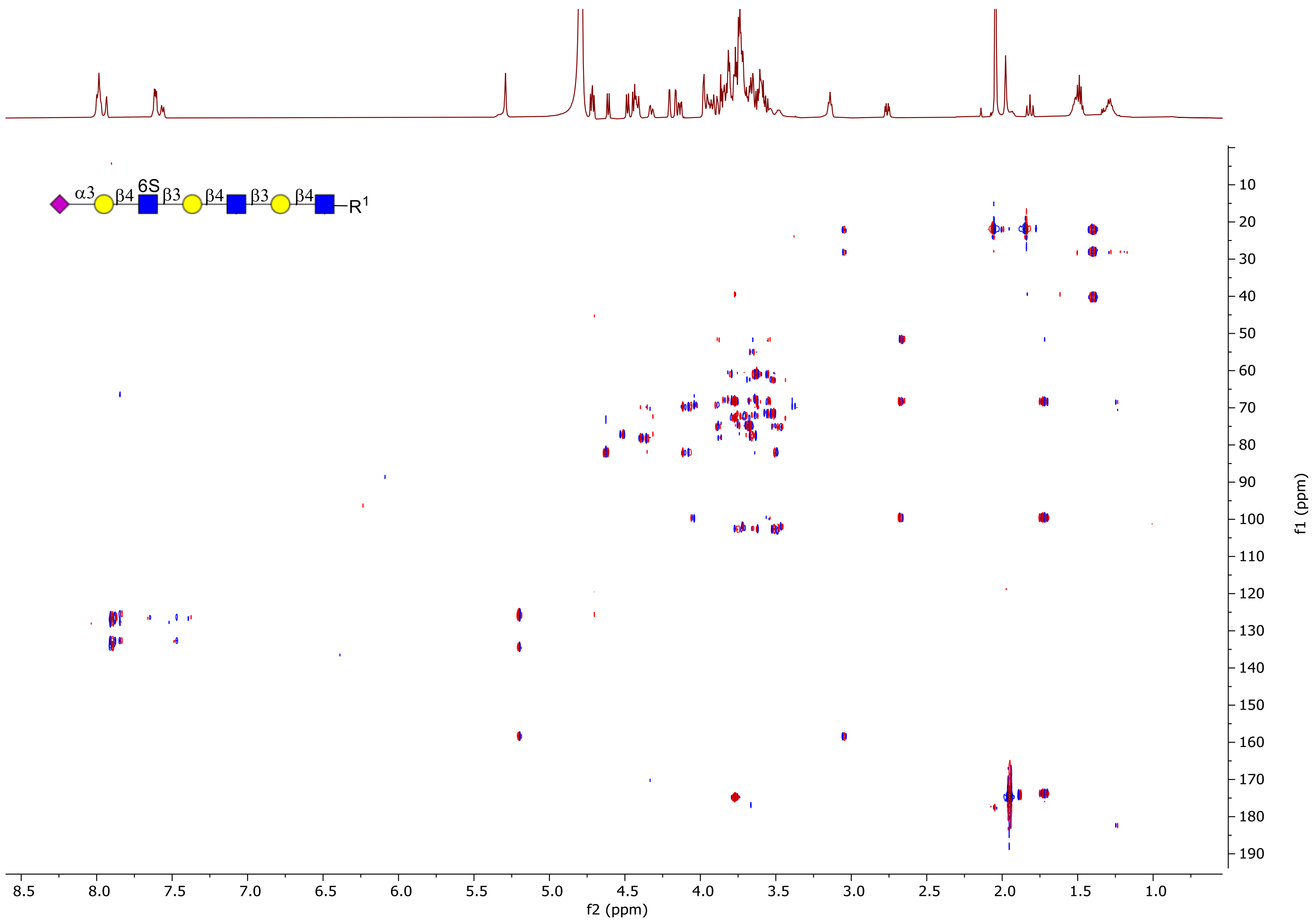

^{1}H NMR of Compound **26**

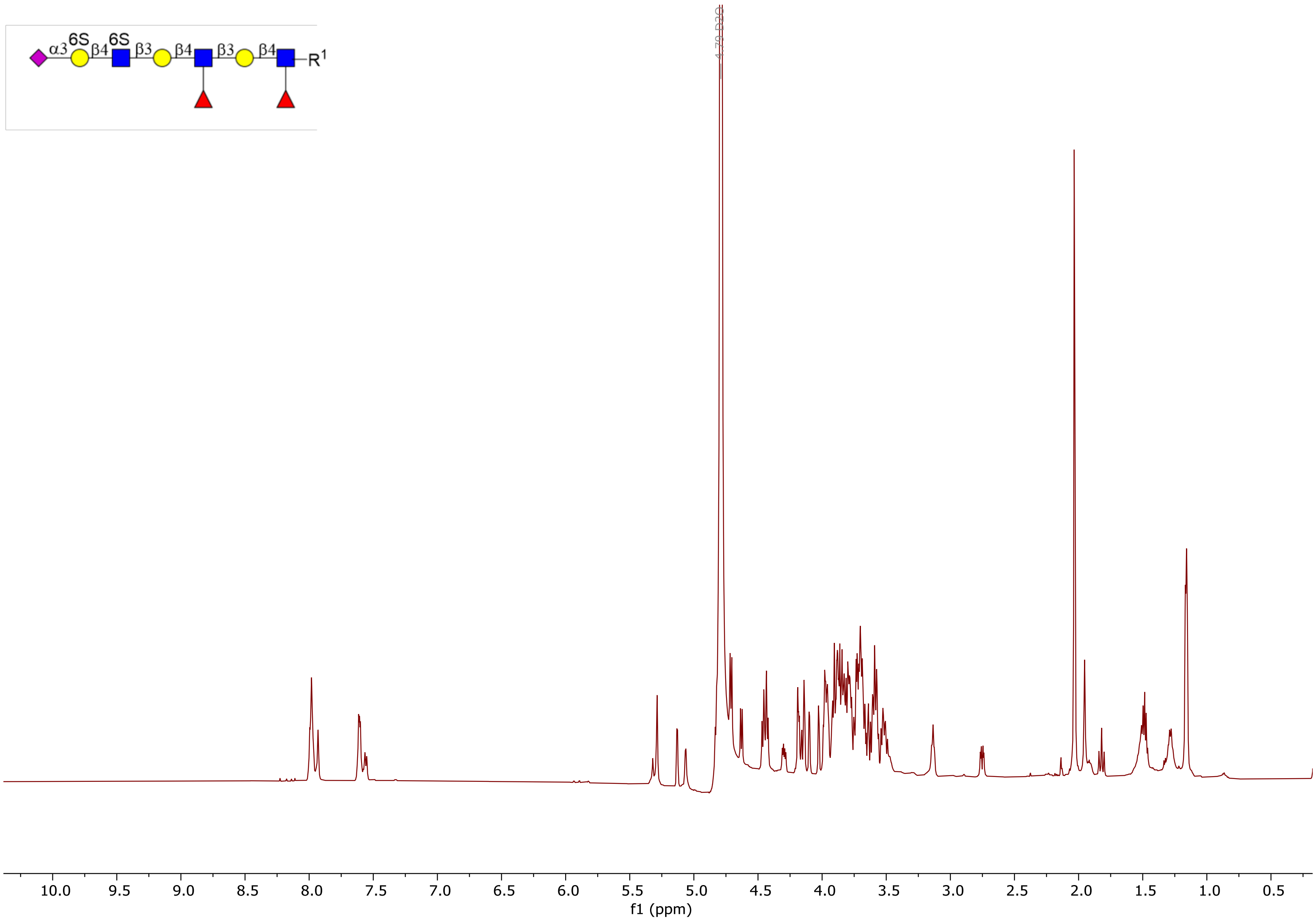

gCOSY NMR of Compound **26**

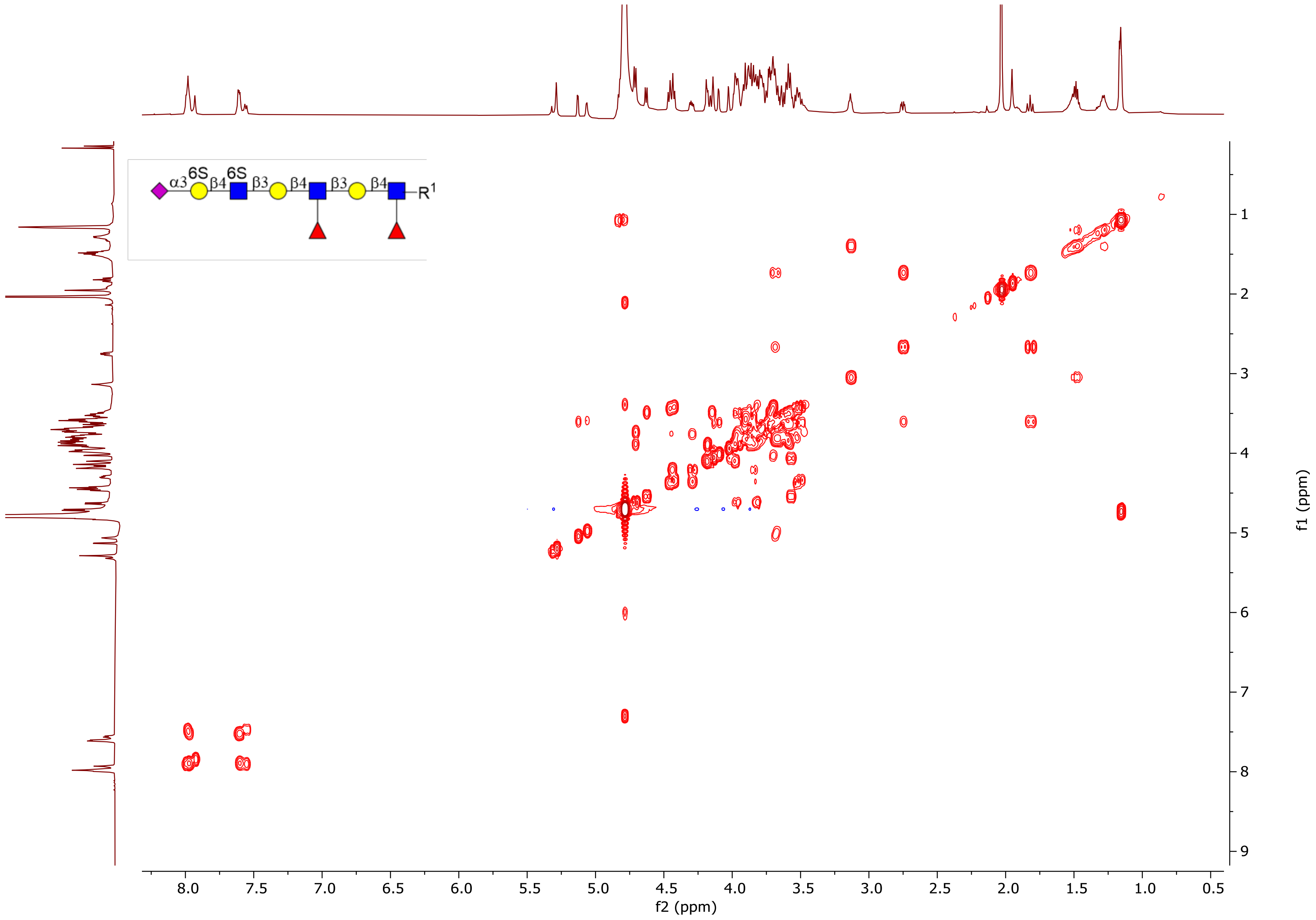

Multiplicity edited gHSQC NMR of Compound **26**

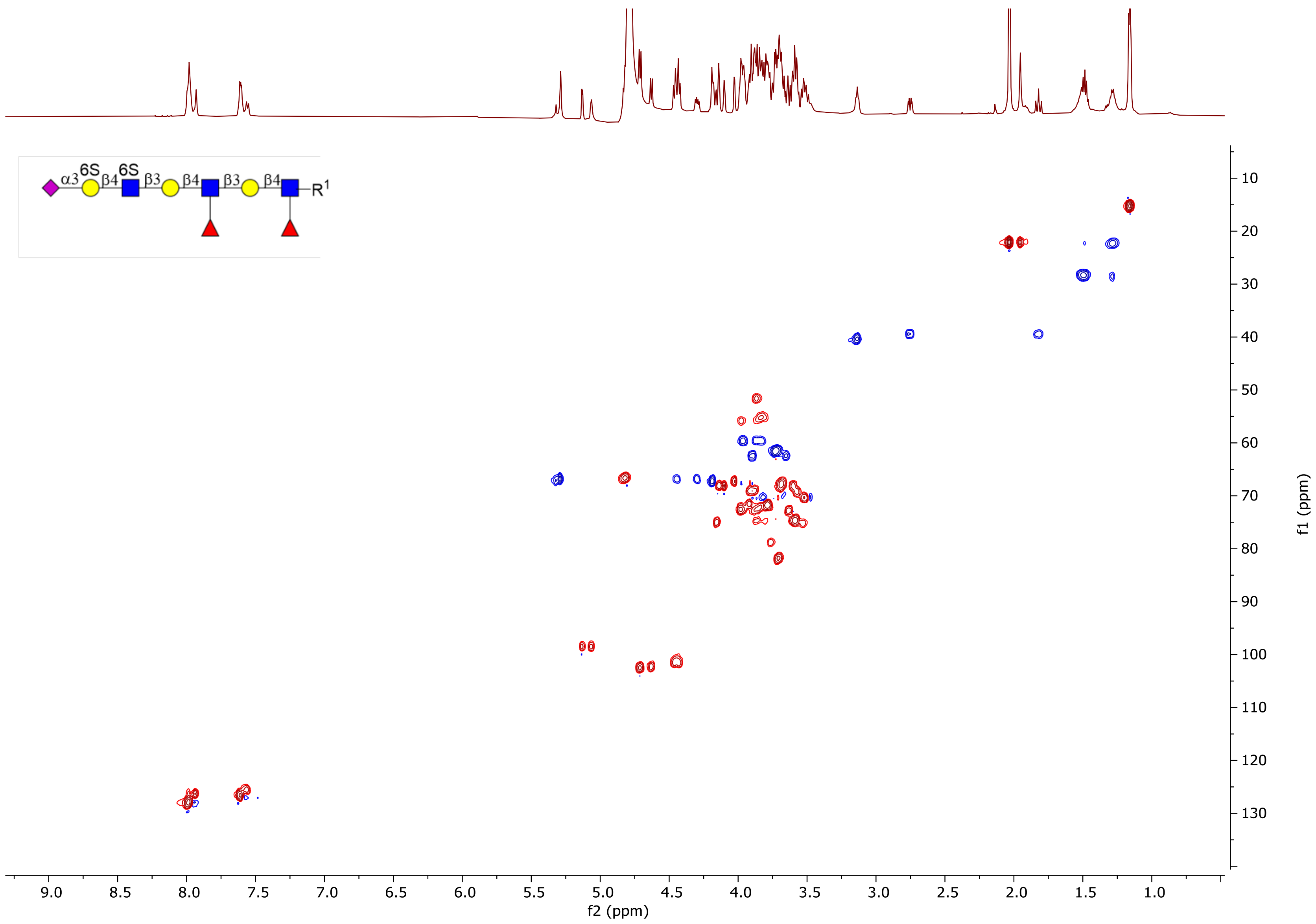

TOCSY-DIPSI NMR of Compound **26**

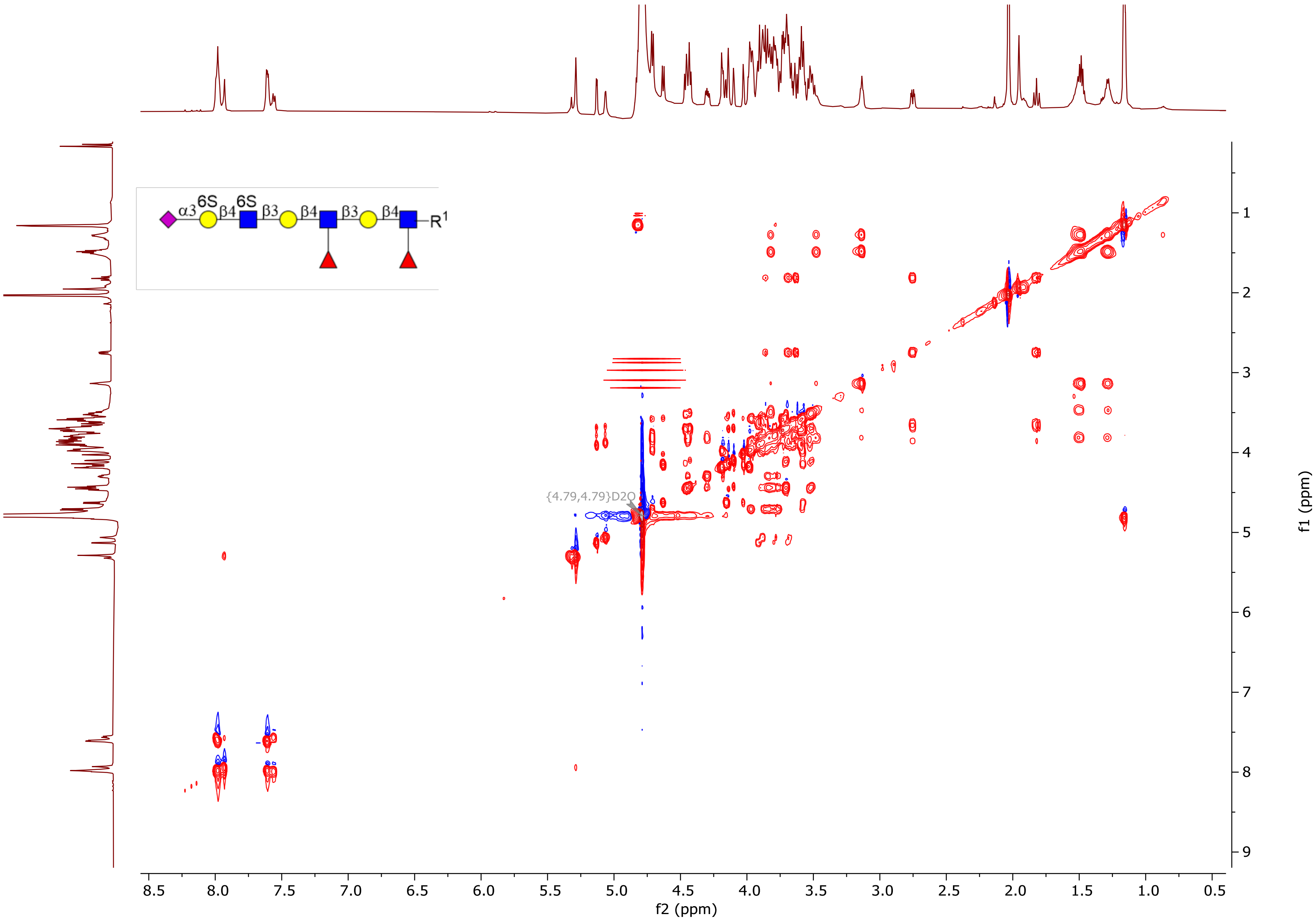

NOESY NMR of Compound **26**

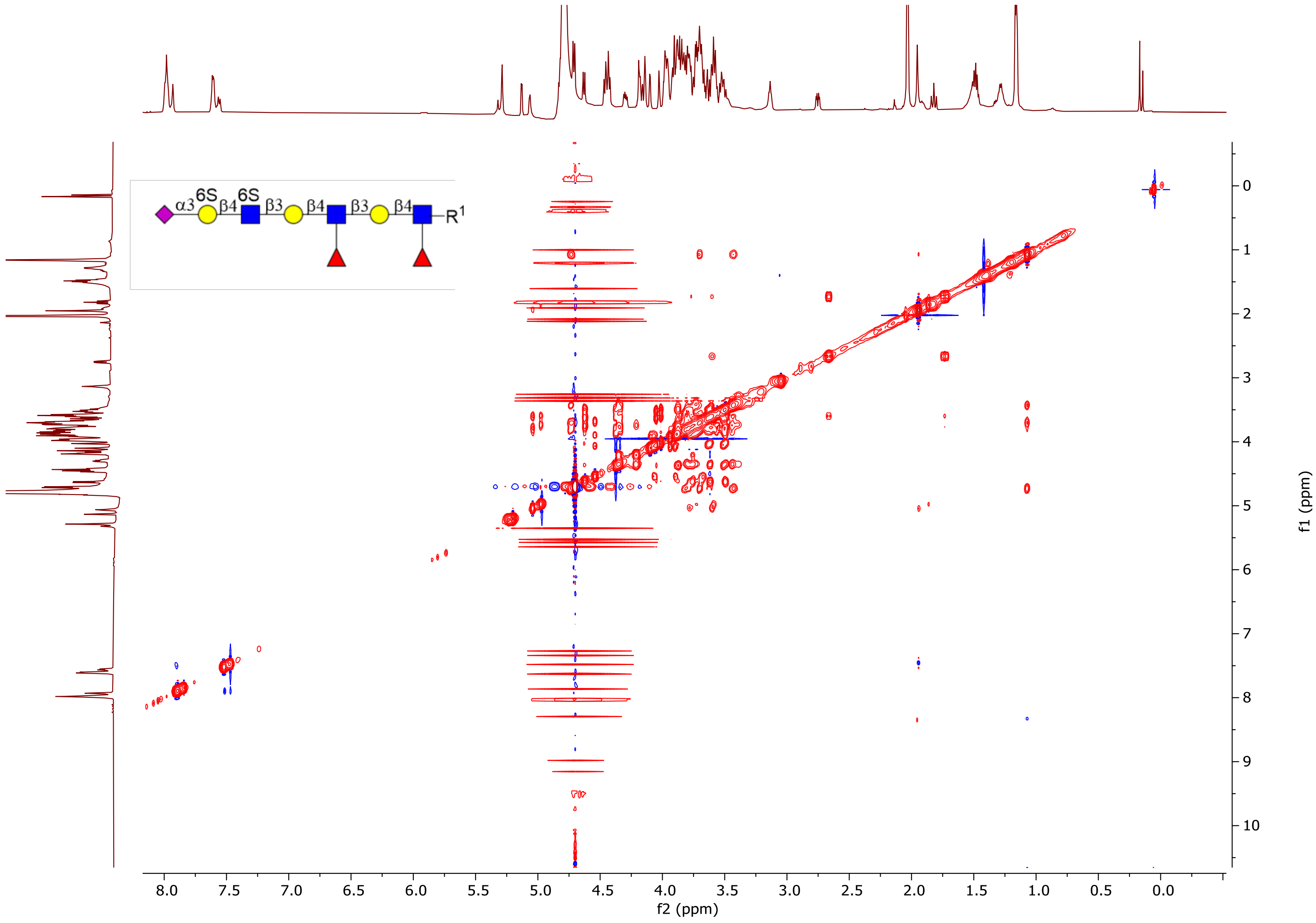

HMBC NMR of Compound **26**

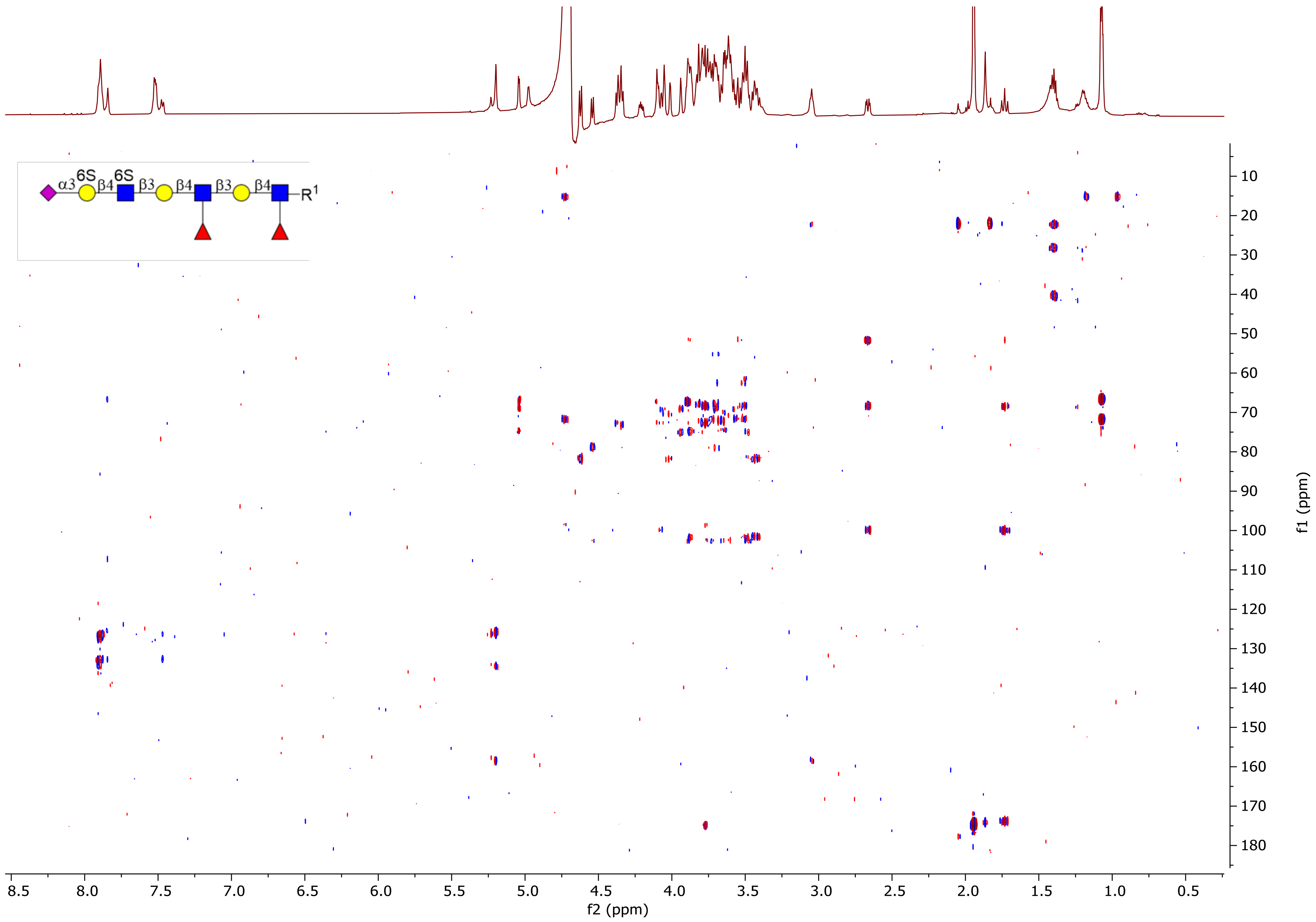

^{1}H NMR of Compound **27**

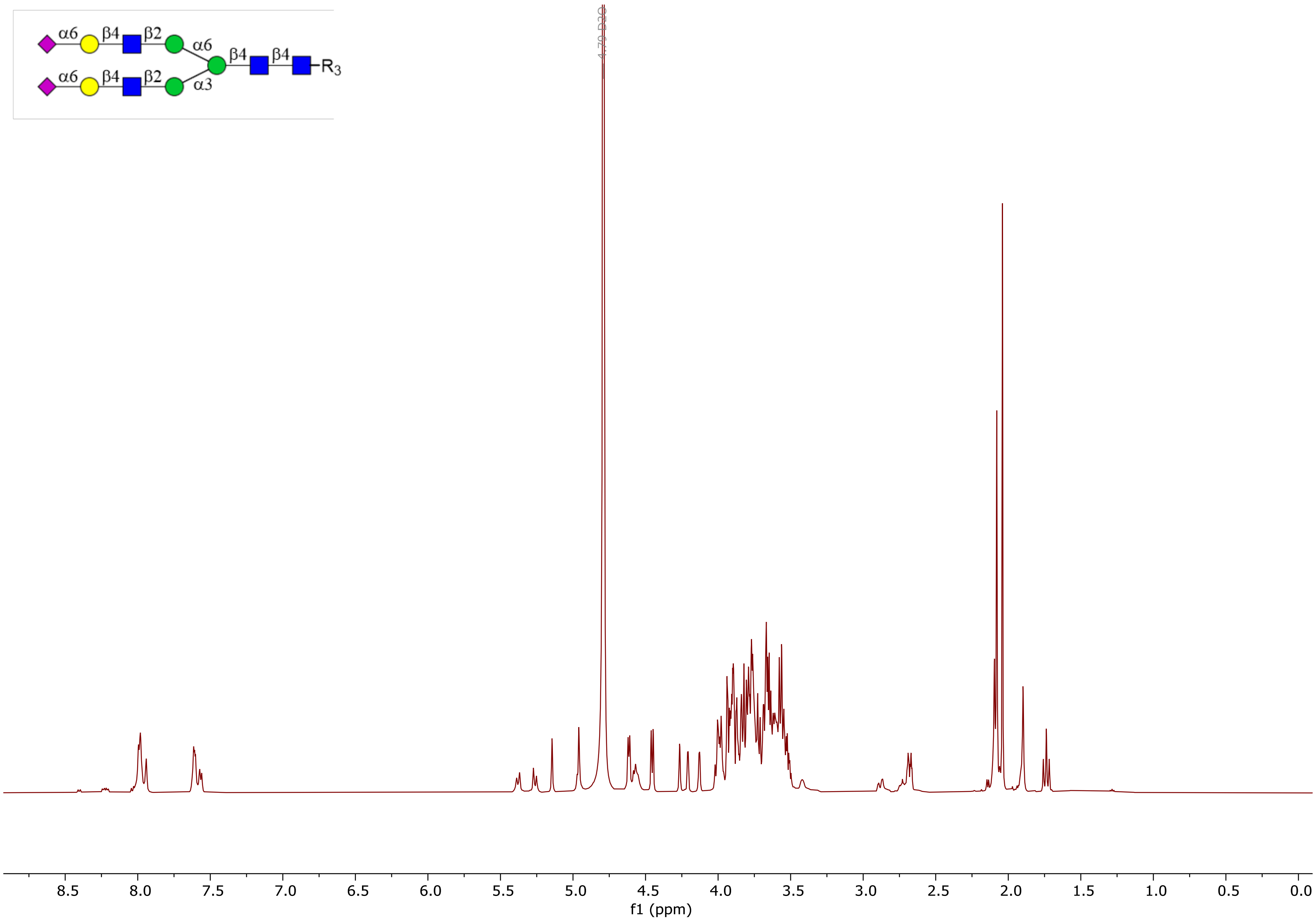

gCOSY NMR of Compound **27**

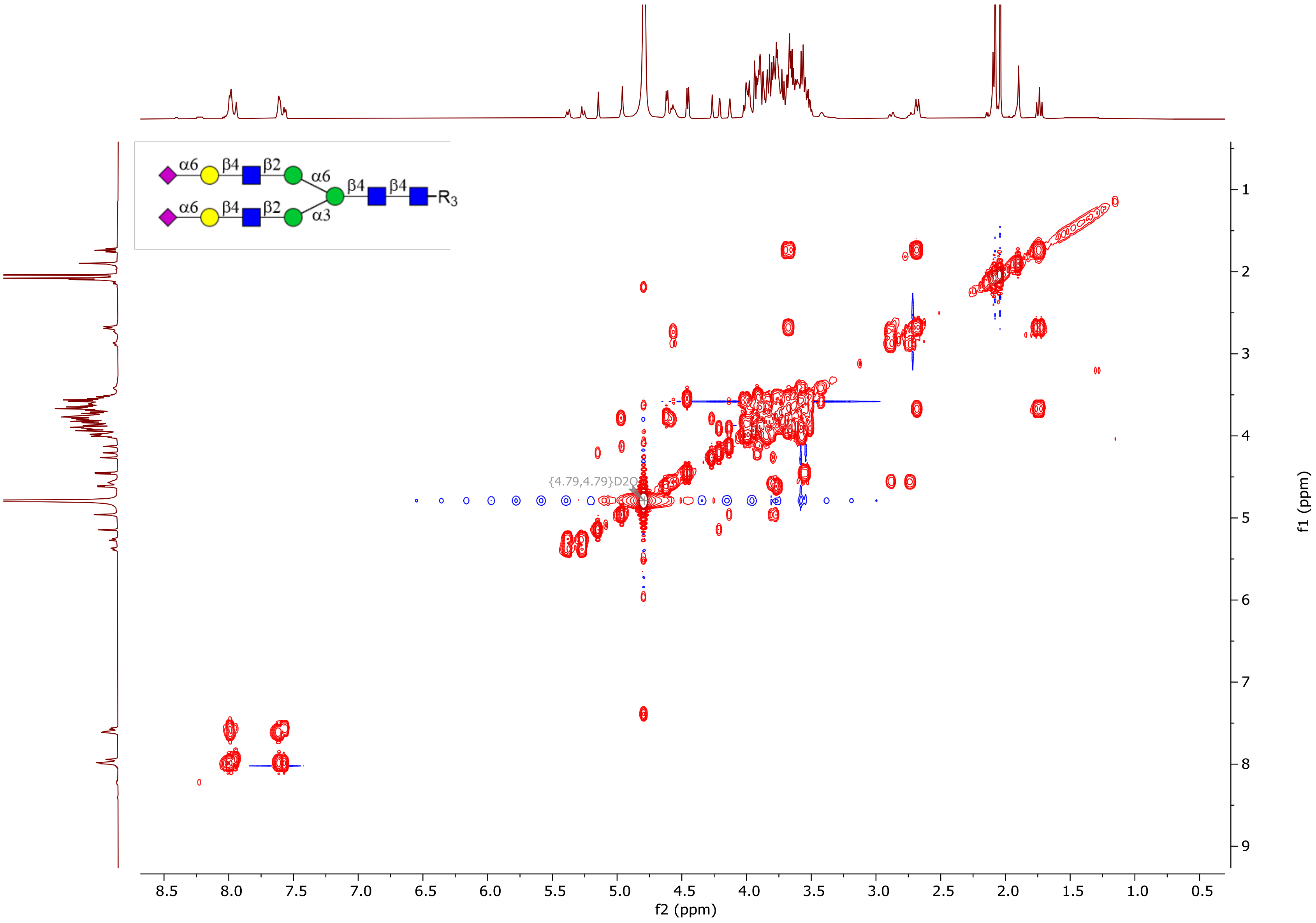

Multiplicity edited gHSQC NMR of Compound **27**

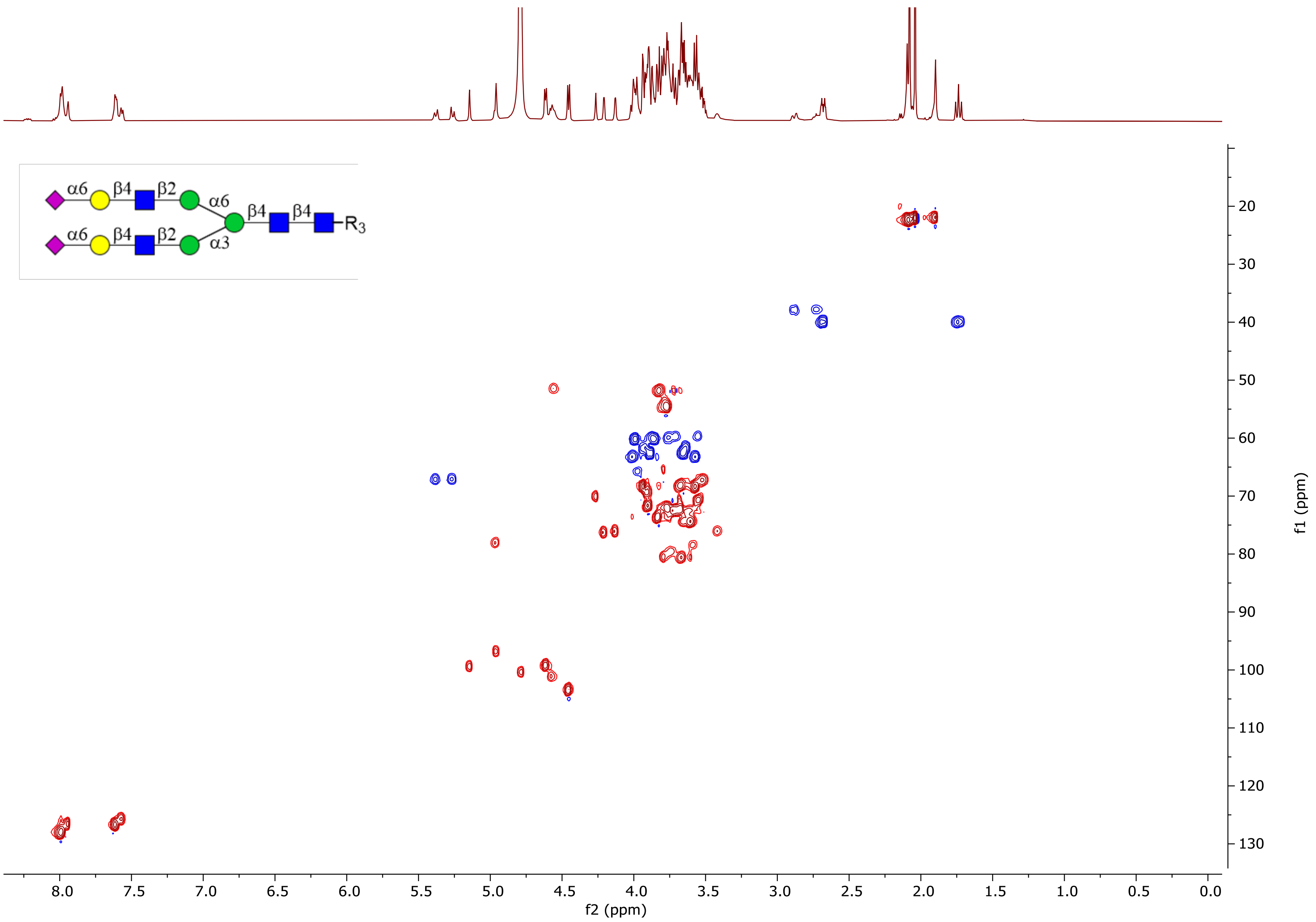

TOCSY-DIPSI NMR of Compound **27**

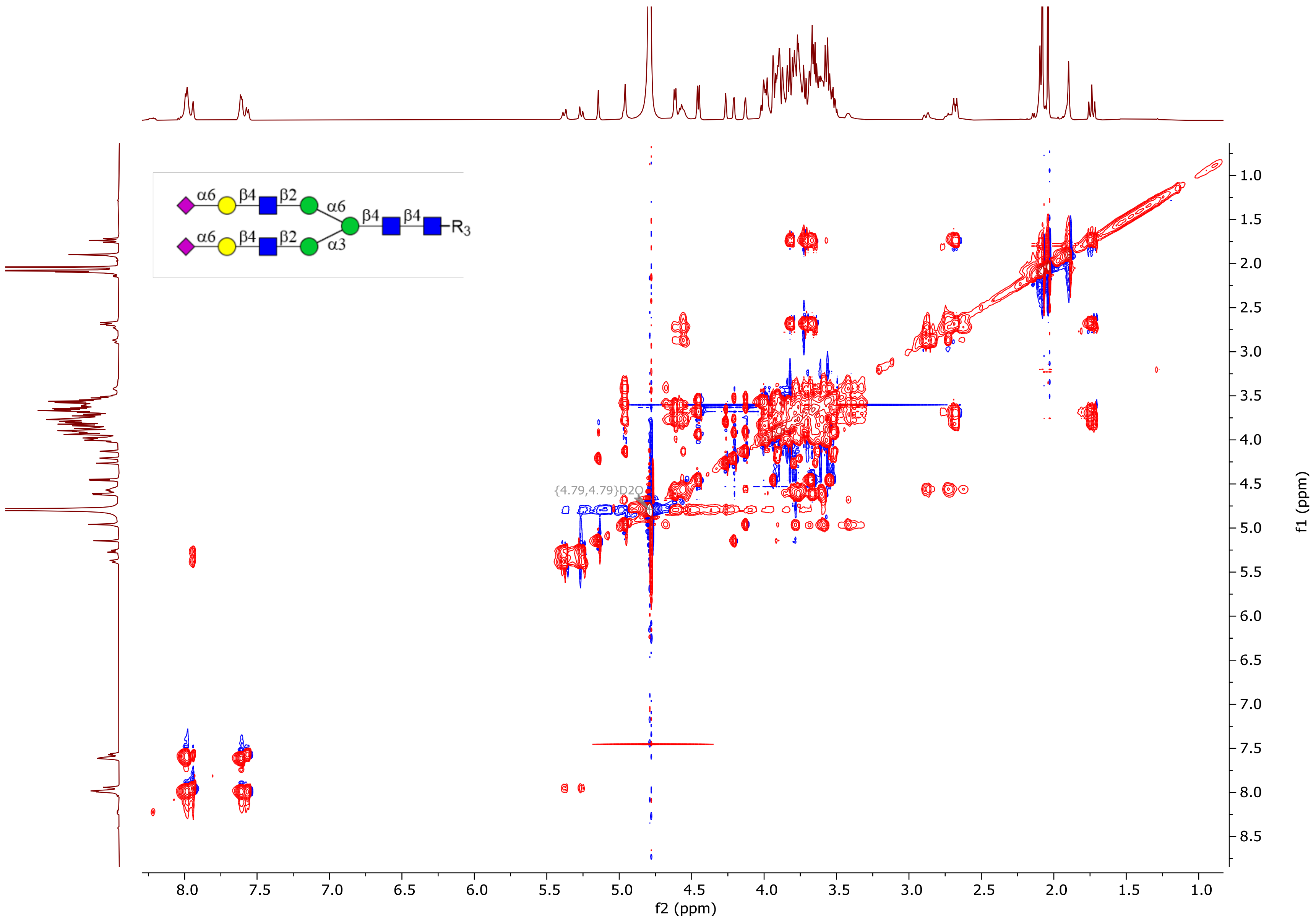

HMBC NMR of Compound **27**

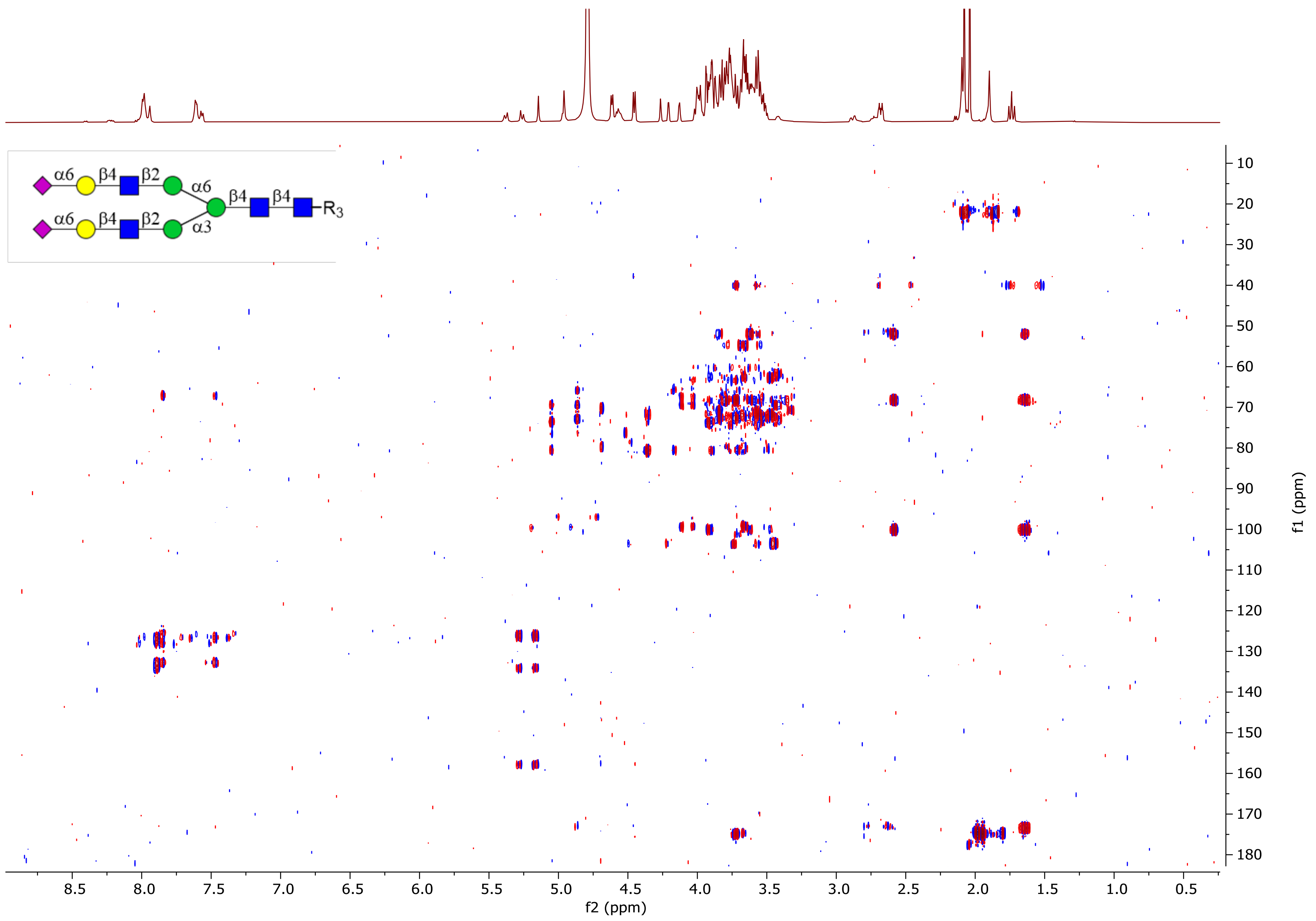

^{1}H NMR of Compound **28**

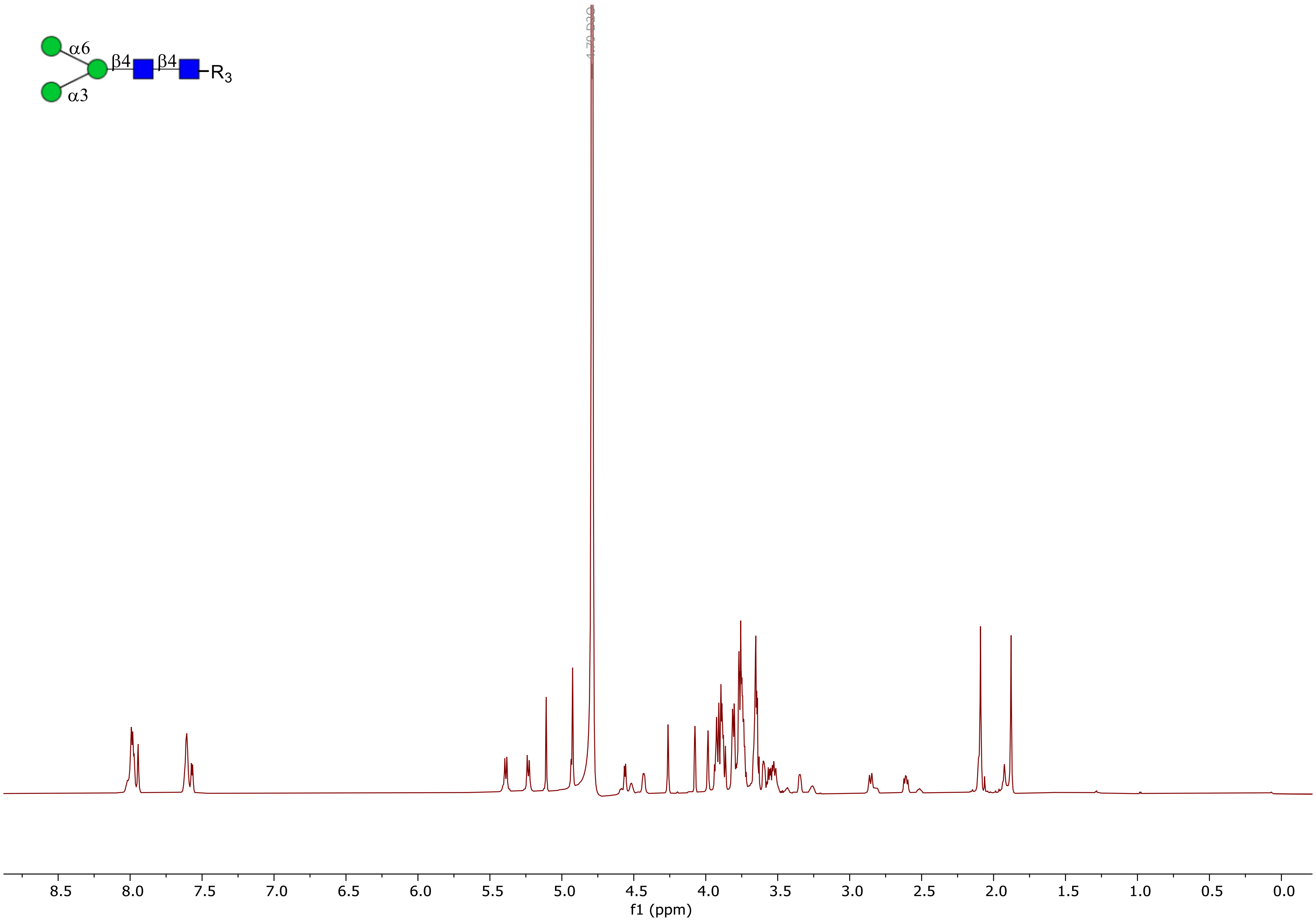

gCOSY NMR of Compound **28**

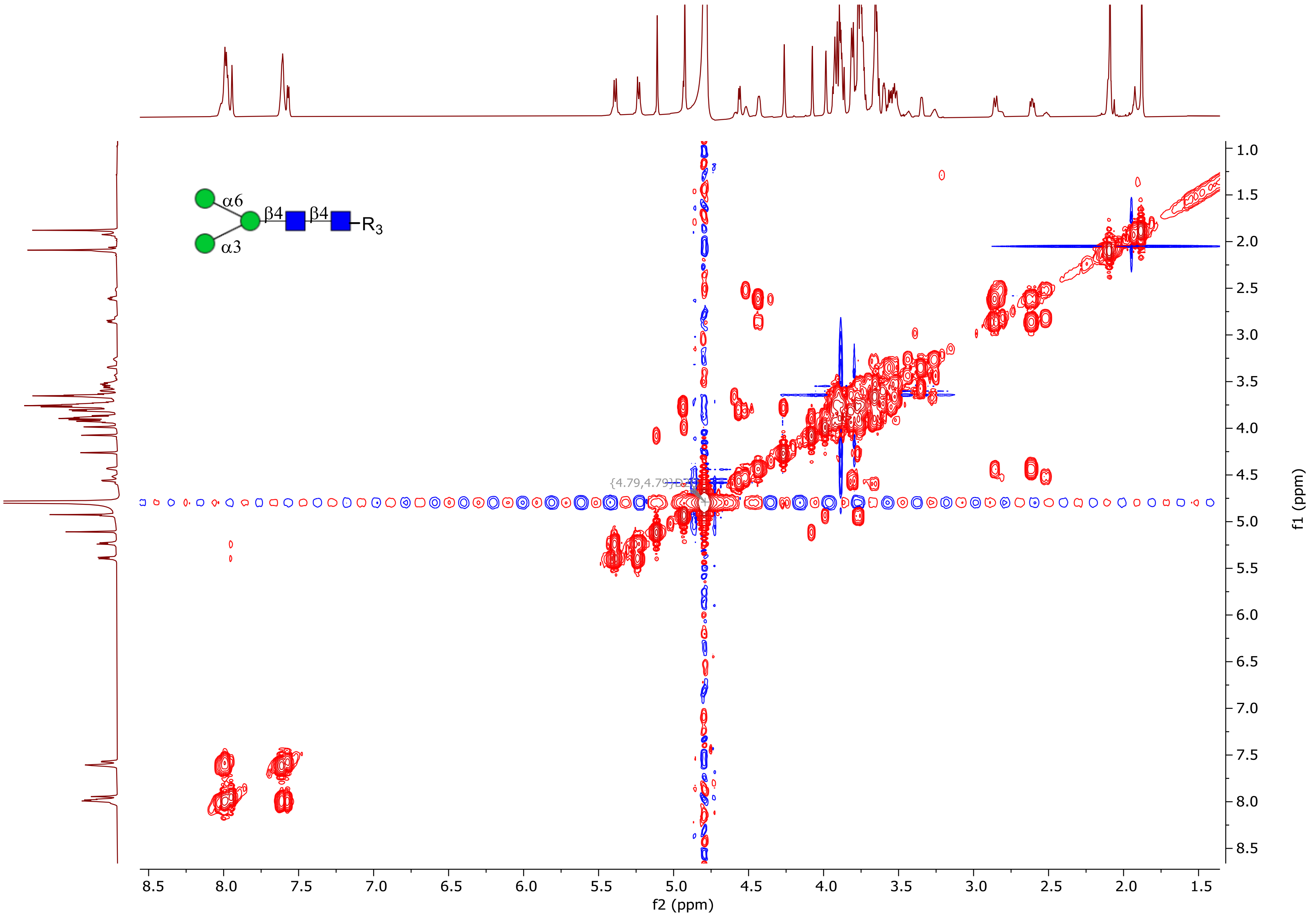

Multiplicity edited gHSQC NMR of Compound **28**

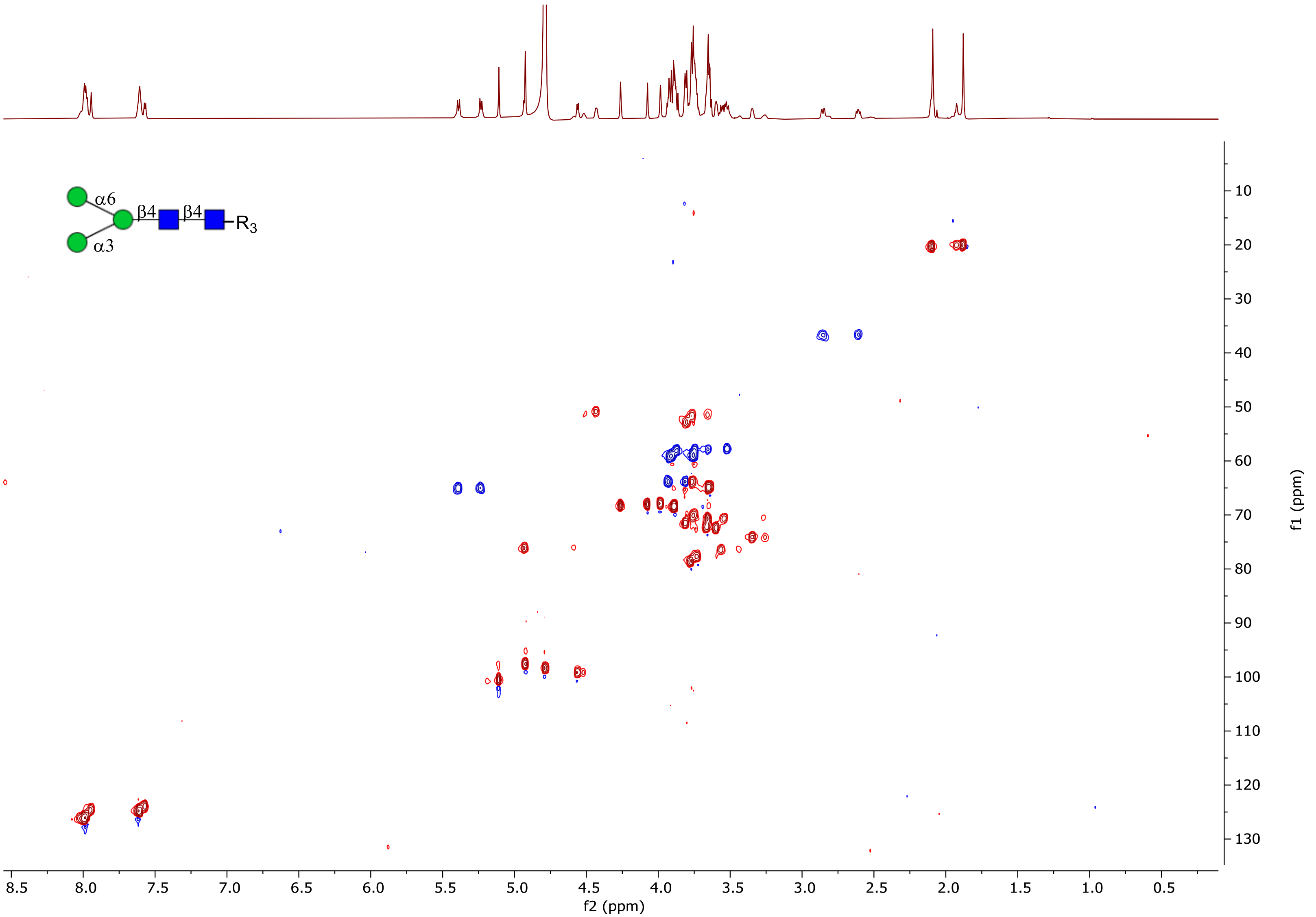

TOCSY-DIPSI NMR of Compound **28**

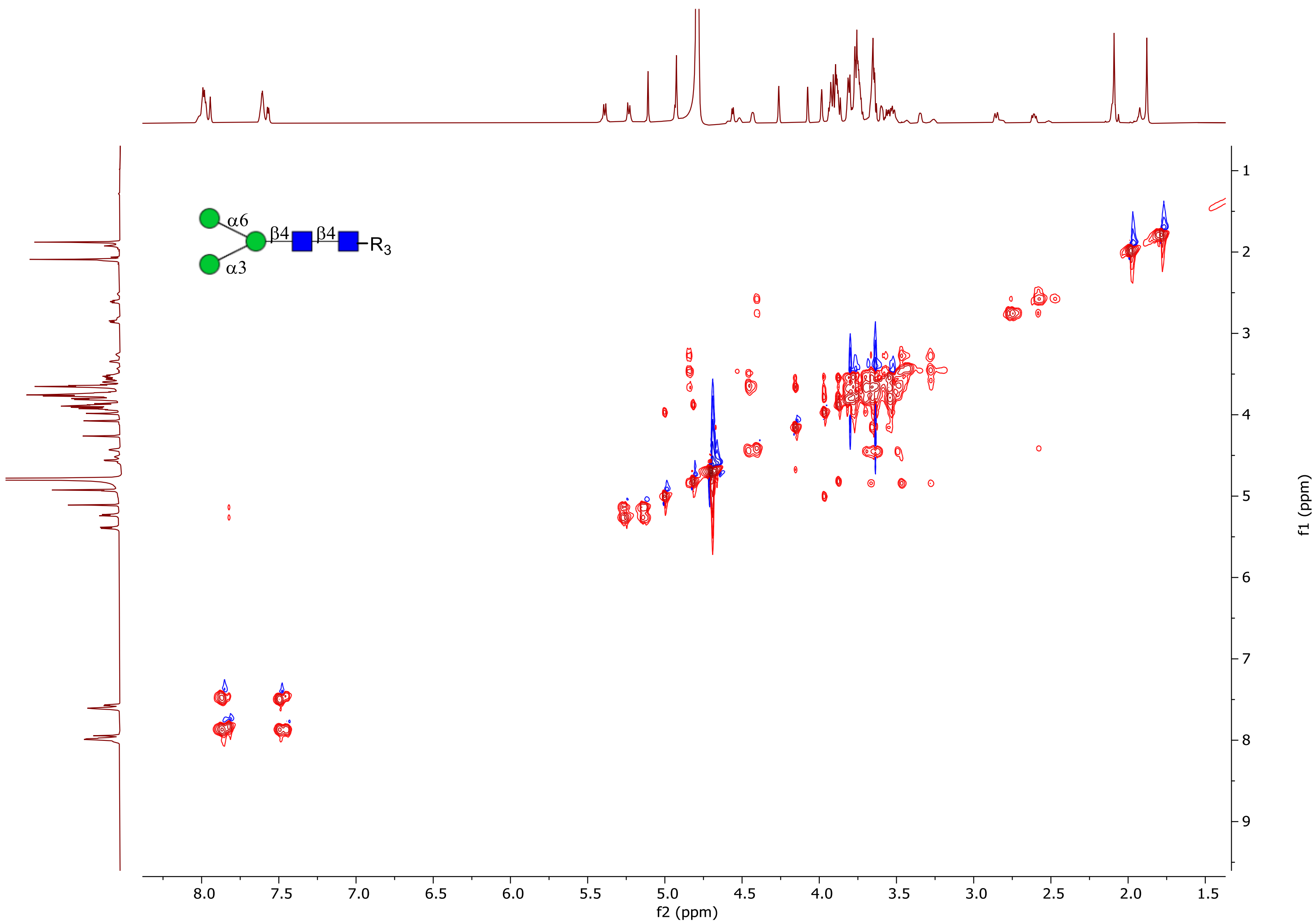

NOESY NMR of Compound **28**

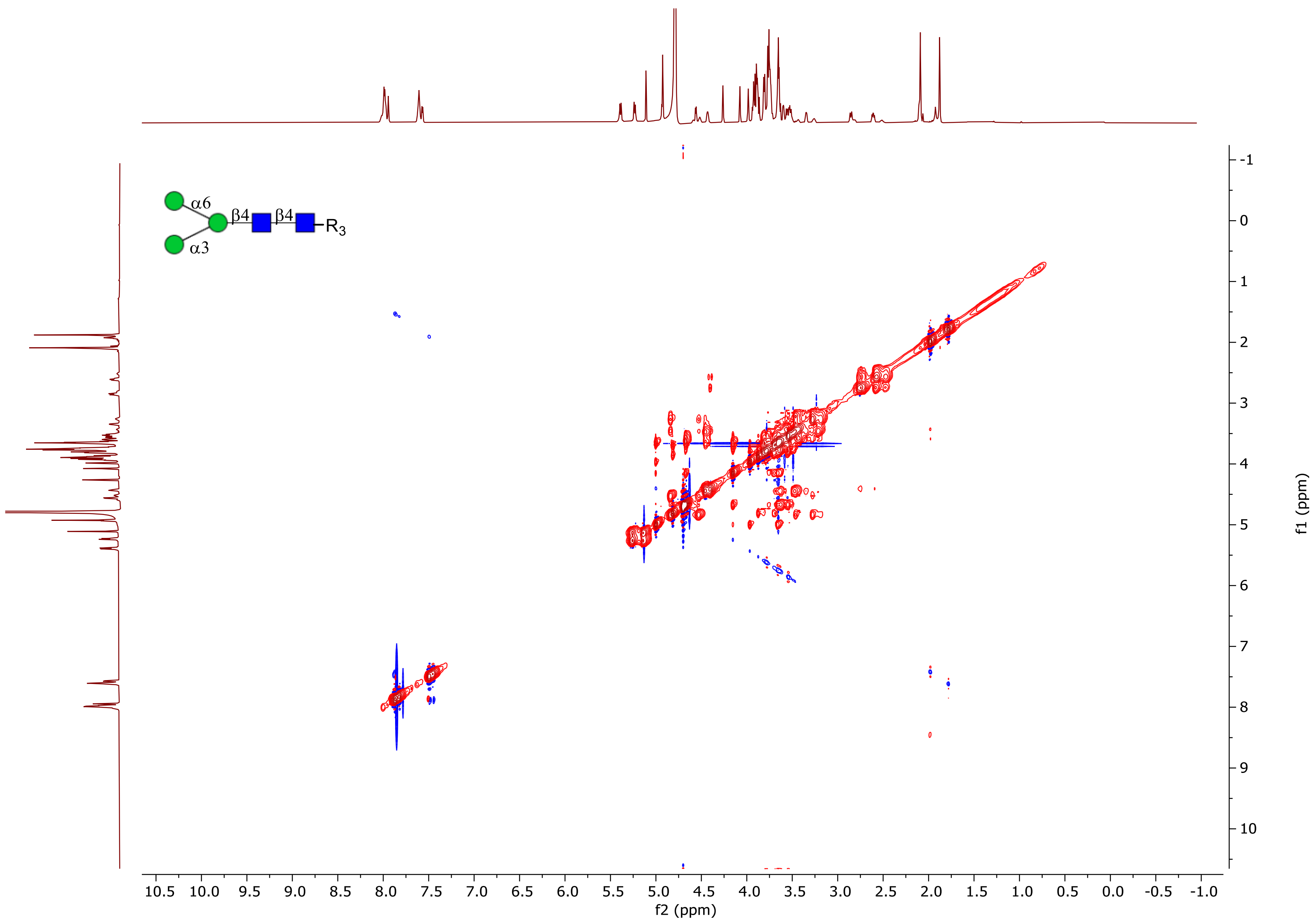

^{1}H NMR of Compound **34**

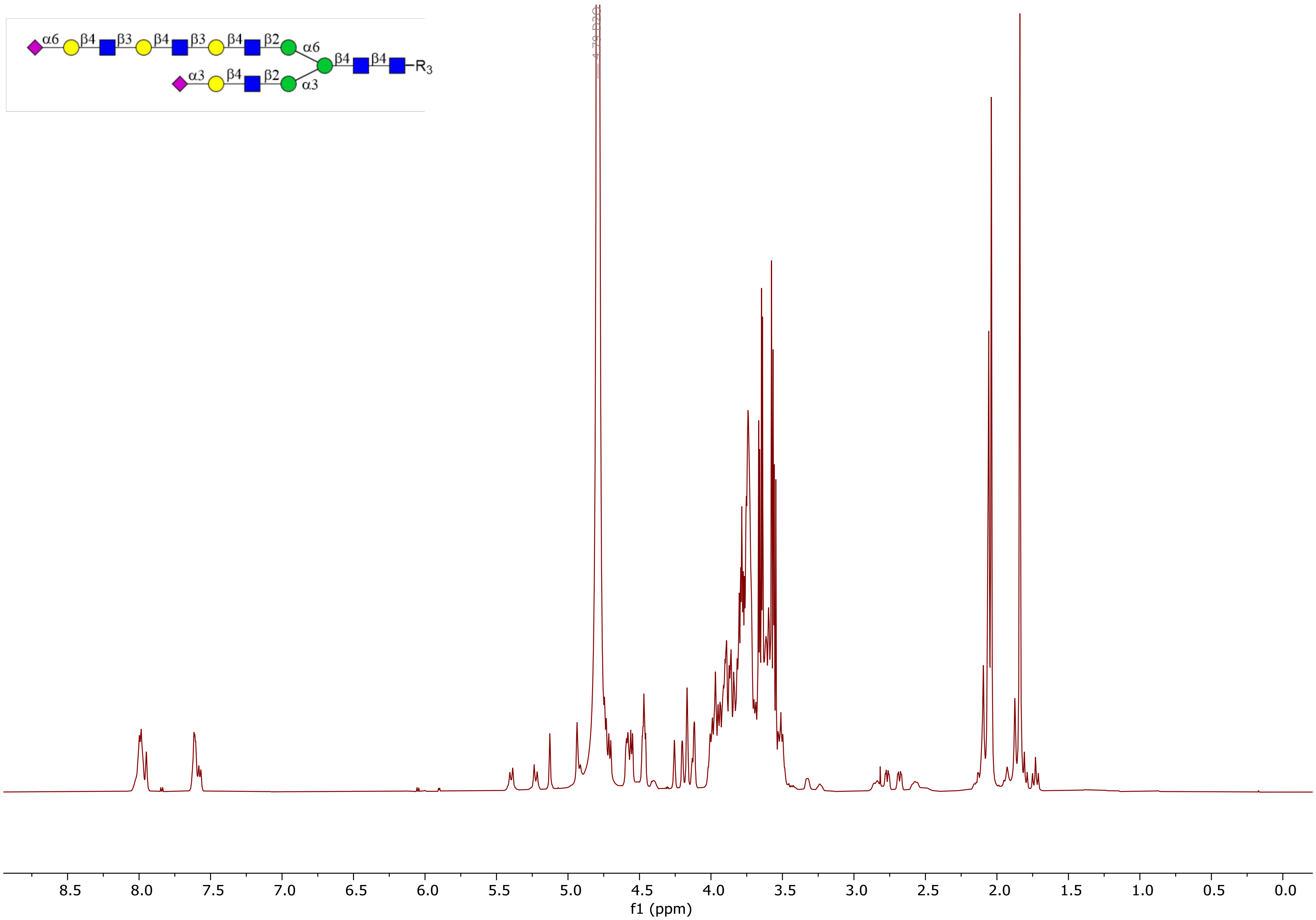

gCOSY NMR of Compound **34**

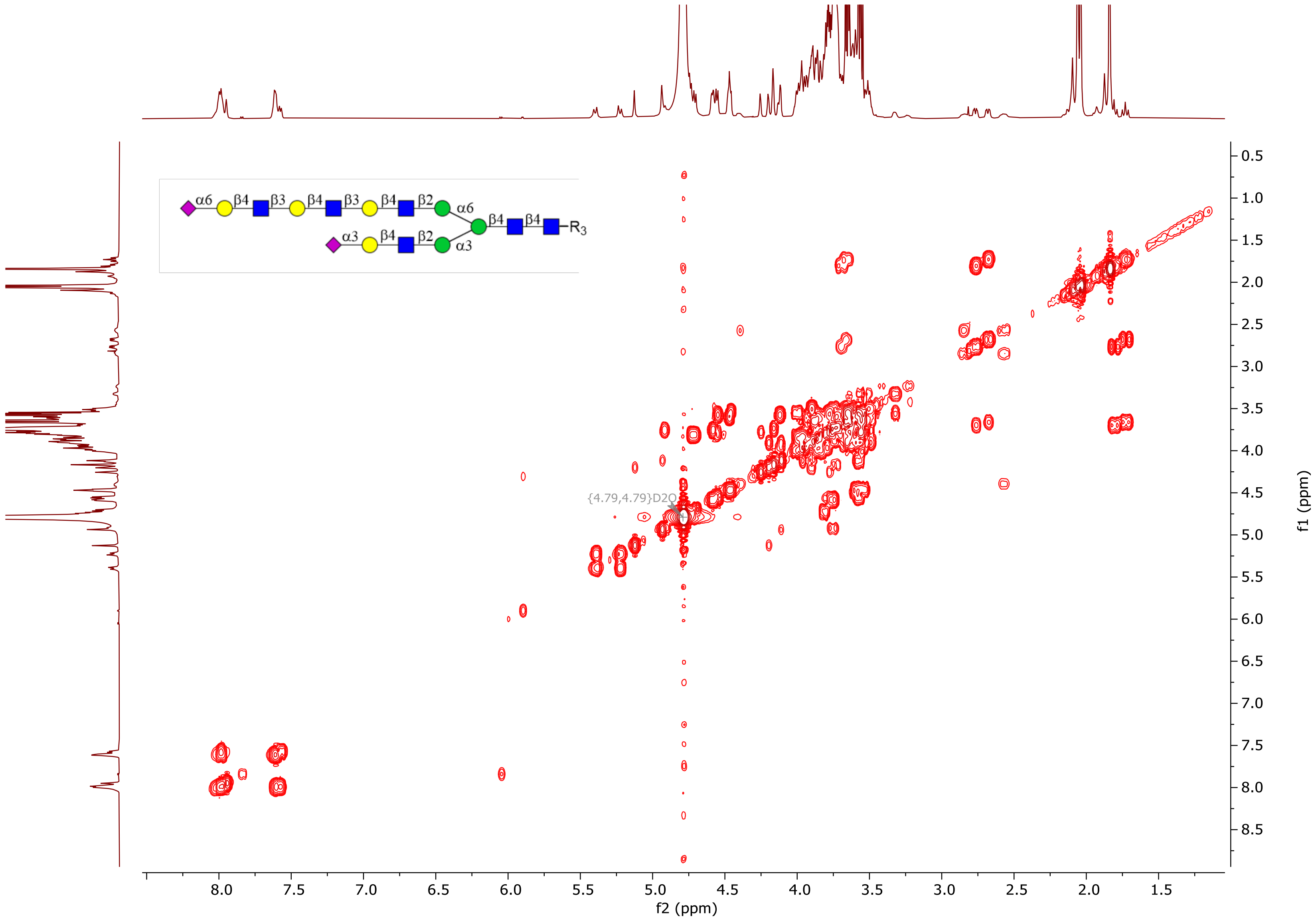

Multiplicity edited gHSQC NMR of Compound **34**

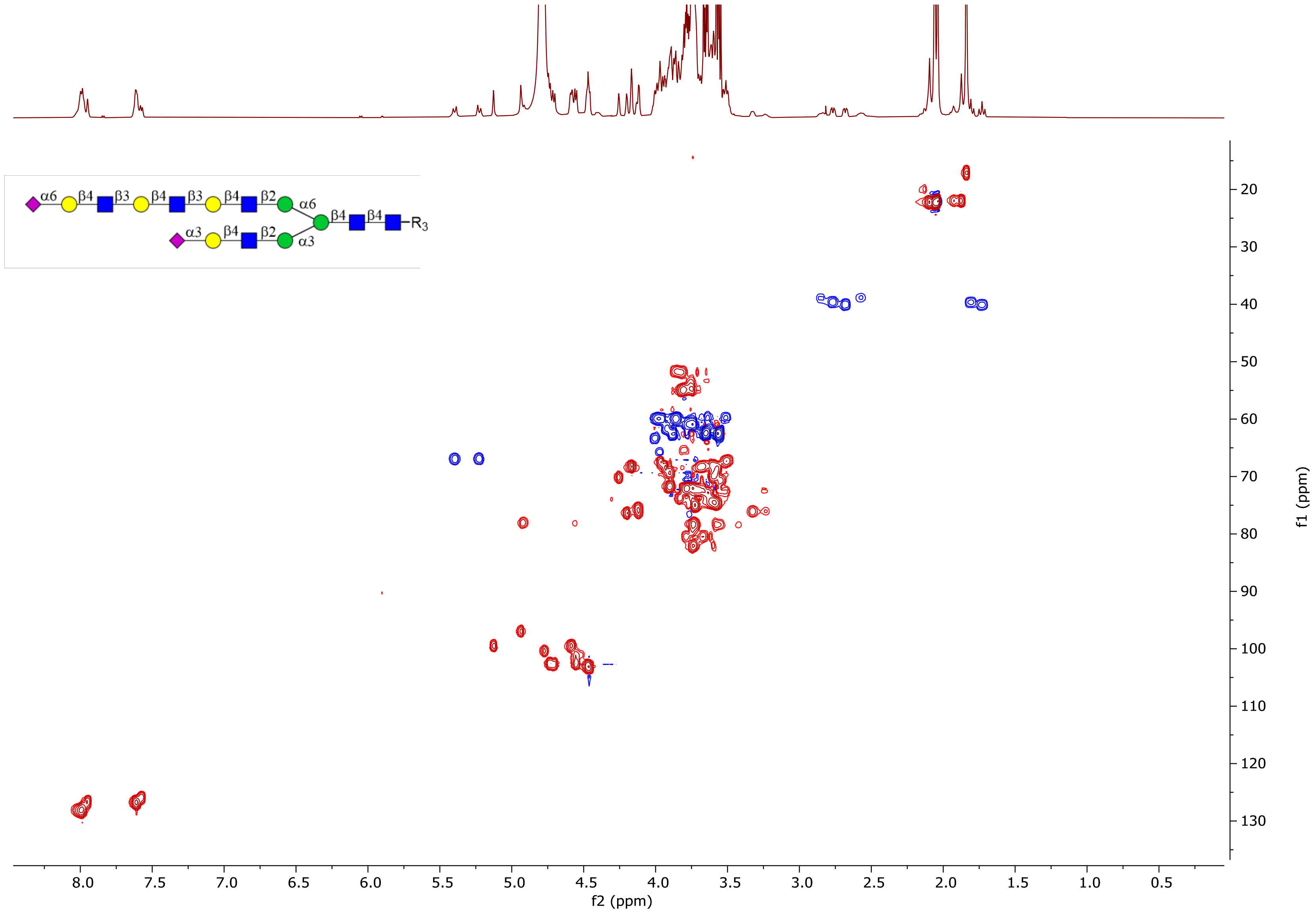

TOCSY-DIPSI NMR of Compound **34**

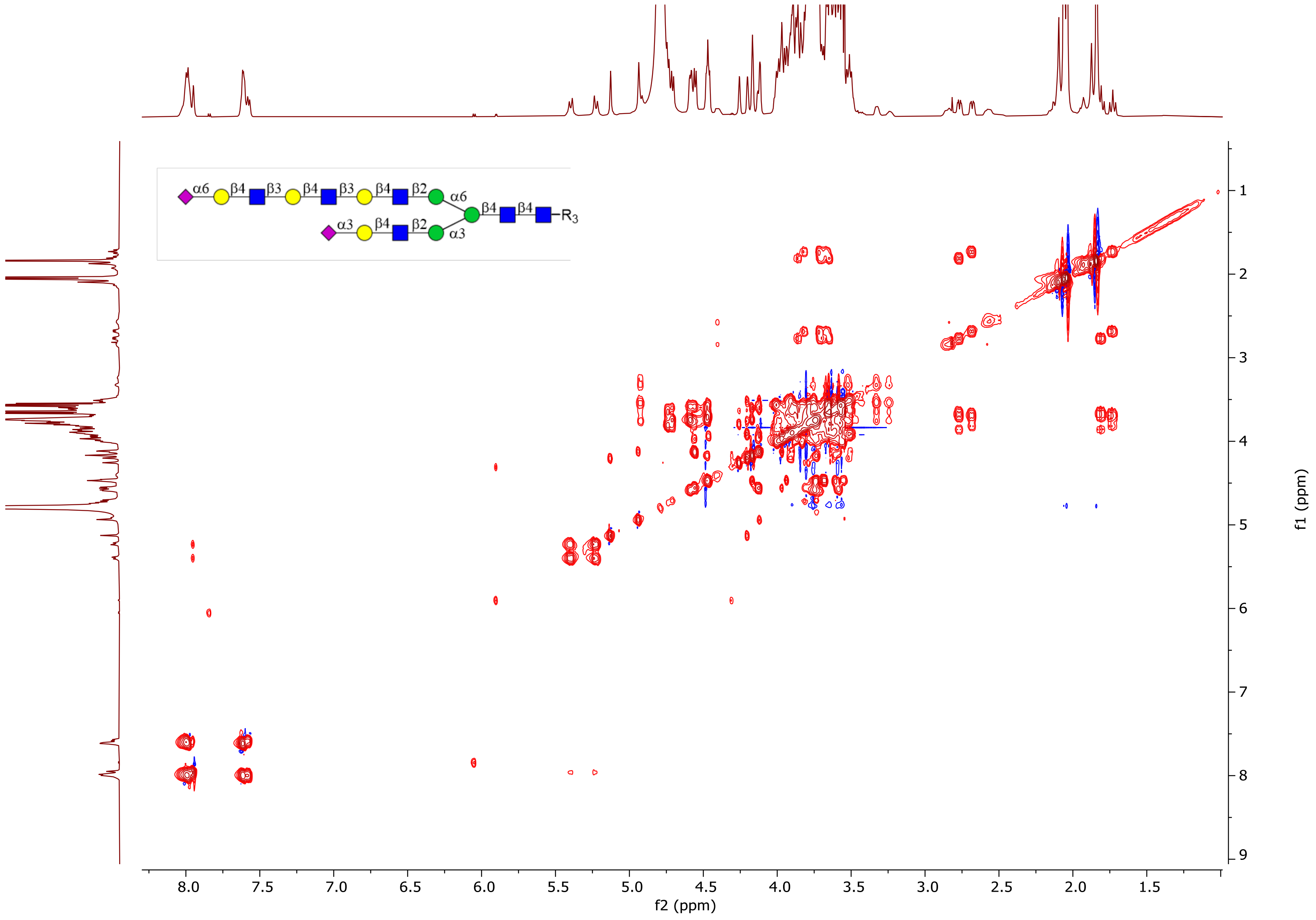

NOESY NMR of Compound **34**

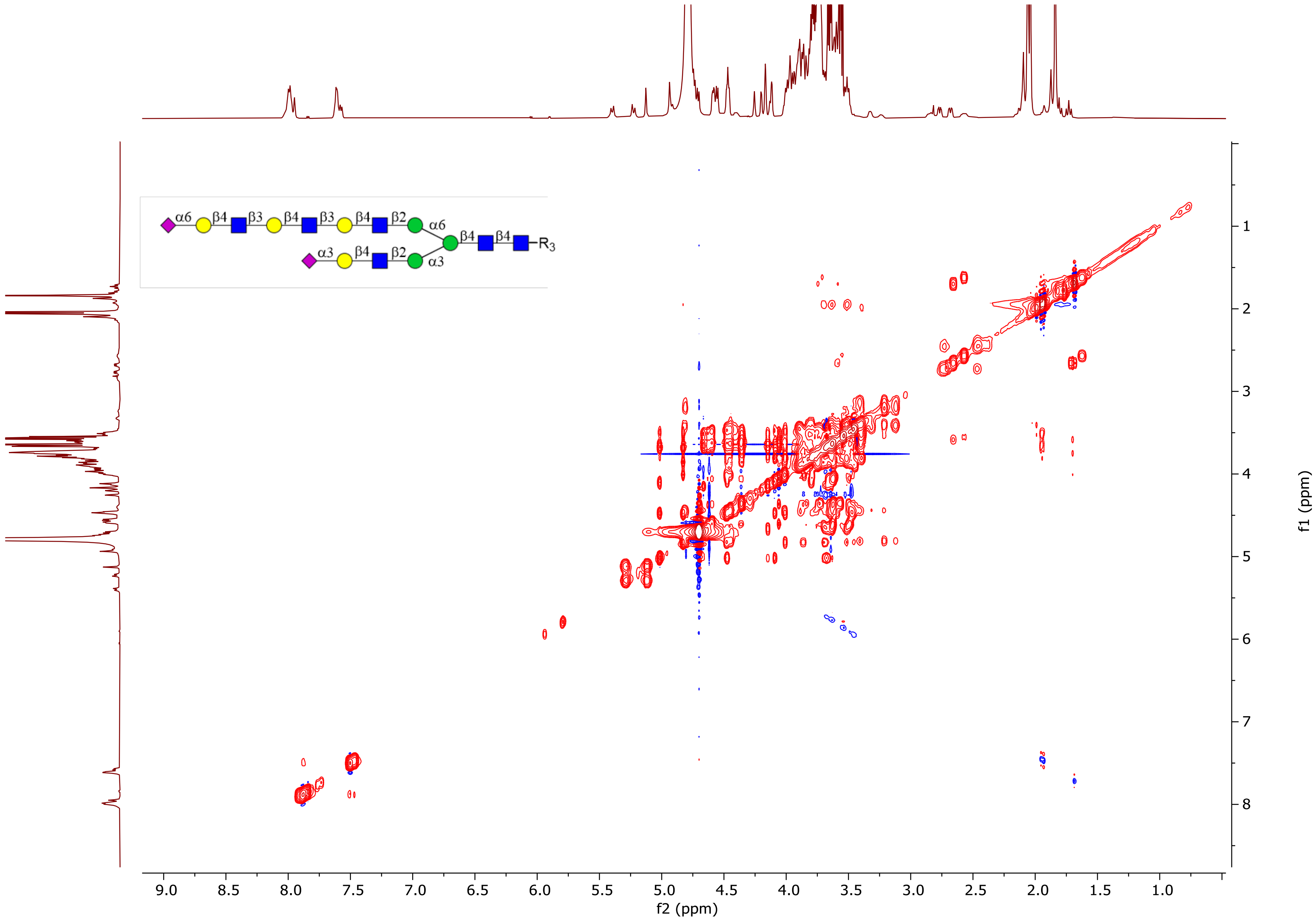

HMBC NMR of Compound **34**

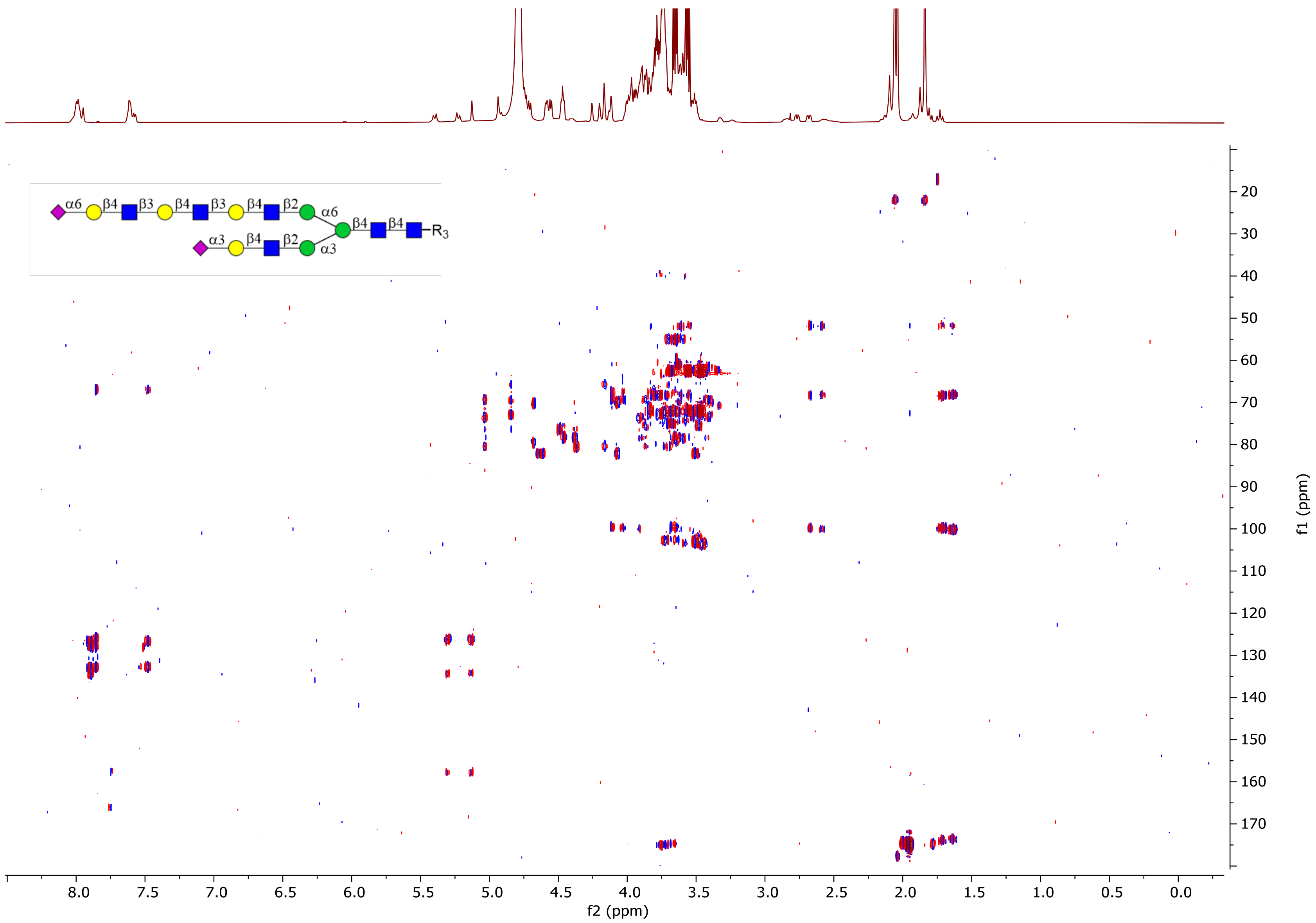

^{1}H NMR of Compound **44**

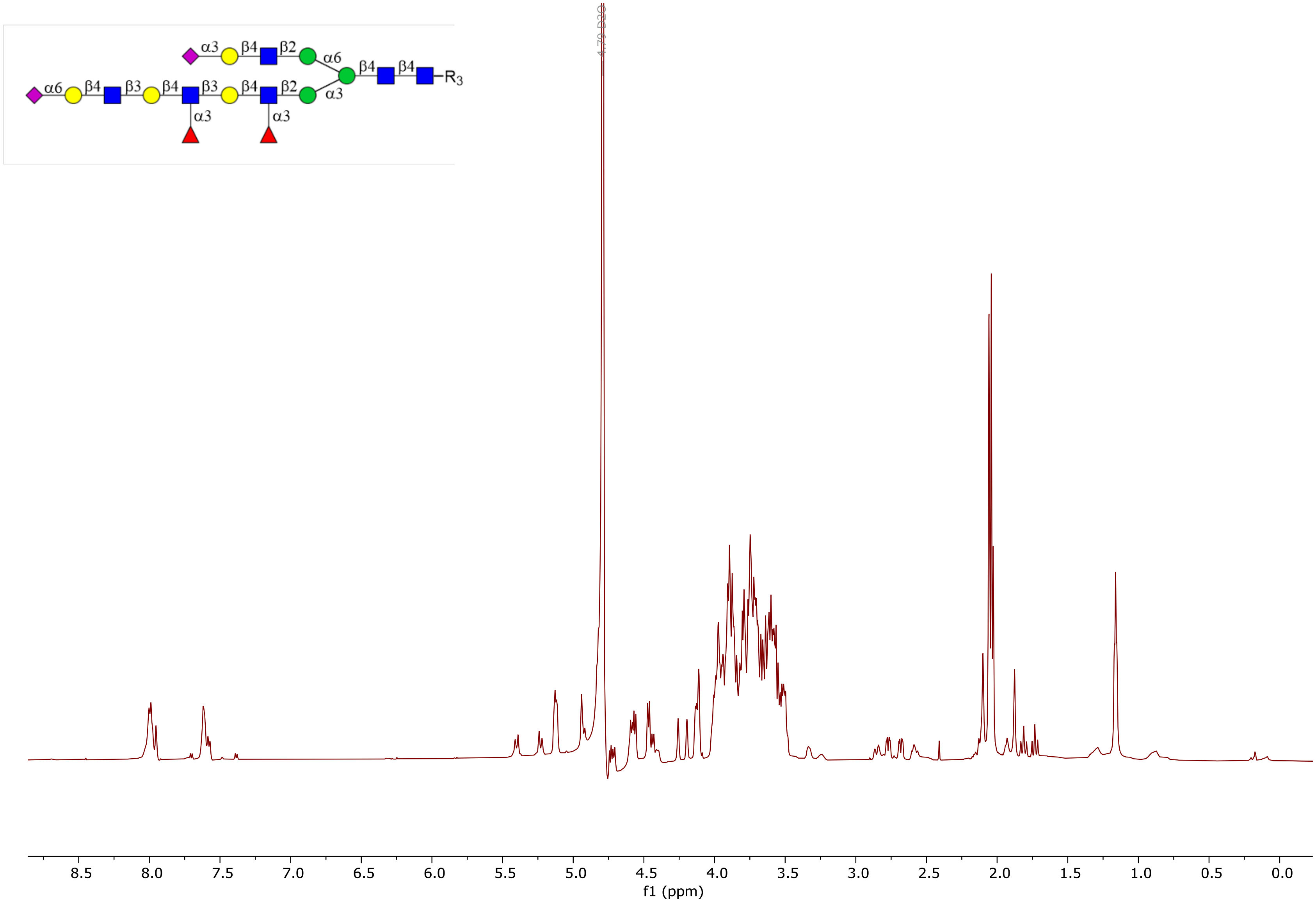

^{1}H (Presat.) NMR of Compound **44**

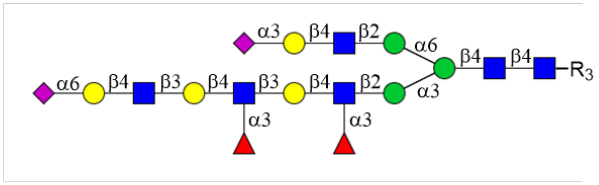

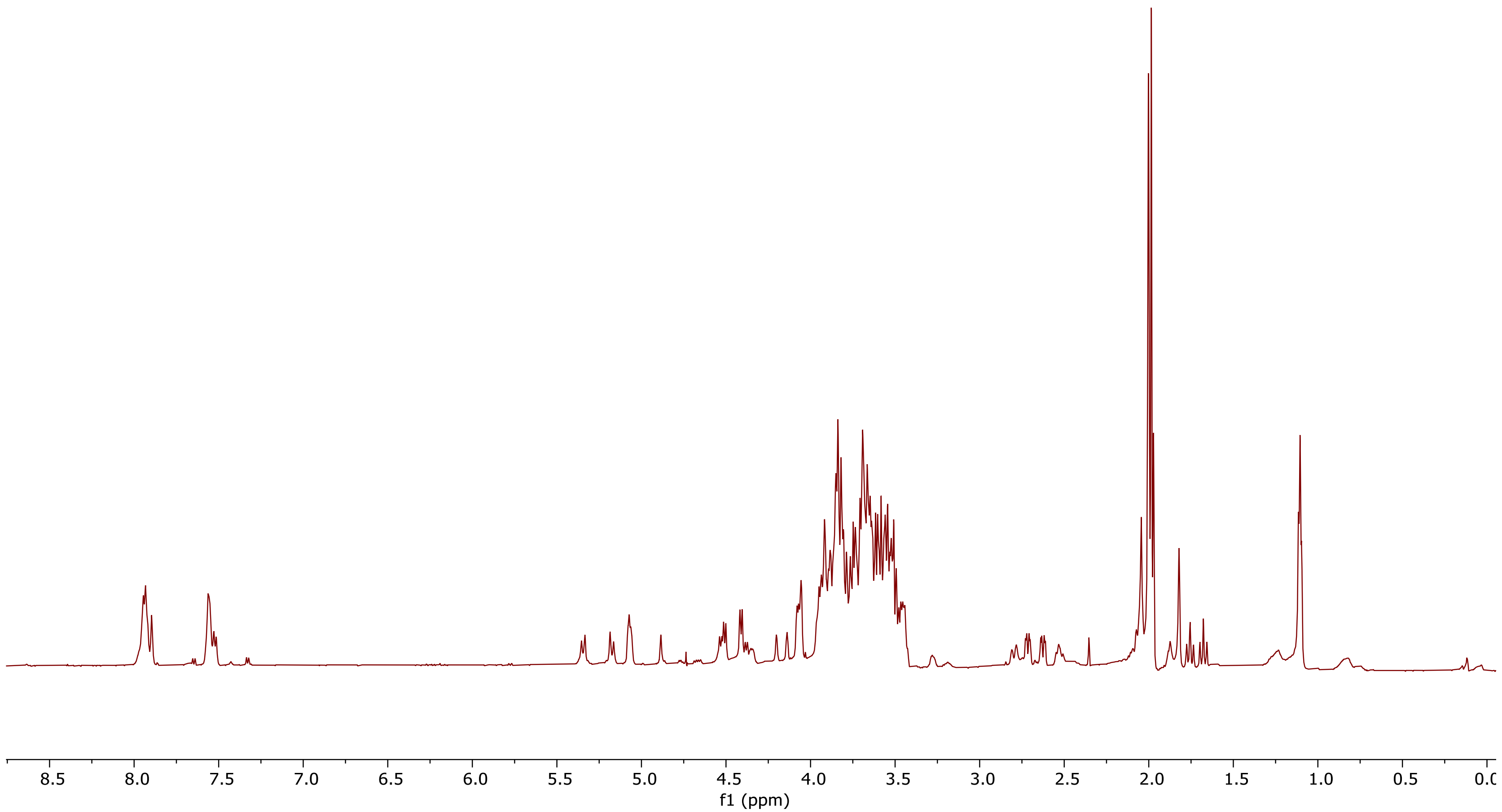

gCOSY NMR of Compound **44**

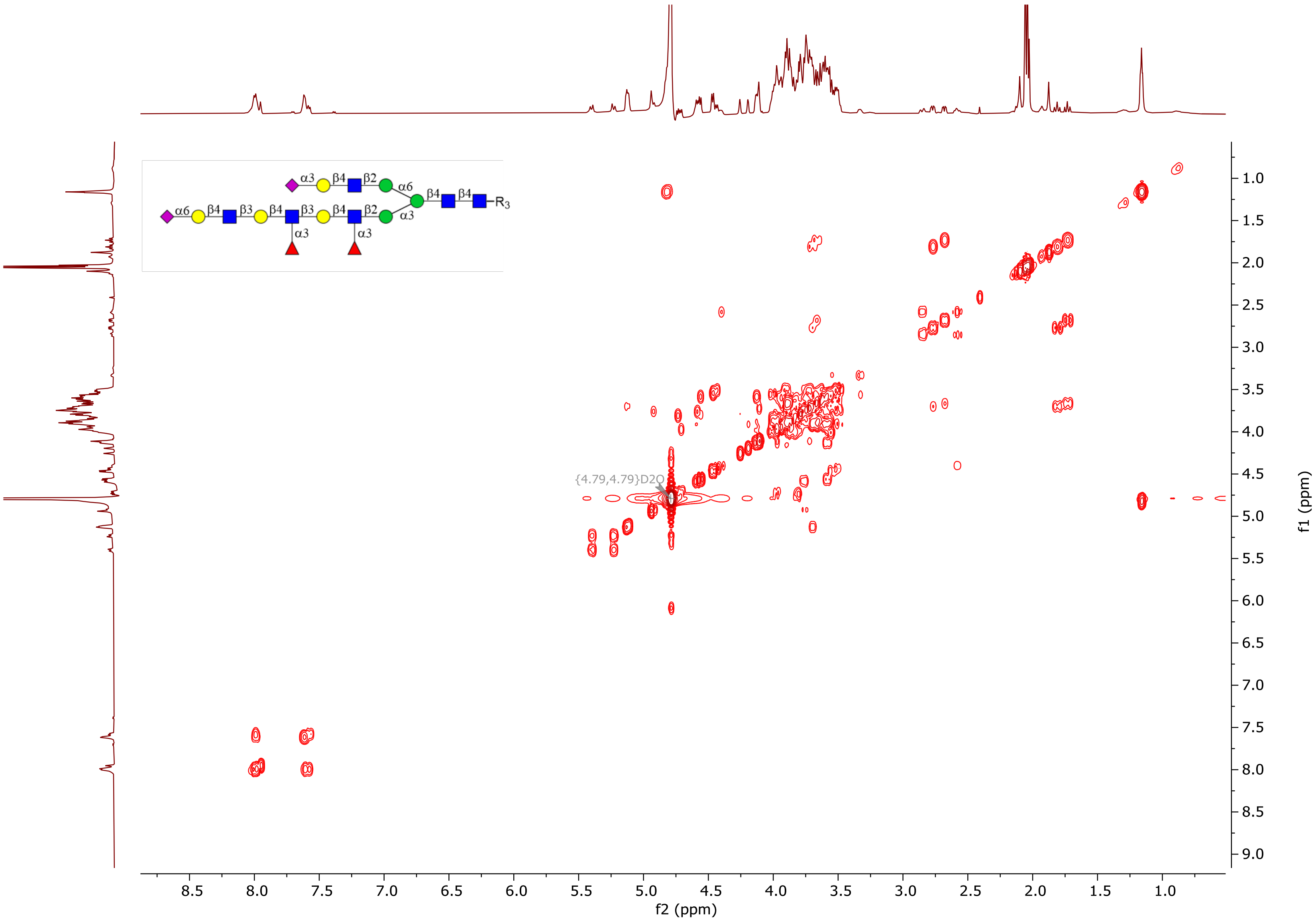

Multiplicity edited gHSQC NMR of Compound **44**

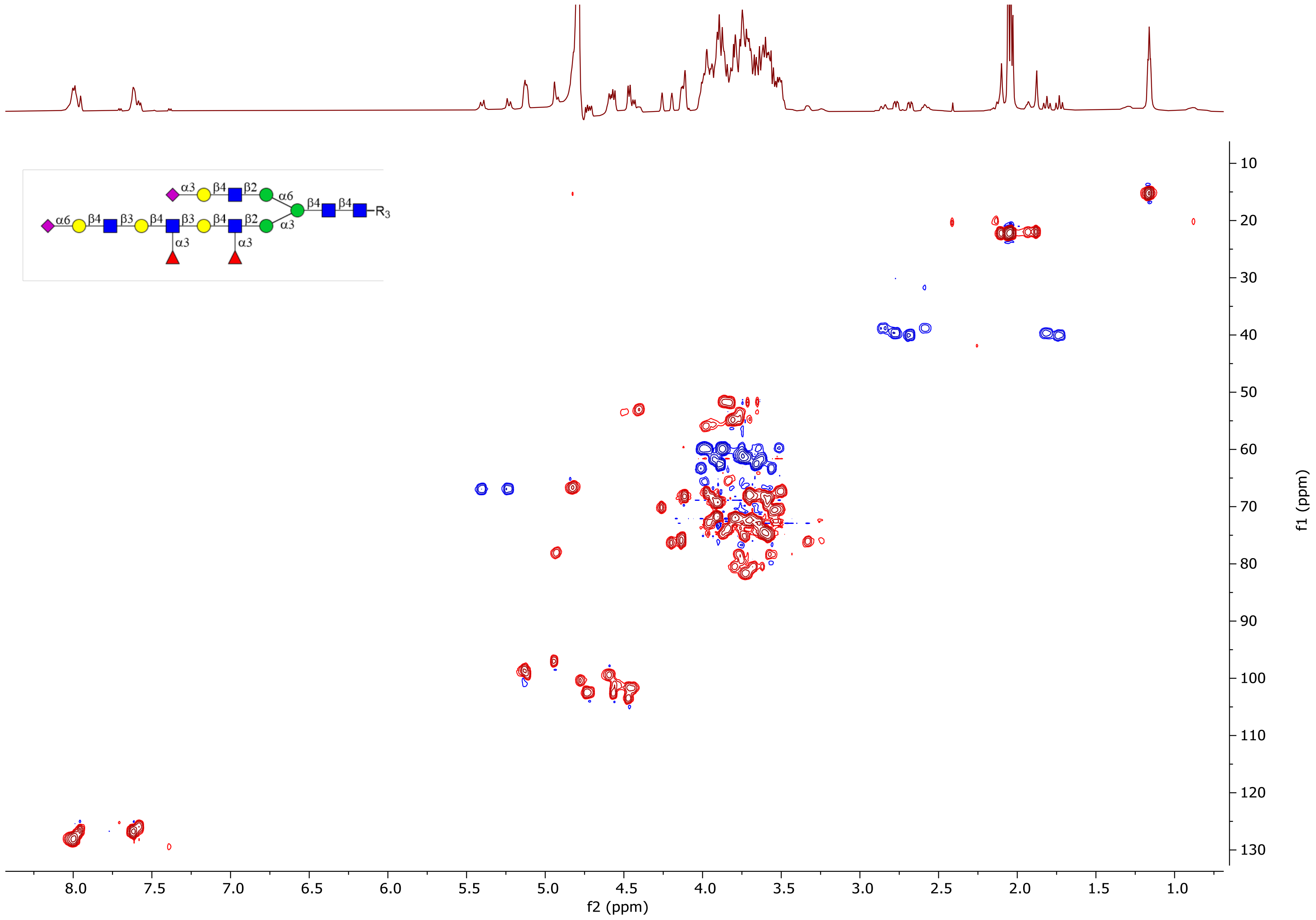

TOCSY-DIPSI NMR of Compound **44**

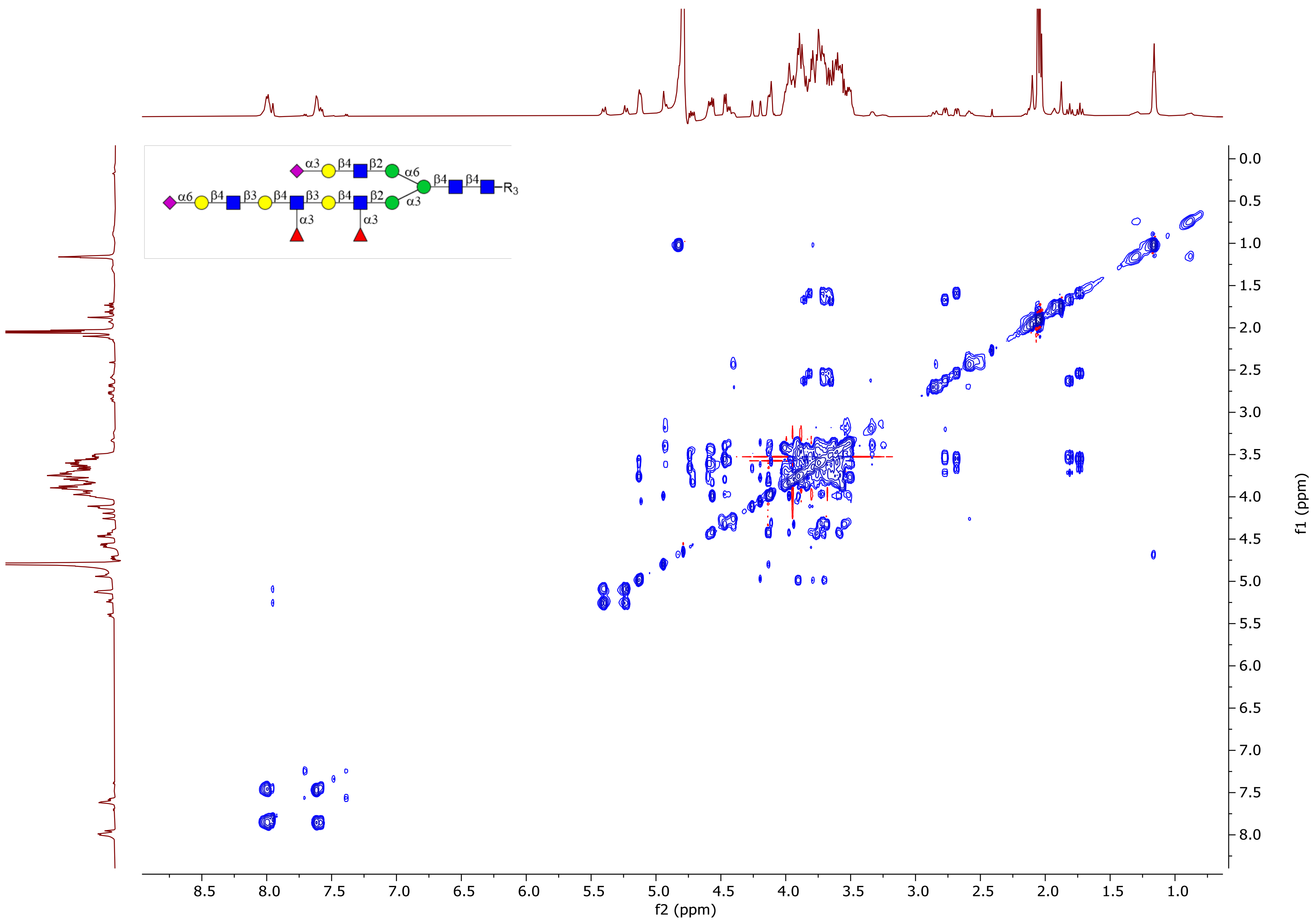

NOESY NMR of Compound **44**

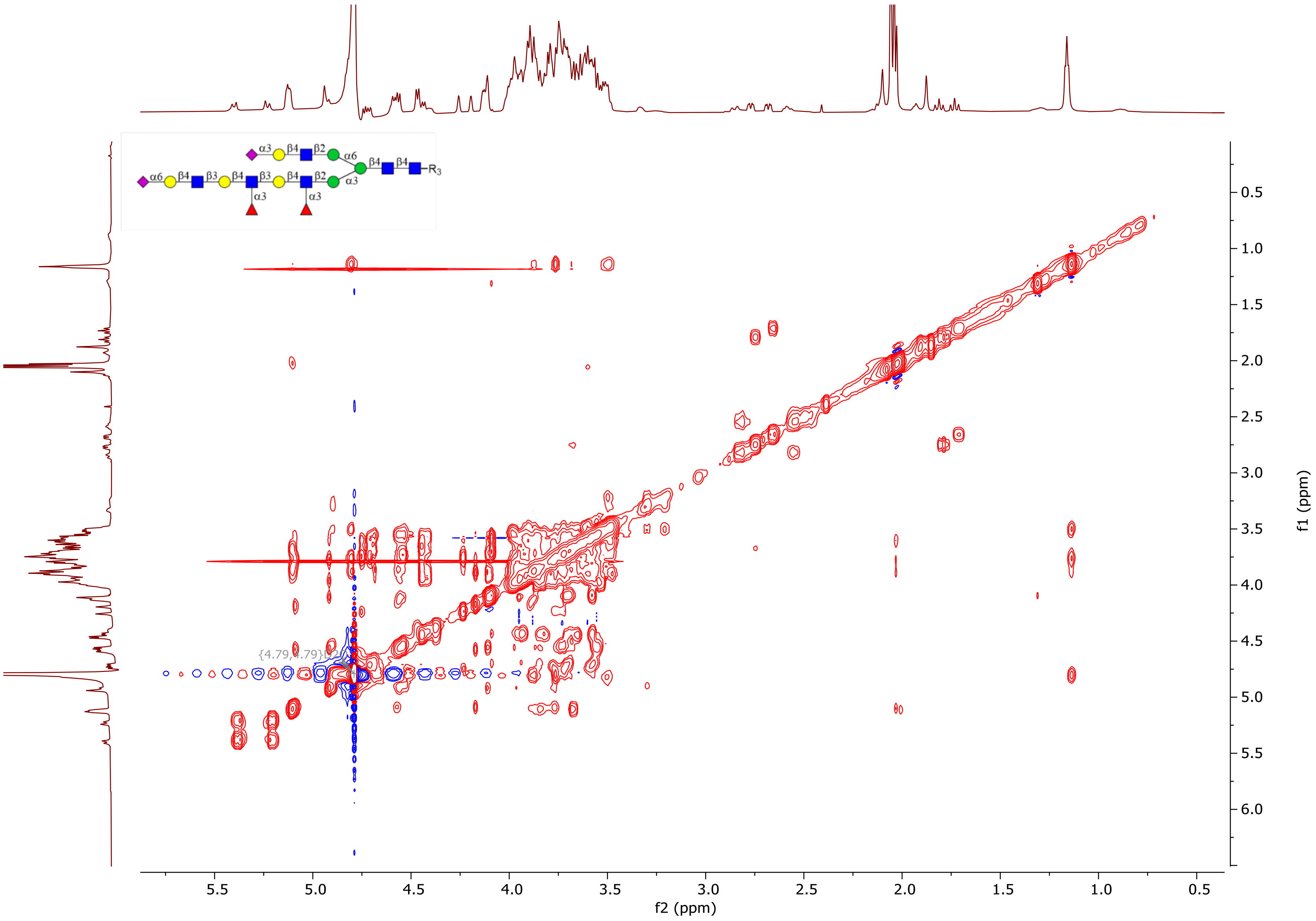

^{1}H NMR of Compound **52**

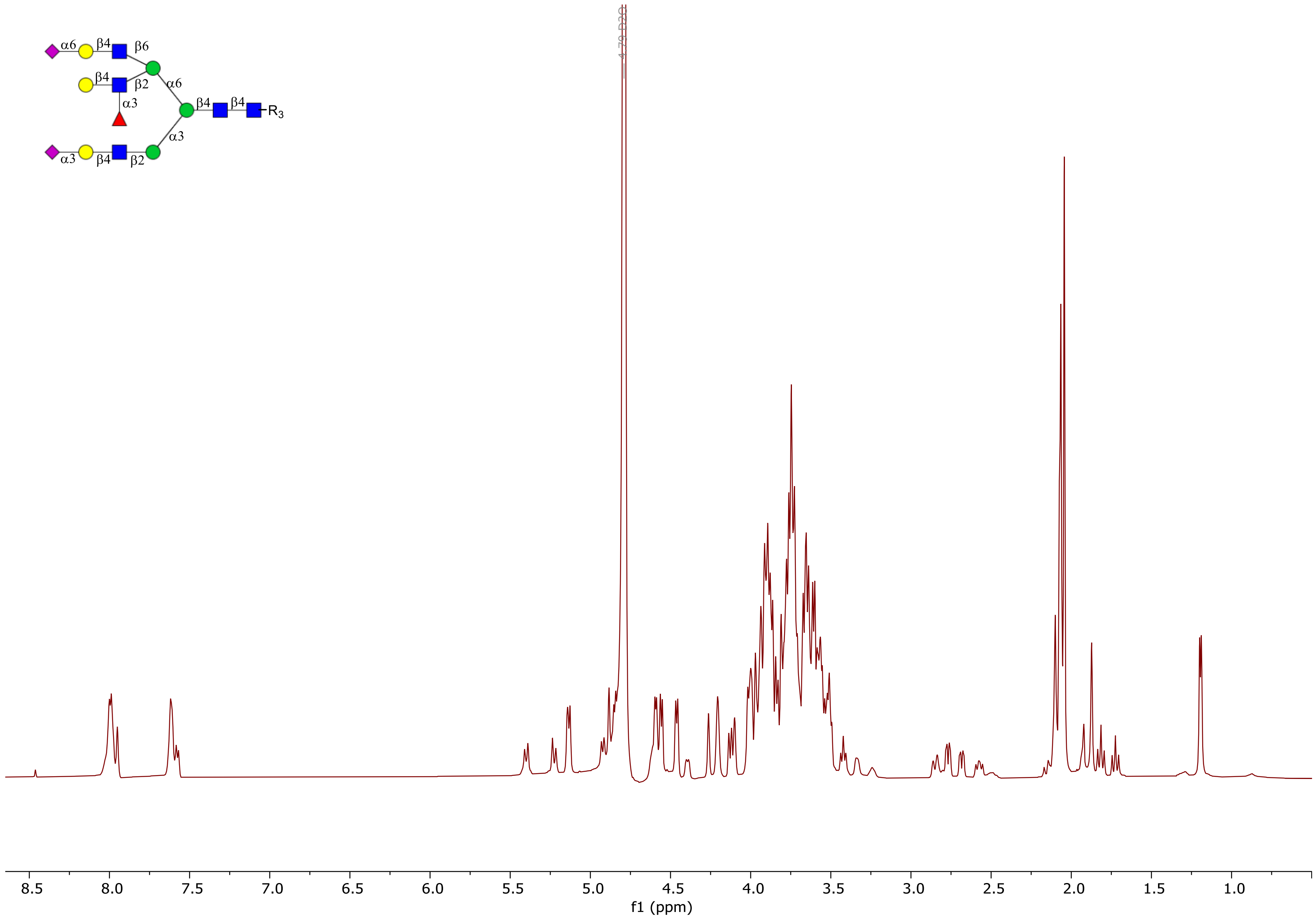

^{1}H NMR (Presat.) of Compound **52**

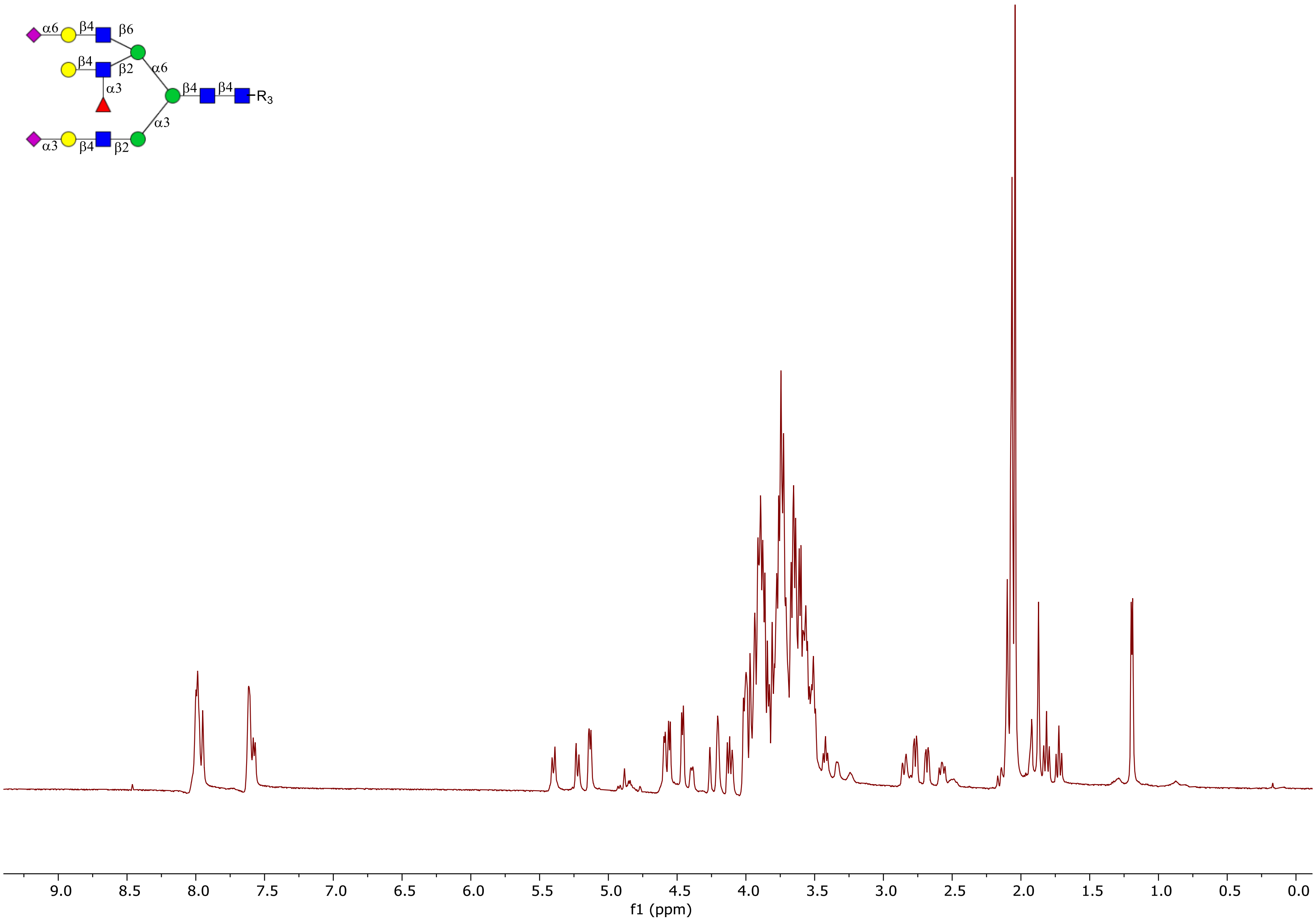

gCOSY NMR of Compound **52**

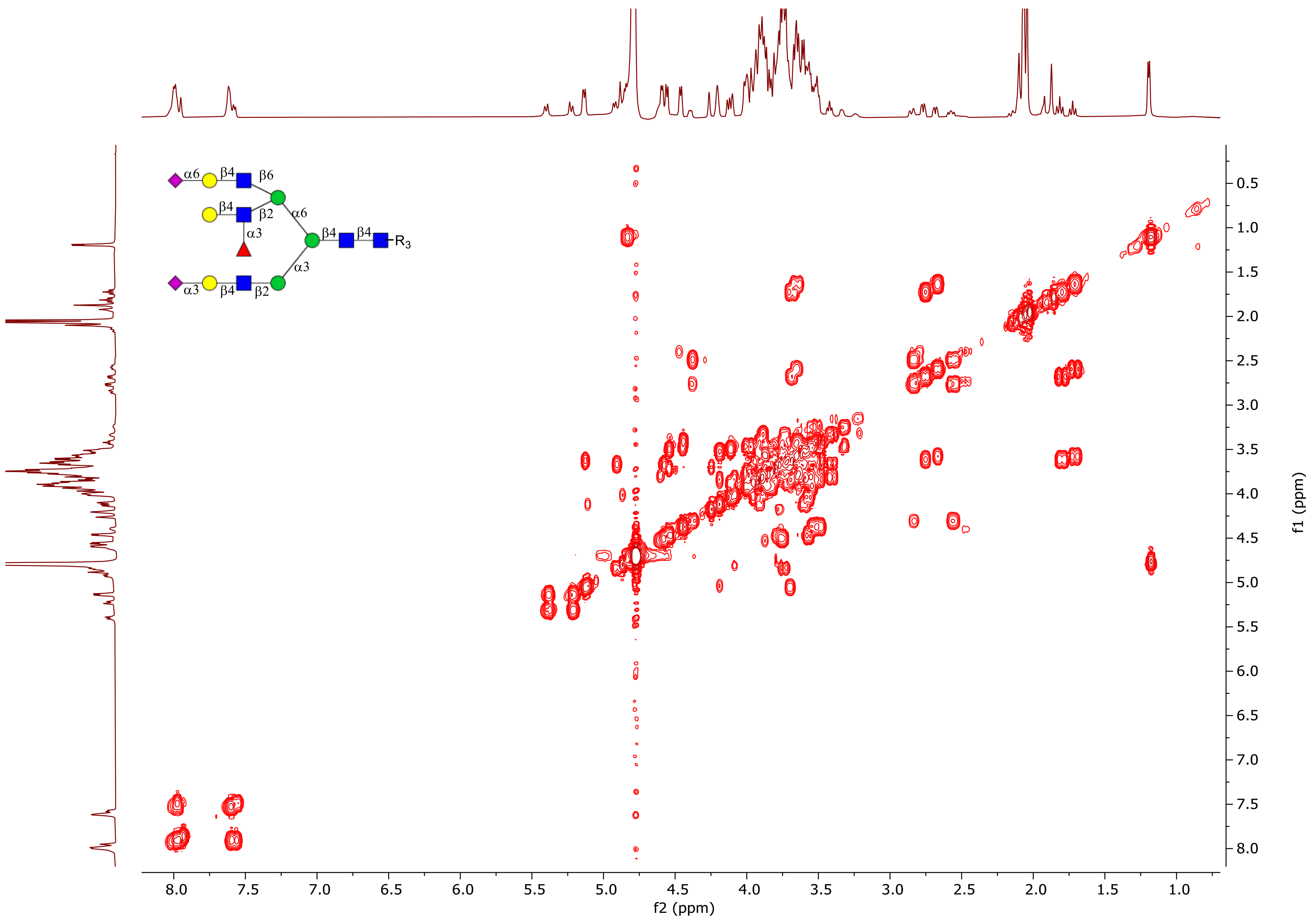

Multiplicity edited gHSQC NMR of Compound **52**

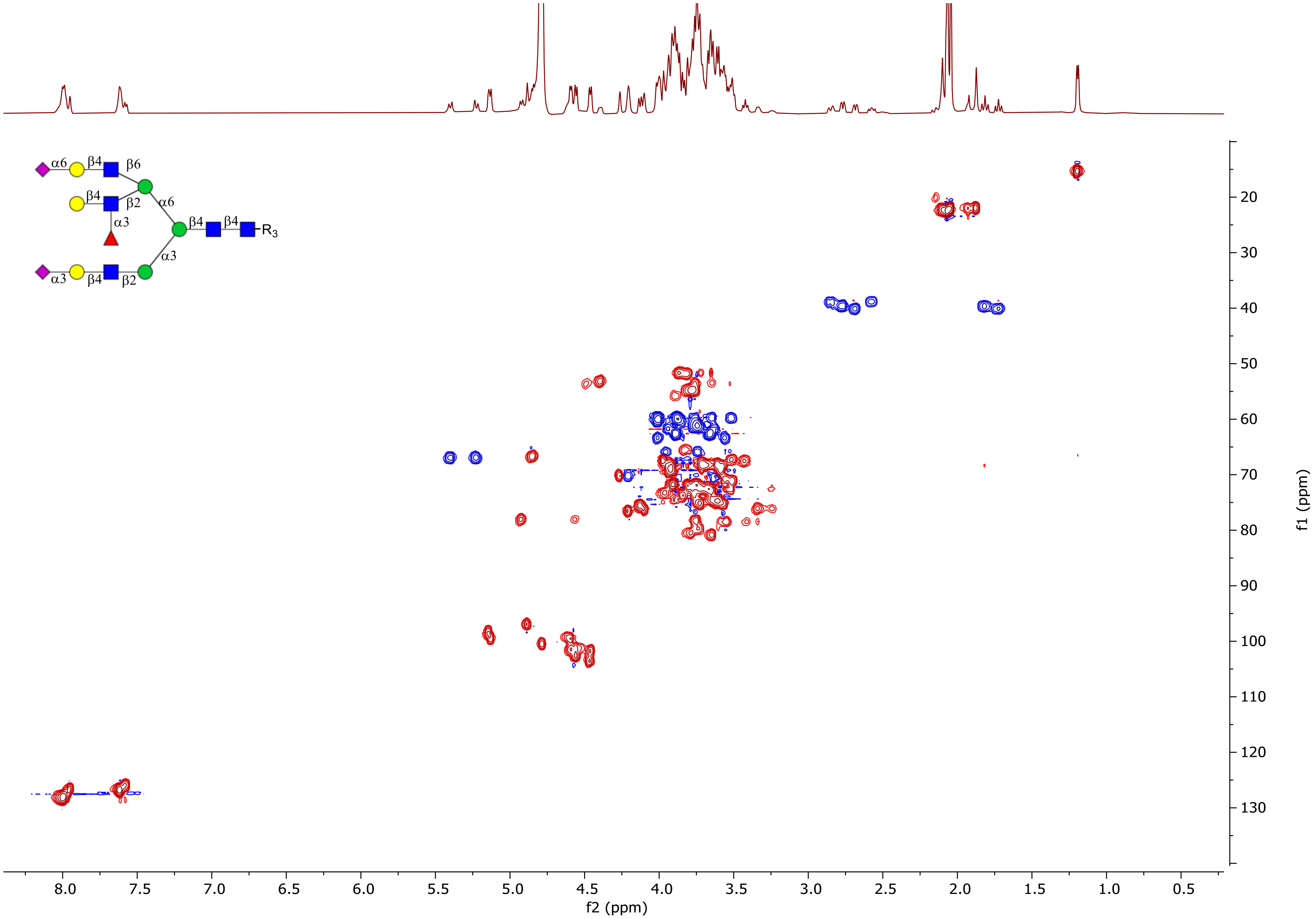

TOCSY-DIPSI NMR of Compound **52**

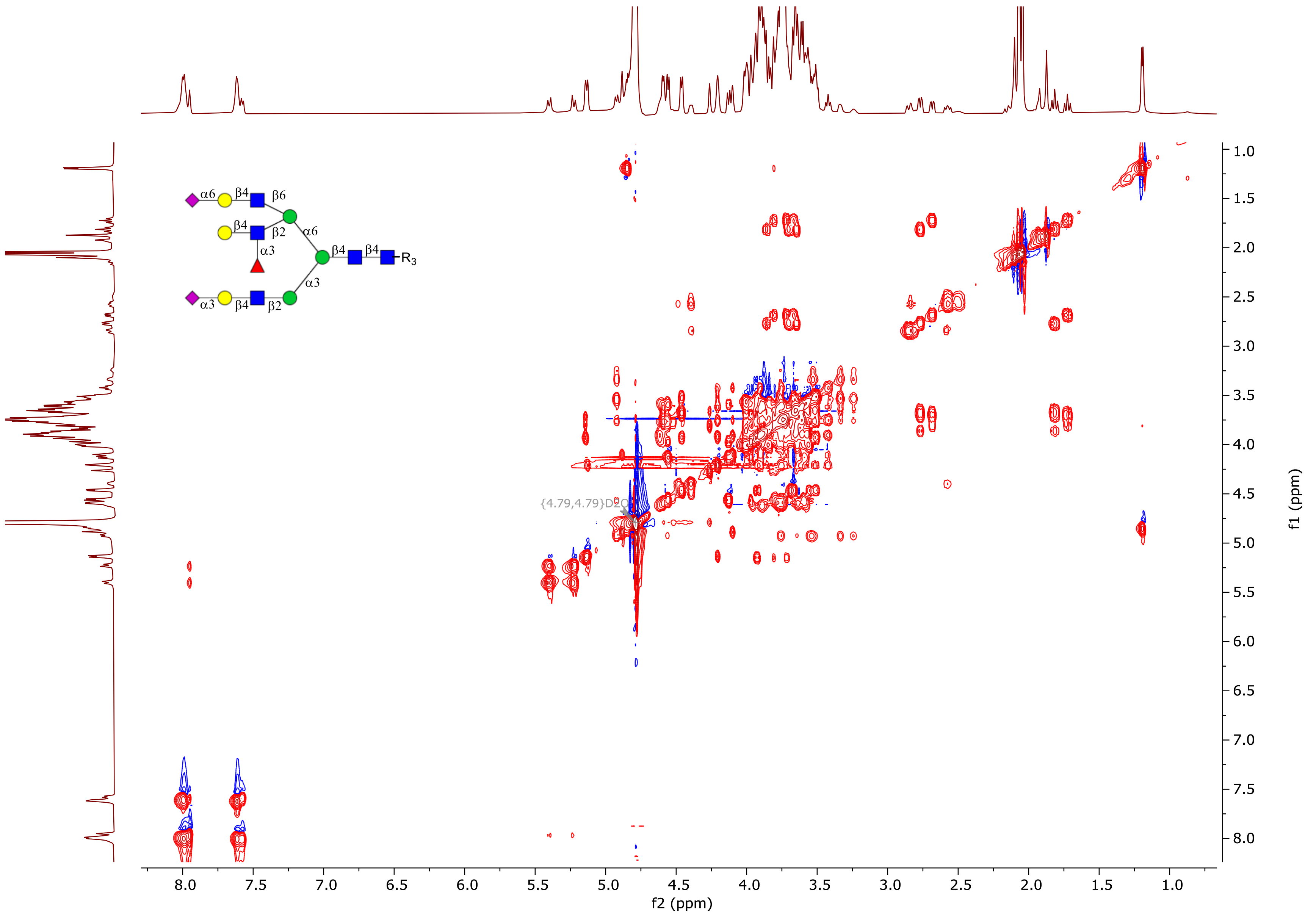

NOESY NMR of Compound **52**

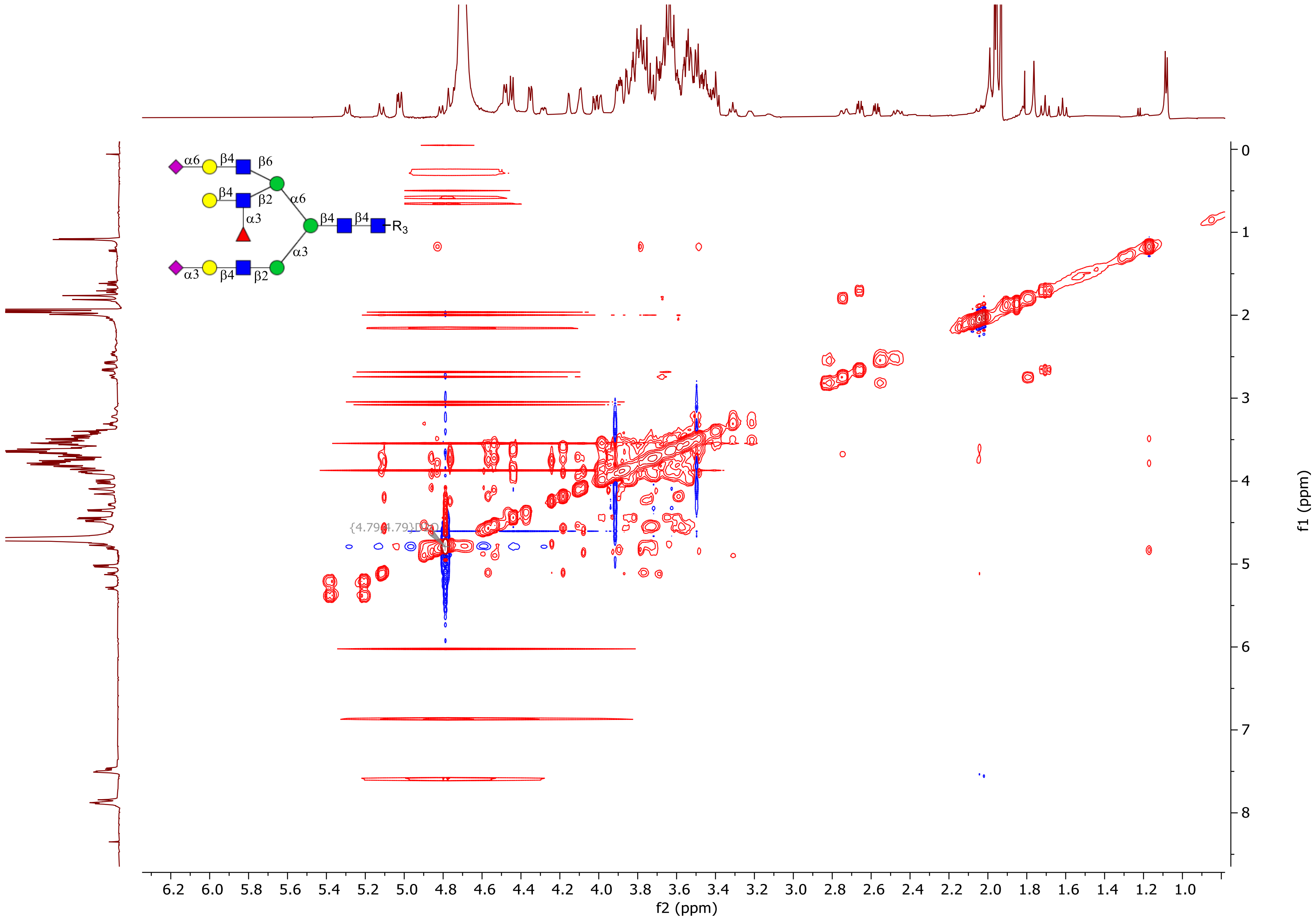

^{1}H NMR of Compound **SI2**

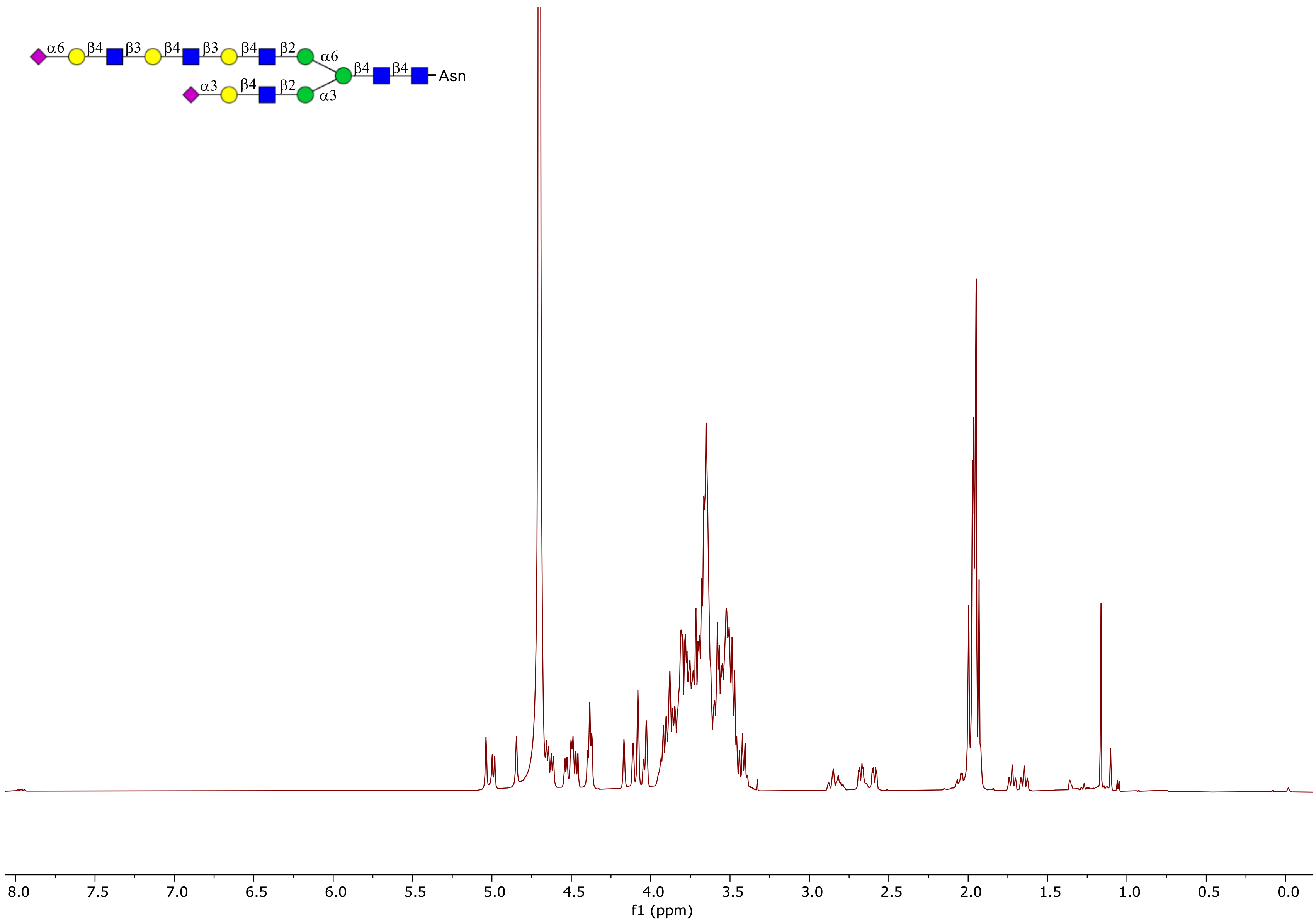

Supplement: Supplementary file 1 [file ja5c22181_si_001.pdf]
